# Ultrasonic Exfoliation of Hydrophobic and Hydrophilic Metal–Organic Frameworks To Form Nanosheets

**DOI:** 10.1002/chem.201803221

**Published:** 2018-11-08

**Authors:** David J. Ashworth, Adam Cooper, Mollie Trueman, Rasha W. M. Al‐Saedi, Liam D. Smith, Anthony J. H. M. Meijer, Jonathan A. Foster

**Affiliations:** ^1^ Department of Chemistry University of Sheffield Sheffield S3 7HF UK

**Keywords:** liquid exfoliation, metal–organic framework, nanosheet, supramolecular chemistry, two-dimensional materials

## Abstract

The modular structure of metal–organic framework nanosheets (MONs) provides a convenient route to creating two‐dimensional materials with readily tuneable surface properties. Here, the liquid exfoliation of two closely related layered metal–organic frameworks functionalised with either methoxy‐propyl (**1**) or pentyl (**2**) pendent groups intended to bestow either hydrophilic or hydrophobic character to the resulting nanosheets is reported. Exfoliation of the two materials in a range of different solvents highlighted significant differences in their dispersion properties, as well as their molecular and nanoscopic structures. Exchange or loss of solvent was found to occur at the labile axial position of the paddle‐wheel based MONs and DFT calculations indicated that intramolecular coordination by the oxygen of the methoxy‐propyl pendant groups may take place. The nanoscopic dimensions of the MONs were further tuned by varying the exfoliation conditions and through “liquid cascade centrifugation”. Aqueous suspensions of the nanosheets were used as sensors to detect aromatic heterocycles with clear differences in binding behaviour observed and quantified.

## Introduction

Metal–organic framework nanosheets (MONs) are free‐standing, nominally two‐dimensional materials formed by the co‐ordination of organic ligands to metal ions or clusters.^1^ A key advantage of MONs over inorganic nanosheets such as graphene, boron nitride and molybdenum disulfide is that their modular structure allows for ready tuning of their properties. This tunability, combined with their large external surface area and high aspect ratio, makes MONs ideal for a diverse range of applications including separation,^2^ sensing,^3^ templation,^4^ electronics^5^ and catalysis.^6^ As with other nanosheets, understanding how to form concentrated suspensions of high aspect ratio nanosheets is an important technological challenge.^7^ The modular structure of MONs potentially provides advantages over simple inorganic nanosheets in allowing easy modification of surface functionalities to enable nanosheets to be designed for use in particular solvents. However, their porosity, flexibility, lability and potential for structural rearrangements also present additional challenges in undertaking this type of study.

Liquid exfoliation provides an attractive, simple and scalable, top‐down approach to producing ultrathin nanosheets from layered materials.^8^ In some cases, immersion of layered MOFs in solvent has been shown to result in spontaneous exfoliation of the materials into nanosheets.^9^ In most cases however, additional energy is required to overcome interlayer interactions in order for exfoliation to occur. A variety of different methods for the liquid exfoliation of MONs have been investigated including ball milling,2b, 10 freeze–thaw^11^ and intercalation,6c, 12 with sonication2b, 10, 13 being the most widely employed approach. In most cases these processes produce a broad distribution of particle sizes. Samples are therefore left to sediment or centrifuged in order to separate out bulk material from the nanosheets. Top‐down approaches are particularly attractive for the study of new systems as the bulk layered materials are typically easier to characterize which aids determining the structure of the nanosheets.

The effect of parameters such as solvent, sonication time and centrifugation time for the liquid exfoliation of other layered materials have been extensively studied and optimized.8a–8c To date, most studies on the liquid exfoliation of MONs have focused on investigating a single framework in a single solvent. Polar solvents such as acetone and alcohols have most commonly been employed. Peng et al. reported a mixture of methanol and propanol as being optimal for exfoliation of a layered ZIF.2b They hypothesize that the small methanol molecules are able to penetrate into layers whilst propanol adsorbs onto the surface of the nanosheets through its hydrophobic tail helping to stabilize the exfoliated nanosheets in suspension. Junggeburth et al. note that their hydrophobic layered MOF showed decreasing exfoliation in THF>toluene>CHCl_3_.^14^ Poor exfoliation was observed when using the polar solvents DMF and H_2_O which was attributed to an inability of the solvents to efficiently penetrate between the hydrophobic interlayer space. In contrast, Moorthy and co‐workers investigated exfoliation of a layered MOF in which there was hydrogen bonding between the layers.^15^ They found a correlation between the Gutmann's hydrogen‐bond‐accepting parameter of the solvent used and the intensity of fluorescence of nanosheets formed following exfoliation. These studies highlight the different roles that different solvent molecules can play in aiding exfoliation of different layered MOFs and stabilizing the resulting nanosheets.

In our work we seek to design new layered MOFs which incorporate features intended to enhance their exfoliation and stabilize the resulting MONs in suspension. We recently communicated a study reporting the liquid exfoliation of Cu(**1**)(DMF), a layered MOF incorporating weakly interacting methoxy‐propyl chains designed to aid exfoliation of the layers into nanosheets.13g The nanosheets are based on the popular metal‐paddlewheel secondary building unit (SBU) which has a labile, Lewis acidic axial coordination site which makes it ideal for a wide range of sensing, catalytic, electronic, separation and storage applications.2c, 3b, 6b, 6c, 6e We hypothesized that liquid exfoliation of layered metal–organic frameworks functionalized with either hydrophobic or hydrophilic functionalities would produce nanosheets with different concentrations, stabilities and thicknesses in different solvents. To investigate this, we compared the liquid exfoliation of the relatively hydrophilic methoxy‐propyl functionalized MOF with an isostructural MOF incorporating a more hydrophobic pentyl‐chain in a wide range of different solvents. We then investigated the molecular and nanoscopic structure of the resulting nanosheets in selected solvents under different conditions in order to understand and optimize the exfoliation process.

## Results and Discussion

### Synthesis of layered MOFs

**Figure 1:**
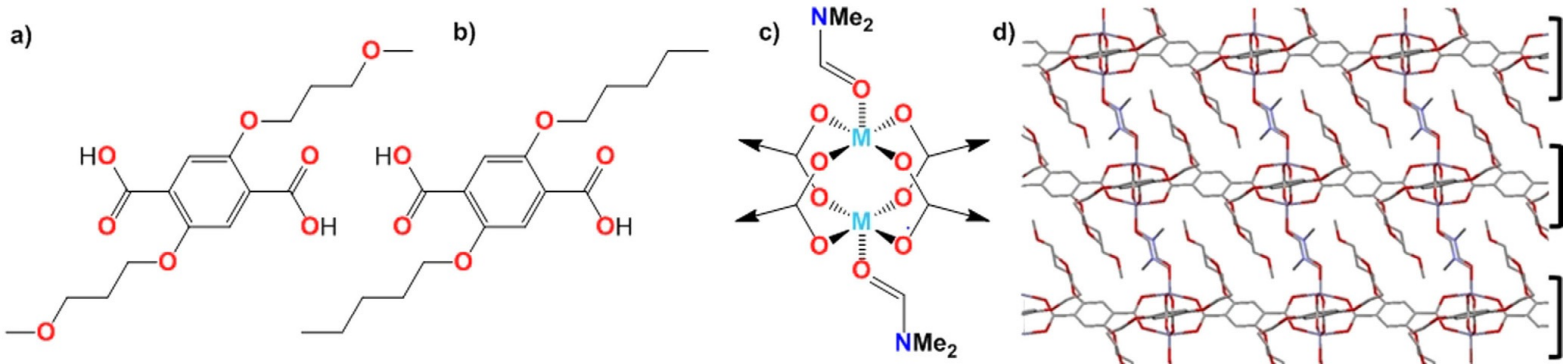
Structure of ligands H_2_ **1** (a) and H_2_ **2** (b). c) Paddlewheel SBU, with DMF coordinated in the axial positions. d) X‐ray crystal structure showing layered structure of Zn(**1**)(DMF).

Compounds H_2_(**1**) and H_2_(**2**) (see Figure 1) were synthesized via Williamson etherification of dimethyl 2,5‐dihydroxy‐1,4‐benzenedicarboxylic acid with 1‐bromo‐3‐methoxypropane and 1‐bromopentane, respectively. The difference in polarity of the ligands was evident during deprotection of the ligands. Compound H_2_ **1** was readily obtained from the corresponding methyl ester by heating under reflux in aqueous NaOH solution.13g Under the same condition only partial deprotection of **2** occurred due to poor solubility so an alternative method involving 1:1 THF/5 % KOH(aq) was employed.^16^ Both compounds were achieved in good yields and the purity of the compounds was established by NMR, mass spectrometry, and elemental analysis.

Heating of H_2_(**1**) or H_2_(**2**) with copper nitrate in DMF in a sealed reaction vial at 110 °C for 18 h resulted in the formation of green microcrystalline powders. Attempts to grow single crystals of these materials were unsuccessful. However, XRPD analysis of the microcrystalline powders indicates these structures are isostructural with the single crystal structure that we have previously reported for Zn(**1**)(DMF).13g In this structure four carboxylate linkers are coordinated to the M_2_‐paddlewheel (PW) while DMF coordinates to the axial sites of the PWs. Importantly, in this form the weakly interacting 3‐methoxypropoxy groups or pentyl chains are positioned between the layers whilst there is strong metal‐carboxylate bonding within the layers. Small differences in the unit cell parameters (Table S1 in the Supporting Information) for the copper complexes are ascribed to the different ligand field effects and different ionic radii of Zn^2+^ and Cu^2+^ and to substitution of the oxygen for a methylene in case of **2**. Elemental analysis is consistent with the proposed formulas and IR and TGA analysis confirms the presence of coordinated DMF in these structures.

### Liquid exfoliation

Exfoliation experiments were undertaken using a bath sonicator. We undertook preliminary experiments investigating the effect of different variables on the degree of exfoliation using DMF and isopropanol as model solvents. Different powers (320 W at 30 % and 100 %), frequencies (37 kHz, 80 kHz) and temperature of sonication were investigated. It was found that high power produced higher concentrations of material in suspension and high frequency increased concentration and avoided dissolution of the nanosheets (Figure S4). Sonication was applied using a sweep mode and samples were rotated through the bath using an overhead stirrer in order to ensure samples were irradiated evenly. Sonication is known to be more effective at lower temperature^17^ and the temperature was maintained over the course of the experiment using a cooling coil giving a temperature of around 16 °C. The set‐up for exfoliation is shown in Figure S2 in the Supporting Information.

The following protocol was therefore established for the exfoliation of the MOFs which was used unless stated otherwise. The layered MOFs were weighed into glass vials to which solvent was added (5 mg in 6 mL) and then exfoliated in a sonicator bath at a frequency of 80 kHz for 30 minutes at a temperature of <20 °C. The samples were then centrifuged at 1500 rpm for 10 minutes to remove larger particles and care was taken to avoid redispersion of the sediment during transport. UV/Vis spectra were measured using the top 3 mL of suspension and highly absorbing samples diluted as required using further solvent.

The solvent that the nanosheets are exfoliated into was expected to have a large effect on the degree of exfoliation and the stability of the resulting suspension. An initial screen of 23 different solvents was undertaken. However, some solvents had to be excluded due to their UV/Vis cut‐off points preventing analysis or their high viscosity resulting in poor dispersion and centrifugation (Table S2 in the Supporting Information). A selection of the 11 solvents representing a diverse range of polarities and chemical functionalities were selected for further investigation: water, dimethylsulfoxide (DMSO), *N*‐Methyl‐2‐pyrrolidone (NMP), dimethylacetamide (DMA), dimethylformamide (DMF), acetonitrile (MeCN), isopropanol (IPA), tetrahydrofuran (THF), diethylether (Et_2_O), cyclohexane and hexane.

Both compounds typically show a single major absorption band the *λ*_max_ of which ranged between 271–303 nm depending on the solvent used (Figure S5 in the Supporting Information). Absorption bands were generally broader and less well defined for Cu(**1**)(DMF), particularly in poorly coordinating solvents such as diethylether, THF and acetonitrile. In acetonitrile, a second local maximum was observed at 361 nm and 304 nm for Cu(**1**)(DMF) and Cu(**2**)(DMF) respectively. The MLCT band was typically too weak and broad to be distinguished so the major peak attributed to the dicarboxylate ligand was used in all subsequent analysis. Neither compound was able to form stable dispersions in either cyclohexane or hexane, nominal values of zero are therefore used for these solvents in the subsequent analysis.

The extinction coefficient for the compounds in each solvent was estimated by dilution of a suspension containing a known mass of each compound. Values ranged from 1892–6693 mol^−1^ dm^3^ cm^−1^ for Cu(**1**)(DMF) to 2467–4489 mol^−1^ dm^3^ cm^−1^ for Cu(**2**)(DMF). These differences in spectra are attributed to exchange of the coordinated DMF, variations in ligand geometry in the different solvents and differences in particle size which are discussed in detail later in this article.

**Figure 2:**
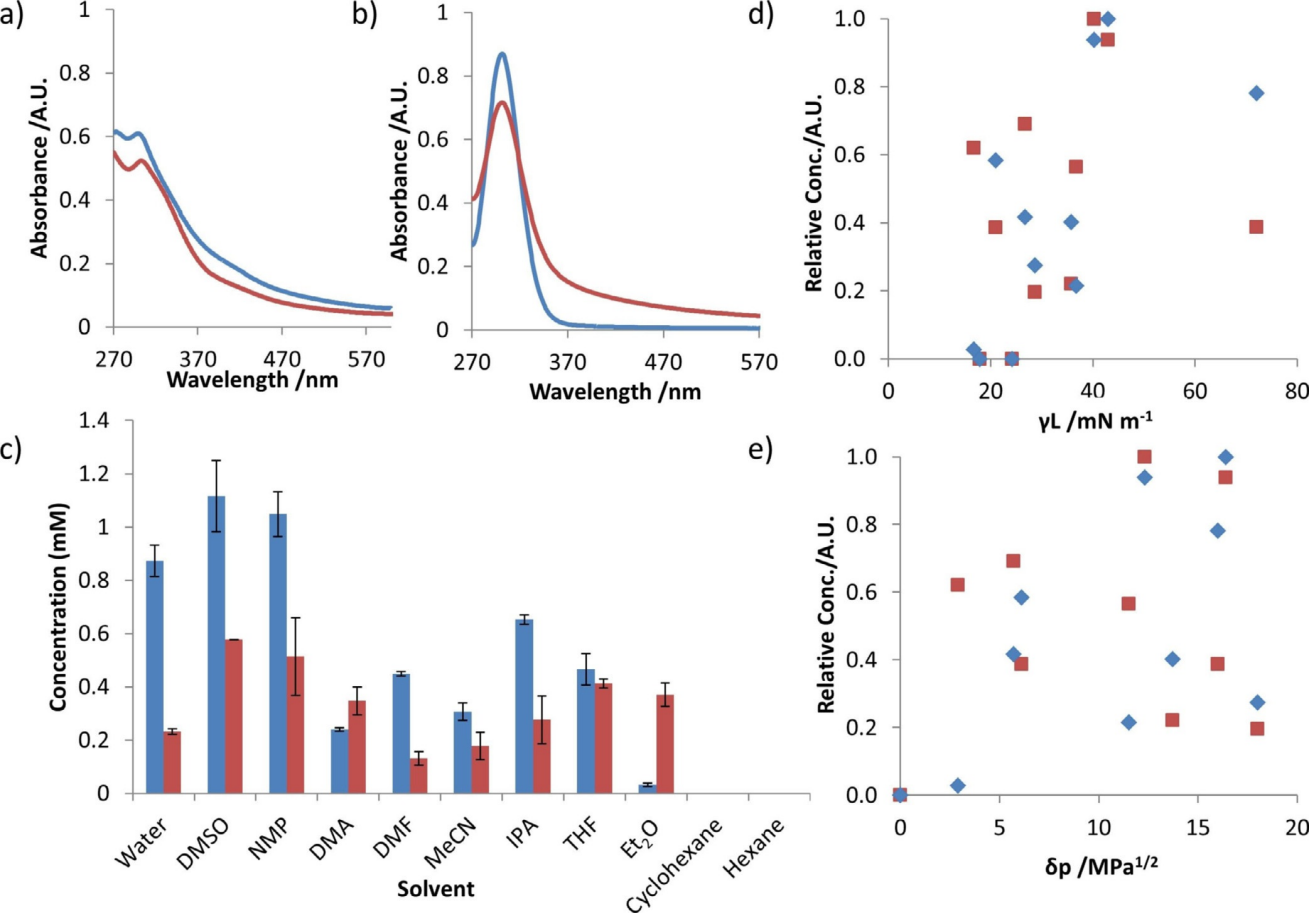
Cu(**1**)(DMF) and Cu(**2**)(DMF) are represented with blue and red data, respectively. a, b) UV/Vis spectral traces of MONs in suspension following exfoliation in DMF (a) and water (b). c) plots of concentrations of nanosheets in suspension, following exfoliation and centrifugation; d, e) normalized concentrations of MON suspensions plotted against the solvent's surface tension (d) and Hansen solubility parameter of energy from dipolar intermolecular force (e).

Clear differences were observed in the concentration of exfoliated material in suspension following sonication and centrifugation of Cu(**1** or **2**)(DMF) in different solvents. Figure 2 shows a plot of the concentration in mm of Cu(**1**)(DMF) [blue] or Cu(**2**)(DMF) [red] suspended in different solvents listed in order of increasing polarity (left to right) as measured by UV/Vis spectroscopy. Data shown are the average of four repeats.

At either extreme, the more hydrophilic Cu(**1**)(DMF) showed a significantly higher degree of dispersion in water than Cu(**2**)(DMF) whilst the opposite is true in diethyl ether where the more hydrophobic Cu(**2**)(DMF) is present at significantly higher concentrations. Higher concentrations are observed for Cu(**1**)(DMF) in all solvents except diethyl ether and DMA. DMSO and NMP give the highest concentrations of both materials and significantly higher than DMA and DMF which have very similar polarities. Samples of both compounds exfoliated into cyclohexane and hexane showed negligible absorbance following centrifugation whilst only Cu(**2**)(DMF) showed any absorbance following exfoliation into toluene.

In studies of other nanosheets formed by liquid exfoliation, a wide range of solubility parameters have been put forward as being important for determining the concentration of exfoliated material in suspension.8b, 8c We plotted the concentration of material in suspension against a range of parameters including polarity, surface tension and Hansen solubility parameters (Figure 2 c–e) as well as Kamlet–Taft, Gutman, Swain, Reichardt's polarity parameters and viscosity (see Section 3.3 in the Supporting Information). The data in these plots is normalized relative to the highest concentration solvent in order to allow easier visual comparison.

In line with similar studies of other nanomaterials, no single parameter by itself was a reliable determinant of the concentration of material left in suspension following exfoliation for either material.8b In many cases, solvents with similar solubility parameters to the best performing solvents showed low concentrations of dispersed materials. For example, the concentration of Cu(**2**)(DMF) exfoliated in DMA is only 20 % of that in NMP even though they have similar surface tensions (*γ*_L_) 36.70 and 40.21 mNm^−1^ respectively. Conversely, water and isopropanol have very different polar Hansen solubility parameters (δp), 16 and 6.1 respectively, but suspensions of Cu(**2**)(DMF) with very similar concentrations are formed. It should be highlighted that the fact that exfoliation of the pentyl functionalized MOF produces stable suspension in water at all, albeit at a lower concentration than the methoxy‐propyl functionalized MOF indicate that they are only “relatively” hydrophobic and hydrophilic. It should also be noted that this experiment provides a comparison of the concentration of material in suspension following exfoliation in different solvents, not necessarily the suitability of the solvents to form nanosheets. A detailed discussion of the nanoscopic dimensions of the materials produced following exfoliation in different solvents is presented in the section entitled nanoscopic analysis later in the paper. First, the differences in UV/Vis spectrum observed for the materials in different solvents also led us to question the composition of the exfoliated material which we discuss in the following section.

### Structural analysis

The relatively labile nature of coordination bonds and the high surface area of the nanosheets mean that it cannot be assumed that the MOF structure is unchanged following liquid exfoliation. In particular, the axial site on the copper paddlewheel is known to be highly labile, allowing for the possibility of loss or exchange of the coordinated DMF molecules with those of the exfoliation solvent. We previously observed differences in the XRPD patterns of Cu(**1**)(DMF) following exfoliation in different solvents.13g Here we undertake a more detailed study to probe the structure of nanosheets of Cu(**1**)(DMF) and Cu(**2**)(DMF) following exfoliation in selected solvents (water, DMF, acetonitrile, NMP and diethylether) representing a range of polarities. The as‐synthesised MOF (5 mg in 6 mL of solvent) was sonicated for 12 h at 80 kHz before centrifugation at 1500 rpm for 1 h and the resulting sediment collected for analysis by using XRPD, IR, TGA and NMR spectroscopy.

The XRPD pattern for Cu(**1**)(DMF) following exfoliation into DMF matches the as‐synthesised compound indicating no structural change occurred. In contrast to this, material analysed following exfoliation in water showed a distinct, new XRPD pattern. For this sample, no nitrogen was observed by elemental analysis while TGA showed a 1.4 % mass loss at 66–94 °C.

Furthermore, the IR pattern shows a loss of the DMF carbonyl peak at 1670 cm^−1^ and a small new peak at 3604 cm^−1^. All these results are consistent with substitution of the axial DMF for H_2_O, giving Cu(**1**)(H_2_O). Material exfoliated in acetonitrile, diethyl ether and NMP all showed correlating peaks in their XRPD patterns corresponding to a third, new phase. In the diethyl ether samples this was accompanied by coincidences with the pattern assigned to Cu(**1**)(H_2_O) indicating a mixture of the desolvated and hydrated phases. In acetonitrile and diethyl ether, negligible weight loss was observed in TGA below the decomposition temperature around 300 °C and elemental analysis showed no nitrogen was present. The same analysis on Cu(**1**)(DMF) exfoliated in NMP shows a mass loss of 4.2 % at 83–205 °C, and small quantities of nitrogen (0.72 wt %) indicating a small amount of non‐coordinated solvent is present. We suggest this new material (formed in acetonitrile, diethyl ether and NMP) is caused by the loss of axial DMF to give a desolvated phase with the structure Cu(**1**). This matches previous findings following exfoliation in acetone and methanol.13g

**Figure 3:**
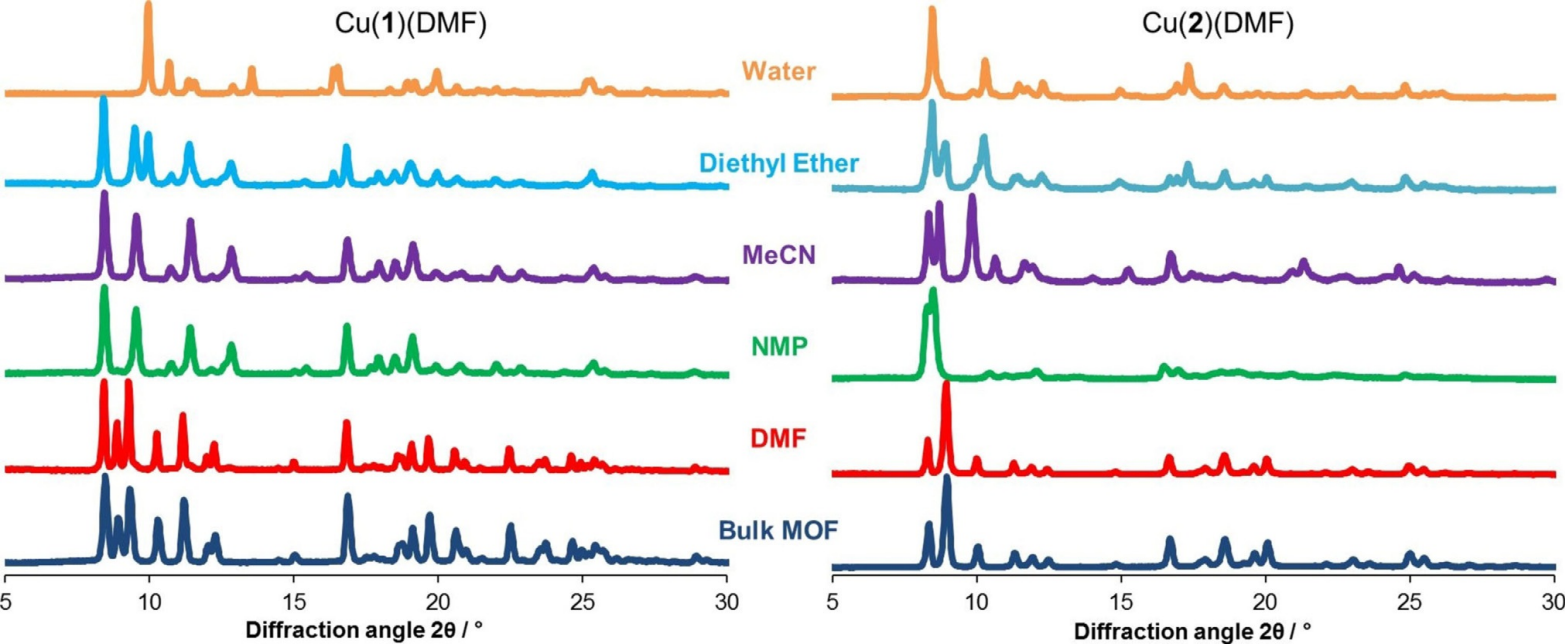
Experimental powder diffraction patterns of Cu(**1**)(DMF) and Cu(**2**)(DMF) as‐synthesised (dark blue), and of post‐exfoliation solids recollected through centrifugation for 1 h at 1500 rpm, in DMF, NMP, MeCN, diethyl ether and water.

Samples of Cu(**2**)(DMF) exfoliated into DMF generated XRPD data correlating with the pattern produced from the parent MOF. Exfoliation in water produced a powder pattern corresponding to a distinct phase. This fact, along with the absence of nitrogen in the elemental analysis and mass loss of 4.6 % at 23–107 °C shown by TGA, is consistent with the formation of Cu(**2**)(H_2_O). In a divergence from the behaviour shown by Cu(**1**)(DMF), exfoliation of Cu(**2**)(DMF) into diethyl ether, acetonitrile and NMP gave materials which showed weak correlation in peak positions between the resulting XRPD patterns (Figure 3). Exfoliation into diethyl ether gave rise to a pattern in which each peak could be assigned to either Cu(**2**)(DMF), or to the phase assigned to Cu(**2**)(H_2_O). Elemental analysis concluded a value of 0.59 wt % nitrogen (in comparison to 2.99 wt % calculated for Cu(**2**)(DMF), which is consistent with incomplete removal of DMF and partial substitution by trace quantities of water. In contrast to this, elemental analysis of the sample produced through exfoliation in acetonitrile showed no detectable nitrogen and TGA showed no mass loss. We therefore assign this powder pattern as corresponding to that of the desolvated materials. Elemental analysis of the Cu(**2**)(DMF) exfoliated into NMP indicates significant levels of nitrogen present (2.21 %) and a decrease in mass at around 105 °C consistent with loss of co‐ordinated solvent, on heating the sample. Proton NMR of the digested samples confirmed the presence of residual DMF, and ruled out substitution by NMP. The two large, but poorly resolved peaks around 8° in the powder pattern are consistent with the formation of the sql topology and the distortions are presumed to be due to partial desolvation.

### DFT modelling

In order to gain further insights into the structure of the different phases, we undertook DFT modelling to visualize the structure of the MONs and confirm the phase assignments. Structures of **1** and **2** were initially modelled using a single PW formed using model monocarboxylate ligands functionalized with only a single methoxy propyl‐ or pentyl‐ chain to speed up the calculation (1* and 2*). Previous studies by us of PW MOFs have shown that using isolated unit‐cells produces very comparable results to calculations performed on extended structures.^18^ Coordinates from the known crystal structure of Zn(**1**)(DMF) were used to generate starting coordinates. The structure was then modified, replacing DMF with water and acetonitrile. The fourth iteration removed any solvent from the axial position. In this final iteration we manipulated the arms, so that the ether functionality could conceivably coordinate in the axial position. For **2** the same procedure was followed. The functional used was B3LYP^19^ with dispersion‐corrections due to Grimme (GD3‐BJ). Structures of 1* were subsequently remodelled with both methoxy‐propyl chains (1**) resulting in slight improvements in the correlation between the calculated and experimental data, but showed no substantive differences. For further details, please see the supporting information.

**Figure 4:**
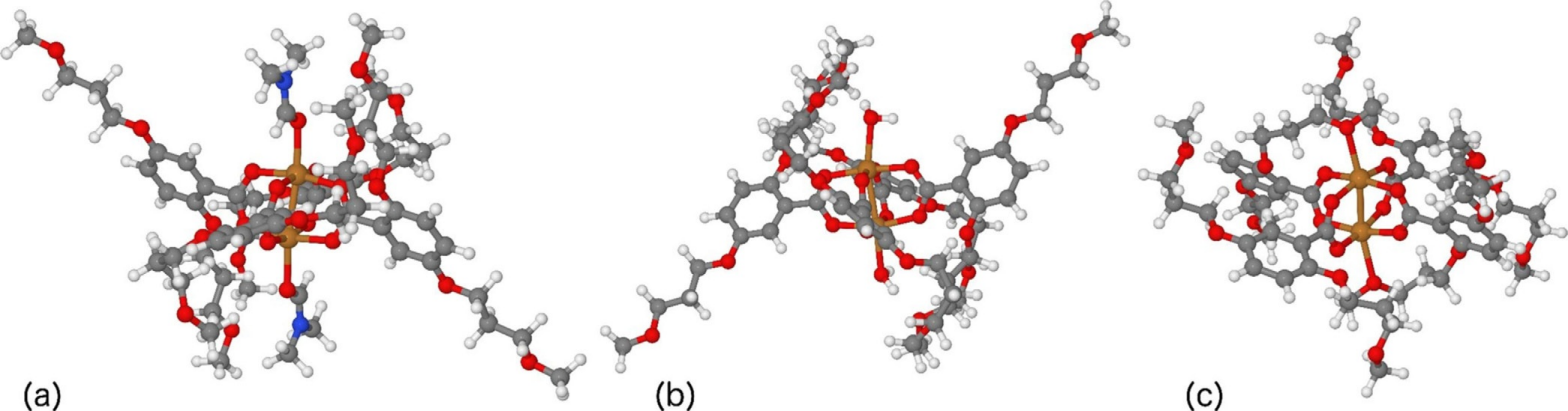
DFT calculations showing optimised structures for (a) Cu_2_(1**)_4_(DMF)_2_, (b) Cu_2_(1**)_4_(H_2_O)_2_, (c) Cu_2_(1**)_4_ where 1** is 2,5‐Bis(3‐methoxypropoxy)benzoate.

Figure 4 a–c shows images of the relaxed structures for the three different phases obtained with 1** in which DMF, water and no‐solvent are coordinated at the axial position, respectively. Similar images are shown for the other derivatives in Figure S54 in the Supporting Information. The corresponding calculated IR patterns for these structures were compared with the experimental patterns (Figures S55–57). Whilst there are some significant shifts in peak position and intensity between the calculated and experimental patterns particularly in the fingerprint region, the presence or absence of characteristic solvent peaks could be used to assign the phases. In particular, characteristic peaks corresponding to the carbonyl of the coordinated DMF molecules at 1706 cm^−1^ and of water around 3500 cm^−1^ were observed in the corresponding calculated and experimental patterns for material exfoliated in DMF and water, respectively. Experimental patterns for material exfoliated in acetonitrile lacked the calculated peaks for acetonitrile at 2200 cm^−1^ as well as those for water and DMF and provided closer matches to the calculated structure with no solvent coordinated. This data therefore supports the assignments given in the previous section.

Coordination of two acetonitrile, ethanol, acetone, DMF and water molecules to Cu(1**) have binding energies of 58, 66, 72, 87 and 119 kJ mol^−1^, respectively, relative to three infinitely separated molecules. This broadly corroborates what is observed experimentally in that more weakly bound solvents such as acetonitrile are lost whilst more strongly coordinating solvents such as DMF and water are retained. However, it should be noted that these values are based on gas phase calculations and so do not take into account solvent–solvent interactions. This may account for discrepancies such as our previous observation that Cu(**1**)(DMF) is the observed structure in 10 % DMF in water mixtures.

It is interesting to note that in the calculated structures obtained for Cu(1**), methoxy propyl chains on either side of the PW are bent over to allow the lone pair of the oxygen to coordinate intramolecularly to the axial positions of the complex. This is not observed in the structure for Cu(2*) where the oxygen is replaced with a methylene group. The binding energy for a single arm coordinating to Cu(1**) (as calculated through the difference between the energies of structures with one coordinated or uncoordinated arm) is 30 kJ mol^−1^. In our calculations coordination of the second arm only has a binding energy of 7 kJ mol^−1^. It should be noted that these calculations are highly dependent on the confirmation around the paddle wheel and a full conformational search would be required to provide a better estimate of the true value for the intramolecular binding which is beyond the scope of this study.

We therefore suggest that this ability of the methoxy‐propyl chains, but not the propyl chains, to intramolecularly coordinate to this axial position with values comparable to those of some solvent molecules may provide at least a partial explanation for some of the differences observed between the nanosheets. For example, the co‐ordinated methoxy‐propyl chains make the surface of the Cu(**1**) structures less polar resulting in high concentrations of nanosheets in apolar solvents than might otherwise be expected. Similarly, the flexibility of the frameworks might reduce the impact of the hydrophobic pentyl chains in polar solvents. These structural insights highlight the challenges of predicting and understanding the effects of even small changes in molecular structure on the macroscopic properties of the nanosheets.

### Nanoscopic analysis

**Figure 5:**
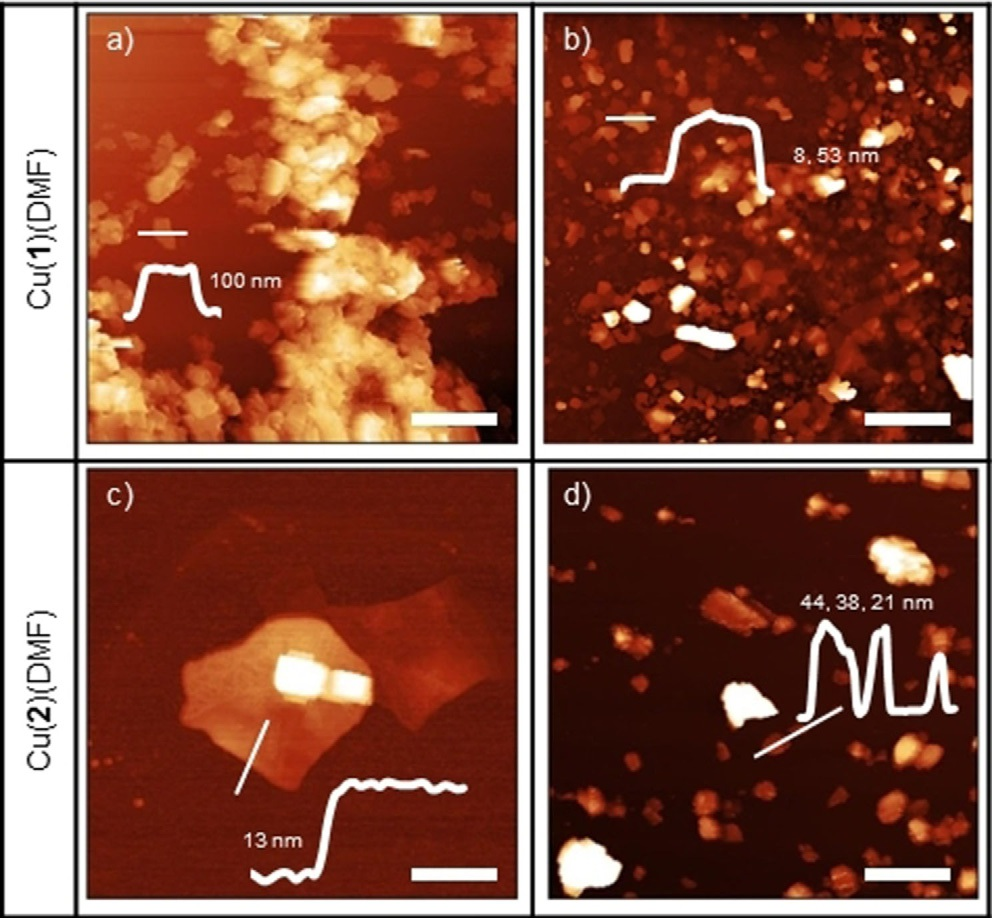
AFM images of Cu(**1**)(DMF) exfoliated in water for different time periods: a) 30 min, b) 12 h. AFM images of Cu(**2**)(DMF) exfoliated in different solvents: c) water and d) diethyl ether (c and d, respectively) for 12 h. Scale bars are 2, 2, 2 and 1 μm, and height scales are 1000, 200, 50 and 150 nm for a–d, respectively.

In addition to understanding the effect of solvent on the molecular structure of the nanosheets, we sought to examine the influence of solvent on the nanoscopic structure of the resulting material. Exfoliation protocols for other layered materials have varied significantly, with sonication times ranging from 20 min to several days. Here, we first investigated the exfoliation of the hydrophilic Cu(**1**)(DMF) and hydrophobic Cu(**2**)(DMF) in water and diethyl ether, using two exfoliation time periods: 30 min and 12 h. It was hypothesized that longer exfoliation times would lead to thinner nanosheets being produced, and anticipated that the Cu(**1**)(DMF) would exfoliate better in H_2_O than diethyl ether, and the reverse true for Cu(**2**)(DMF). After exfoliation, centrifugation at 1500 rpm for 10 min removed large, unexfoliated material, and AFM was used to assess nanosheets produced (see Figure 5.)

Both exfoliation procedures resulted in nanosheets with varying size distributions. In general, more nanosheets with smaller heights were observed from 12 h exfoliation than 30 min, suggesting that longer exposure to ultrasonic waves results in increased exfoliation. For example, Cu(**2**)(DMF) in diethyl ether exfoliated for 30 min and 12 h resulted in nanosheets with thicknesses of 20–100 and 20–50 nm, respectively. Selected examples of nanosheets observed using AFM can be found in Figure 5, and additional figures found in the Supporting Information (Figures S13–19). There are noticeably large agglomerates and sheet‐like particles with heights over 100 nm in many of these images, suggesting that 10 min centrifugation at 1500 rpm is not effective at removing all larger particles from the post‐sonication suspension.

In order to compare the effect of solvent on the nanoscopic dimensions of the nanosheets formed, Cu(**1**)(DMF) and Cu(**2**)(DMF) were exfoliated for 12 hrs in water, DMF, NMP, acetonitrile and diethyl ether. Samples were centrifuged at 1500 rpm for 1 h as longer/ faster centrifugation times resulted in insufficient material for analysis in some solvents. Typical AFM images of observed nanosheets can be found in Figures S27–36.

In general, exfoliation in DMF and NMP resulted in nanosheets of low quality—lateral dimensions and aspect ratios were low, with observed particles having relatively large heights of >40 nm. Particles appeared to be rounded in nature, rather than lamellar, particularly in NMP. This could suggest that the energetic input upon prolonged exposure times to ultrasound facilities MON breakdown and dissolution of ligand and Cu into solution–both H_2_ **1** and H_2_ **2** are soluble at these low concentrations in DMF and NMP.

We investigated the stability of the nanosheets in DMF over 5 days by UV/Vis spectroscopy and found broadening of the ligand absorption band which was attributed to the formation of a new peak corresponding to the neutral ligand (Figure S13 a,b in the Supporting Information). In contrast, material exfoliated in water and diethylether showed no shift in absorbance maximum over time. Furthermore, the intensity of these bands remained constant over 5 days indicating that stable suspensions had been formed (Figure S13 c).

**Figure 6:**
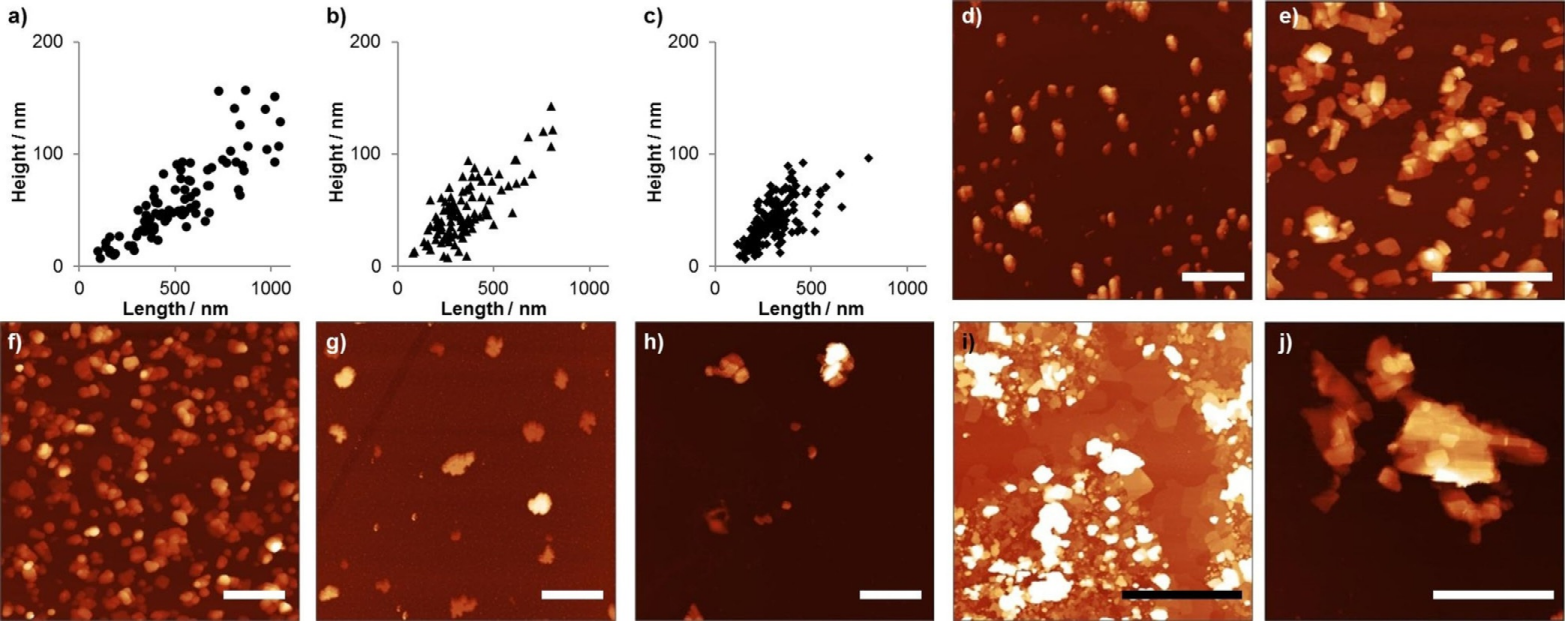
Scatter plots of height and lateral dimensions of Cu(**1**)(DMF) nanosheets observed from exfoliation in MeCN and cascade centrifuged for 1 h at 1500 rpm (a), then 30 min at 4500 rpm (b), then 4 hrs at 4500 rpm (c). Topographical AFM images of Cu(**1**)(DMF) (d) and Cu(**2**)(DMF) (e) exfoliated for 12 hrs, and Cu(**2**)(DMF) exfoliated for 30 min (f) and centrifuged at 4500 rpm for 4 hrs. AFM images of Cu(**1**)(DMF) exfoliated in water (g) and diethyl ether (h), and Cu(**2**)(DMF) exfoliated in water (i) and diethyl ether (j). AFM scale bars are 2 μm, and height scales are 150 nm (d, e, j), 80 nm (f), 40 nm (g), 200 nm (h), and 50 nm (i).

Nanosheets of Cu(**2**)(DMF) exfoliated in water were angular and typically <1 μm laterally with heights 10–30 nm. Some examples of ultrathin flakes of 5 μm*×*2 nm were observed (Figure 6). Nanosheets of Cu(**1**)(DMF) exfoliated in H_2_O were more irregularly shaped and typically 10–40 nm in height, with lateral dimensions up to 1.5 μm, consistent with our previous report.13g Exfoliation of Cu(**1**)(DMF) in diethyl ether produced low concentrations of materials in suspension and the nanosheets observed have relatively low aspect ratios, typically 50–100 nm in height and <600 nm laterally. In contrast, Cu(**2**)(DMF) exfoliated in diethyl ether produces nanosheets which were typically <40 nm with examples observed below 10 nm thickness and with lateral dimensions up to 2 μm (Figure S37 in the Supporting Information).

It is interesting to note the more hydrophobic ligand **2** produced nanosheets with higher aspect ratios and more regular shapes than those of the hydrophilic ligand **1** in both water and diethylether. This is contrary to our expectation that closer matching of the solvent and nanosheet properties would lead to thinner nanosheets. An alternative explanation might be that the thinner nanosheets formed from Cu(**2**)(DMF) are the result of weaker interlayer interactions between the pentyl chains compared to the methoxy‐propyl chains aiding exfoliation during sonication. Another factor to consider is that poorer interactions between the nanosheets and solvent may result in more of the thicker nanosheets produced during sonication being removed from suspension during centrifugation. This would mean that on average thinner nanosheets are observed when there is a mismatch in solvent and nanosheet properties. Optimising nanosheet design must therefore balance minimizing inter‐layer interactions with complimenting solvent properties to form stable dispersions of nanosheets and developing centrifugation protocols that ensure removal of larger particles.

In order to investigate nanosheet size control, Cu(**1**)(DMF) was selected as a test system, and exfoliated in acetonitrile for 12 h. Acetonitrile was chosen as we observed good particle separation and minimal agglomeration upon deposition for AFM analysis using this solvent, which enabled more accurate sizing of nanosheets. Liquid cascade centrifugation (LCC) is a versatile strategy that uses multiple sequential centrifugation steps of increasing rate or time period, using the supernatant of the previous step as the suspension for the next, in order to remove particles of various size from suspension.^20^ We employed LCC using steps of 1500 rpm for 1 h, 4500 rpm for 30 min then 4500 rpm for 4 h. The particle size distribution of the resulting nanosheets as determined through a statistical analysis (*n*=94–161) can be seen in Figure 6 a–c and the mean (x̄) and standard deviation (SD) in particle size are summarized in Table 1. AFM images used for these analyses can be found in the Supporting Information (Figures S20–22).

**Table 1:** Statistics calculated from nanosheets produced from the 12 h exfoliation of Cu(**1**)(DMF) and Cu(**2**)(DMF) in acetonitrile, and cascade centrifuged.

Sample	Cu(**1**)(DMF)			Cu(**2**)(DMF)	
Centrifugation Cycle	1500 rpm, 1 h	4500 rpm, 30 min	4500 rpm, 4 h	1500 rpm, 1 h	4500 rpm, 4.5 h
*n*	95	111	161	94	134
Conc. [mm]	0.33	0.12	0.09	0.12	0.04
*x̄* LD ±SD [nm]	512±234	347±154	307±108	348±202	367±155
*x̄* H ± SD [nm]	59±35	49±26	41±19	20±12	19±9
H range [nm]	7–157	8–143	6–96	5–64	4–58
LD range [nm]	100–1050	80–810	120–800	80–830	120–820

*n*=number of analysed nanosheets, conc.=concentration, determined through UV/Vis spectroscopy, *x̄*=mean, SD=standard deviation, LD=lateral dimension (recorded as the largest lateral vector across a nanosheet), H=height. See the Supporting Information for AFM images used.

The results of the statistical analyses show that the average nanosheet thickness and length of Cu(**1**)(DMF) decrease sequentially from 59×512 nm to 41×307 nm between the first and last steps due to the removal of larger particles. This correlates with a decrease in the concentration of material in suspension from 0.33 mm to 0.09 mm. The smallest nanosheets observed in each case are of a similar size at 6–8 nm. The concentration of Cu(**2**)(DMF) in suspension following the final centrifugation step is lower than for Cu(**1**)(DMF), however the nanosheets are significantly thinner and larger than Cu(**1**)(DMF) with minimum thicknesses of 4 nm and average dimensions of 19×367 nm following the final step.

DLS data were also collected for both systems after each of the three steps of LCC (Figures S28–S29 in the Supporting Information). The trend observed by DLS is consistent with that observed by AFM in that LCC lowers the average particle diameter of the MONs by reducing the number of larger particles remaining in the supernatant. However, the diameters determined by DLS are consistently lower (Table S9) than those obtained in the AFM analysis. For example, the mean LD for Cu(**1**)(DMF) exfoliated in acetonitrile for 12 h followed by the three steps of LCC is measured as 106 nm by DLS and 307 nm by AFM. Obtaining accurate particle size measurements from high aspect ratio nanosheets using DLS is known to be problematic as the Stokes‐Einstein equation assumes spherical particles^21^ and previous comparisons have also shown DLS produces lower average particle sizes than AFM.^22^

Exfoliation by sonication is recognized to be an effective delaminative technique. For MONs, long exfoliation times at low temperatures produce more, thinner nanosheets. Solvent choice is important in determining the thickness and morphology of the nanosheets obtained and avoiding dissolution over time. Small differences in ligand too can have a significant impact on the strength of interlayer interactions. Complimentary solvents may play a role at weakening interlayer interactions and aiding exfoliation. However, poor matching of solvent‐nanosheet interactions may also result in thinner nanosheets being observed as thicker nanosheets are removed from solution by centrifugation. The wide distributions of particle sizes that result from prolonged exposure of the bulk MOF to ultrasonic waves can be narrowed through LCC and the average particle size reduced. Controlling the centrifugation rate enables nanosheet size distribution to be optimized for particular applications. In some applications having a narrow distribution of ultrathin nanosheets will be essential, for others having a broader distribution of thicker nanosheets at a higher concentration could be more important.

### Sensing

We have previously reported the sensing of the small aromatic heterocycle pyridine from aqueous solution, using aqueous suspension of Cu(**1**)(H_2_O) nanosheets. Titration of pyridine was found to bind to the axial position of the Cu_2_‐paddlewheel, with a *K_a_* of 30±8 m ^−1^. When this experiment was replicated, instead using Cu(**2**)(H_2_O), a drop‐off in absorbance at *λ*_max_ was observed, as well as the suspension of nanosheets visibly turning cloudy upon addition of pyridine. This could be attributed to agglomeration of nanosheets upon addition of pyridine, which displaces coordinated H_2_O. This would render the MON surface increasingly hydrophobic, which may cause agglomeration.

In order to be able to compare the binding strength of Cu(**1**) and Cu(**2**) MONs, imidazole was selected as a more hydrophilic binding substrate to prevent agglomeration. Cu(**1**)(DMF) and Cu(**2**)(DMF) were exfoliated in water for 12 h and centrifuged at 1500 rpm for 1 h to give suspensions with concentrations of 0.65 and 0.2 mm respectively. The samples were diluted with water and aliquots of the guest substrate (73 mm and 43 mm for Cu(**1**)(H_2_O) and Cu(**2**)(H_2_O), respectively) in aqueous host suspension (0.13 mm Cu(**1**)(H_2_O) and 0.08 mm (Cu(**2**)(H_2_O)) were titrated into host suspension and monitored using UV/Vis spectroscopy. Addition of imidazole in both cases resulted in bathochromic shifts of *λ*_max_ from 301‐297 nm and 42 % and 36 % increases, respectively, in the absorption intensity (Figures S48 and S51 in the Supporting Information). These changes are consistent with expected substitution of water molecules for imidazole at the axial positions of the Cu_2_‐paddlewheel, which would result in changes to the absorption band of the coordinated dicarboxylate ligands **1** and **2**. It is most likely that imidazole binds to the Cu atoms through the *sp* ^2^‐hybridised N electron pair donation.

This data was used to calculate binding constants of *K*_a_=1370±180 and 1950±140 m ^−1^ for imidazole to Cu(**1**)(H_2_O) and Cu(**2**)(H_2_O) respectively. The 43 % increase of *K*_a_ observed between Cu(**1**) and Cu(**2**) is consistent with the hypothesis of the terminal methoxy oxygen of the ligand alkyl‐ether arm in **1** being able to bind to the axial Cu sites, as this would provide an extra competing species for substrate coordination in Cu(**1**)(H_2_O) which is not present in Cu(**2**)(H_2_O), which could explain why imidazole binds more strongly to Cu(**2**)(H_2_O).

## Conclusions

MONs are an emerging class of two dimensional materials with significant potential for use in a wide range of applications thanks to their tuneable structure, high surface area and nanoscopic dimensions.^1e^ Liquid exfoliation using ultrasound is an appealing route to generating nanosheets from layered MOFs thanks to its broad applicability to different systems, the wide availability of ultrasonic baths and scalability of the approach. However, there have so far been few studies investigating the impact of ligand design, solvent choice and exfoliation conditions on the molecular and nanoscopic structures of the nanosheets formed and their stability in suspension.

We investigated two layered Cu‐PW based MOFs formed using dicarboxylic acid ligands functionalised with either methoxy‐propyl or pentyl pendant groups intended to bestow hydrophilic and hydrophobic character, respectively. Exfoliation of Cu(**1**)(DMF) using an ultrasonic bath produced higher concentrations of material suspended in water than diethylether whilst the opposite trend was observed for Cu(**2**)(DMF). Cu(**1**)(DMF) typically showed higher dispersed concentrations than Cu(**2**)(DMF) and NMP and DMSO gave the highest overall concentrations for both compounds. Exfoliation in a wide range of other solvents showed significant differences in the degree of exfoliation between the two compounds, however this was not found to correlate with any single solvent parameter.

The lack of simple correlation was partially explained by solid state analysis which showed that whilst the two‐dimensional connectivity of the layered MOFs is maintained following exfoliation, the presence of a labile axial site on the Cu‐PW SBUs mean that the surface functionalization of the nanosheets can vary depending on the exfoliation solvent. This effect is not typically observed in simple inorganic nanosheets but is likely to be common amongst MONs with exchangeable metal sites. DFT analysis indicated that the oxygen of the methoxy‐propyl ligand **1** is able to coordinate intramolecularly to the axial position of the copper paddlewheels. This may further explain the complex dispersion behaviour of the MONs.

The nanoscopic dimensions of the exfoliated material were investigated using AFM and nanosheets with thickness as low as 2 and 10 nm were observed. Cu(**2**)(DMF) typically formed nanosheets which were thinner, had higher aspect ratios and were more angular than those of Cu(**1**)(DMF) in both water, diethylether and acetonitrile. This is hypothesized to be the result of the apolar pentyl chains resulting in weaker interlayer interactions than those of the methoxy‐propyl chains aiding exfoliation during sonication. However, as with the dispersion study, a complex balance of sometimes competing factors will determine the profile of the nanosheets generated. Longer exfoliation times typically produced higher concentrations of thinner nanosheets whilst liquid cascade centrifugation could be used to remove larger particles and narrow the size distribution.

The ability of the axial position to exchange solvent molecules and the photophysical properties of the nanosheets were exploited for use as sensors. Addition of pyridine resulted in aggregation of Cu(**2**) but not Cu(**1**) whilst imidazole was shown to bind significantly stronger to Cu(**2**) than Cu(**1**). We note that the weaker binding seen for Cu(**1**) may be in part due to competition from intramolecular binding by the oxygen of the methoxy‐propyl chain.

Overall, this study demonstrates the potential of the modular structure of MONs in allowing systematic tuning of their surface properties through isoreticular substitutions. It also highlights the subtle interplay between ligand, metal cluster, solvents and exfoliation conditions in determining the molecular, nanoscopic and macroscopic structure and properties of nanosheets. Only by better understanding these structure–property relationships will we be able to harness the potential of MONs for use as sensors, catalysts and for processing into composite materials for separation and electronics applications.

## Experimental Section

### Synthesis

Commercial solvents and reagents were used without further purification. Synthesis of organic ligands was carried out in dry glassware with a nitrogen overpressure. Solvothermal synthesis of MOFs was undertaken using borosilicate vials with Teflon faced rubber lined caps.

Dimethyl 2,5‐dihydroxyterephthalate and 2,5‐Bis(3‐methoxypropoxy)‐1,4‐benzenedicarboxylate (**1**) were synthesised according to previously reported procedures.^23^ 2,5‐Bis(pentoxy)‐1,4‐benzenedicarboxylate was similarly synthesised, however the hydrolysis of the protected acid groups was achieved instead through refluxing in THF with aq. KOH (5 %). See Section 1.1 in the Supporting Information for details and full materials characterisation.

Cu(**1**)(DMF) was synthesised according to our previous method⋅13g Cu(**2**)(DMF) was similarly synthesised. Specifically, Cu(NO_3_)_2_.6H_2_O and ligand H_2_ **1** or H_2_ **2** were dissolved in DMF and sealed into reaction vials, and heated to 110 °C for 18 hrs, then slow‐cooled, resulting in a 77 % yield of green, microcrystalline Cu(**2**)(DMF). Synthetic details and characterisation including elemental analysis, FTIR, TGA and PXRD can be found in the Supporting Information.

### Exfoliation

MOF and solvent were added to 10 mL reaction vials in the quantities stated in‐text. These were rotated using an adapted Heidolph RZR 2020 overhead stirrer with a multi sample holder, in a Fisher brand Elmasonic P 30H ultrasonic bath (2.75 L, 380/350 W, UNSPSC 42281 712) filled with water. The ultrasonic bath was operated at 100 % power, at 80 kHz, and was fitted with a cooling coil so as to prevent bath heating upon prolonged exfoliation times.

### Characterisation

NMR spectra were recorded on a Bruker Advance DPX 400 spectrometer. ^1^H and ^13^C chemical shifts are reported in ppm on the δ scale and were referenced to the residual solvent peak. All coupling constants are reported in Hz. Mass spectra were collected using an Agilent 6530 QTOF LC‐MS in positive ionization mode. Elemental analyses were obtained on an Elementar vario MICRO cube. X‐Ray powder diffraction patterns were collected using a Bruker D8 Advance powder diffractometer equipped with a copper k_α_ source (*λ*=1.5418 Å) operating at 40 kV and 40 mA. The instrument was fitted with an energy‐dispersive LYNXEYE detector. IR spectroscopy was performed on a PerkinElmer ATR‐FTIR Spectrum 2. Thermogravimetric analyses were collected using a PerkinElmer Pyris 1 TGA from 30–600 °C at 10 °C min^−1^, under a 10 cm^3^ min^−1^ flow of nitrogen. UV/Vis absorption spectra were obtained on a Varian Cary 50 UV or Varian Cary 5000 UV/Vis‐NIR spectrophotometer, using standard 1 cm width quartz cells and PerkinElmer Spectrum One software. The nanoscopic morphology of the samples was investigated using a Bruker Multimode 5 AFM with an equipped Nokia 10x visualising lens, operating in soft tapping‐mode using Bruker OTESPA‐R3 cantilever. Samples were prepared by dropping 10 μL (sample dependant) of suspension onto a freshly cleaved mica substrate. Images were processed using standard techniques with Gwyddion software. DLS data were collected using a Malvern Zetasizer Nano Series particle size analyser equipped with a He‐Ne laser at 633 nm, operating in backscatter mode (173 °).

### DFT modelling

All calculations were performed using Gaussian 09, version D.01.^24^ The functional used was B3LYP.^19^ For all atoms the 6–311G** basis set was used^25^ apart from Cu, for which we used the SDD pseudo‐potential [SDD]. All calculations were run with ultrafine integrals ignoring any potential symmetry in the calculations. All optimizations were performed with the standard parameters as implemented in G09. All systems were assumed to be dry, so that no additional solvent field was included. For all optimized structures, frequencies were calculated in the harmonic approximation. In a few cases a small (between 0 and −10 cm^−1^) imaginary frequency was found, which was subsequently ignored, following standard practice, since these are usually caused by quadrature errors. For all comparisons between theory and experiment presented below, a scaling factor of 0.973 was used for values below 2000 cm^−1^, while for values above 2000 cm^−1^ a scaling factor of 0.95 was used. It is noted that in previous work it was found that using a single PW to describe a 2D structure resulted in a reasonable agreement between theory and experiment.^26^ The computational part of the Supporting Information was created using in‐house developed software based on the OpenEye toolkit.

## Conflict of interest

The authors declare no conflict of interest.

## Supporting information

As a service to our authors and readers, this journal provides supporting information supplied by the authors. Such materials are peer reviewed and may be re‐organized for online delivery, but are not copy‐edited or typeset. Technical support issues arising from supporting information (other than missing files) should be addressed to the authors.

Supplementary
