# Supplementary material for: Ultrasonic Exfoliation of Hydrophobic and Hydrophilic Metal–Organic Frameworks To Form Nanosheets
Source: Chemistry. 2018 Nov 8;24(68):17986–96. doi: 10.1002/chem.201803221 (PMC6348380; doi:10.1002/chem.201803221)
Supplement: Supplementary file 1 — Supplementary [file CHEM-24-17986-s001.pdf]

CHEMISTRY
A European Journal

Supporting Information

Ultrasonic Exfoliation of Hydrophobic and Hydrophilic Metal–Organic Frameworks To Form Nanosheets

David J. Ashworth, Adam Cooper, Mollie Trueman, Rasha W. M. Al-Saedi, Liam D. Smith, Anthony J. H. M. Meijer, and Jonathan A. Foster*[a]

chem_201803221_sm_miscellaneous_information.pdf

Contents

1. General Experimental 2
2. MOF Syntheses and Characterisation 3
3. UV-Vis Studies 5
   3.2 Molar Extinction Coefficients 9
   3.3 Dispersed Concentration vs Solvent Parameter Plots 10
   3.4 Stability Tests 15
4. Atomic Force Microscopy 17
   4.1 Method Development 17
   4.2 Particle Sizing Studies 21
   4.4 DLS Studies 27
   4.5 Exfoliation Studies in Various Solvents 29
5. Structural Analysis 34
   5.1 Powder X-Ray Diffraction 34
   5.2 Fourier Transform Infrared Spectroscopy 35
   5.3 Thermogravimetric Analysis 36
   5.4 Elemental Analysis 39
6. Binding Studies 40
   6.1 Estimation of Nanosheet Concentration in Suspension 40
   6.2 Binding Studies 40
7. References 44

2. General Experimental

Commercial solvents and reagents were used without further purification, solvents used for UV-vis studies were HPLC or spectroscopic grade. Synthesis of organic ligands was carried out in dry glassware with a nitrogen overpressure. Solvothermal synthesis of metal-organic frameworks was undertaken using borosilicate vials with Teflon faced rubber lined caps.

NMR spectra were recorded on a Bruker Advance DPX 400 spectrometer. Chemical shifts for 1H and 13C are reported in ppm on the δ scale; 1H and 13C chemical shifts were referenced to the residual solvent peak. All coupling constants are reported in Hz. Mass spectra were collected using an Agilent 6530 QTOF LC-MS in positive ionization mode.

Elemental analyses were obtained on an Elementar vario MICRO cube CHNS analyser equipped with a thermal conductivity detector. X-Ray powder diffraction patterns were collected using a Bruker D8 Advance powder diffractometer equipped with a copper k_α source (λ=1.5418 Å) operating at 40 kV and 40 mA. The instrument was fitted with an energy-dispersive LYNXEYE detector. Measurements were conducted using a rotatable capillary goniometer stage or a fixed goniometer stage with a rotating flat plate sample holders. IR spectroscopy was performed on a Perkin Elmer ATR-FTIR Spectrum 2. Thermogravimetric analysis was collected using a Perkin Elmer Pyris 1 TGA from 30-600 ºC at 10 ºC min^{-1}, under a 20 mL min^{-1} N_2 flow.

UV-vis absorption spectra were collected on a Cary 5000 UV-vis-NIR instrument, using a 1 cm internal length quartz cuvette and Cary WinUV (version 3.00) software in absorbance mode, with a resolution of 1 nm^{-1}. Calculation of absorption coefficients were undertaken with Excel software.

Nanoscopic characterisation was performed using a Bruker Multimode 5 AFM, operating in soft tapping mode under ambient conditions. Bruker OTESPA-R3 cantilever were used, with a drive amplitude and nominal resonance frequency of 20.4 mV and 290 kHz, respectively. Images were processed using standard techniques with free Gwyddion (version 2.47) software.

DLS data were collected using a Malvern Zetasizer Nano Series particle size analyser, using a He-Ne laser at 633 nm, operating in backscatter mode (173 °). Samples were equilibrated at 298 K for 60 s prior to analysis.

3. MOF Syntheses and Characterisation

Synthesis of ligand H_2**1**

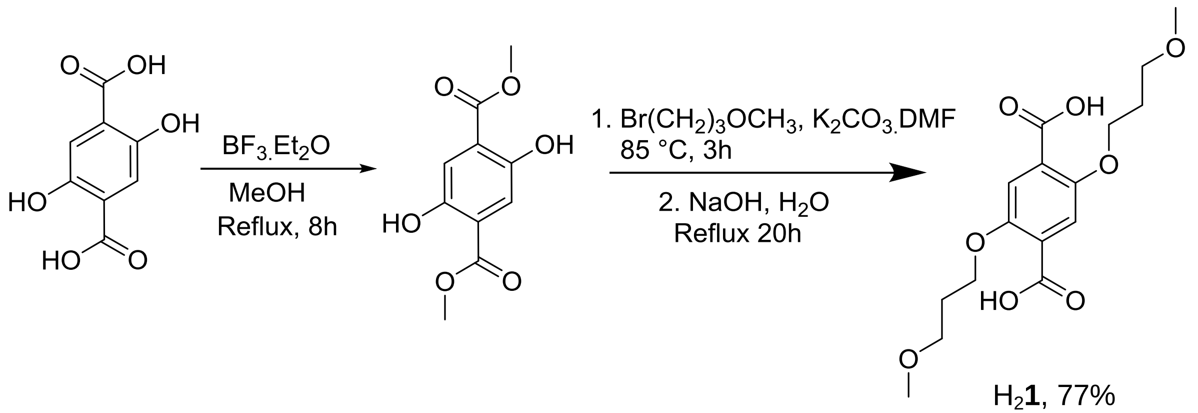

H_2**1**, 77%

Scheme S1. Synthetic pathway for the synthesis of 2,5-Bis(3-methoxypropoxy)-1,4-benzenedicarboxylate

Dimethyl 2,5-dihydroxyterephthalate,[1] ligand H_2**1** synthesised were synthesised according to a previously reported method summarised in scheme S1.[2, 3] This method had to be adapted for the synthesis of H_2**2** in order to improve the solubility of the more hydrophobic ligand during the final deprotection step.

Synthesis of ligand H_2**2**

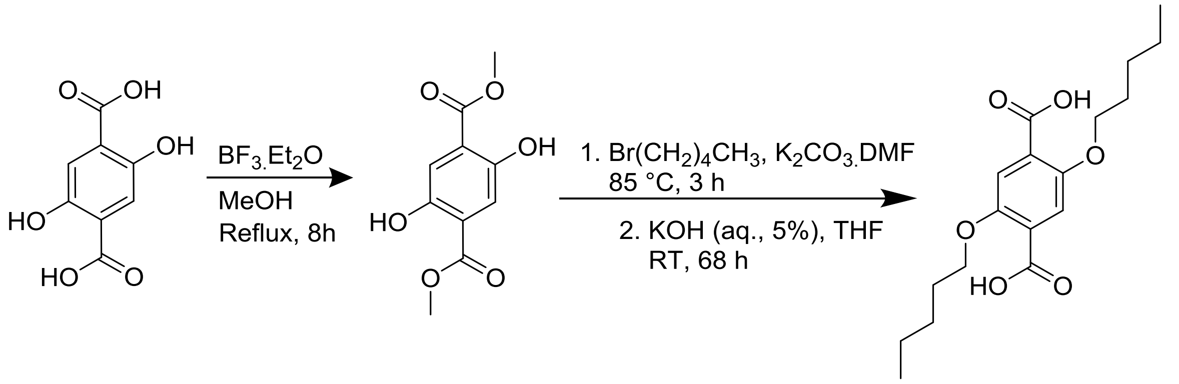

H_2**2**, 87%

Scheme S2. Synthetic pathway for the synthesis of 2,5-Bis(pentoxy)-1,4-benzenedicarboxylate

Dimethyl 2,5-dihydroxyterephtalate (2.089 g) and K_2CO_3 (5.228 g) were suspended in DMF (70 mL). 1-bromopentane (3.4 ml) was then added and the mixture heated at 85°C for 3 h. The mixture was then allowed to cool to RT overnight. The mixture was filtered and the filtrate collected. The solvent was removed under reduced pressure to yield a brown oil. This was refluxed in THF (70 mL) with aq. KOH (5%, 70 mL) for 68 h. The THF was removed under reduced pressure and the remaining mixture was acidified with aq. HCl (10%) then filtered to yield H_2**2** (2.714 g, 87%) as a white solid. ^{1}H NMR (400 MHz, $CDCl_3$) δ/ppm 11.34 (2H, s, COO*H*), 7.88 (2H, s, Ar-*H*), 4.40 (4H, t, *J*=5.68, Ar-OC*H*$_2$), 3.63 (4H, t, *J*=5.32, CH_3OC*H*$_2$), 3.40 (6H, s, OC*H*$_3$), 2.19 (4H, q, *J*=5.44, 5.64, OCH_2C*H*$_2$). ESI-MS (CH_3Cl): m/z 321.2 ($[M-H_2O]^+$), 339.2 ($[MH]^+$), 361.2 ($[M+Na]^+$). Elemental Analysis, calculated for $C_{18}H_{26}O6$: Expected: C, 63.89; H, 7.74. Found: C, 63.70; H, 7.46.

Synthesis of Cu(**1**)(DMF)

$Cu(NO_3)_2.3H_2O$ (0.1208 g, 0.5 mmol) and H_2**1** (0.1712 g, 0.5 mmol) were dissolved in DMF (10 mL) and sealed in a 10 mL reaction vial. The vial was heated at 110°C for 18 h then cooled to room temperature at 0.1 °C min^{-1}. The precipitate was filtered then washed with DMF (3x5 mL) and DCM (1x5 mL) to yield Cu(**1**)(DMF) (0.1625 g, 68%) as a light green powder. Elemental Analysis, calculated for $C_{19}H_{27}CuNO_9$: Expected: C, 47.83; H, 5.43; N, 2.94 %. Found: C, 45.95; H, 5.43; N, 2.78 %.

Synthesis of Cu(**2**)(DMF)

$Cu(NO_3)_2.3H_2O$ (0.1358 g, 0.56 mmol) and H_2**2** (0.1685 g, 0.5 mmol) were dissolved in DMF (10 mL) and sealed in a 10 mL reaction vial. The vial was heated at 110°C for 18 h then cooled to room temperature. The precipitate was filtered and washed with DMF (3x5 mL) and DCM (1x5 mL) to yield Cu(**2**)(DMF) (0.1825 g, 77%) as a light green powder. Elemental Analysis, calculated for $C_{21}H_{31}CuNO_7$: Expected: C, 53.30; H, 6.61; N, 2.96 %. Found: C, 51.11; H, 6.38; N, 2.96 %.

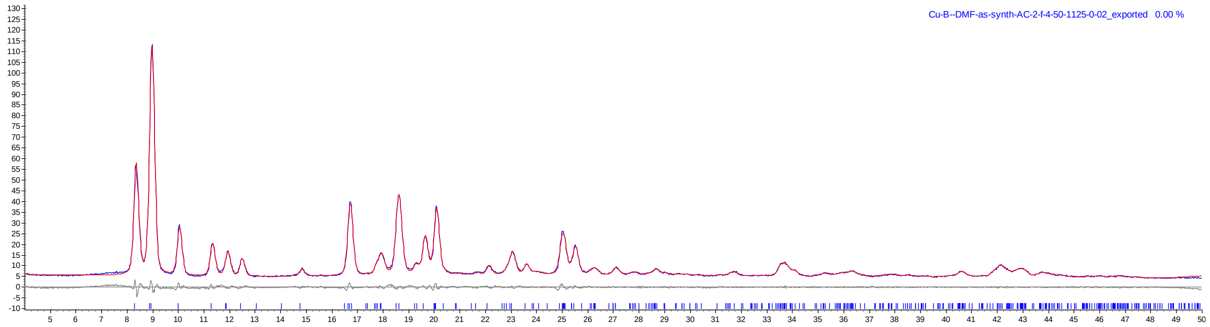

Figure S1. Pawley fit to the X-ray diffraction pattern of Cu(**2**)(DMF). The blue lines, red lines, and black lines represent the experimental, calculated, and difference profiles respectively. The blue tick marks indicate the positions of allowed Bragg reflections in the space group P-1. Experimental data was collected with the diffractometer operating in flat plate mode.

The corresponding Pawley fit for Cu(**1**)(DMF) can be found in reference S3.

Table S1. Crystallographic data of the Cu(X)(DMF) materials determined by Pawley refinement of XRPD data, where X is **1** or **2**.

Sample	Zn(**1**)(DMF)[a]	Cu(**1**)(DMF)[b]	Cu(**2**)(DMF)
Space Group	*P*-1	*P*-1	*P*-1
a / Å	10.4273(6)	10.5245(6)	10.825(3)
b / Å	10.8211(5)	10.7624(6)	10.795(2)
c / Å	10.8805(3)	10.8072(6)	10.835(3)
a / °	85.208(3)	85.293(8)	83.81(1)
b / °	74.992(3)	77.110(5)	79.95(2)

g / °	67.508(5)	68.264(4)	67.55(2)
V / Å^3	1095.45(9)	1108.43(12)	1151.0(6)
R_{wp}		2.898	4.353
R_{exp}		1.081	2.105

[a] Reference single crystal data collected at 100 K (CCDC 1460747).[3] [b] Data previously reported in [[3]].

4. UV-Vis Studies

Liquid Exfoliation method

The Cu(**1**)(DMF) or Cu(**2**)(DMF) powders were lightly ground to ensure homogeneity and 5 mg was added to a 10 mL glass vial along with 6 mL of the desired solvent. The sample was mixed in a vortex mixer for 30 seconds to disperse the sediment. The samples were sonicated using a Fisherbrand Elmasonic P 30H ultrasonic bath (2.75 L, 380/350 W, UNSPSC 42281712) filled with water. Samples were sonicated for 30 min at a frequency of 80 kHz with 100% power and the temperature was thermostatically maintained at 16-20°C using a steel cooling coil. Sonication was applied using a sweep mode and samples were rotated through the water using an overhead stirrer to minimise variation due to ultrasound "hot-spots". The set-up for exfoliation is shown in **Figure S2**. Following sonication, the vials were transferred to centrifuge tubes and centrifuged at 1500 RPM for 10 minutes and care was taken to avoid redispersing the samples on removal. All analysis was conducted on the top 3 mL of suspension.

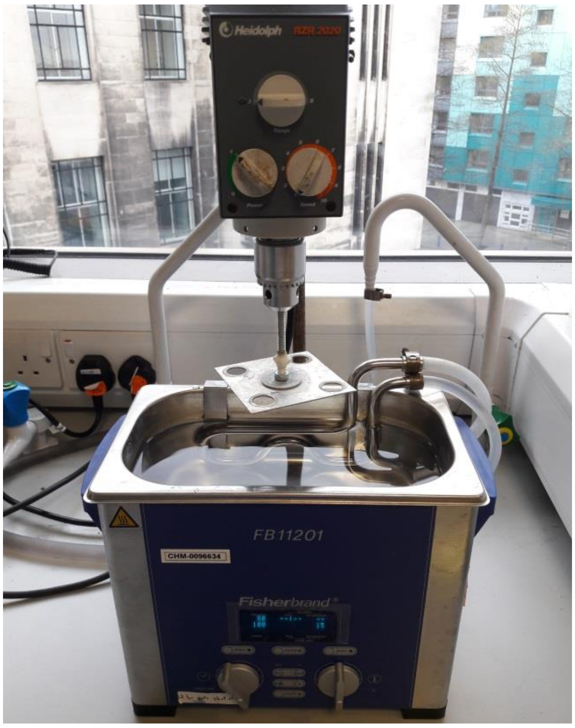

Figure S2. Experimental set-up for exfoliation using ultrasonication.

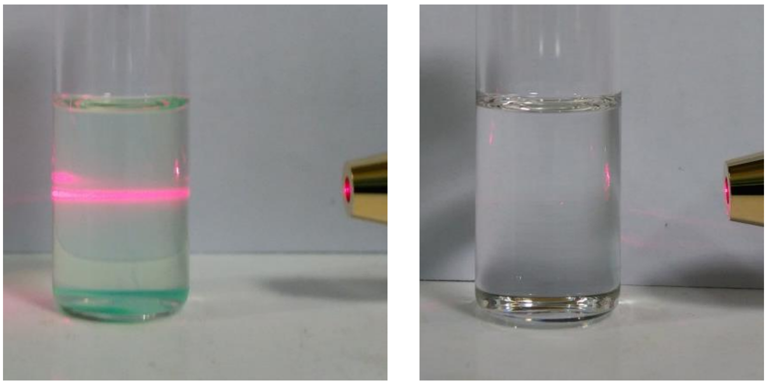

Figure S3. Images showing the presence of Tyndall scattering following the exfoliation of Cu(**1**)(DMF) in DMF (left) which is absent in the reference solution of DMF (right).

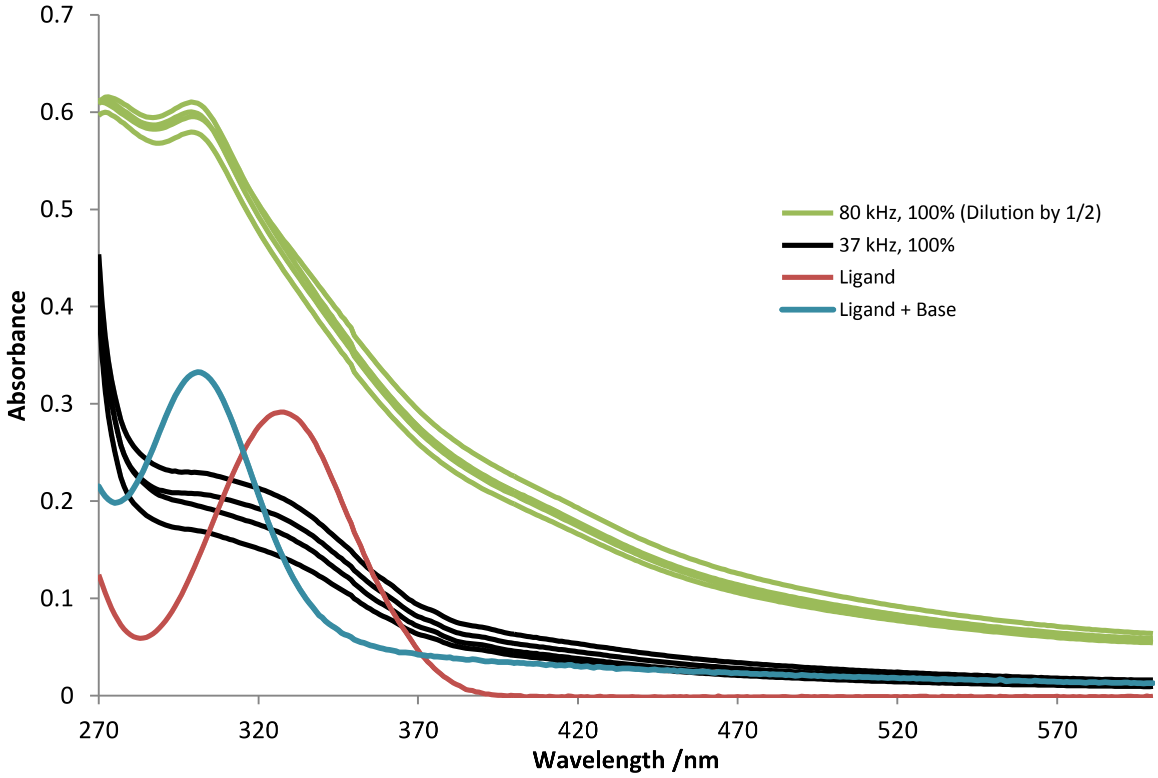

Figure S4 Preliminary data showing UV-vis spectra of suspension following liquid exfoliation of Cu(**1**)(DMF) for 30 mins in DMF at 80 KHz (green) and 37 KHz (black) as well as for **1** in DMF (red) and **1** + 2 eq. NaOH. It should be noted that the temperature was not regulated during this experiment.

UV-vis Method

A background was taken of a quartz cuvette with 1 cm internal path length filled with the relevant solvent. The cuvette was then loaded with 3 mL of sample taken from the top of a freshly prepared suspension and loaded into the spectrophotometer. Spectra were recorded in absorbance mode with a scan range of 200-800 nm and a resolution of 1 nm^{-1}. Highly absorbing samples were diluted with additional solvent.

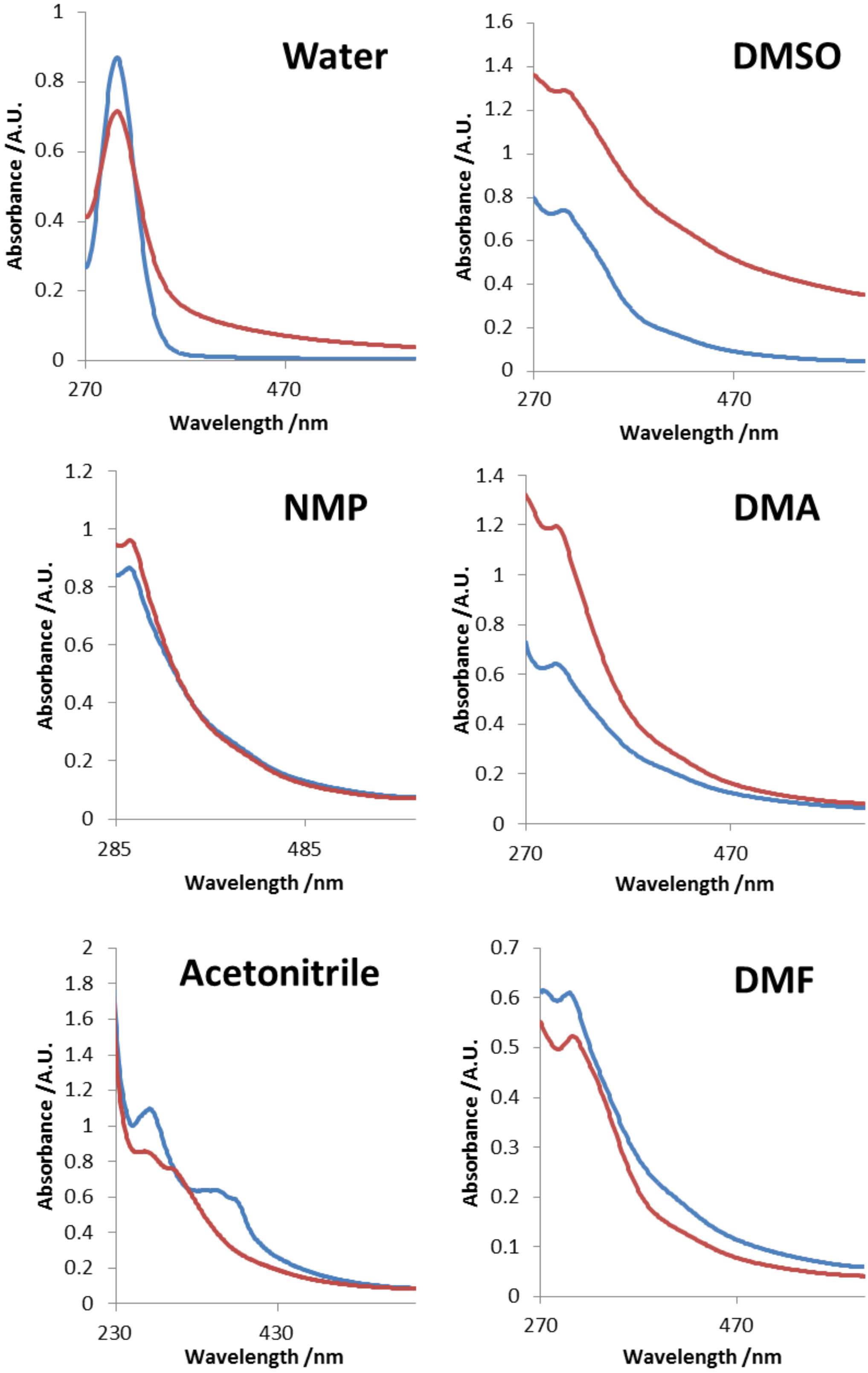
Water
Absorbance /A.U.
1
0.8
0.6
0.4
0.2
0
270
470
Wavelength /nm
DMSO
Absorbance /A.U.
1.6
1.4
1.2
1
0.8
0.6
0.4
0.2
0
270
470
Wavelength /nm
NMP
Absorbance /A.U.
1.2
1
0.8
0.6
0.4
0.2
0
285
485
Wavelength /nm
DMA
Absorbance /A.U.
1.4
1.2
1
0.8
0.6
0.4
0.2
0
270
470
Wavelength /nm
Acetonitrile
Absorbance /A.U.
2
1.8
1.6
1.4
1.2
1
0.8
0.6
0.4
0.2
0
230
430
Wavelength /nm
DMF
Absorbance /A.U.
0.7
0.6
0.5
0.4
0.3
0.2
0.1
0
270
470
Wavelength /nm

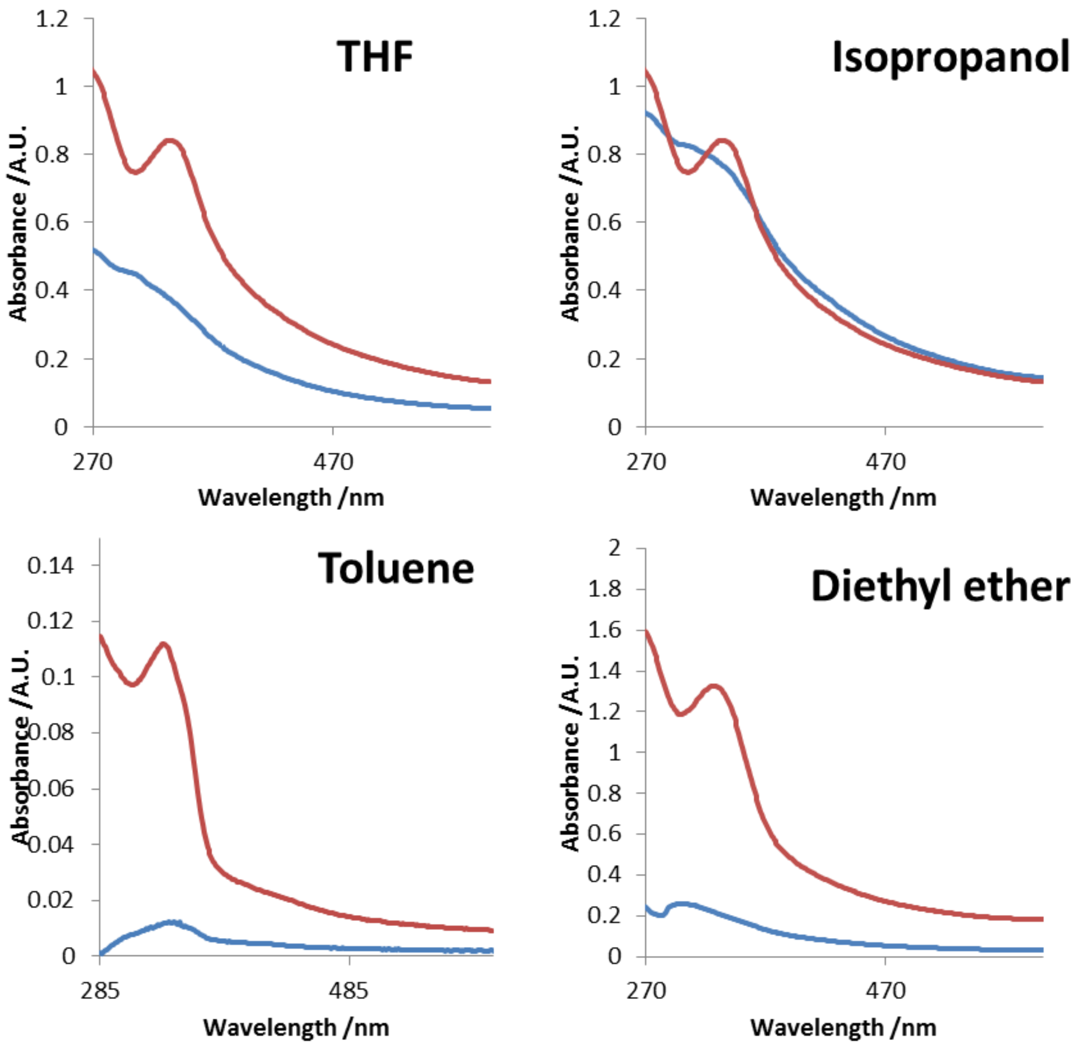

Figure S5. Representative spectra for the exfoliation of Cu(**1**)(DMF) [blue] and Cu(**2**)(DMF) [red] in a range of solvents.

Table S2. List of solvents initially investigated but excluded from further analysis

Excluded solvents	Reason
Acetone	UV/Vis cut off too high (330 nm).
Benzonitrile	UV/Vis cut off too high (330 nm). No absorption maximum in 300-305 nm region.
Benzyl alcohol	Hygroscopic. Handling recommended under inert atmosphere.
tert-Butanol	Solid at ambient temperature.
Carbon disulfide	UV/Vis cut-off too high (380 nm).
Ethanol	No absorption maximum in 300-305 nm region.
Ethyl acetate	No absorption maximum in 300-305 nm region.
Ethylene glycol	Viscosity too high.

Glycerol	Viscosity too high.
Methanol	No absorption maximum in 300-305 nm region.
Methyl isobutyl ketone	UV/Vis cut-off too high (335 nm).

4.2 Molar Extinction Coefficients

A known mass of MOF (approximately 8 mg) was added to a 10 mL volumetric flask which was then made up with the relevant solvent. The flask was sonicated at 80 kHz until all solid material was dispersed in solution i.e. no sediment remained on the bottom of the flask and large particles could not be seen in solution. This solution was then diluted accordingly and analysed by UV/Vis spectrometry. The absorbance values at λ_{max} of the spectra obtained was plotted against MOF concentration. This yielded a linear plot to which the gradient corresponds to the molar extinction coefficient for the MOF in that specific solvent.

Note, it was not possible to calculate extinction coefficients for samples dispersed in cyclohexane or hexane due to the low degree of dispersion.

Table S3. Molar extinction coefficients for Cu(**1**)(DMF) exfoliated in the selected solvents.

Solvent	λ_{max} /nm	Molar extinction coefficient / $mol^{-1}\ dm^{3}\ cm^{-1}$	%Standard Error
Acetonitrile	276	3466	8
DCM	274	3274	7
Diethyl ether	299	6693	13
DMA	300	2732	5
DMF	302	2644	4
DMSO	299	1892	6
IPA	271	5104	10
NMP	299	2705	6
THF	304	2404	6
Water	302	2827	5

Table S4. Molar extinction coefficients for Cu(**2**)(DMF) exfoliated in the selected solvents.

Solvent	λ_{max} /nm	Molar extinction coefficient / $mol^{-1}\ dm^{3}\ cm^{-1}$	%Standard Error
Acetonitrile	304	4208	1
DCM	271	2467	10
Diethyl ether	334	3519	6
DMA	303	3423	2
DMF	303	3976	2

IPA	338	4339	4
NMP	301	3731	3
THF	331	4489	3
Water	303	3088	3

Table S5. List of solvent properties used for analysis

	Polarity index	∂_t	∂_d	∂_p	∂_h	γ_L @25 C /(mN/m)
Acetonitrile	5.8	24.4	15.3	18.0	6.1	28.66
DMSO	7.2	26.7	18.4	16.4	10.2	42.92
Water	9	47.8	15.6	16	42.3	71.99
DMF	6.4	24.8	17.4	13.7	11.3	35.74
NMP	6.7	22.9	18	12.3	7.2	40.21
DMA	6.5	22.7	16.8	11.5	10.2	36.70
Isopropanol	3.9	23.5	15.8	6.1	16.4	20.93
Tetrahydrofuran	4	19.4	16.8	5.7	8.0	26.70
Diethyl ether	2.8	15.8	14.5	2.9	5.1	16.65
Cyclohexane	0.2	16.8	16.8	0.0	0.2	24.16
n-Hexane	0.1	14.9	14.9	0.0	0.0	17.89

4.3 Dispersed Concentration vs Solvent Parameter Plots

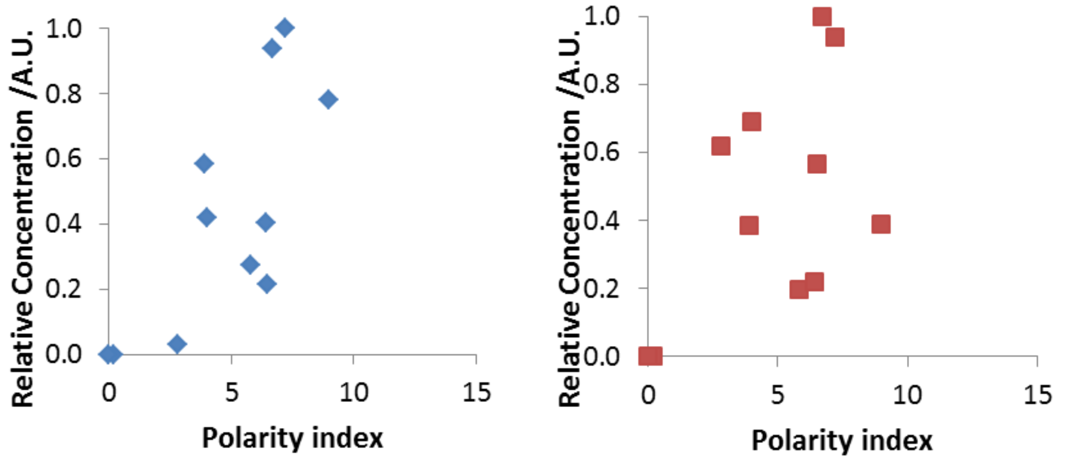

Figure S6. Plots showing the concentration Cu(**1**)(DMF) (left/blue) and Cu(**2**)(DMF) (right/red) normalised relative to the highest value against polarity index of the exfoliation solvent.

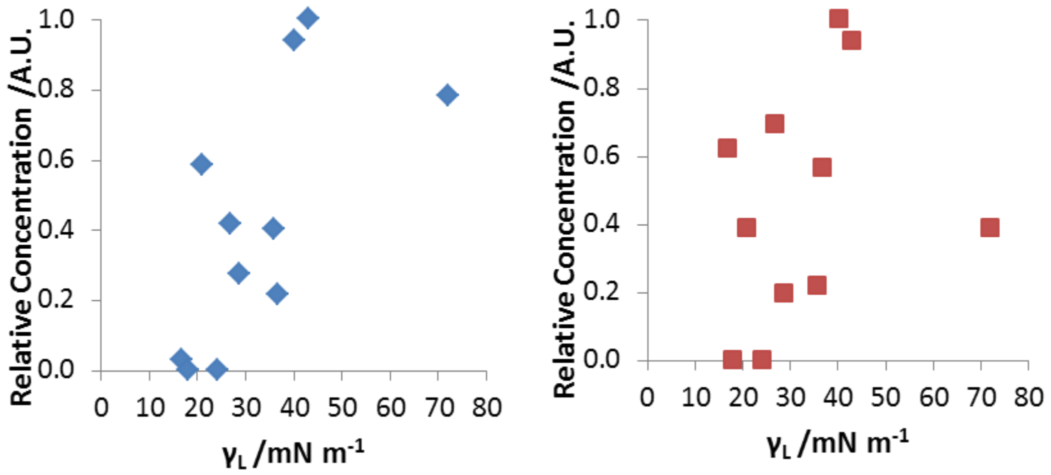

Figure S7. Plots showing the concentration Cu(**1**)(DMF) (left/blue) and Cu(**2**)(DMF) (right/red) normalised relative to the highest value against the surface tension γL of the exfoliation solvent.

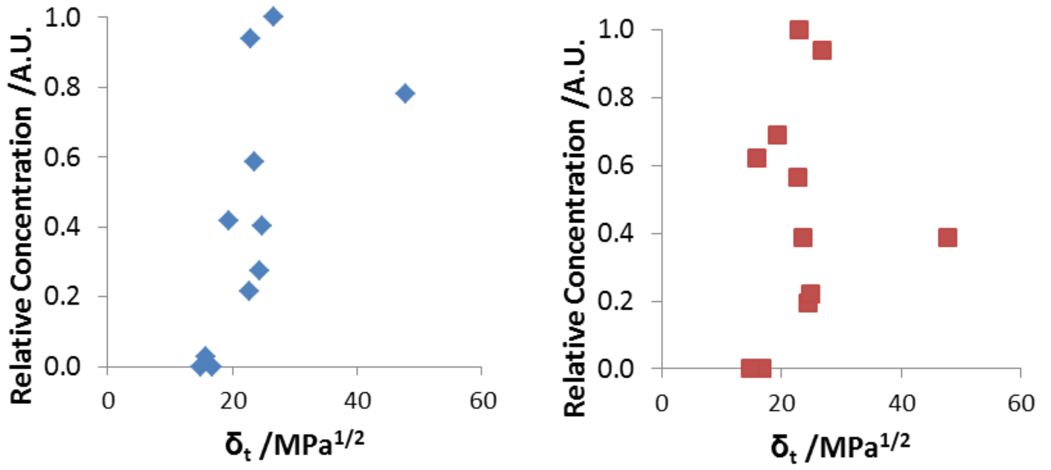

Figure S8. Plots showing the concentration Cu(**1**)(DMF) (left/blue) and Cu(**2**)(DMF) (right/red) normalised relative to the highest value against the total Hansen Solubility parameter (δt) of the exfoliation solvent.

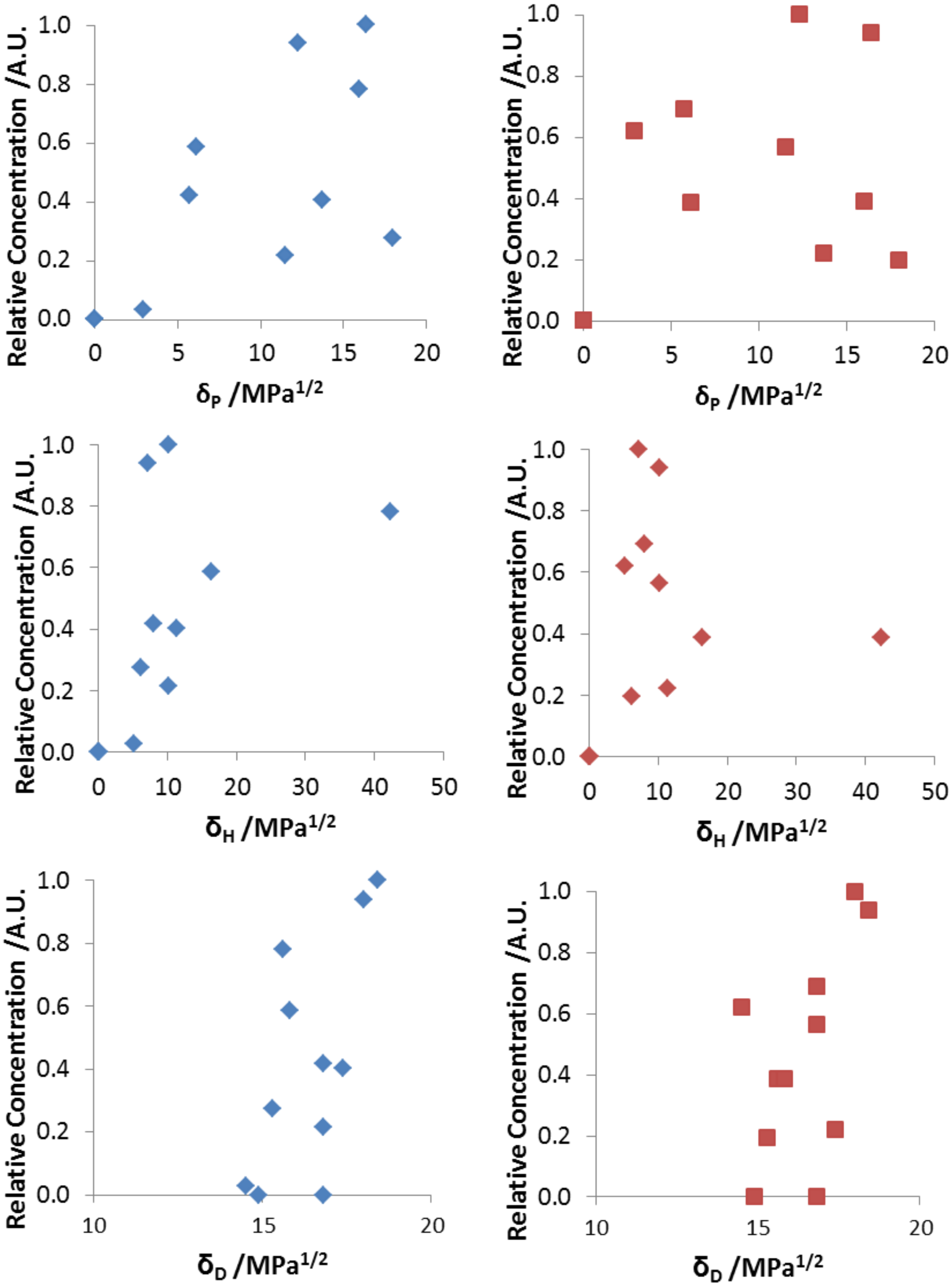

Figure S9. Plots showing the concentration Cu(**1**)(DMF) (left/blue) and Cu(**2**)(DMF) (right/red) normalised relative to the highest value against (top to bottom) the polar (δp), hydrogen bonding (δd) and dispersive (δd) Hansen Solubility parameters of the exfoliation solvent.

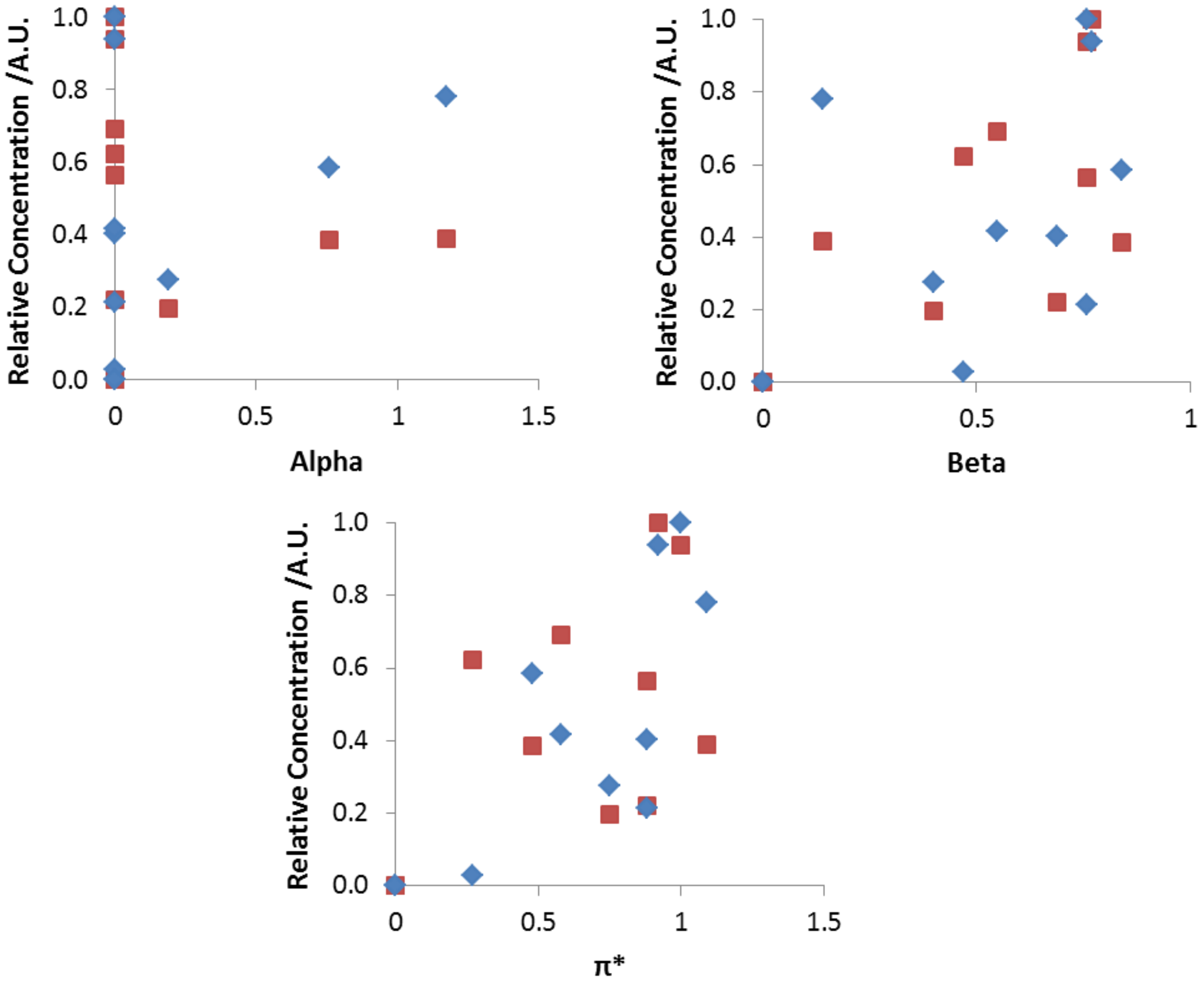

Figure S10. Plots showing the concentration Cu(**1**)(DMF) (blue) and Cu(**2**)(DMF) (red) normalised relative to the highest value against hydrogen bond donor (alpha), hydrogen bond acceptor (beta) and dipolarity/polarizability (π*) Kamlet-Taft parameters of the exfoliation solvent.

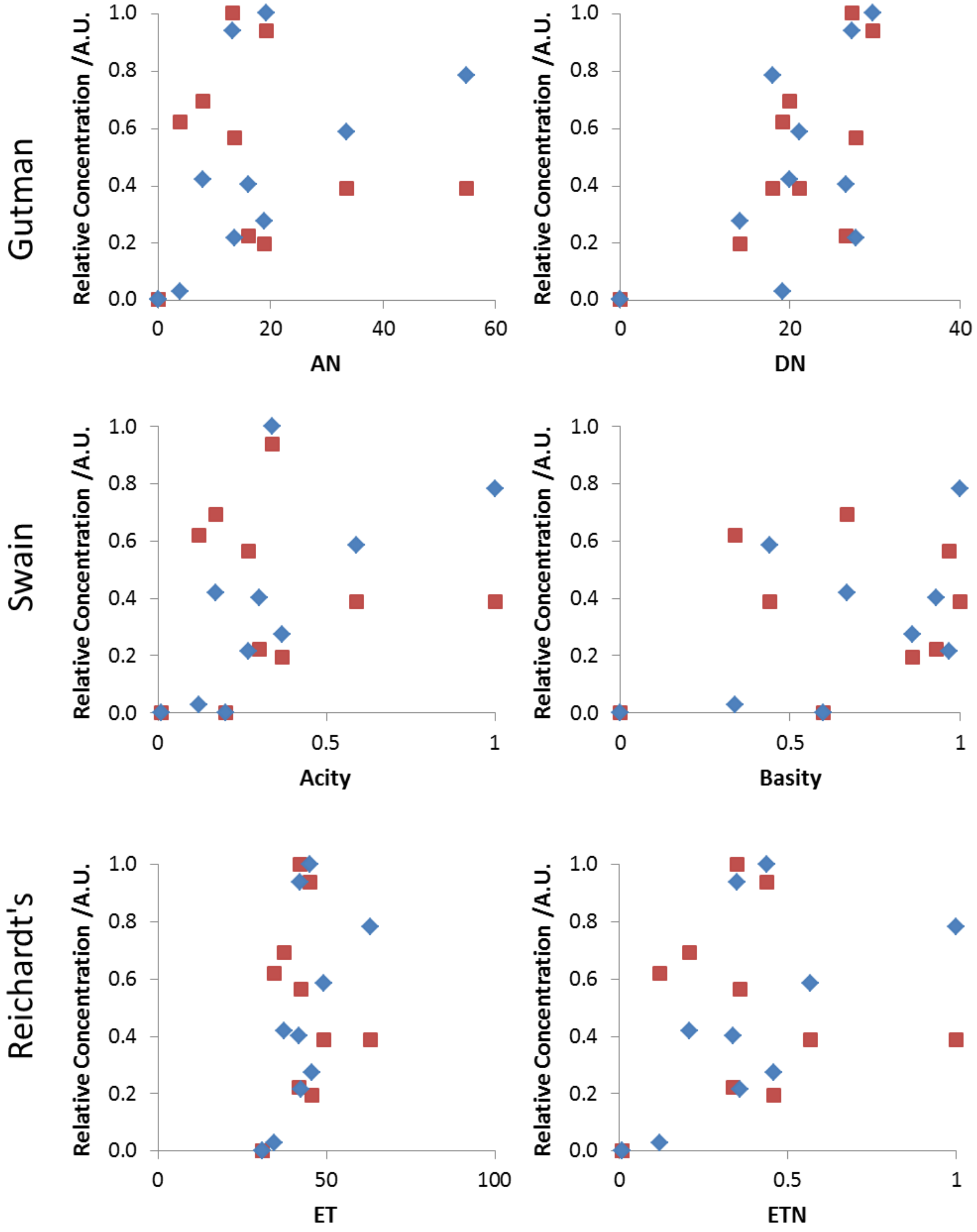

Figure S11. Plots showing the concentration Cu(**1**)(DMF) (blue) and Cu(**2**)(DMF) (red) normalised relative to the highest value against (top to bottom) the Gutman, Swain and Reichardt's solubility parameters of the exfoliation solvent.

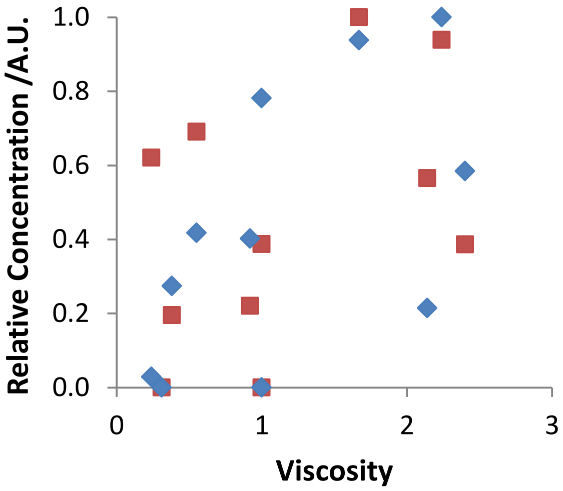

Figure S12. Plots showing the concentration Cu(1)(DMF) (blue) and Cu(2)(DMF) (red) normalised relative to the highest value against the viscosity of the exfoliation solvent.

4.4 Stability Tests

a)

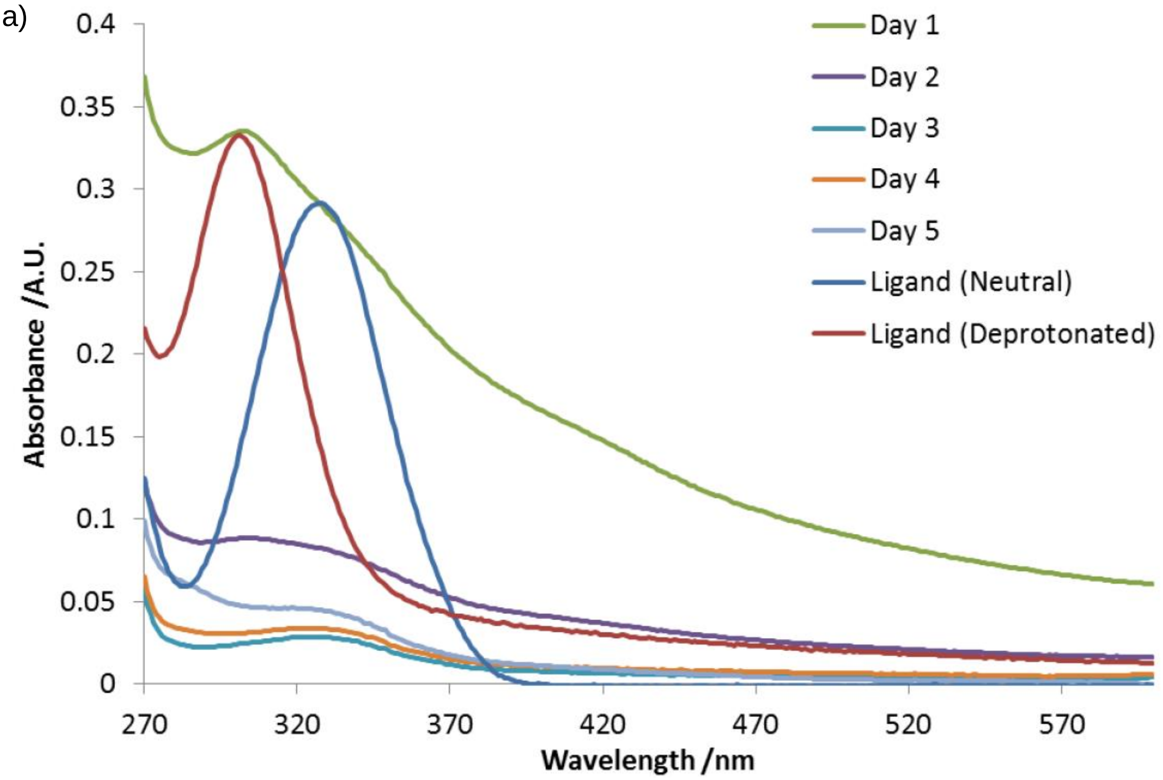

b)

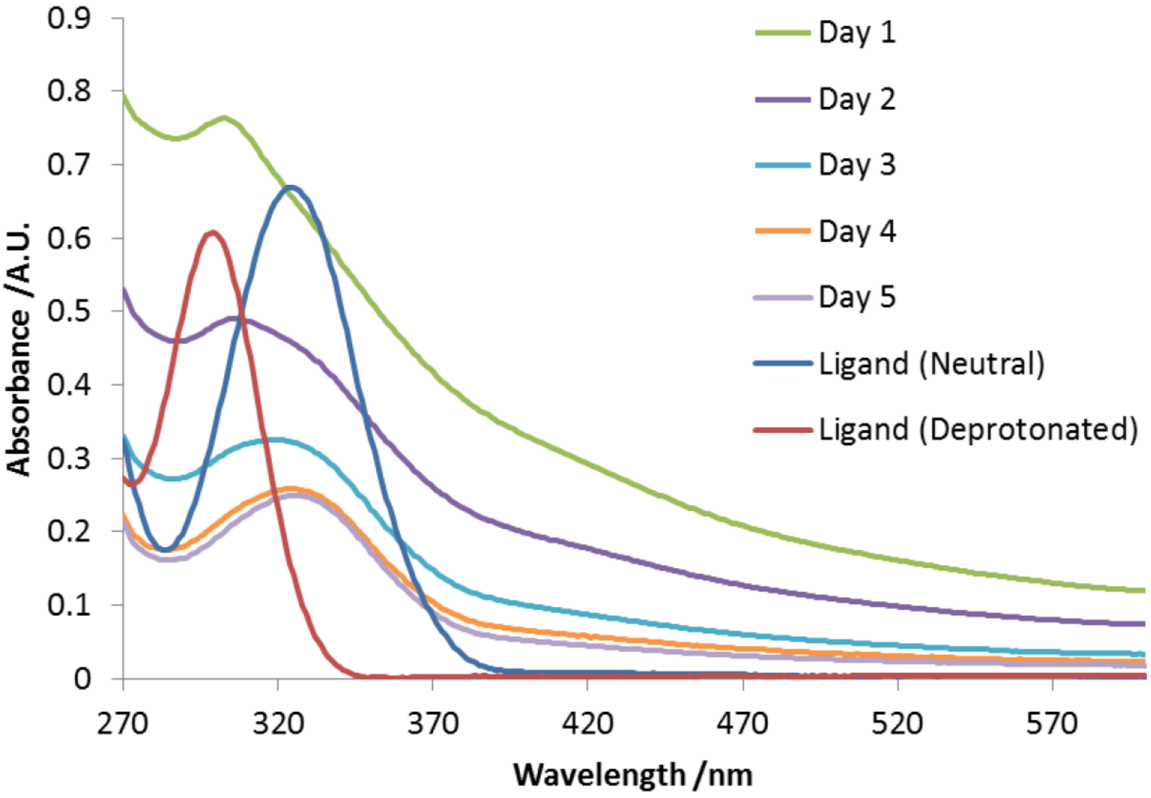

c)

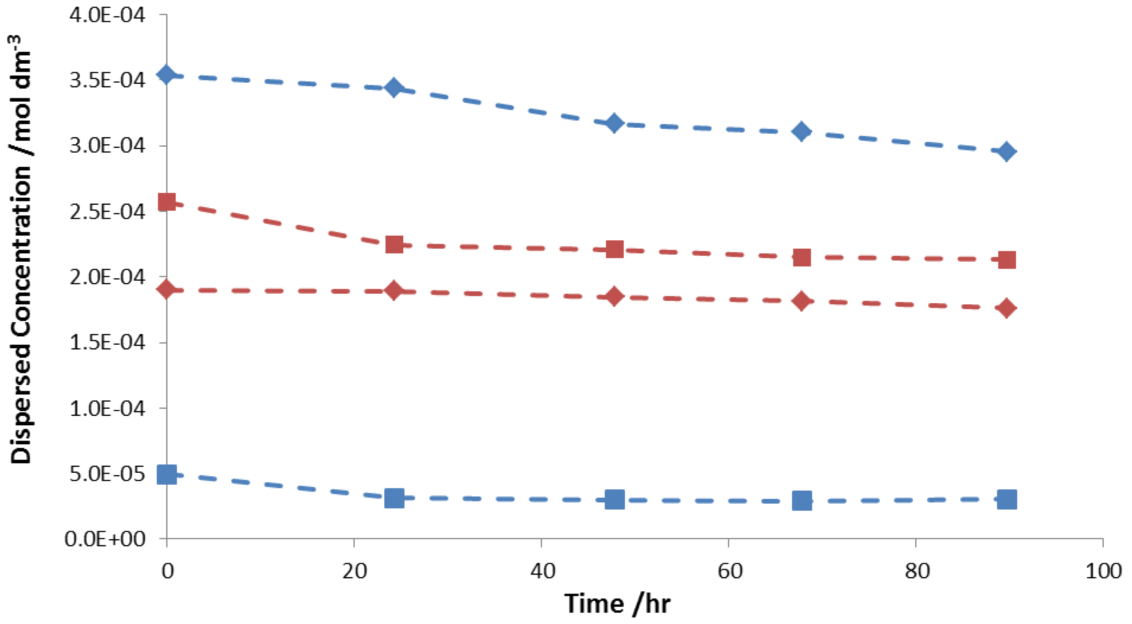

Figure S13. a) Change in absorption spectra over 5 days for Cu(**1**)(DMF) exfoliated in DMF**. b)** Change in absorption spectra over 5 days for Cu(2)(DMF) exfoliated in DMF. c) Dispersed concentration of Cu(**1**)(DMF) [blue] and Cu(**2**)(DMF) [red] exfoliated in water [diamonds] and diethyl ether [squares] over time.

5. Atomic Force Microscopy

5.1 Method Development

Samples for AFM analysis were prepared by pipetting 5x5 µl of suspension onto a freshly cleaved mica substrate heated to 80°C. AFM images were captured in soft tapping mode under ambient conditions using a Bruker Multimode 5 Atomic Force Microscope. Bruker OTESPA-R3 cantilevers were used with a drive amplitude and resonance frequency of approximately 20.4 mV and 290 kHz, respectively. Captured images were processed using Gwyddion (version 2.47) software.

Table S6. Table summarising AFM images obtained following sonication of Cu(1)(DMF)

Solvent	Sonication Time (h)	Thickness	Lateral Dimension	Notes
Water	0.5	Typically 100-200 nm. Thinnest ~30 nm	Discrete sheets usually 0.5-1 µm	-
	12	Typically 400 nm. Stepped edges of 15 nm observed	0.5-2 µm	-
Et_2O	0.5	100-300 nm	Broad distribution. Between 0.1-4 µm	Spherical and not “sheet-like”
	12	100-150 nm	1.5 µm	-

Table S7. Table summarising AFM images obtained following sonication of Cu(1)(DMF)

Solvent	Sonication Time	Thickness	Lateral Dimensions	Notes
Water	30 minutes	Typically 400 nm, Thinnest 15-60 nm.	0.5-2 µm	-
	12 hours	Range 9-600 nm, Typically ~100 nm	0.5-1.5 µm	Single large flake, 9 nm thick and almost 2 µm square
Et_2O	30 minutes	Thinnest 13 nm, Typically 20-100 nm	0.5-1 µm	Significant amount of aggregates
	12 hours	20-50 nm	0.5-1.5 µm	Large proportion of sheet-like material

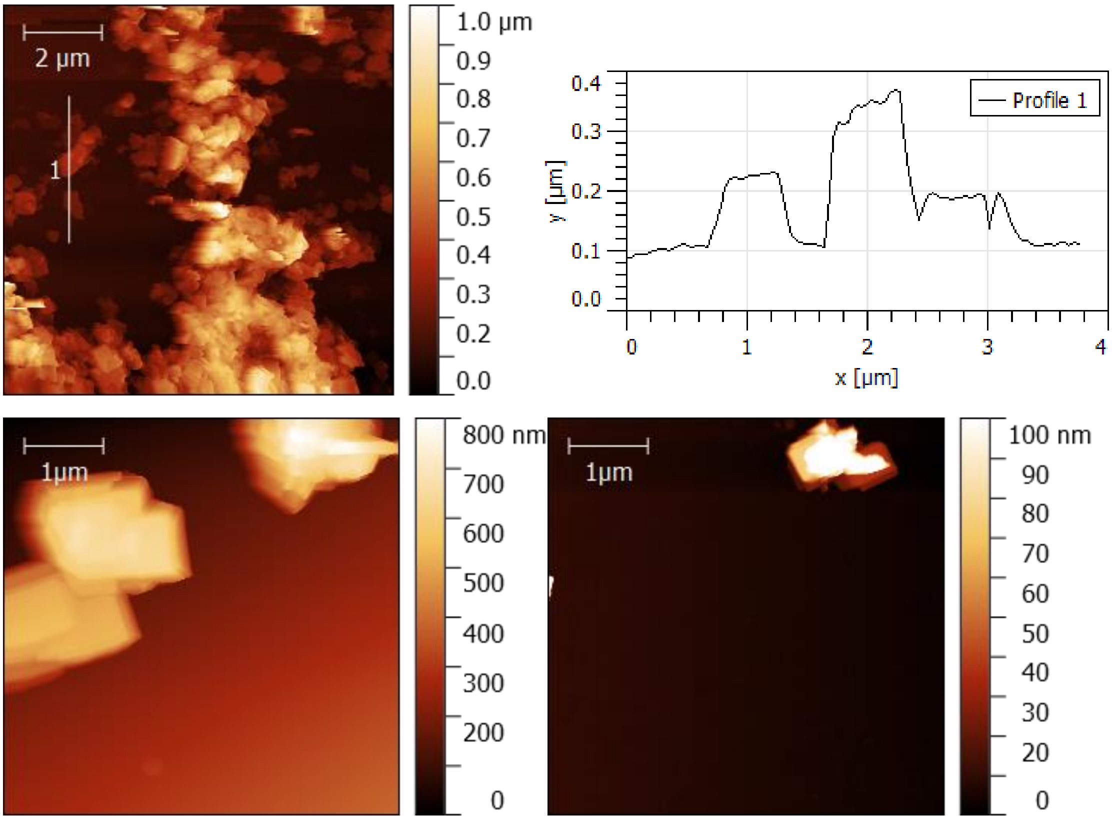

Figure S14. AFM image and height profile of Cu(**1**)(DMF) exfoliated for 30 minutes in water.

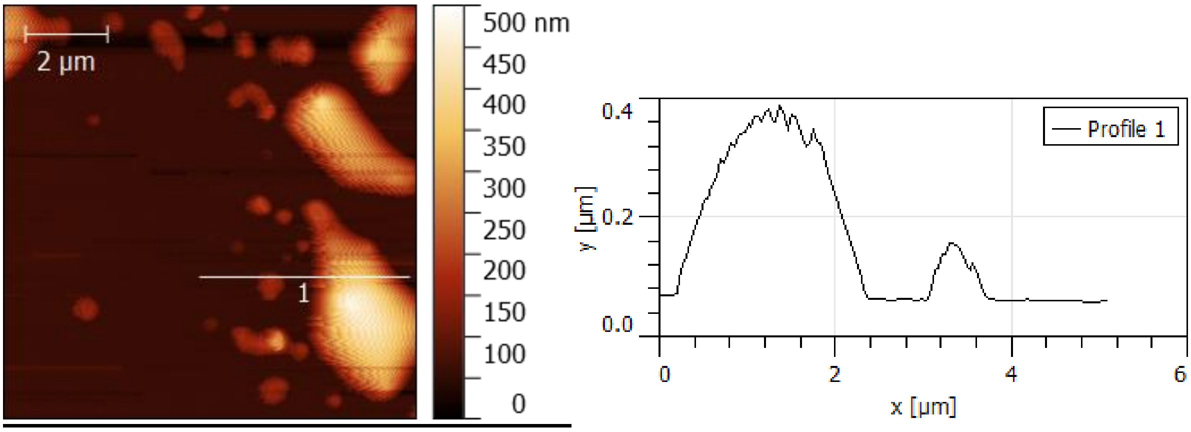

Figure S15. AFM image and height profile for Cu(**1**)(DMF) exfoliated for 30 minutes in diethyl ether.

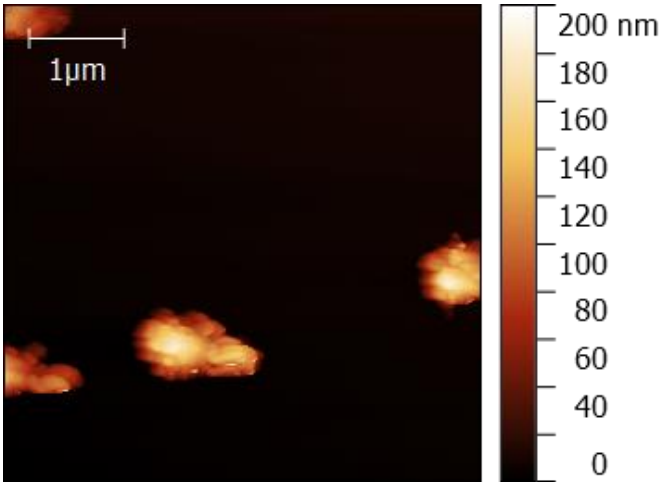

Figure S16. AFM image of Cu(**1**)(DMF) exfoliated in diethyl ether for 12 hours.

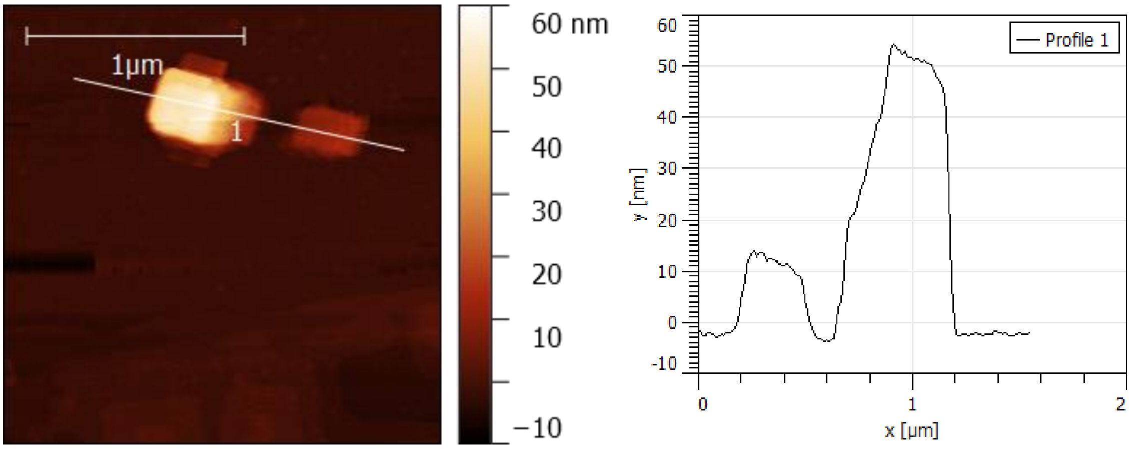

Figure S17. AFM image and height profile of Cu(**2**)(DMF) exfoliated for 30 minutes in water.

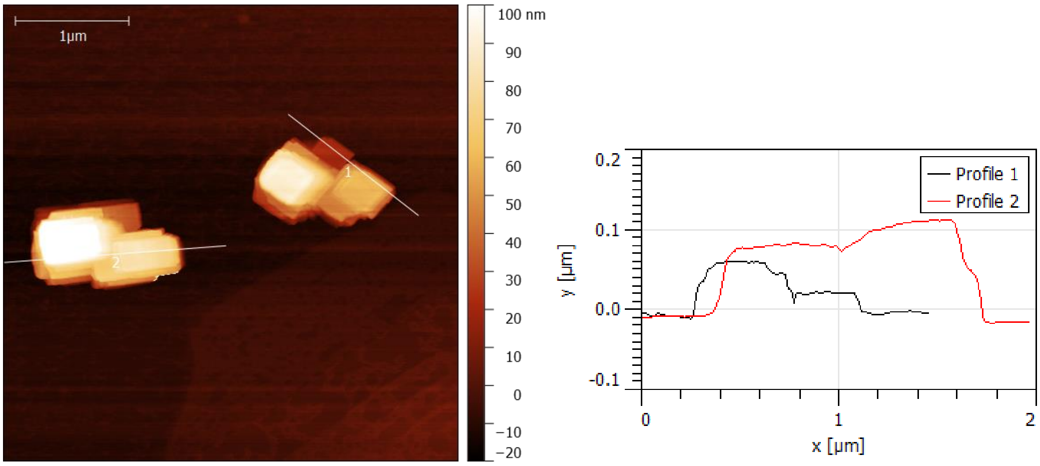

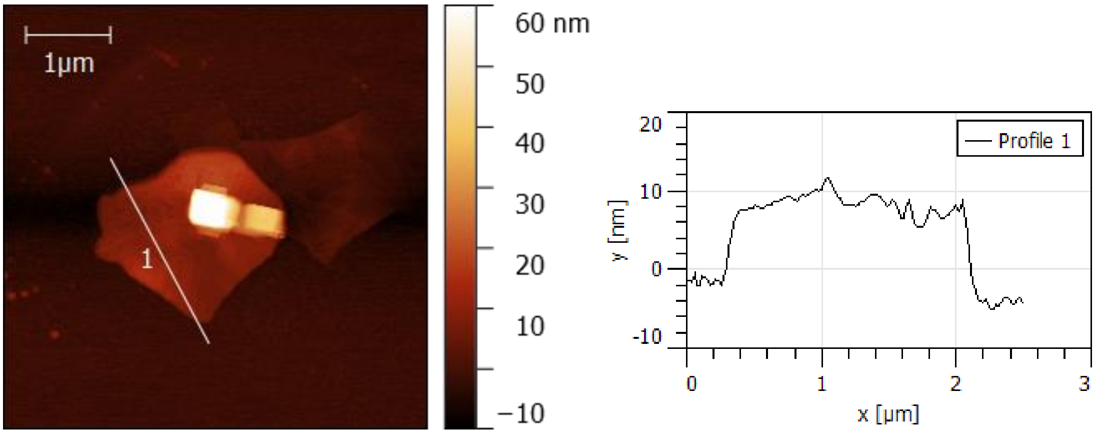

Figure S18. AFM images and height profiles for Cu(**2**)(DMF) exfoliated for 12 hours in diethyl ether.

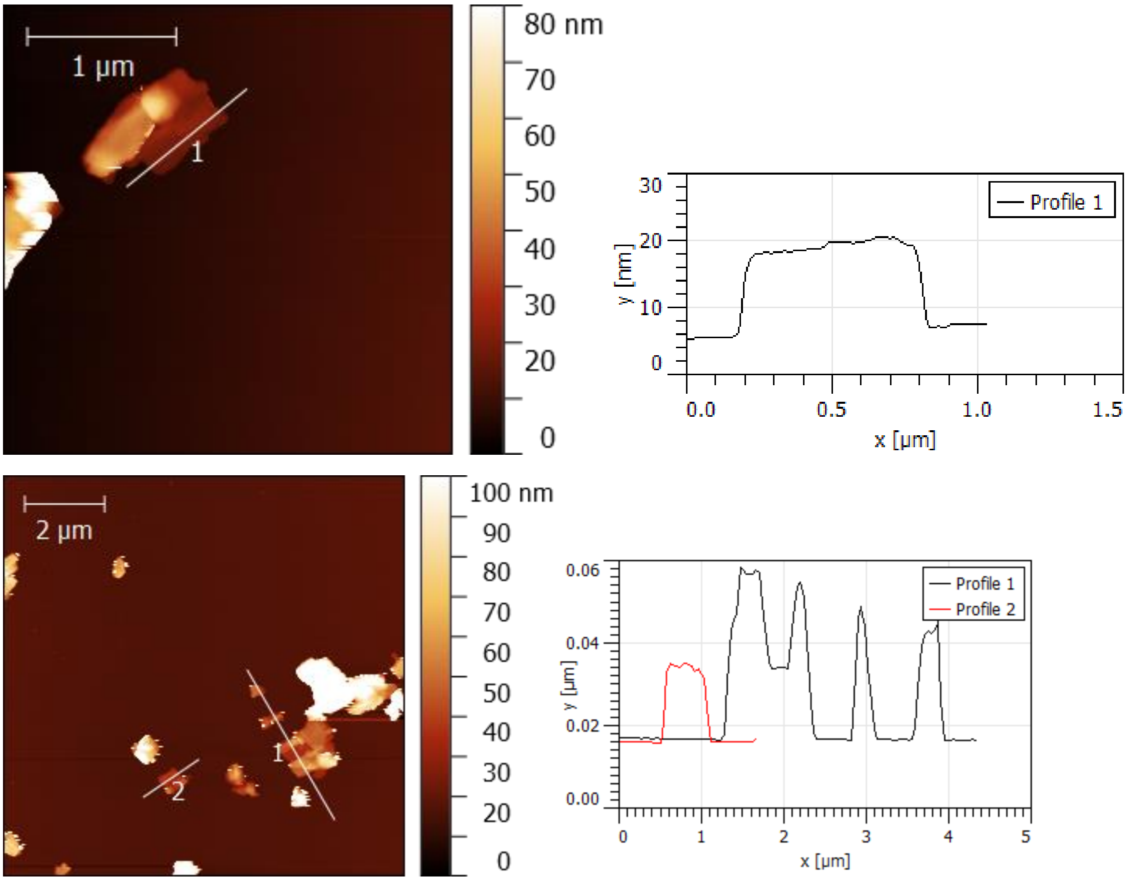

Figure S19. AFM images and height profiles for Cu(**2**)(DMF) exfoliated for 30 minutes in diethyl ether. For the bottom image, the height profile of line 1 and 2 are shown in black and red, respectively.

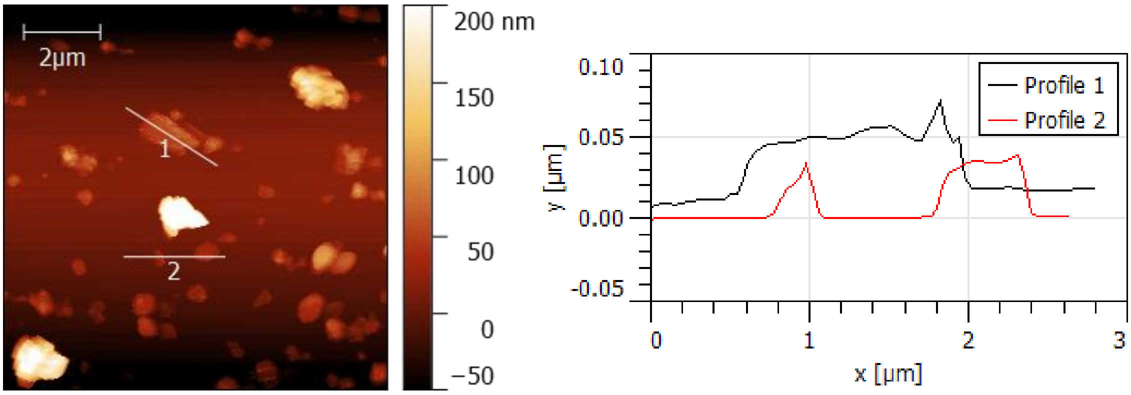

Figure S20. AFM image and height profile for Cu(**2**)(DMF) exfoliated in 12 hours in diethyl ether. The height profile of line 1 and 2 are shown in black and red, respectively.

5.2 Particle Sizing Studies

Cu(1)(DMF) exfoliated in MeCN for 12 hrs, sequential centrifugation steps.

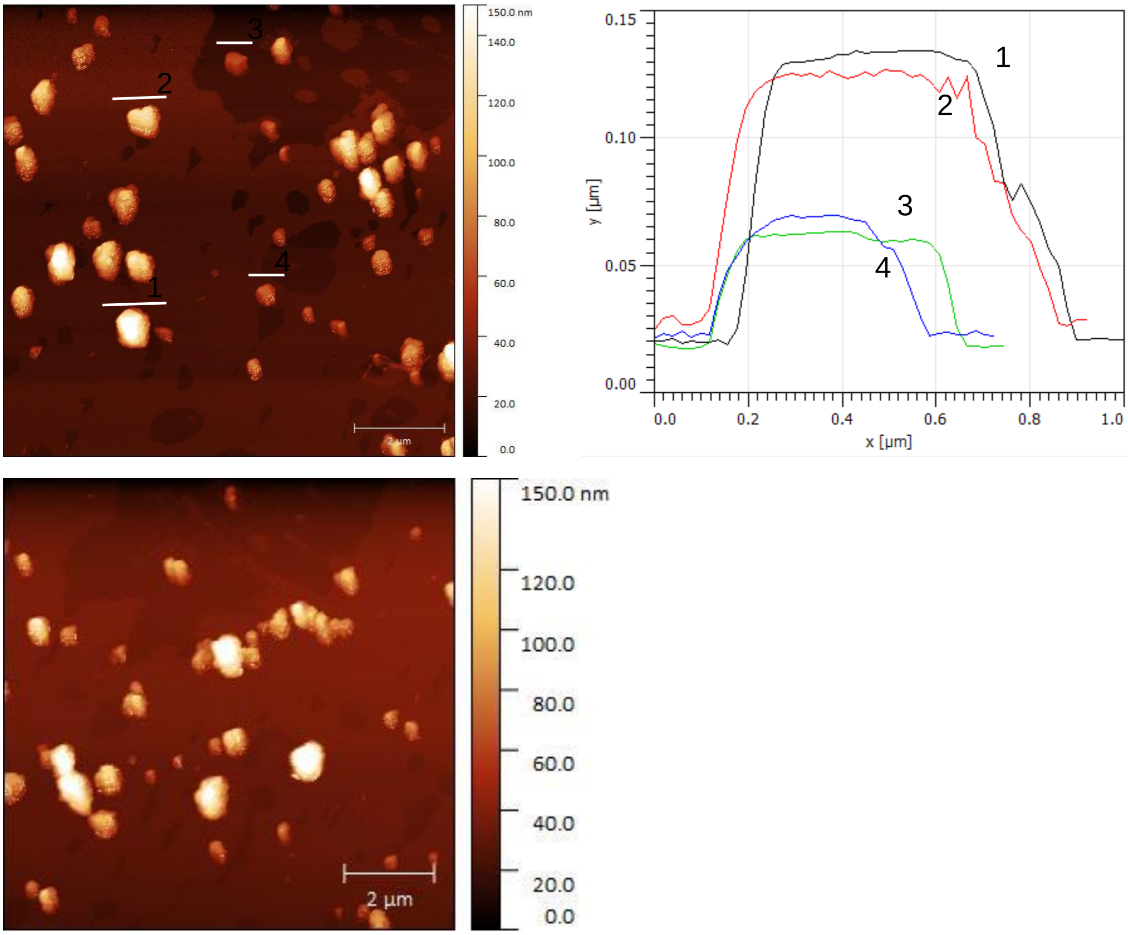

Figure S21. AFM images and example height plot used for the particle sizing of Cu(**1**)(DMF) exfoliated for 12 hrs and centrifuged at 1500 rpm for 1 hr.

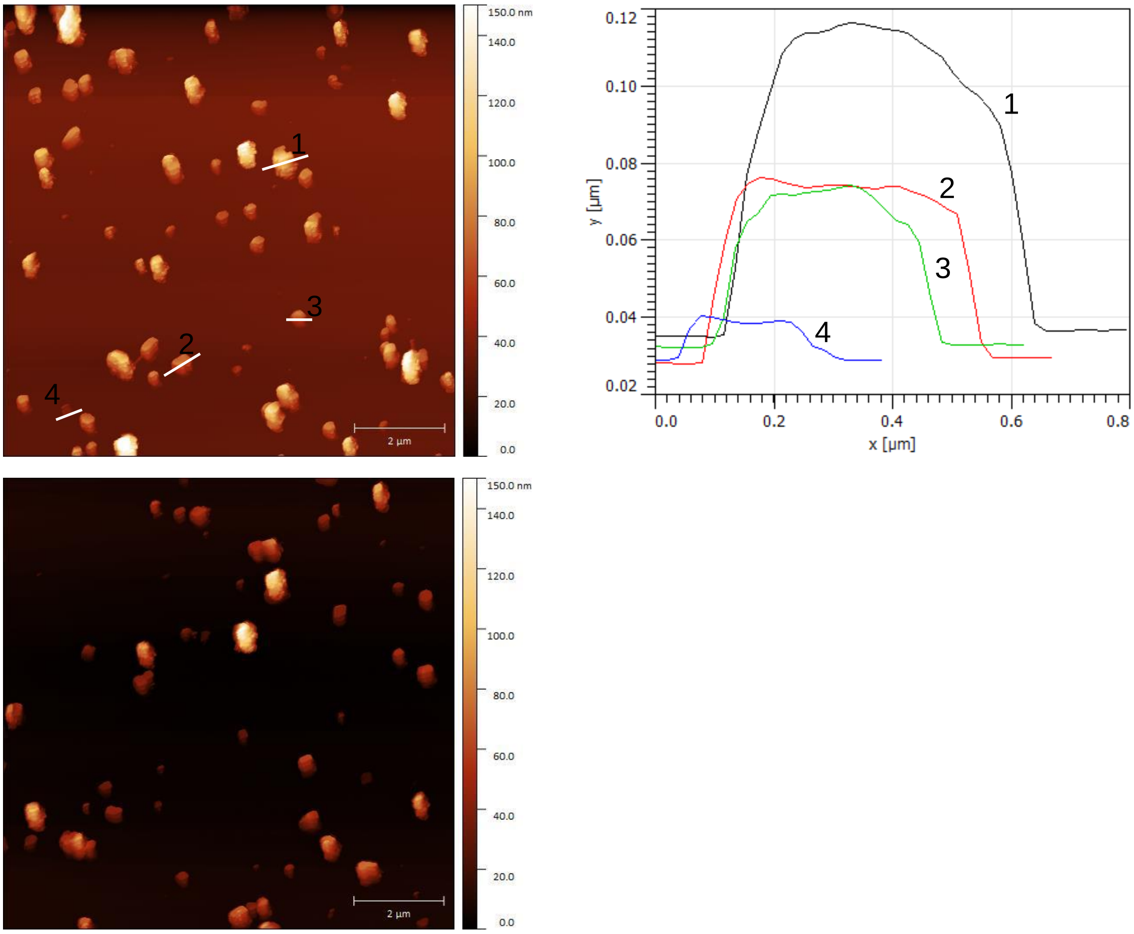

Figure S22. AFM images and example height plot used for the particle sizing of Cu(**1**)(DMF) exfoliated for 12 hrs and centrifuged at 4500 rpm for 30 mins.

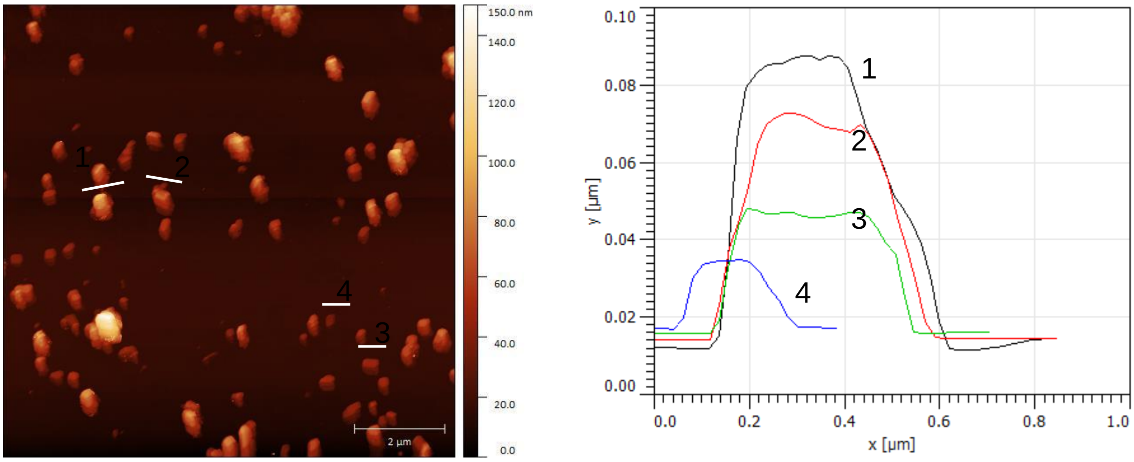

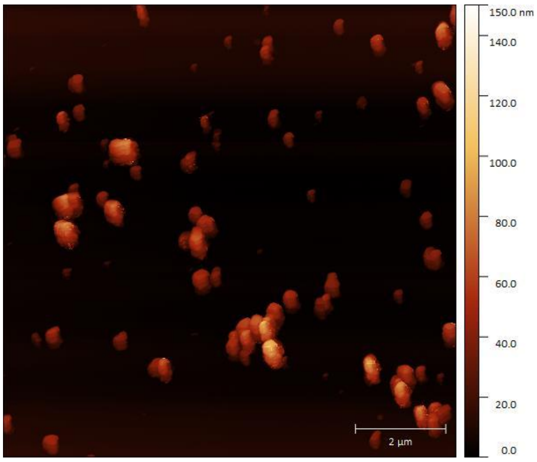

Figure S23. AFM images and example height plot used for the particle sizing of Cu(**1**)(DMF) exfoliated for 12 hrs and centrifuged at 4500 rpm for 4 hrs.

Cu(2)(DMF) exfoliated in MeCN for 12 hrs with sequential centrifugation

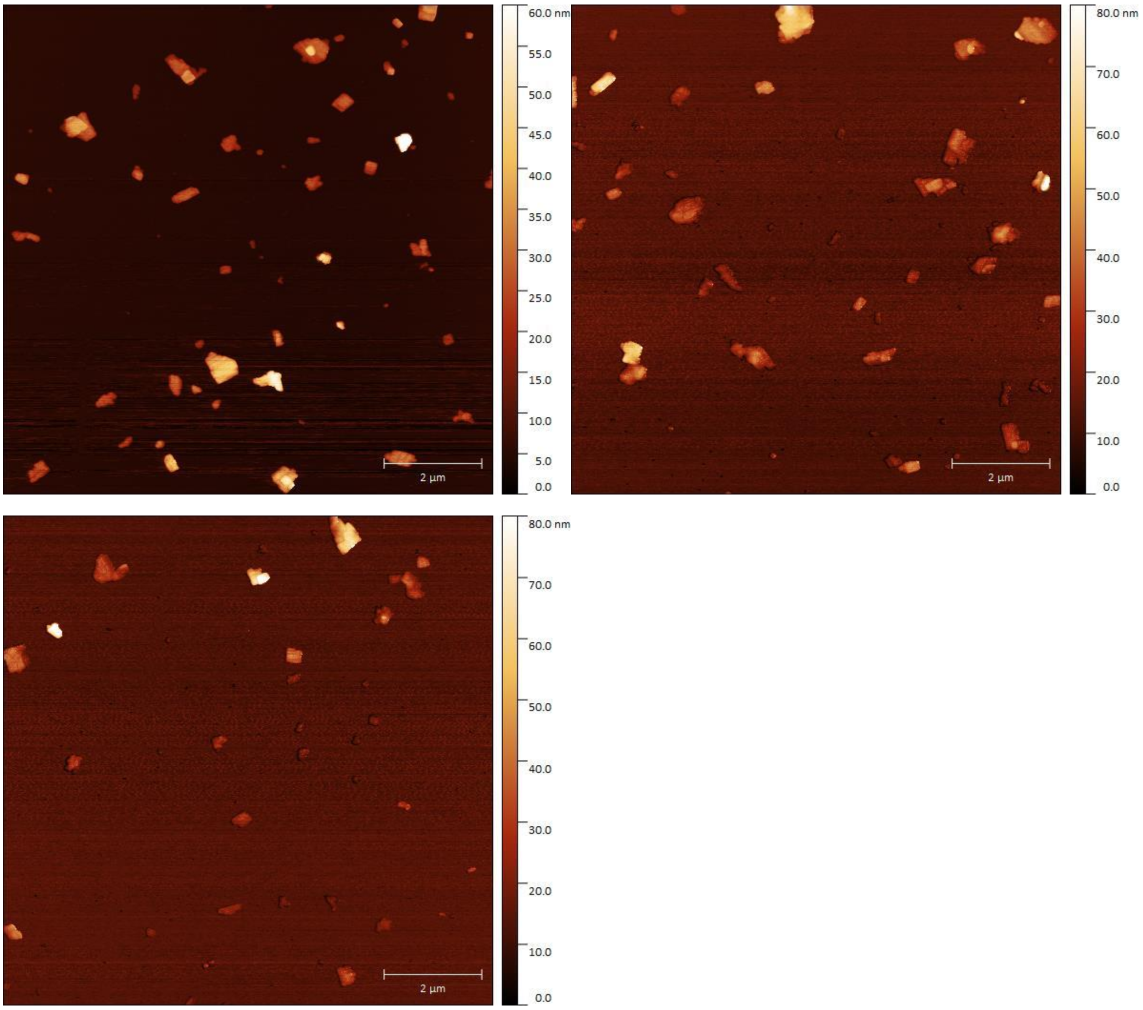

Figure S24. AFM images and example height plot used for the particle sizing of Cu(**2**)(DMF) exfoliated for 12 hrs and centrifuged at 1500 rpm for 30 mins.

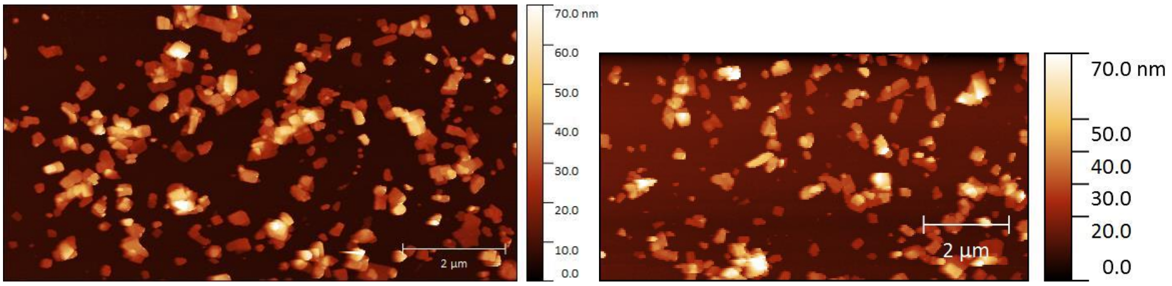

Figure S25. AFM images and example height plot used for the particle sizing of Cu(**2**)(DMF) exfoliated for 12 hrs and centrifuged at 4500 rpm for 4 hrs. N.B. scanning down the image on the left, the cantilever disengaged from the mica substrate. The tip was re-engaged and scanning continued, producing the image on the right. N.B Particle sizing analysis was carried out on the right hand image, as this gave n=138 nanosheets.

Cu(2)(DMF) exfoliated in MeCN for 30 mins with sequential centrifugation

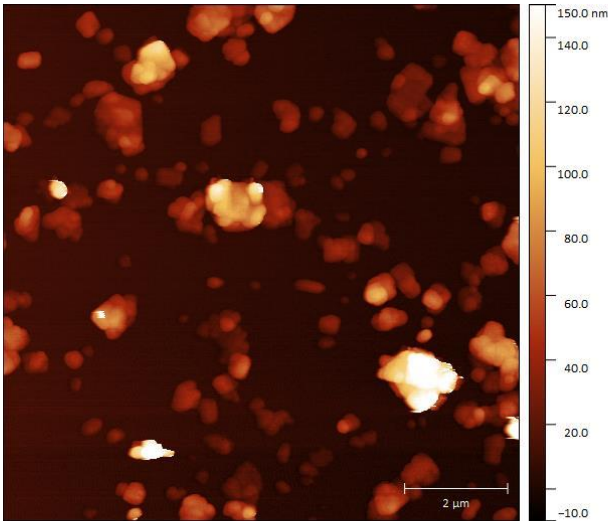

Figure S 26. AFM images and example height plot used for the particle sizing of Cu(**2**)(DMF) exfoliated for 30 mins and centrifuged at 1500 rpm for 30 mins.

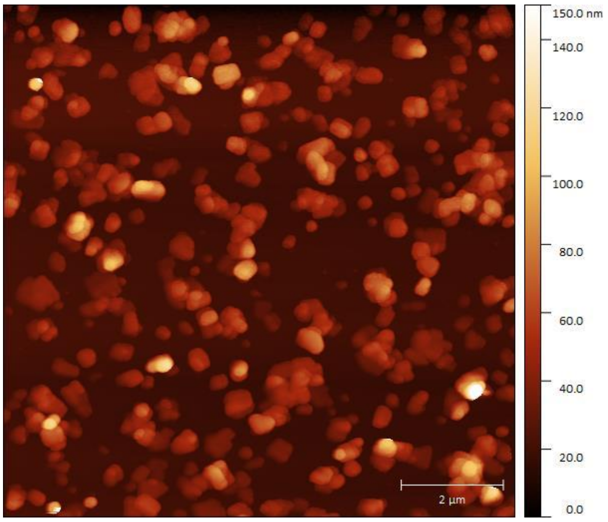

Figure S 27. AFM images and example height plot used for the particle sizing of Cu(**2**)(DMF) exfoliated for 30 mins and centrifuged at 4500 rpm for 4 hrs.

Table S 8. Size distribution statistics of the samples portrayed above. All lengths are in nm.

Sample	Cu(**1**)(DMF)			Cu(**2**)(DMF)			
Exfoliation Time	12 hr			12 hr		30 min	
Centrifugation	1500 rpm, 1 hr	4500 rpm, 30 mins	4500 rpm, 4 hrs	1500 rpm, 1 hr	4500 rpm, 4 hrs	1500 rpm, 1 hr	4500 rpm, 4 hrs
# Nanosheets	95	111	161	94	138	117	223
Mean lateral dimension*	512	347	307	348	367	468	413
SD lateral dimension*+	234	153	108	202	155	206	132
Mean height	59	49	41	20	19	28	26
SD height+	35	26	19	12	10	18	15
% > 600 nm lateral	30	9	2	14	11	24	8
% < 400 nm lateral	37	73	83	63	61	42	50
% < 40 nm height	32	41	57	93	97	83	86
% < 30 nm height	18	30	44	83	90	69	73
% > aspect ratio of 10$	42	23	39	83	92	90	90

* Lateral dimension was recorded as the largest edge-to-edge distance across the nanosheet, to the nearest 10 nm. + SD = standard deviation. $ Aspect ratio calculated as recorded lateral dimension divided by the average height of a nanosheet.

5.4 DLS Studies

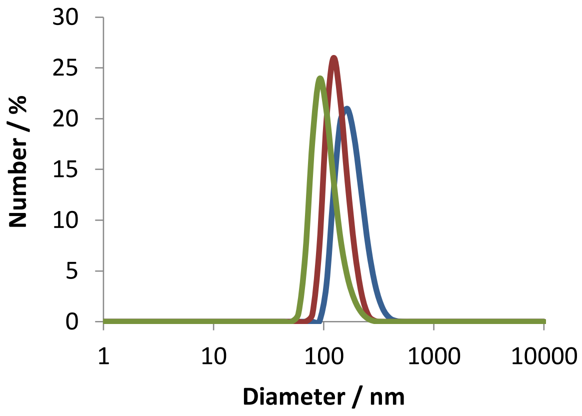

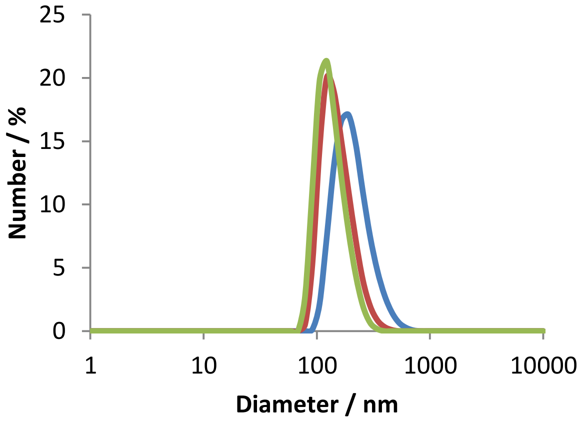

Figure S 28. Number average of three repeat collections of DLS data. Cu(**1**)(DMF) (left) and Cu(**2**)(DMF) (right) exfoliated for 12 hrs in MeCN then cascade centrifuged at 1500 rpm for 1 hr (blue), then 4500 rpm for 30 mins (red), then 4500 rpm for 4 hrs (green).

Table S 9. DLS data: average determined diameter of MONs using Z-average, intensity average and number average, and polydispersity index (PdI)

**Sample**		Cu(**1**)(DMF)			Cu(**2**)(DMF)	
**Centrifugation**	1500 rpm, 1 hr	4500 rpm, 30 mins	4500 rpm, 4 hrs	1500 rpm, 1 hr	4500 rpm, 30 mins	4500 rpm, 4 hrs
**Z-average (diameter) / nm**	204	153	135	276	195	168
**Intensity average (diameter) / nm**	220	159	146	320	213	181
**Number average (diameter) / nm**	179	135	106	213	155	137
**PdI**	0.075	0.0183	0.068	0.163	0.118	0.064

It should be noted that size averages determined through these DLS measurements do not match exactly with those determined through statistical analysis of topographical AFM images. DLS measurements are based on a translational diffusion coefficient, which is converted to a hydrodynamic diameter using the Stokes-Einstein equation. This equation is valid for spherical particles. It is widely known that diffusion of 2D nanosheets with high aspect ratios is not well characterised by DLS, and the presence of periodic porosity within the MON structures may complicate the diffusion behaviour further. As such, information is provided on the relative size of the MONs in dispersion; however absolute values should not be taken from this analysis.

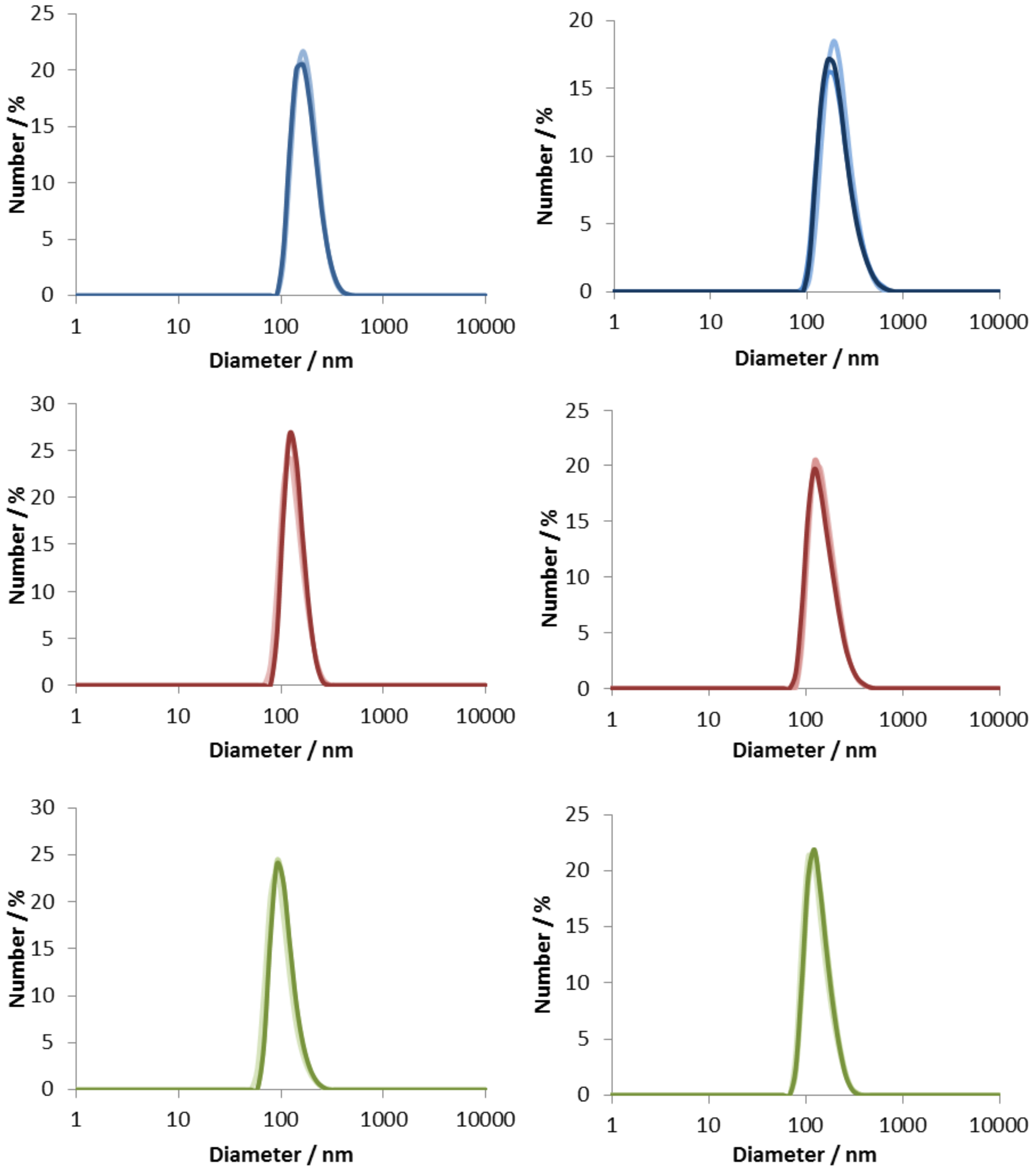

Figure S 29. DLS data from three runs for each respective sample. Cu(**1**)(DMF) (left) and Cu(**2**)(DMF) (right) exfoliated in MeCN for 12 hrs and then cascade centrifuged at 1500 rpm for 1 hr (blue), then 4500 rpm for 30 mins (red), then 4500 rpm for 4 hrs (green). Each run is an average of between 10-18 analyses (number computationally selected in an automated data collection optimisation).

5.5 Exfoliation Studies in Various Solvents

6 mL of solvent was added to 5 mg of Cu(**1**)(DMF) or Cu(**2**)(DMF). These were exfoliated for 12 hrs, and then the resulting mixtures were centrifuged at 1500 rpm for 1 hr. 5 mL of supernatant was removed, and this suspension of nanosheets in solvent was used for AFM analysis. Typically, 10 μL of suspension was drop cast onto a freshly-cleaved mica substrate, which was held at a temperature around the boiling point of the solvent used in order to aid the evaporation of solvent, which has been previously suggested to aid in nanosheet characterisation, as this may prevent nanosheet aggregation throughout a slow evaporative process. Representative AFM images of deposited material from these suspensions, using the solvents DMF, NMP, water, diethyl ether and acetonitrile, can be found below.

DMF

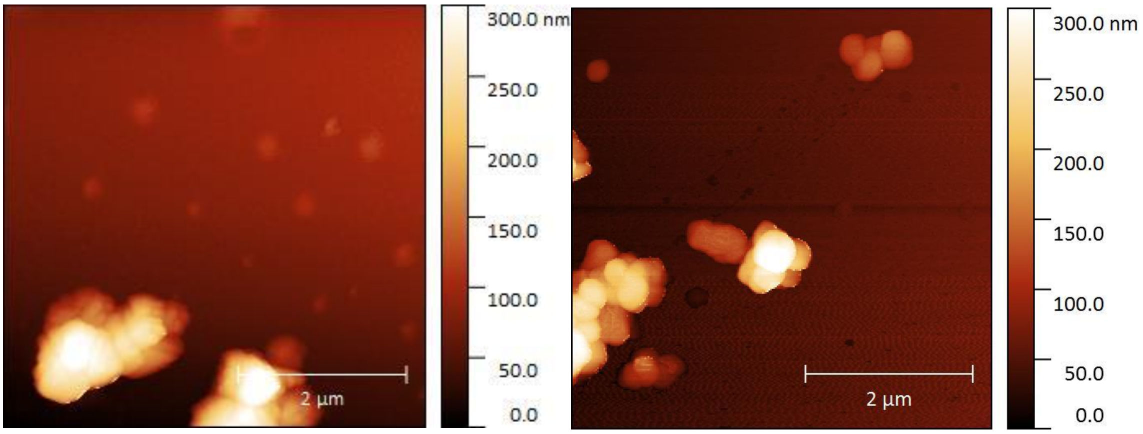

Figure S30. AFM images of Cu(**1**)(DMF) exfoliated in DMF.

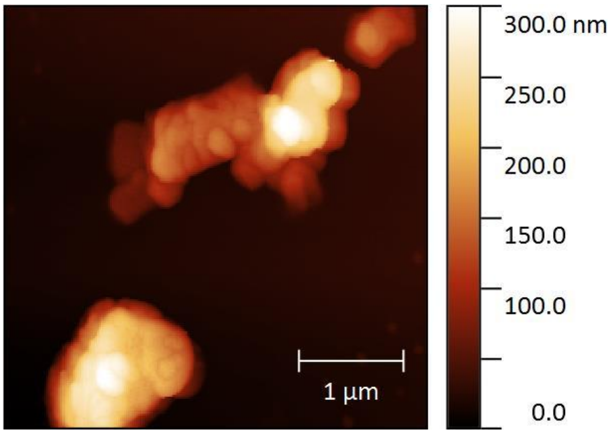

Figure S31. AFM image of Cu(**2**)(DMF) exfoliated in DMF.

NMP

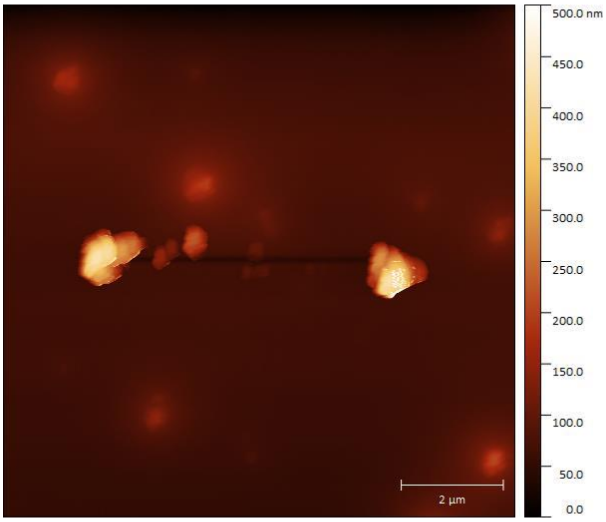

Figure S32. AFM images of Cu(**1**)(DMF) exfoliated in NMP.

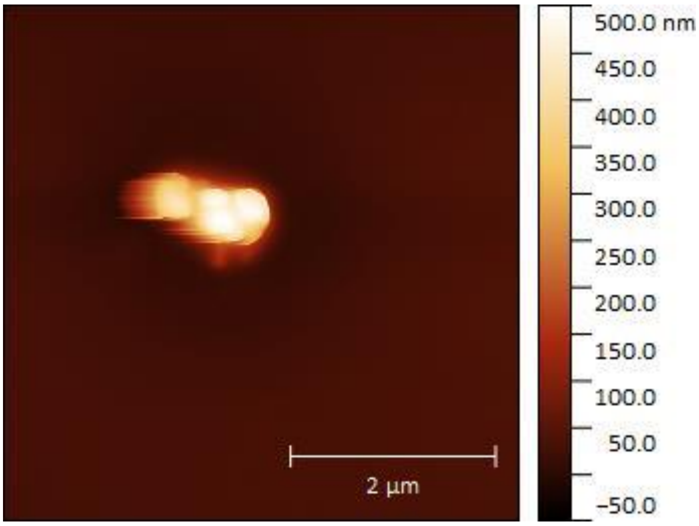

Figure S33. AFM images of Cu(**2**)(DMF) exfoliated in NMP. N.B. Very little was observed under AFM, however attached light microscope showed what appear to be a large number of particles across the substrate surface, potentially indicating a high level of nanosheet/particle agglomeration.

Water

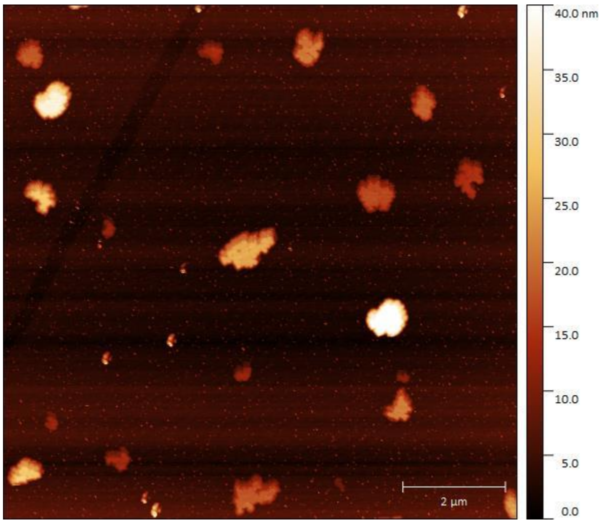

Figure S34. AFM images of Cu(**1**)(DMF) exfoliated in water.

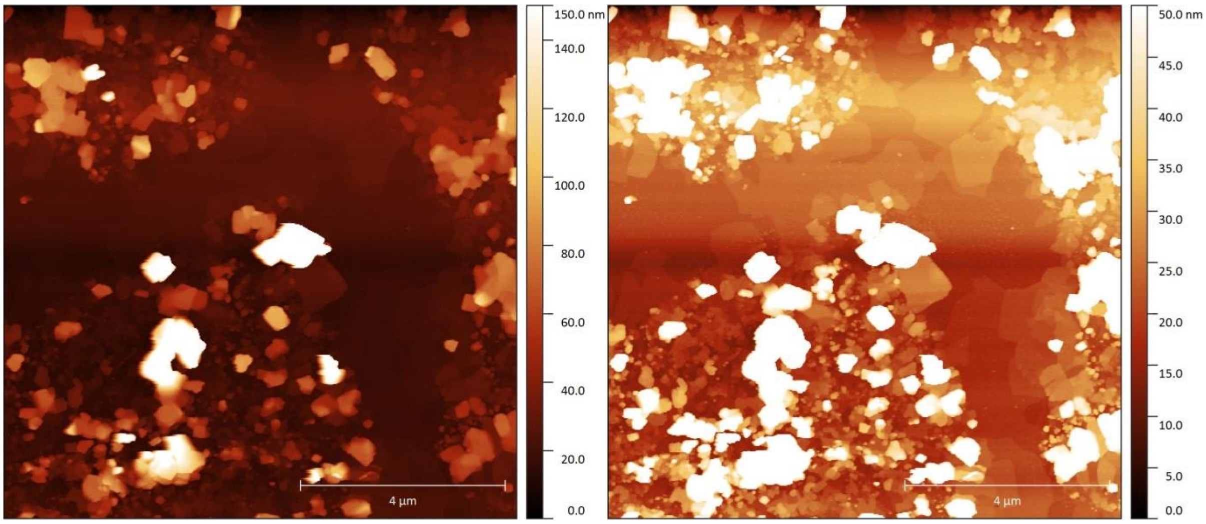

Figure S35. AFM images of Cu(**2**)(DMF) exfoliated in water. Both images are identical, with different height scales, to illustrate both larger and thinner nanosheets.

Diethyl ether

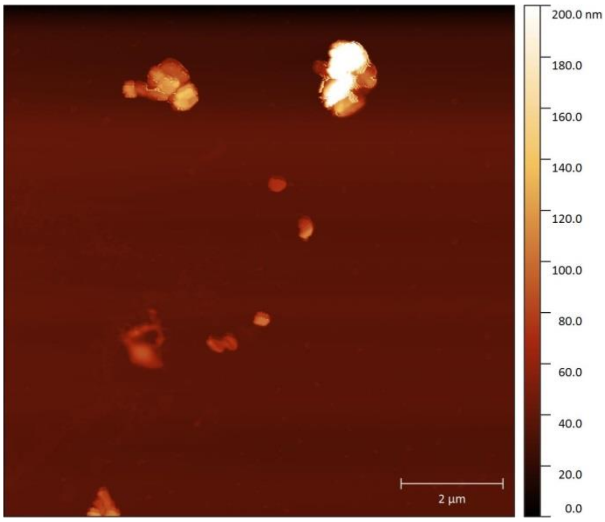

Figure S36. AFM images of Cu(**1**)(DMF) exfoliated in diethyl ether.

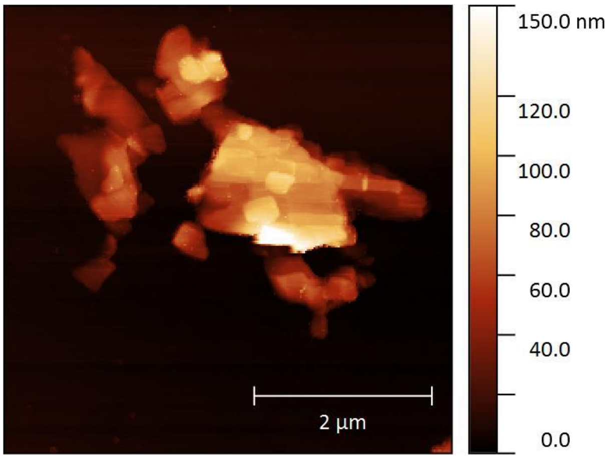

Figure S37. AFM images of Cu(**2**)(DMF) exfoliated in diethyl ether.

Acetonitrile

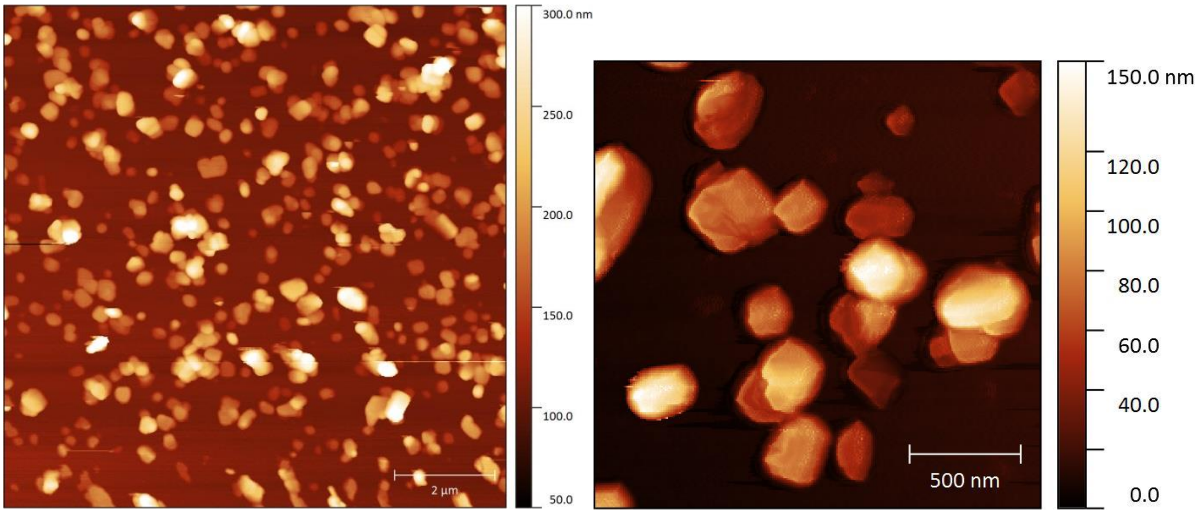

Figure S38. AFM images of Cu(**1**)(DMF) exfoliated in acetonitrile.

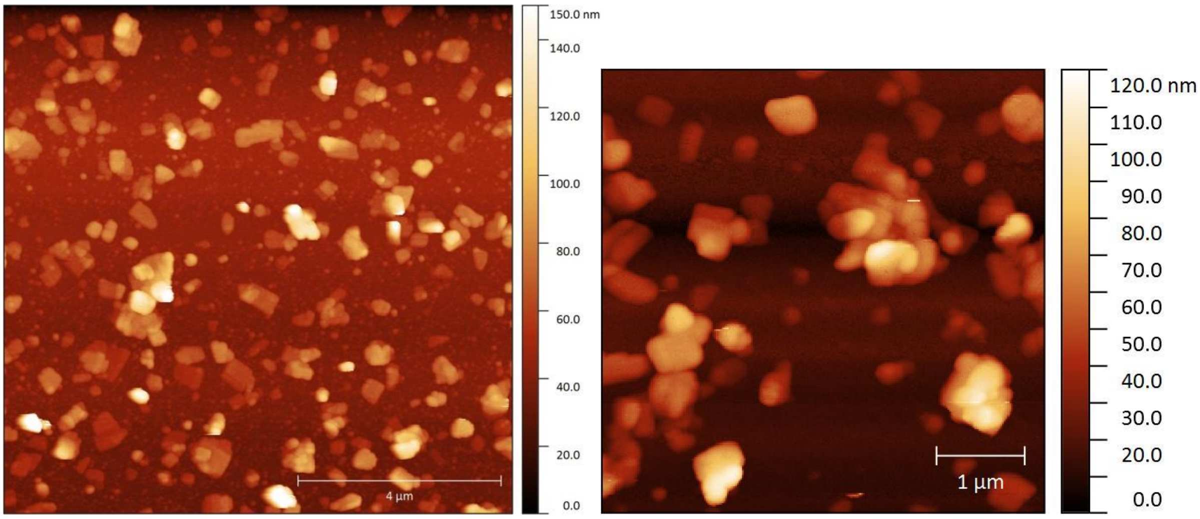

Figure S39. AFM images of Cu(**2**)(DMF) exfoliated in acetonitrile.

6. Structural Analysis

5.1 Powder X-Ray Diffraction

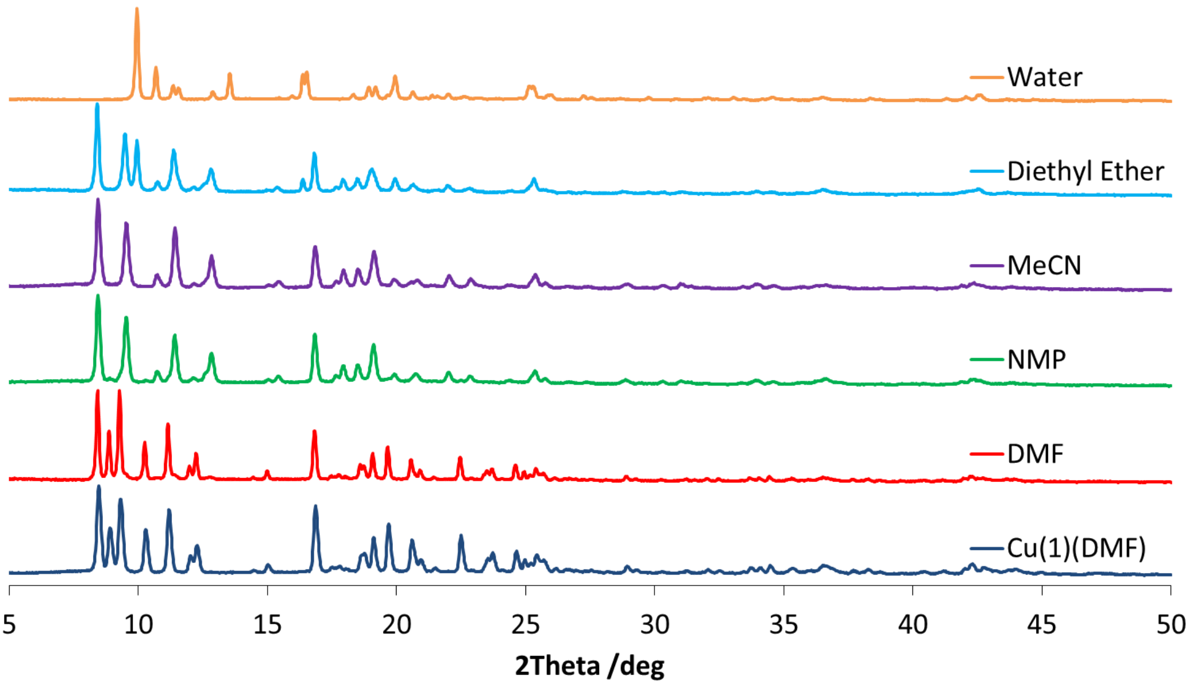

Figure S40. XRPD patterns for parent Cu(**1**)(DMF) as synthesised and following exfoliation in named solvents

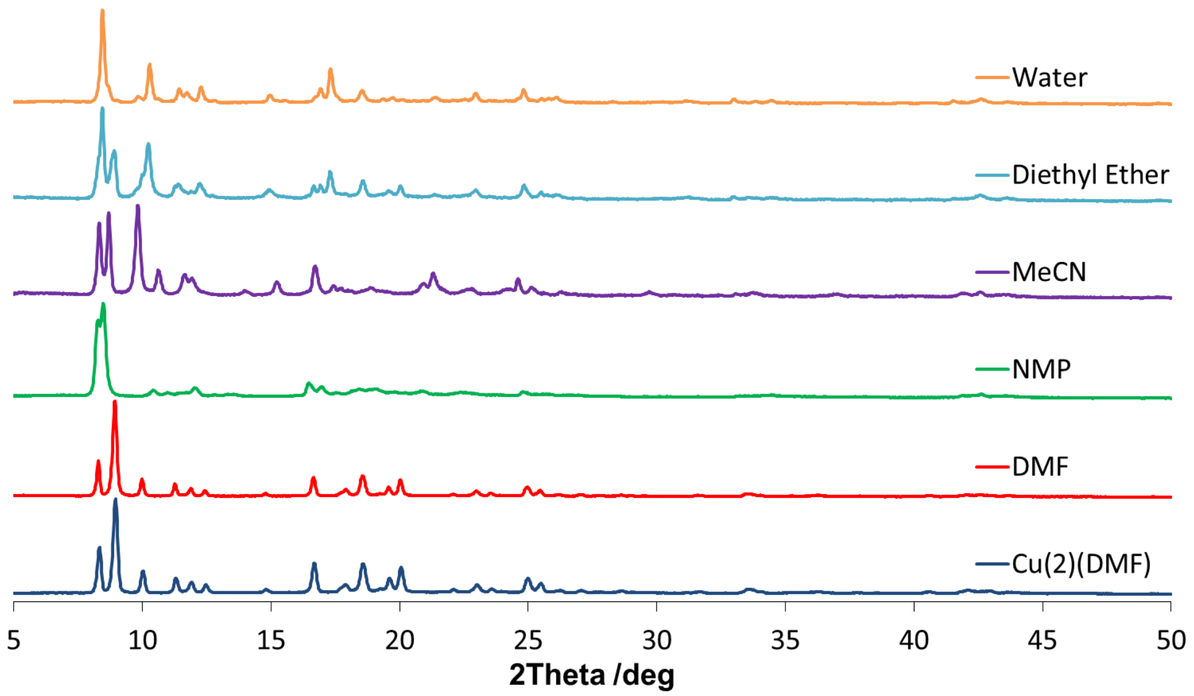

Figure S41. XRPD patterns for parent Cu(**2**)(DMF) as synthesised and following exfoliation in named solvents

6.2 Fourier Transform Infrared Spectroscopy

FTIR spectra were recorded using a Perkin-Elmer Spectrum Two FTIR spectrometer equipped with a diamond UATR (universal attenuated total reflectance) accessory. A background of air was taken. A spatula tip of sample was then loaded onto the stage and pressure was applied. Spectra were recorded in reflectance mode with a scan range of 4000-500 cm^{-1}, a resolution of 1 cm^{-1}, with 8 scans recorded.

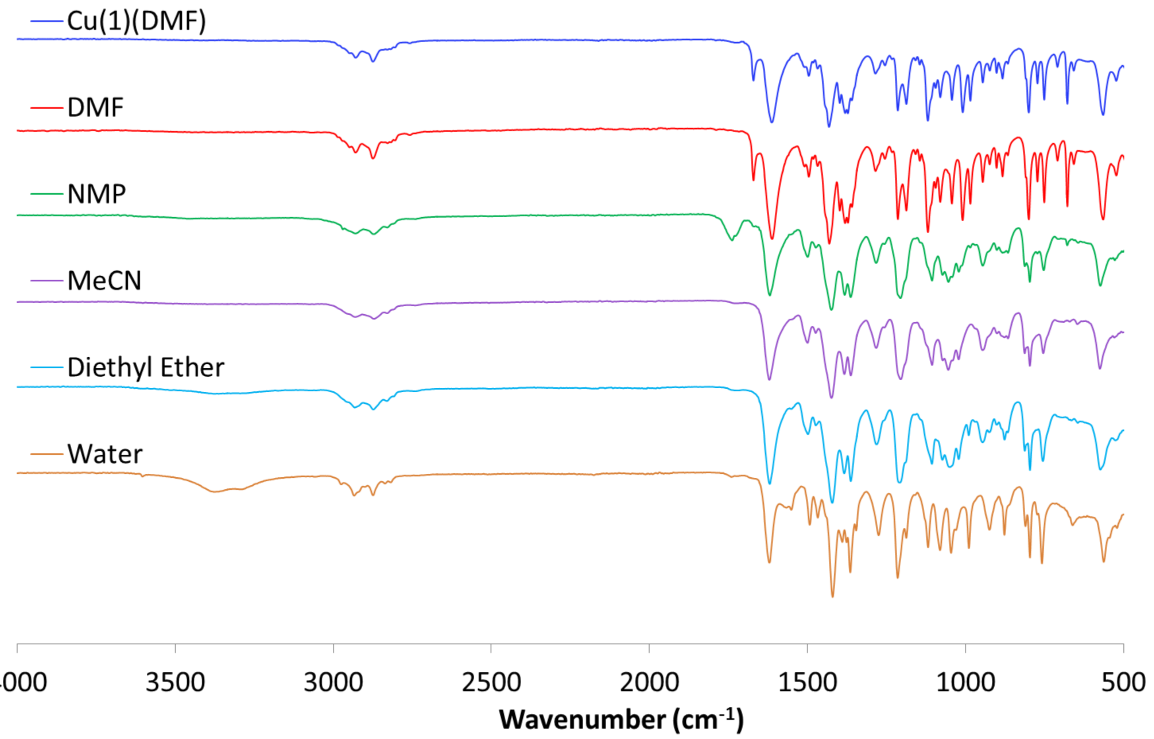

Figure S42. IR spectra for parent Cu(**1**)(DMF) as synthesised and following exfoliation in named solvents.

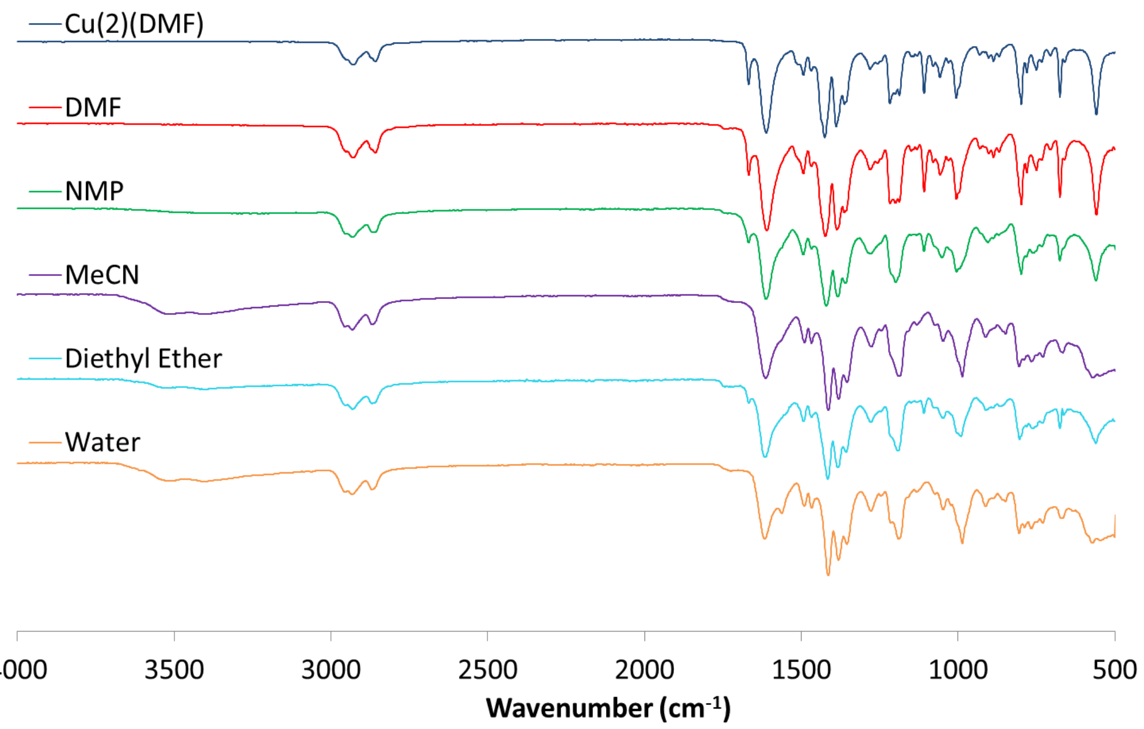

Figure S43. IR spectra for parent Cu(**2**)(DMF) as synthesised and following exfoliation in named solvents.

6.3 Thermogravimetric Analysis

Thermogravimetric analyses were performed using a Perkin-Elmer Pyris 1 TGA instrument. 4 – 6 mg was accurately weighed into a ceramic TGA pan and loaded into the instrument. Sample was held under a constant 20 mL min^{-1} N_2 flow. An initial equilibration period of 10 mins at 30 °C was used, and then sample was heated at a continuous rate of 10 °C min^{-1} to the end temperature.

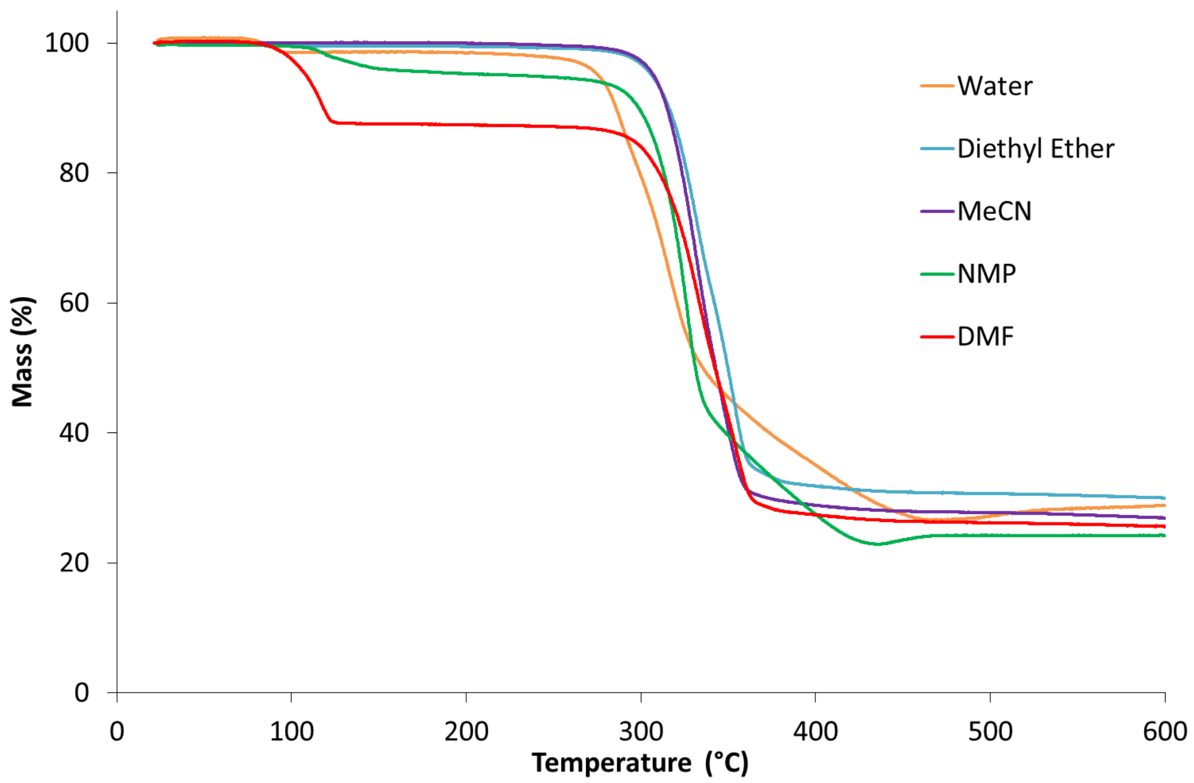

Figure S44. TGA thermograms for parent Cu(**1**)(DMF) following exfoliation in named solvents.

Table S10. Summary of TGA thermogram data below 250°C for bulk Cu(**1**)(DMF) and following exfoliation in different solvents.

Sample	Suggested Solvent Lost	Event Temperature / °C	Difference %
Cu(**1**)(DMF)	DMF	65-123	13.5
DMF	DMF	68-135	12.3
NMP	NMP	83-205	4.2
MeCN	MeCN	No solvent loss	
Diethyl Ether	Et_2O	66-86	0.5
Water	H_2O	66-94	1.4

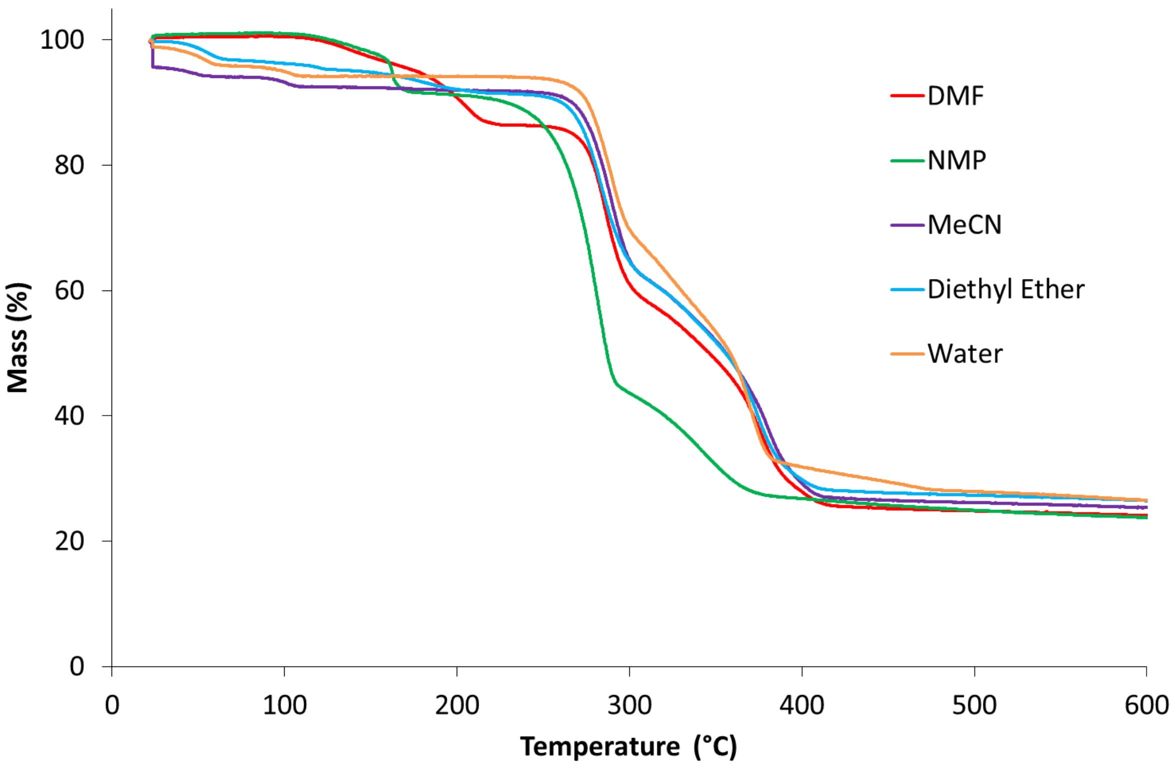

Figure S45. TGA thermograms for parent Cu(**2**)(DMF) following exfoliation in named solvents.

Table S11. Summary of TGA thermogram data below 250°C for bulk Cu(**2**)(DMF) and following exfoliation in different solvents.

Sample	Suggested Solvent Lost	Event Temperature / °C	Difference %
Cu(**2**)(DMF)	DMF	65-123	13.6
DMF	DMF	103-223	13.4
NMP	H_2O/NMP	94-178	8.6
MeCN	MeCN/H_2O	56-115	3.1
Diethyl Ether	H_2O	66-127	4.7
Water	H_2O	71-107	4.6

6.4 Elemental Analysis

Table S12. Calculated and experimental elemental analysis data for Cu(**1**)(DMF) derivatives

Calculated Structure	Calculated / wt% C	H	N	Exfoliation Solvent	Experimental / wt% C	H	N
Cu(**1**)	47.58	4.99	0				
Cu(**1**)(DMF)	47.85	5.71	2.94	DMF	44.00	5.26	2.45
Cu(**1**)(NMP)	50.14	5.81	2.78	NMP	43.38	4.84	0.72
Cu(**1**)(MeCN)	47.39	4.91	3.25	MeCN	44.35	4.76	0
Cu(**1**)(Et_2O)	50.26	6.33	0	Diethyl ether	41.27	4.43	0
Cu(**1**)(H_2O)	45.55	5.26	0	Water	39.83	5.15	0

Table S13. Calculated and experimental elemental analysis data for Cu(**2**)(DMF) derivatives

Calculated Structure	Calculated / wt% C	H	N	Exfoliation Solvent	Experimental / wt% C	H	N
Cu(**2**)	54.06	6.05	0				
Cu(**2**)(DMF)	58.59	7.52	2.99	DMF	51.1	6.15	2.76
Cu(**2**)(NMP)	55.35	6.67	2.81	NMP	51.5	6.14	2.21
Cu(**2**)(MeCN)	53.45	5.90	3.28	MeCN	45.98	6.13	0
Cu(**2**)(Et_2O)	55..74	7.23	0	Diethyl ether	47.79	6.23	0.59
Cu(**2**)(H_2O)	51.73	6.27	0	Water	45.64	5.96	0

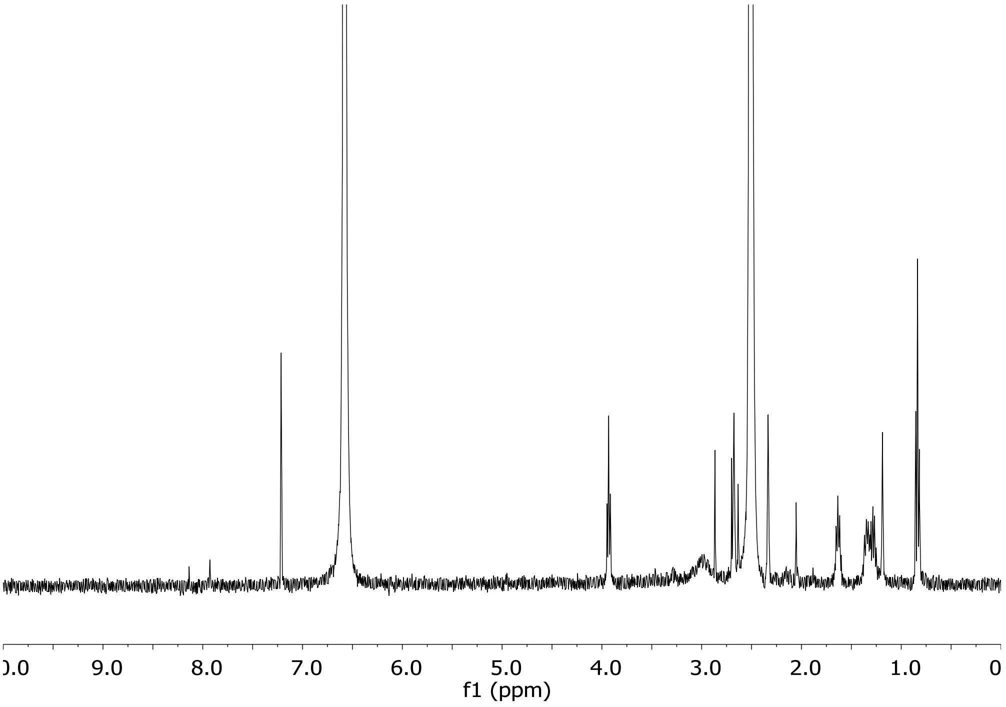

Figure S46. NMR showing Cu(2)(DMF) following exfoliation in NMP, digested in DCl(aq)/DMSO.

7. Binding Studies

7.1 Estimation of Nanosheet Concentration in Suspension

Compounds Cu(**1**)(DMF) or Cu(**2**)(DMF) (5 mg) were suspended in H_2O (6 mL) and sonicated for 12 hrs, using 80 kHz, 100 % power, 21 °C. The resulting suspensions of Cu(**1**)(H_2O) and Cu(**2**)(H_2O) were centrifuged at 1500 rpm for 1 hr in order to collect larger unexfoliated particles from suspension, giving MON suspensions of 0.65 mM and 0.20 mM respectively. Cu(**1**)(H_2O) was diluted in water (1 in 5) to give a suspension with an absorption maximum at 0.37 AU. Using the extinction coefficient determined in section 1.2.2, this indicates a concentration of 0.13 mM. Cu(**2**)(H2O) suspension was diluted in water (1 in 2.5), giving an absorption maxima of 0.25 AU, indicating a concentration of 0.08 mM. These were used as host suspensions.

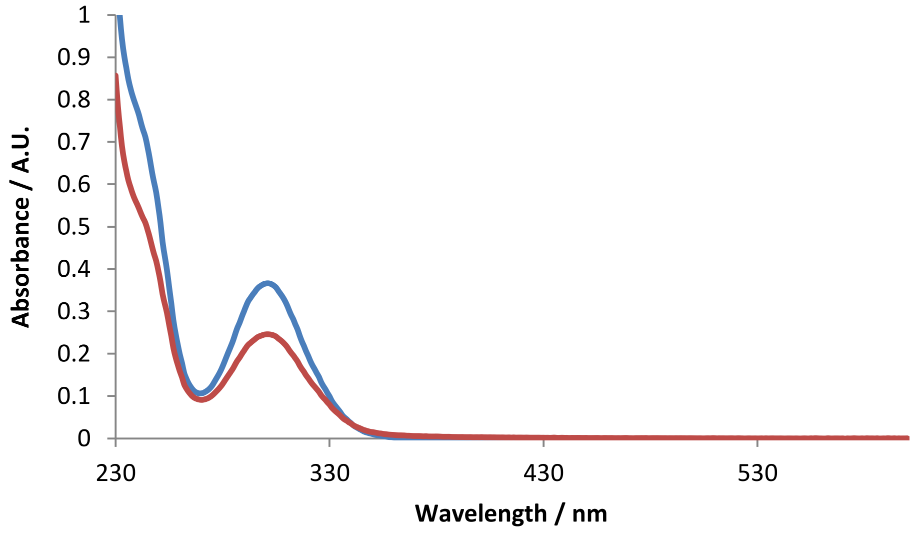

Figure S47. UV-vis spectra of suspensions of Cu(**1**)(H_2O) (0.13 mM) (blue) and Cu(**2**)(H_2O) (0.08 mM) (red) in water.

7.2 Binding Studies

Imidazole (10 mg and 6 mg), respectively was dissolved in 2 mL of the Cu(**1**)(H_2O) and Cu(**2**)(H_2O) suspensions, giving 43 mM and 73 mM aqueous solutions, respectively. These were used as the guest solutions. UV titration bidning experiments were performed three separate times in each case. Binding constants were calculated by fitting the experimental data to a binding isotherm using 14Allmaster, a macro based excel fitting programme written by Prof. Christopher A. Hunter (University of Cambridge). Values are reported as the global average of the three repeat measurements, with the error quoted as two standard deviations from the mean. Tyndall scattering confirmed the presence of nanosheets following addition of imidazole.

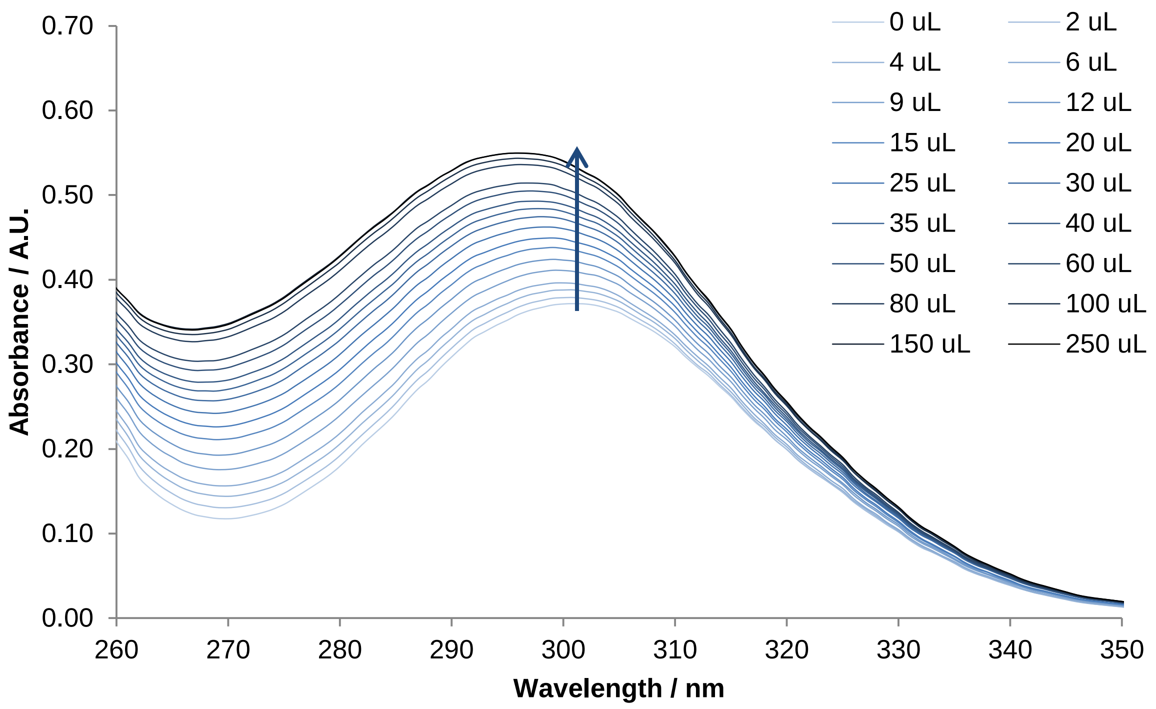

Figure S48. UV-vis plot showing the addition of aliquots of X uL imidazole solution (73 mM in water) to 2.5 mL of aqueous suspension of Cu(**1**)(H_2O) (0.13 mM). Aliquots of volume 0, 2, 6, 9, 12, 15, 20, 25, 30, 35, 40, 50, 60, 80, 100, 150 and 250 uL were used.

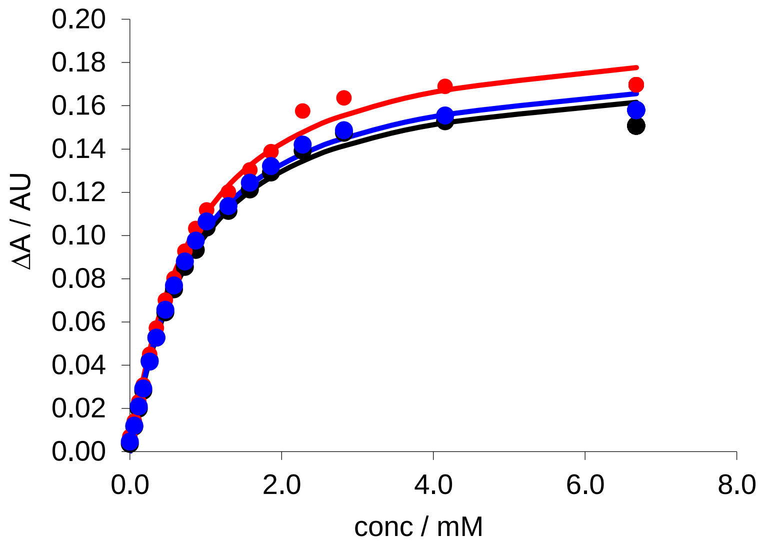

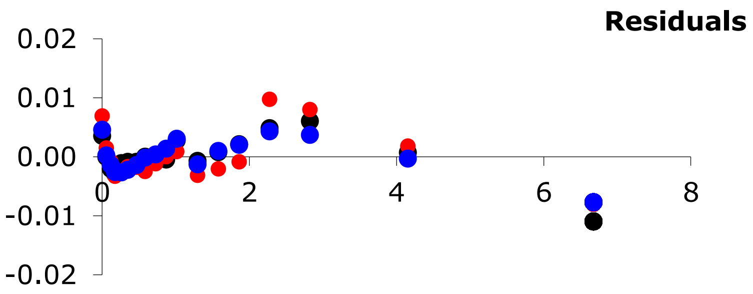

Figure S49. UV-vis binding titration showing the change in absorbance at λmax = 301 nm for Cu(**1**)(H_2O) with increasing concentration of imidazole. The three experiments were repeated three times, with individual experiments shown in red, black and blue. Residuals for the fits are shown below.

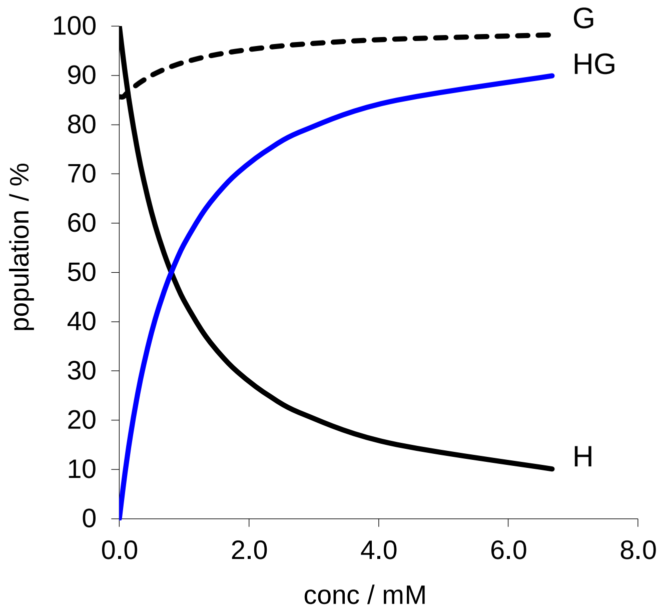

Figure S50. Speciation plot showing formation of HG complex upon addition of imidazole (G) to a suspension of Cu(**1**)(H_2O) nanosheets (H).

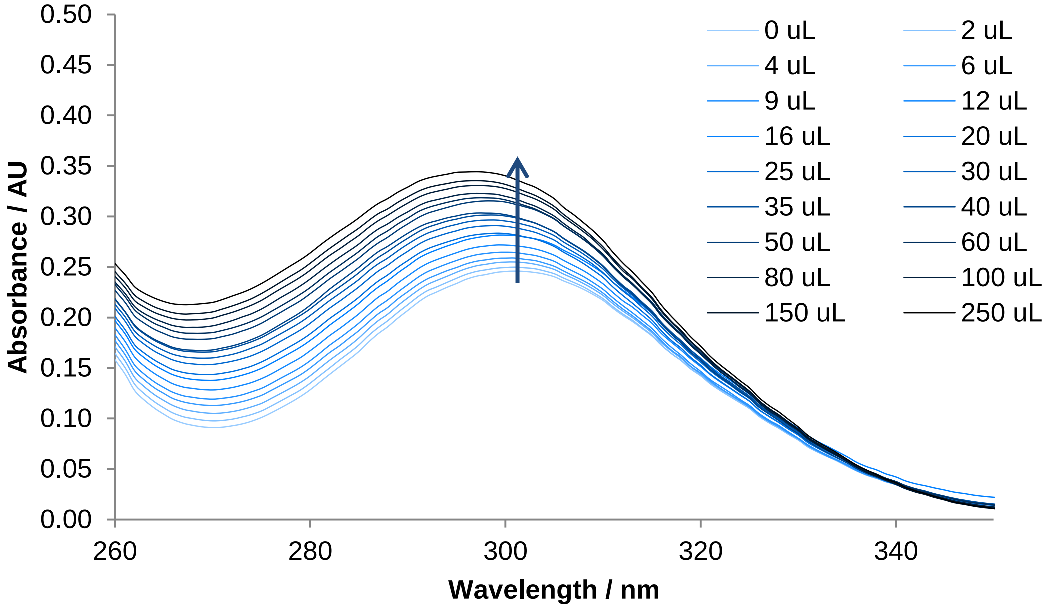

Figure S51. UV-vis plot showing the addition of aliquots of X uL imidazole solution (43 mM in water) to 2.5 mL of aqueous suspension of Cu(**2**)(H_2O) (0.08 mM). Aliquots of volume 0, 2, 4, 6, 9, 12, 15, 20, 25, 30, 35, 40, 50, 60, 80, 100, 150 and 250 uL were used.

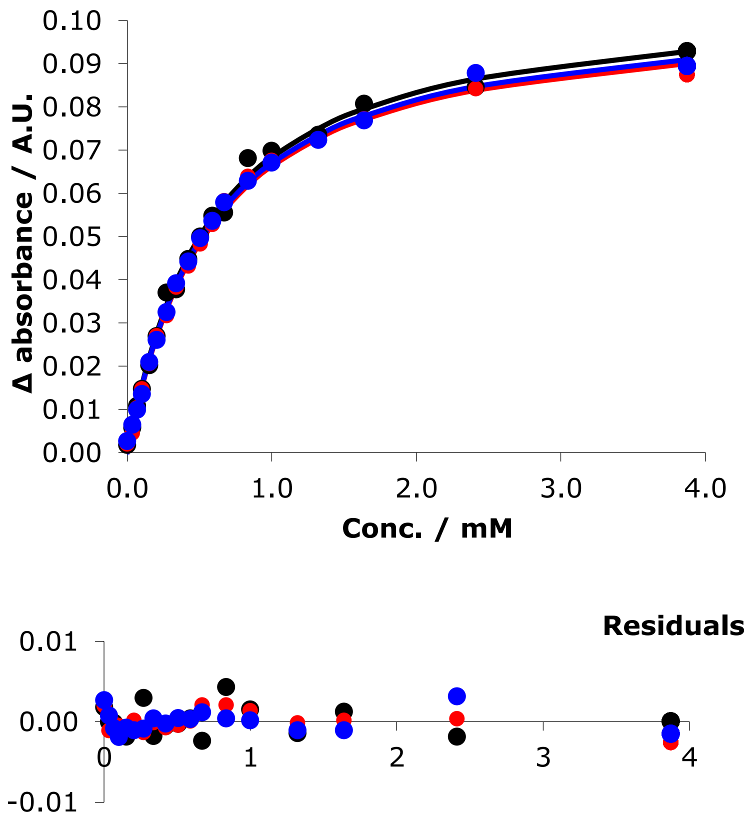

Figure S52. UV-vis binding titration showing the change in absorbance at λmax = 301 nm for Cu(**2**)(H_2O) with increasing concentration of imidazole. The three experiments were repeated three times, with individual experiments shown in red, black and blue. Residuals for the fits are shown below.

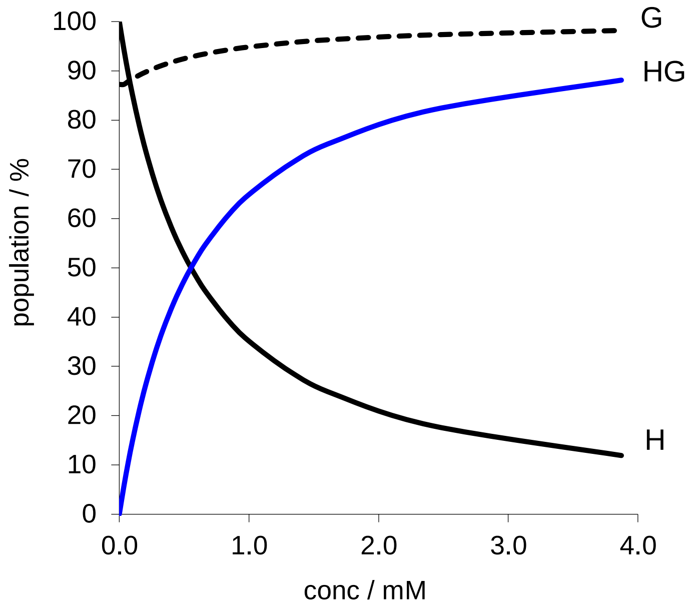

Figure S 53. Speciation plot showing formation of HG complex upon addition of imidazole (G) to a suspension of Cu(**2**)(H_2O) nanosheets (H).

Detection limit and sensitivity

The sensitivity of imidazole sensing depends on both the [host] and [guest] and the response curve is steepest following addition of the first aliquot of imidazole solution. The concentration of host was maintained at 0.13 mM and 0.08 mM for Cu(**1**)(H_2O) and Cu(**2**)(H_2O) respectively and the concentration of the guest in solution following addition of the first aliquot was 59 μM and 34 μM. This resulted in a positive Δ(absorbance) at λ_{max} in the UV spectra of +0.0075 and +0.0034 A.U. for Cu(**1**)(H_2O) and Cu(**2**)(H_2O). The standard deviation in absorbance of the three blank host runs was 0.0026 and 0.0008 respectively – both lower than the average Δ(absorbance). The detection limit of Cu(**1**)(H_2O) and Cu(**2**)(H_2O) for imidazole is therefore below 59 μM and 34 μM respectively."

8. References

1. S. Henke, A. Schneemann, A. Wutscher and R. A. Fischer, *J Am Chem Soc*, 2012, **134**, 9464-9474.
2. S. Henke and R. A. Fischer, *J Am Chem Soc*, 2011, **133**, 2064-2067.
3. J. A. Foster, S. Henke, A. Schneemann, R. A. Fischer and A. K. Cheetham, *Chemical Communications*, 2016, **52**, 10474-10477.

CONTENTS

S6. DFT Calculations S47
- S6.1. Calculations on $Cu_2(\mathbf{1}^{**})_4$ S51
  - S6.1.1. Cartesian Co-ordinates (XYZ format) S51
  - S6.1.2. Frequencies S55
- S6.2. Calculations on $Cu_2(\mathbf{1}^{**})_4(H_2O)_2$ S63
  - S6.2.1. Cartesian Co-ordinates (XYZ format) S63
  - S6.2.2. Frequencies S67
- S6.3. Calculations on $Cu_2(\mathbf{1}^{**})_4(DMF)_2$ S76
  - S6.3.1. Cartesian Co-ordinates (XYZ format) S76
  - S6.3.2. Frequencies S80
- S6.4. Calculations on $Cu_2(\mathbf{1}^{**})_4(MeCN)_2$ S90
  - S6.4.1. Cartesian Co-ordinates (XYZ format) S90
  - S6.4.2. Frequencies S94
- S6.5. Calculations on $Cu_2(\mathbf{1}^{*})_4$ S103
  - S6.5.1. Cartesian Co-ordinates (XYZ format) S103
  - S6.5.2. Frequencies S106
- S6.6. Calculations on $Cu_2(\mathbf{1}^{*})_4(H_2O)_2$ S112
  - S6.6.1. Cartesian Co-ordinates (XYZ format) S112
  - S6.6.2. Frequencies S115
- S6.7. Calculations on $Cu_2(\mathbf{1}^{*})_4(MeCN)_2$ S121
  - S6.7.1. Cartesian Co-ordinates (XYZ format) S121
  - S6.7.2. Frequencies S124
- S6.8. Calculations on $Cu_2(\mathbf{1}^{*})_4(DMF)_2$ S130
  - S6.8.1. Cartesian Co-ordinates (XYZ format) S130
  - S6.8.2. Frequencies S133
- S6.9. Calculations on $Cu_2(\mathbf{2}^{*})_4(DMF)_2$ S140
  - S6.9.1. Cartesian Co-ordinates (XYZ format) S140
  - S6.9.2. Frequencies S143
- S6.10. Calculations on $Cu_2(\mathbf{2}^{*})_4(MeCN)_2$ S150
  - S6.10.1. Cartesian Co-ordinates (XYZ format) S150
  - S6.10.2. Frequencies S153
- S6.11. Calculations on $Cu_2(\mathbf{2}^{*})_4(H_2O)_2$ S160
  - S6.11.1. Cartesian Co-ordinates (XYZ format) S160
  - S6.11.2. Frequencies S163

S6. DFT CALCULATIONS

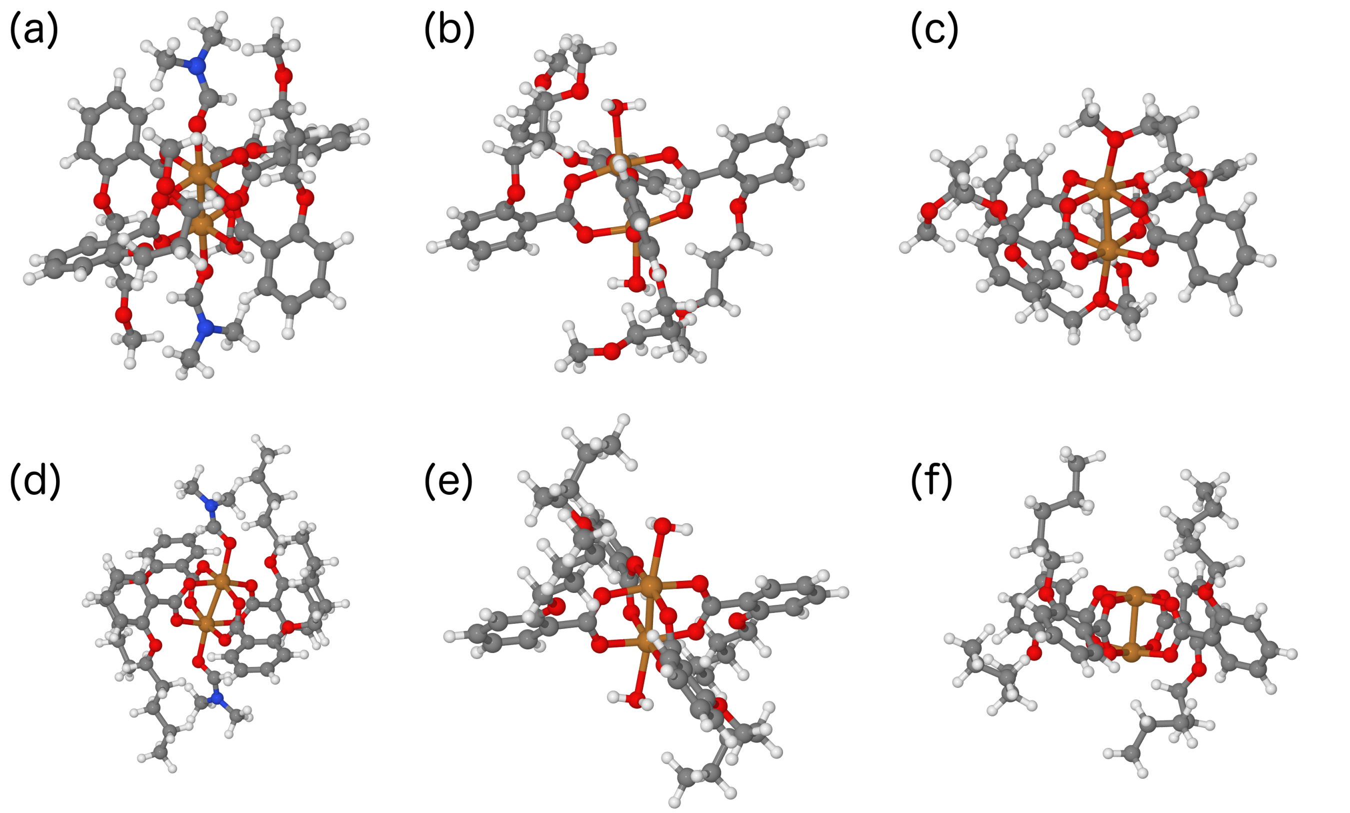

FIG. S54. DFT calculations showing optimised structures for (a) $Cu_2(1^*)_4(DMF)_2$, (b) $Cu_2(1^*)_4(H_2O)_2$, (c) $Cu_2(1^*)_4$, (d) $Cu_2(2^*)_4(DMF)_2$, (e) $Cu_2(2^*)_4(H_2O)_2$, (f) $Cu_2(2^*)_4$ where 1^* is 2-(3-methoxypropoxy)benzoate and 2^* is 2-(pentyloxy)benzoate.

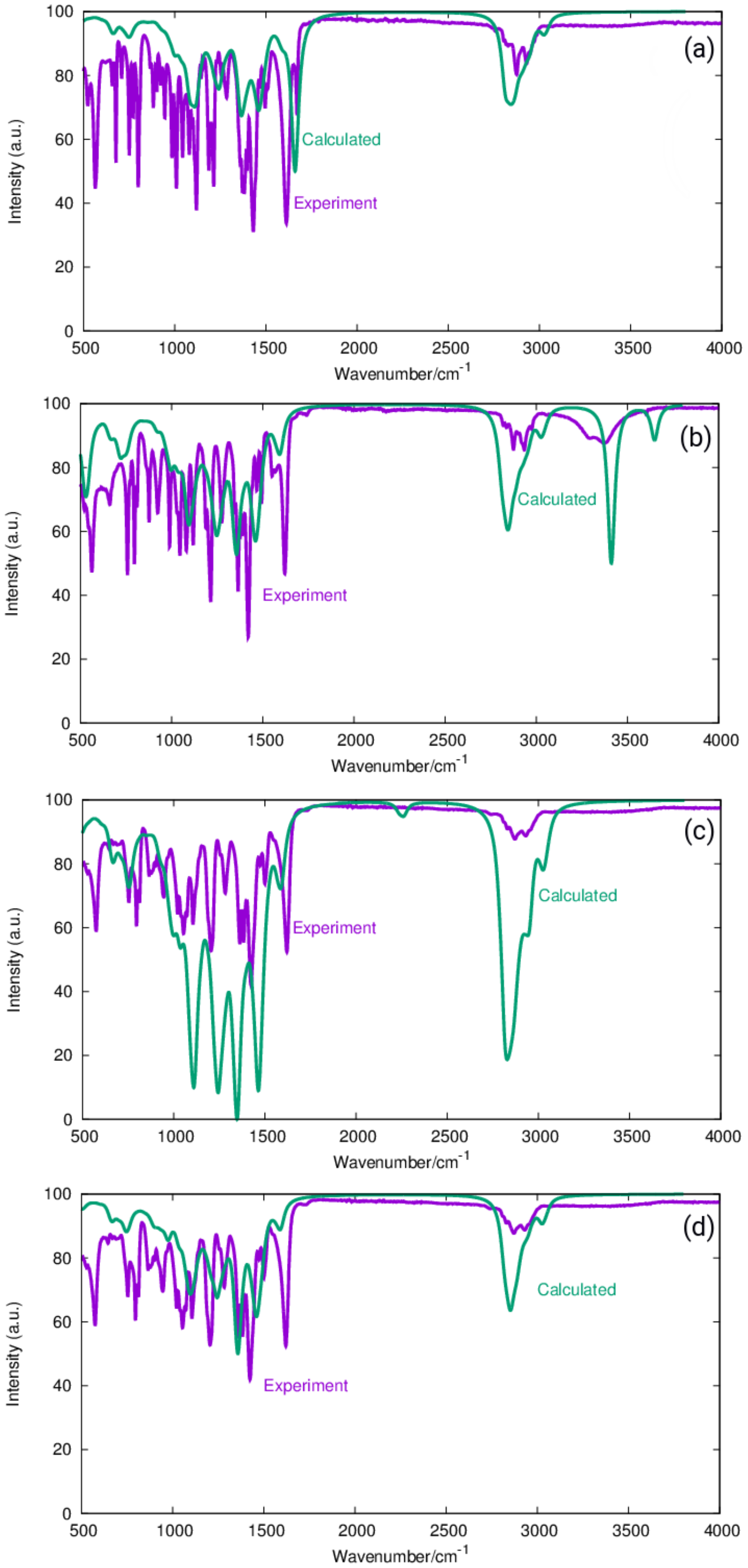

FIG. S55. Comparison of calculated (green) and experimental (purple) IR patterns for (a) $Cu_2(1^*)_4(DMF)_2$ with Cu(**1**)(DMF) exfoliated in DMF, (b) $Cu_2(1^*)_4(H_2O)_2$ with Cu(**1**)(DMF) exfoliated in H_2O, (c) $Cu_2(1^*)_4(CH_3CN)_2$ with Cu(**1**)(DMF) exfoliated in CH_3CN [theoretical intensity *2] (d) $Cu_2(1^*)_4$ with Cu(**1**)(DMF) exfoliated in CH_3CN where 1^* is 2-(3-methoxypropoxy)benzoate. All theoretical spectra are scaled, so that the maximum intensity is of a similar size to the experimental maximum intensity.

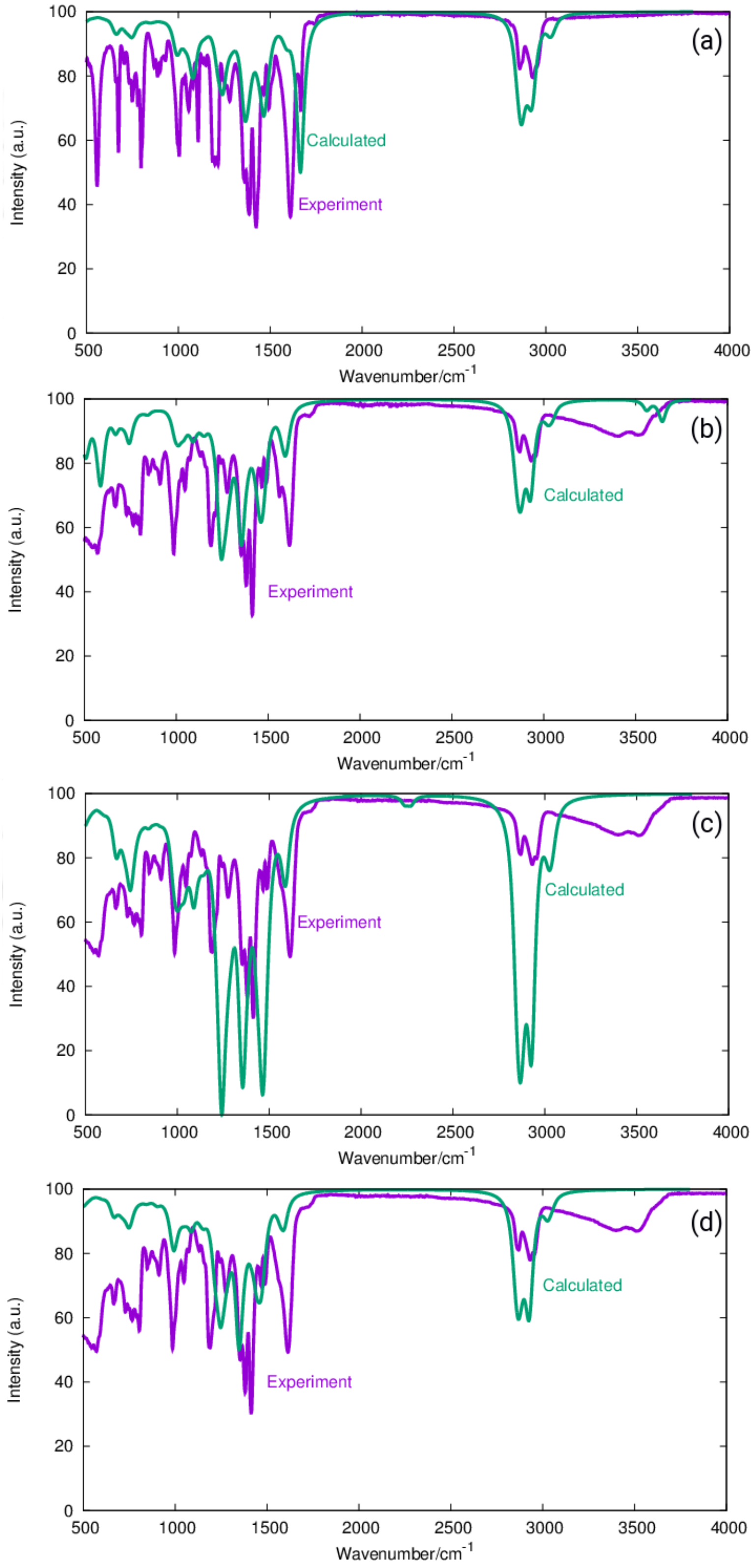

FIG. S56. Comparison of calculated (green) and experimental (purple) IR patterns for (a) $Cu_2(2^*)_4(DMF)_2$ with Cu(**2**)(DMF) exfoliated in DMF, (b) $Cu_2(2^*)_4(H_2O)_2$ with Cu(**2**)(DMF) exfoliated in H_2O, (c) $Cu_2(2^*)_4(CH_3CN)_2$ with Cu(**2**)(DMF) exfoliated in CH_3CN and (d) $Cu_2(2^*)_4$ with Cu(**2**)(DMF) exfoliated in CH_3CN where 2^* is 2-(pentyloxy)benzoate.

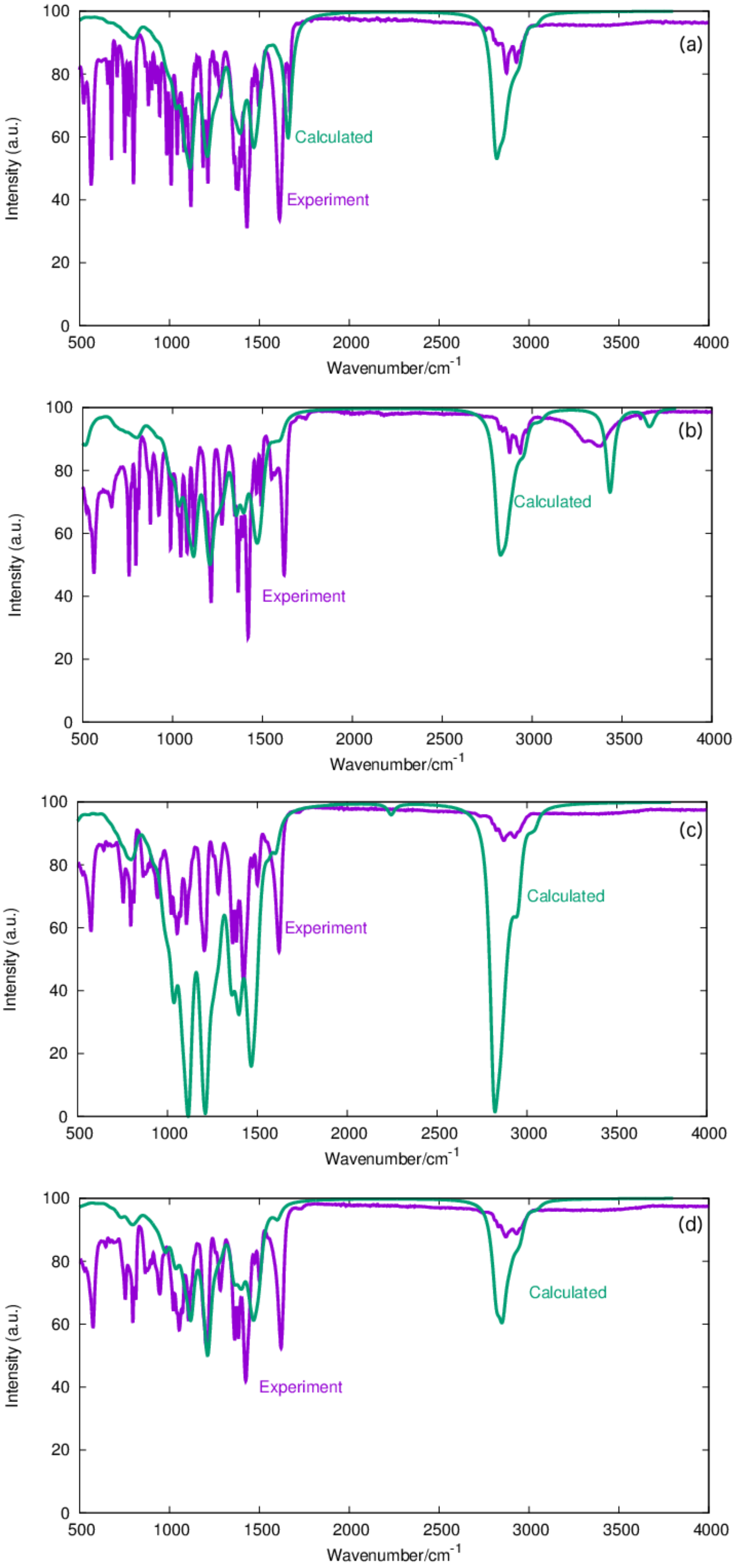

FIG. S57. Comparison of calculated (green) and experimental (purple) IR patterns for (a) $Cu_2(1^{**})_4(DMF)_2$ with Cu(**1**)(DMF) exfoliated in DMF, (b) $Cu_2(1^{**})_4(H_2O)_2$ with Cu(**1**)(DMF) exfoliated in H_2O, (c) $Cu_2(1^{**})_4(CH_3CN)_2$ with Cu(**1**)(DMF) exfoliated in CH_3CN [theoretical intensity *2] (d) $Cu_2(1^{**})_4$ with Cu(**1**)(DMF) exfoliated in CH_3CN where 1^{**} is 2,5-bis(3-methoxypropoxy)benzoate. All theoretical spectra are scaled, so that the maximum intensity is of a similar size to the experimental maximum intensity.

S6.1. Calculations on $Cu_2(1^{**})_4$

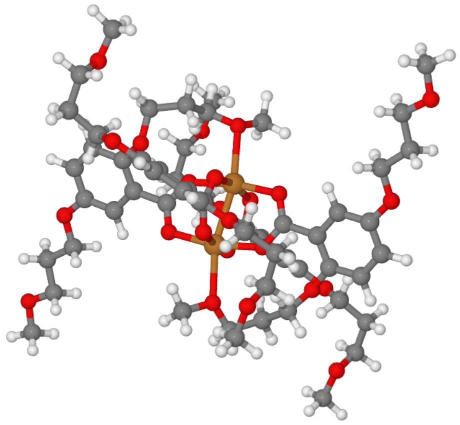

Route	: # opt freq b3lyp/genecp geom=connectivity int=ultrafine scf=noincfock	
SMILES	: COCCCOc1ccc2c(c1)[C]3O[Cu]456(O[C]7c8cc(ccc8OCCCO([Cu]4 (O7)(O3)(O[C](O5)c9cc(ccc9OCCCOC)OCCCOC)O[C](O6) c1cc(ccc1OCCCOC)OCCCOC)C)OCCCOC)O(CCCO2)C	
Formula	: $C_{60}H_{84}Cu_2O_{24}$	
Charge	: 0	
Multiplicity	: 1	
Dipole	: 3.9767	Debye
Energy	: -4538.13777176	a.u.
Gibbs Energy	: -4536.90345600	a.u.
Number of imaginary frequencies	: 0	

S6.1.1. Cartesian Co-ordinates (XYZ format)

170

O	-0.59924698	2.16826391	-0.22277699
O	0.01185300	1.36725700	1.80174601
O	-2.03033900	-0.12865099	-1.18730104
O	-1.72280300	-0.67645597	0.97815102
C	-0.35410100	2.27482605	1.01107597
C	-2.40382910	-0.60022300	-0.08039200
O	-2.48224401	3.15643597	2.60664105
O	-2.97480893	-3.14117908	0.96999300
O	0.02175700	-1.59941196	3.30314898
C	-1.50255001	4.08171988	2.37500310
O	-4.02531719	1.80512404	5.62724018
C	-4.04954815	-2.35432410	0.65986198
C	0.60299200	4.55590010	1.25908995
H	1.40827501	4.20720816	0.62640703
C	-0.43229601	3.67581010	1.57263303
C	-4.87523317	-0.35821199	-0.45454901
H	-4.64608192	0.56823301	-0.96184301
C	-3.81101203	-1.12247705	0.03383000
C	-3.66989708	3.56968999	3.28625011

H	-4.15007687	4.38138294	2.72578597
H	-3.42611003	3.93059397	4.29080486
C	-2.84568095	-3.74750304	2.27298188
H	-3.01097393	-4.82338715	2.16169095
H	-3.61706805	-3.35003090	2.94187498
C	-1.43374503	-3.47523308	2.79856610
H	-1.12770998	-4.29790688	3.45268011
H	-0.75349897	-3.47819996	1.94459295
C	-4.59352112	2.36508393	3.38091207
H	-4.74371576	1.94467199	2.38226008
H	-5.56685305	2.70328903	3.75093007
C	-1.28893495	-2.14097095	3.53807807
H	-2.03432703	-1.42054701	3.19134998
H	-1.41744697	-2.28521609	4.61882401
C	-4.06967306	1.27832496	4.30809689
H	-4.73272800	0.40073499	4.27391005
H	-3.06845808	0.95980197	3.98797202
C	0.43514299	-0.66961199	4.30345201
H	-0.21996599	0.20758601	4.33018780
H	1.44182503	-0.34793401	4.03944016
H	0.45146900	-1.15173101	5.28876019
C	-3.48511505	0.90139502	6.56686878
H	-4.08299398	-0.01991900	6.63308811
H	-3.49496388	1.39764798	7.53807688
H	-2.45016599	0.62696201	6.31716299
O	1.56205404	6.78938723	1.51450300
C	0.59150600	5.85975695	1.76022899
O	2.66589403	8.31341076	-1.57902205
C	-1.51184201	5.39193916	2.86515999
H	-2.32543302	5.73826694	3.48778605
C	-0.47413900	6.26705408	2.56408000
C	2.67687607	6.41708708	0.69637799
H	3.23398209	5.60387802	1.17541301
H	2.33033204	6.07053709	-0.28158101
C	3.55741191	7.64627790	0.53398699
H	3.83934999	8.02117729	1.52294397
H	4.47593880	7.34820604	0.01872200
C	2.89260697	8.76702309	-0.25250000
H	3.54010892	9.65733528	-0.26460201
H	1.94139194	9.04724121	0.22103401
C	1.97241294	9.25402641	-2.37084198
H	2.53454208	10.19467068	-2.46887302
H	1.84592199	8.81516171	-3.36132407
H	0.98157901	9.48367310	-1.95307004
O	-7.29732895	-0.10443600	-0.67979997
O	-9.78078461	3.36888194	-2.76915503
C	-6.18940210	-0.78561801	-0.26341099
C	-5.37016678	-2.78041506	0.83407700
H	-5.56413221	-3.74063110	1.29780400
C	-6.42813683	-1.99777997	0.39464900
C	-7.12290287	1.12537003	-1.38164306
H	-6.58336878	1.84268606	-0.74913001
H	-6.52507401	0.95588797	-2.28676701
C	-8.50666332	1.64720798	-1.73395896
H	-9.08675098	1.78397703	-0.81763500
H	-9.02959919	0.90513903	-2.34273410
C	-8.45309258	2.96622300	-2.49164510
H	-7.88686895	2.85157204	-3.43062806
H	-7.93977594	3.73702598	-1.89341104
C	-9.84974575	4.59005594	-3.47589898
H	-9.34536839	4.52352905	-4.45104218
H	-10.90529537	4.80966187	-3.63817406
H	-9.39741707	5.41484118	-2.90594101
O	0.37799200	-1.30312598	-1.94487202

O	1.78901303	0.92212999	-0.86243099
O	0.45929801	-2.21971893	0.11867700
O	2.23392797	-0.16508000	1.05600798
C	0.47430599	-2.26840591	-1.14323294
C	2.55471611	0.55084598	0.06959800
O	2.63072300	-3.19520593	-2.69433188
O	3.43856001	3.04606795	-1.28729296
O	0.27156401	1.45630002	-3.42206502
C	1.61120296	-4.08371782	-2.51504898
O	3.99995494	-1.66157997	-5.68023586
C	4.35044384	2.16824198	-0.75063598
C	-0.53621399	-4.50904417	-1.45941806
H	-1.33696604	-4.14257193	-0.83165801
C	0.53392798	-3.65692210	-1.73023701
C	4.96780014	0.23350200	0.58748400
H	4.63861704	-0.63408101	1.14030695
C	3.98283005	1.00669301	-0.04486200
C	3.77218008	-3.59118009	-3.45709610
H	4.26543713	-4.44226503	-2.97117591
H	3.46761203	-3.88729692	-4.46624517
C	2.92205501	2.75660491	-2.60748506
H	3.61695790	3.16877604	-3.35038209
H	2.84735799	1.67938900	-2.74173808
C	1.54052901	3.39897704	-2.71898603
H	1.63631701	4.47052908	-2.92235589
H	1.03270495	3.29647899	-1.76007199
C	4.71159506	-2.39699292	-3.52722192
H	4.93828106	-2.05549502	-2.51291609
H	5.65073776	-2.72052407	-3.98763895
C	0.65619701	2.78379297	-3.79440308
H	1.17779803	2.73888111	-4.76273584
H	-0.24430200	3.39783502	-3.93118906
C	4.14425087	-1.23604095	-4.33112192
H	4.82507992	-0.37309799	-4.27908611
H	3.17291403	-0.92989701	-3.92008805
C	-0.47164199	0.79082102	-4.43711185
H	0.09790800	0.75870901	-5.37479687
H	-0.64869398	-0.22494200	-4.08861399
H	-1.43018198	1.29356503	-4.61912680
C	3.40282989	-0.68554002	-6.50552702
H	4.00776005	0.23255000	-6.55065584
H	3.32934403	-1.10794103	-7.50855112
H	2.39523792	-0.42107600	-6.15397596
O	-1.56694603	-6.70220709	-1.77761400
C	-0.55942100	-5.79973412	-1.99047804
O	-3.11987090	-7.73808193	1.38408601
C	1.58840203	-5.38510799	-3.02890205
H	2.40693402	-5.74847221	-3.63512301
C	0.51156098	-6.22838211	-2.77511597
C	-2.76839709	-6.24054384	-1.15132594
H	-3.25647902	-5.50121403	-1.79730201
H	-2.54344106	-5.76488400	-0.19381000
C	-3.66689301	-7.44629812	-0.92644298
H	-3.79355407	-7.98440695	-1.87094295
H	-4.65422297	-7.09258318	-0.61286801
C	-3.13003302	-8.40433121	0.12706999
H	-3.76492000	-9.30129623	0.18525299
H	-2.11330700	-8.72522736	-0.13856100
C	-2.55744505	-8.52388382	2.41487908
H	-3.12588310	-9.45230579	2.57098103
H	-2.59096289	-7.93316412	3.33116007
H	-1.51260805	-8.78737831	2.19838095
O	7.33493185	-0.11263500	1.07915497
O	9.41343594	-3.57039309	3.59462404

C	6.31246090	0.58565098	0.50628799
C	5.70448399	2.51618910	-0.80660999
H	5.97441578	3.42591906	-1.32991004
C	6.67640686	1.73960400	-0.19817901
C	7.02712107	-1.28756595	1.82941401
H	6.51091003	-2.01396108	1.18831801
H	6.35625505	-1.03311503	2.66010189
C	8.34050274	-1.85399699	2.34432292
H	8.99675274	-2.07465005	1.49842203
H	8.84321213	-1.10311103	2.95935202
C	8.14236641	-3.12033606	3.16520000
H	7.49603510	-2.92126703	4.03579283
H	7.64716911	-3.89971900	2.56298304
C	9.34684372	-4.74855185	4.37097406
H	8.75532436	-4.59864092	5.28607416
H	10.36821270	-5.00980711	4.64914179
H	8.90674973	-5.58294582	3.80540991
H	-7.45392418	-2.31942391	0.52769899
H	0.49123701	-7.23277187	-3.18058705
H	7.72422123	2.01070309	-0.24507500
H	-0.47873700	7.28008890	2.94769597
Cu	0.25230101	-0.52656299	1.15964496
Cu	-0.11326900	0.50752002	-1.22159195

S6.1.2. Frequencies

Mode	IR frequency	IR intensity	Raman intensity
1	4.61940000	0.02160000	0.00000000
2	6.66000000	0.04460000	0.00000000
3	7.78030000	0.00530000	0.00000000
4	8.01610000	0.01230000	0.00000000
5	8.91440000	0.01950000	0.00000000
6	10.98540000	0.08830000	0.00000000
7	12.49820000	0.20410000	0.00000000
8	13.17630000	0.13700000	0.00000000
9	14.68650000	0.04910000	0.00000000
10	16.94880000	0.12320000	0.00000000
11	17.77660000	0.57590000	0.00000000
12	18.81560000	0.12170000	0.00000000
13	20.11440000	0.00760000	0.00000000
14	23.16470000	0.49600000	0.00000000
15	25.00770000	0.00700000	0.00000000
16	25.99810000	0.01730000	0.00000000
17	26.55780000	0.05020000	0.00000000
18	27.09730000	0.08410000	0.00000000
19	33.61090000	0.22020000	0.00000000
20	37.60530000	0.14440000	0.00000000
21	39.93920000	0.72920000	0.00000000
22	41.23080000	0.04030000	0.00000000
23	42.90570000	0.50420000	0.00000000
24	46.58710000	0.98390000	0.00000000
25	49.49200000	0.37720000	0.00000000
26	49.83250000	5.93740000	0.00000000
27	50.63010000	2.65690000	0.00000000
28	54.63240000	0.29210000	0.00000000
29	57.44960000	0.05080000	0.00000000
30	61.10090000	0.79120000	0.00000000
31	63.97230000	0.09880000	0.00000000
32	66.01560000	0.40040000	0.00000000
33	68.77090000	0.11200000	0.00000000
34	71.91550000	0.21420000	0.00000000
35	74.35260000	3.97650000	0.00000000
36	77.96960000	0.31630000	0.00000000
37	80.98110000	0.28020000	0.00000000
38	81.47320000	0.26880000	0.00000000
39	82.41350000	0.71000000	0.00000000
40	85.27160000	0.45140000	0.00000000
41	87.02170000	2.64780000	0.00000000
42	92.46260000	0.03290000	0.00000000
43	93.76970000	0.93890000	0.00000000
44	94.59170000	0.46810000	0.00000000
45	95.17650000	1.32940000	0.00000000
46	97.11510000	0.98810000	0.00000000
47	98.69940000	1.37910000	0.00000000
48	99.53150000	13.16350000	0.00000000
49	106.29860000	0.16420000	0.00000000
50	106.96210000	0.18520000	0.00000000
51	110.45760000	0.73830000	0.00000000
52	115.84740000	0.53000000	0.00000000
53	120.08000000	10.56550000	0.00000000
54	124.01210000	3.45760000	0.00000000
55	128.27950000	3.12080000	0.00000000
56	129.05040000	1.07630000	0.00000000
57	130.29240000	0.55780000	0.00000000
58	133.13560000	0.48190000	0.00000000
59	136.30380000	0.54270000	0.00000000
60	138.31240000	0.96080000	0.00000000

61	139.97860000	3.76910000	0.00000000
62	143.02400000	0.16050000	0.00000000
63	144.28780000	0.77640000	0.00000000
64	150.60900000	6.60930000	0.00000000
65	153.17390000	4.60230000	0.00000000
66	157.37830000	1.10920000	0.00000000
67	160.09240000	0.00190000	0.00000000
68	164.28800000	0.17570000	0.00000000
69	169.94190000	1.67940000	0.00000000
70	174.79870000	2.94450000	0.00000000
71	177.45950000	5.02630000	0.00000000
72	180.60080000	1.57730000	0.00000000
73	184.74560000	0.06790000	0.00000000
74	186.24260000	1.80720000	0.00000000
75	191.10540000	1.48500000	0.00000000
76	194.77990000	2.88970000	0.00000000
77	197.64510000	2.79380000	0.00000000
78	200.55540000	0.04590000	0.00000000
79	200.99620000	0.99500000	0.00000000
80	203.69390000	0.41840000	0.00000000
81	205.75560000	1.22490000	0.00000000
82	208.94160000	2.08600000	0.00000000
83	212.76540000	4.56130000	0.00000000
84	216.03770000	1.75470000	0.00000000
85	218.57740000	0.07580000	0.00000000
86	223.82460000	5.88630000	0.00000000
87	225.99030000	2.54770000	0.00000000
88	228.03940000	4.80990000	0.00000000
89	230.30630000	2.11760000	0.00000000
90	230.57440000	0.90190000	0.00000000
91	231.80070000	2.80930000	0.00000000
92	235.39440000	2.28390000	0.00000000
93	238.07230000	1.29090000	0.00000000
94	238.48040000	6.60260000	0.00000000
95	243.50620000	0.37060000	0.00000000
96	245.13160000	12.06450000	0.00000000
97	248.44000000	13.64970000	0.00000000
98	249.83220000	1.20430000	0.00000000
99	256.76800000	4.57050000	0.00000000
100	261.23600000	23.00720000	0.00000000
101	267.70330000	5.39740000	0.00000000
102	270.58660000	1.81760000	0.00000000
103	275.80050000	10.78060000	0.00000000
104	277.79200000	2.01650000	0.00000000
105	282.51100000	12.50810000	0.00000000
106	291.75960000	12.45730000	0.00000000
107	307.59180000	0.09720000	0.00000000
108	308.45730000	0.90500000	0.00000000
109	313.54870000	8.47620000	0.00000000
110	320.80530000	3.49700000	0.00000000
111	321.71650000	1.13220000	0.00000000
112	323.29270000	2.68740000	0.00000000
113	333.50890000	4.22210000	0.00000000
114	337.92880000	2.39820000	0.00000000
115	341.79560000	20.08380000	0.00000000
116	362.71210000	0.85810000	0.00000000
117	370.06900000	0.14680000	0.00000000
118	376.93410000	0.18220000	0.00000000
119	382.13330000	0.18280000	0.00000000
120	383.92800000	0.17490000	0.00000000
121	393.47780000	1.93940000	0.00000000
122	395.19550000	14.35890000	0.00000000
123	399.43760000	0.36050000	0.00000000
124	413.89350000	0.13320000	0.00000000

125	417.48760000	0.48890000	0.00000000
126	419.12910000	2.84250000	0.00000000
127	425.43720000	6.23770000	0.00000000
128	431.96960000	0.01580000	0.00000000
129	436.10790000	4.70340000	0.00000000
130	440.99170000	1.38460000	0.00000000
131	446.76660000	0.15000000	0.00000000
132	446.92270000	4.98780000	0.00000000
133	450.34370000	0.31250000	0.00000000
134	454.55010000	2.19160000	0.00000000
135	466.47390000	18.00460000	0.00000000
136	470.57830000	0.66590000	0.00000000
137	477.88950000	0.41580000	0.00000000
138	481.62510000	0.59220000	0.00000000
139	485.91630000	6.24290000	0.00000000
140	486.37860000	19.41270000	0.00000000
141	499.29320000	47.25970000	0.00000000
142	508.35900000	1.12940000	0.00000000
143	514.30610000	2.52400000	0.00000000
144	519.52850000	12.55160000	0.00000000
145	524.11810000	1.14720000	0.00000000
146	528.42680000	5.66850000	0.00000000
147	533.07270000	0.73000000	0.00000000
148	533.20240000	0.02920000	0.00000000
149	540.49590000	2.58170000	0.00000000
150	543.95140000	5.54300000	0.00000000
151	545.40150000	13.28220000	0.00000000
152	566.85240000	6.10740000	0.00000000
153	605.43850000	6.08160000	0.00000000
154	610.26000000	7.06790000	0.00000000
155	618.71150000	6.89510000	0.00000000
156	620.45190000	0.54930000	0.00000000
157	620.71540000	1.06210000	0.00000000
158	637.59540000	1.65200000	0.00000000
159	640.91120000	1.50360000	0.00000000
160	641.41750000	0.21510000	0.00000000
161	686.88870000	10.78450000	0.00000000
162	687.47330000	8.33250000	0.00000000
163	690.03810000	12.83830000	0.00000000
164	696.01060000	5.94020000	0.00000000
165	712.09580000	4.89940000	0.00000000
166	720.74110000	5.05470000	0.00000000
167	734.91980000	25.45620000	0.00000000
168	740.69530000	37.72060000	0.00000000
169	751.09940000	40.73590000	0.00000000
170	754.02560000	14.30970000	0.00000000
171	756.39790000	32.72810000	0.00000000
172	757.66670000	3.97180000	0.00000000
173	786.57670000	3.43260000	0.00000000
174	788.02460000	4.94270000	0.00000000
175	791.74150000	8.46800000	0.00000000
176	793.33450000	17.58430000	0.00000000
177	795.99010000	0.90740000	0.00000000
178	796.88850000	41.41900000	0.00000000
179	799.47230000	6.58410000	0.00000000
180	801.87460000	7.05580000	0.00000000
181	811.52340000	51.99690000	0.00000000
182	815.82720000	21.92080000	0.00000000
183	820.74160000	29.41020000	0.00000000
184	821.86060000	28.05100000	0.00000000
185	826.74420000	11.39940000	0.00000000
186	828.01790000	0.83600000	0.00000000
187	829.76560000	24.72510000	0.00000000
188	837.04320000	2.39620000	0.00000000

189	840.92730000	37.36000000	0.00000000
190	843.68420000	11.00000000	0.00000000
191	845.30880000	1.04880000	0.00000000
192	854.80780000	29.34880000	0.00000000
193	881.39460000	12.24330000	0.00000000
194	890.54260000	9.02720000	0.00000000
195	893.79280000	1.74610000	0.00000000
196	897.65720000	9.40930000	0.00000000
197	899.02890000	0.87510000	0.00000000
198	899.70430000	3.10410000	0.00000000
199	901.08970000	2.57350000	0.00000000
200	901.70480000	1.73900000	0.00000000
201	904.83760000	8.73830000	0.00000000
202	905.53910000	1.20020000	0.00000000
203	907.71550000	1.80960000	0.00000000
204	908.07780000	10.58280000	0.00000000
205	917.82820000	34.86180000	0.00000000
206	933.11750000	3.63880000	0.00000000
207	934.50460000	18.30290000	0.00000000
208	935.43060000	16.87450000	0.00000000
209	940.85030000	10.24910000	0.00000000
210	946.17960000	7.82180000	0.00000000
211	947.53670000	15.77450000	0.00000000
212	947.67080000	14.11670000	0.00000000
213	949.76380000	6.58050000	0.00000000
214	950.10550000	13.49220000	0.00000000
215	951.30440000	1.48570000	0.00000000
216	954.76250000	11.21630000	0.00000000
217	956.58300000	5.78460000	0.00000000
218	965.05540000	3.38930000	0.00000000
219	973.23090000	58.41150000	0.00000000
220	973.66740000	22.22350000	0.00000000
221	986.10930000	79.54050000	0.00000000
222	997.46780000	24.00860000	0.00000000
223	998.51450000	26.49840000	0.00000000
224	1003.82840000	107.87690000	0.00000000
225	1008.15950000	160.54050000	0.00000000
226	1008.62500000	34.73300000	0.00000000
227	1049.63520000	5.17790000	0.00000000
228	1052.30970000	60.48430000	0.00000000
229	1052.74200000	123.83860000	0.00000000
230	1057.91860000	59.22320000	0.00000000
231	1063.63280000	10.06540000	0.00000000
232	1064.54870000	1.21360000	0.00000000
233	1066.50920000	44.12130000	0.00000000
234	1066.85500000	91.74730000	0.00000000
235	1067.01360000	56.02890000	0.00000000
236	1067.62150000	94.39680000	0.00000000
237	1092.90490000	45.04320000	0.00000000
238	1099.11930000	2.14010000	0.00000000
239	1099.92250000	1.27500000	0.00000000
240	1103.34730000	20.44540000	0.00000000
241	1103.89170000	28.73420000	0.00000000
242	1105.62560000	35.11500000	0.00000000
243	1106.56320000	9.64540000	0.00000000
244	1107.51790000	43.86710000	0.00000000
245	1111.99360000	6.26700000	0.00000000
246	1114.46570000	41.52150000	0.00000000
247	1116.19410000	67.93340000	0.00000000
248	1116.41640000	11.81930000	0.00000000
249	1117.03760000	9.51970000	0.00000000
250	1117.59510000	13.40410000	0.00000000
251	1117.95040000	8.63590000	0.00000000
252	1118.38270000	17.85140000	0.00000000

253	1118.90920000	41.00750000	0.00000000
254	1123.34480000	116.80480000	0.00000000
255	1125.06490000	15.71170000	0.00000000
256	1131.54880000	64.21180000	0.00000000
257	1146.55500000	120.15400000	0.00000000
258	1150.09190000	50.25670000	0.00000000
259	1150.45010000	369.84940000	0.00000000
260	1150.47750000	7.49190000	0.00000000
261	1152.25010000	289.54040000	0.00000000
262	1152.73240000	155.97760000	0.00000000
263	1157.43720000	2.10120000	0.00000000
264	1161.16850000	4.45570000	0.00000000
265	1163.40010000	0.36220000	0.00000000
266	1163.59160000	0.35390000	0.00000000
267	1170.49340000	8.34700000	0.00000000
268	1170.73330000	2.93630000	0.00000000
269	1178.34340000	3.79360000	0.00000000
270	1178.36470000	3.06740000	0.00000000
271	1178.64550000	5.61200000	0.00000000
272	1178.82610000	1.24270000	0.00000000
273	1178.86560000	2.61320000	0.00000000
274	1178.87440000	4.13910000	0.00000000
275	1186.25050000	7.40830000	0.00000000
276	1186.42350000	8.11580000	0.00000000
277	1209.21320000	26.30200000	0.00000000
278	1211.66910000	24.34740000	0.00000000
279	1212.79430000	37.24720000	0.00000000
280	1213.27350000	17.23390000	0.00000000
281	1213.51820000	12.46760000	0.00000000
282	1213.74430000	23.70540000	0.00000000
283	1223.08120000	74.79290000	0.00000000
284	1223.10770000	0.37410000	0.00000000
285	1235.09020000	198.95880000	0.00000000
286	1237.23680000	1.10330000	0.00000000
287	1237.27860000	1.50340000	0.00000000
288	1241.24940000	365.46500000	0.00000000
289	1247.46520000	270.42860000	0.00000000
290	1249.02170000	571.87290000	0.00000000
291	1253.27620000	65.56510000	0.00000000
292	1253.78380000	48.84620000	0.00000000
293	1255.54460000	5.61870000	0.00000000
294	1256.38700000	2.52360000	0.00000000
295	1256.68910000	60.96040000	0.00000000
296	1259.08540000	10.18690000	0.00000000
297	1262.72830000	2.17710000	0.00000000
298	1263.24480000	5.30840000	0.00000000
299	1263.82340000	12.60400000	0.00000000
300	1264.23360000	10.98640000	0.00000000
301	1264.66280000	15.56870000	0.00000000
302	1273.98870000	2.78020000	0.00000000
303	1281.69280000	4.65350000	0.00000000
304	1291.28930000	100.15610000	0.00000000
305	1293.33700000	63.94650000	0.00000000
306	1293.97720000	17.91910000	0.00000000
307	1294.91180000	7.51730000	0.00000000
308	1299.78000000	11.87630000	0.00000000
309	1307.22380000	14.43990000	0.00000000
310	1308.42540000	17.84410000	0.00000000
311	1309.33270000	0.13340000	0.00000000
312	1309.44800000	1.11900000	0.00000000
313	1309.54760000	37.68450000	0.00000000
314	1309.87600000	2.64160000	0.00000000
315	1315.27970000	0.34580000	0.00000000
316	1315.61260000	0.37540000	0.00000000

317	1317.53550000	4.95650000	0.00000000
318	1317.83720000	51.49760000	0.00000000
319	1324.16980000	9.02170000	0.00000000
320	1325.87260000	59.51680000	0.00000000
321	1326.08570000	5.12730000	0.00000000
322	1326.91980000	30.74550000	0.00000000
323	1329.08480000	32.35650000	0.00000000
324	1329.45390000	31.63800000	0.00000000
325	1329.50030000	15.35000000	0.00000000
326	1329.81710000	3.41490000	0.00000000
327	1361.17970000	4.25540000	0.00000000
328	1367.17220000	2.11010000	0.00000000
329	1385.60290000	10.35070000	0.00000000
330	1387.16840000	16.82900000	0.00000000
331	1387.46110000	20.61790000	0.00000000
332	1388.71630000	15.21270000	0.00000000
333	1389.96380000	18.95260000	0.00000000
334	1394.64610000	334.33110000	0.00000000
335	1399.07160000	158.17760000	0.00000000
336	1402.79930000	3.49380000	0.00000000
337	1413.38090000	63.13350000	0.00000000
338	1415.04610000	1.87710000	0.00000000
339	1415.08770000	18.64560000	0.00000000
340	1416.50080000	11.93250000	0.00000000
341	1418.37460000	8.47490000	0.00000000
342	1419.15980000	5.72290000	0.00000000
343	1424.71560000	49.82630000	0.00000000
344	1425.90980000	0.52500000	0.00000000
345	1427.08150000	29.20890000	0.00000000
346	1428.17350000	37.49820000	0.00000000
347	1428.75450000	17.69900000	0.00000000
348	1431.57170000	46.87860000	0.00000000
349	1433.39580000	27.22570000	0.00000000
350	1434.03310000	28.77810000	0.00000000
351	1440.69510000	134.21140000	0.00000000
352	1441.28190000	47.59170000	0.00000000
353	1445.08490000	59.97440000	0.00000000
354	1445.63530000	46.18590000	0.00000000
355	1446.63310000	61.57840000	0.00000000
356	1451.04250000	46.46330000	0.00000000
357	1463.16040000	9.81350000	0.00000000
358	1463.32340000	6.81240000	0.00000000
359	1463.68710000	11.09680000	0.00000000
360	1463.87530000	5.62960000	0.00000000
361	1475.66600000	18.23220000	0.00000000
362	1477.22910000	32.12690000	0.00000000
363	1477.55330000	3.94970000	0.00000000
364	1478.54400000	2.94120000	0.00000000
365	1479.09670000	5.52200000	0.00000000
366	1479.99980000	6.82580000	0.00000000
367	1480.79460000	0.17170000	0.00000000
368	1480.81260000	0.18050000	0.00000000
369	1482.08470000	44.81240000	0.00000000
370	1482.65860000	9.94400000	0.00000000
371	1482.86950000	5.51670000	0.00000000
372	1483.27010000	5.13130000	0.00000000
373	1483.34410000	2.56850000	0.00000000
374	1483.74650000	7.40210000	0.00000000
375	1483.91000000	7.28510000	0.00000000
376	1485.03170000	100.73190000	0.00000000
377	1490.99470000	92.14460000	0.00000000
378	1491.54940000	1.35110000	0.00000000
379	1491.71280000	8.17010000	0.00000000
380	1495.56180000	13.29180000	0.00000000

381	1500.37710000	20.62840000	0.00000000
382	1501.09690000	92.28000000	0.00000000
383	1502.20860000	3.58800000	0.00000000
384	1502.37400000	26.20740000	0.00000000
385	1502.99540000	28.01310000	0.00000000
386	1503.46350000	6.95200000	0.00000000
387	1503.80180000	33.95850000	0.00000000
388	1504.01310000	1.97630000	0.00000000
389	1505.03240000	124.32850000	0.00000000
390	1506.47670000	8.33110000	0.00000000
391	1507.35440000	48.95360000	0.00000000
392	1509.15460000	161.82680000	0.00000000
393	1509.91250000	127.61070000	0.00000000
394	1515.45170000	9.14360000	0.00000000
395	1515.87110000	47.98690000	0.00000000
396	1516.72190000	8.64580000	0.00000000
397	1517.74950000	65.01080000	0.00000000
398	1520.86380000	18.56540000	0.00000000
399	1522.81270000	2.77150000	0.00000000
400	1524.30370000	36.68370000	0.00000000
401	1527.37800000	9.82000000	0.00000000
402	1527.74510000	10.29480000	0.00000000
403	1528.04840000	12.58720000	0.00000000
404	1528.32560000	8.27000000	0.00000000
405	1529.47540000	8.26320000	0.00000000
406	1532.23960000	175.78470000	0.00000000
407	1535.95320000	1.18020000	0.00000000
408	1537.21710000	88.75840000	0.00000000
409	1539.45700000	6.70530000	0.00000000
410	1540.63770000	287.02500000	0.00000000
411	1545.57190000	11.82020000	0.00000000
412	1562.51450000	6.94280000	0.00000000
413	1604.48560000	1.04700000	0.00000000
414	1611.62150000	0.21040000	0.00000000
415	1622.33330000	8.64180000	0.00000000
416	1623.07990000	7.12900000	0.00000000
417	1645.54390000	89.09280000	0.00000000
418	1646.39780000	1.63550000	0.00000000
419	1649.76440000	55.76230000	0.00000000
420	1650.62890000	0.56910000	0.00000000
421	2938.50910000	43.04330000	0.00000000
422	2939.37020000	41.60010000	0.00000000
423	2957.20370000	30.24090000	0.00000000
424	2958.78570000	28.21750000	0.00000000
425	2959.03800000	34.08060000	0.00000000
426	2962.00750000	26.89930000	0.00000000
427	2962.31040000	53.65640000	0.00000000
428	2963.38800000	53.40190000	0.00000000
429	2964.34290000	72.89030000	0.00000000
430	2964.41500000	9.99350000	0.00000000
431	2964.50680000	101.67450000	0.00000000
432	2967.45610000	155.06290000	0.00000000
433	2970.36540000	54.63130000	0.00000000
434	2970.56470000	103.40180000	0.00000000
435	2971.85380000	129.38940000	0.00000000
436	2985.23230000	28.60900000	0.00000000
437	2985.56750000	96.40880000	0.00000000
438	2988.22660000	28.64490000	0.00000000
439	2989.74580000	9.43200000	0.00000000
440	2998.18090000	148.10210000	0.00000000
441	2998.89980000	41.26750000	0.00000000
442	2999.65910000	46.86370000	0.00000000
443	3000.83720000	43.20590000	0.00000000
444	3002.35270000	80.27320000	0.00000000

445	3003.85840000	106.89300000	0.00000000
446	3004.22840000	63.27550000	0.00000000
447	3004.43730000	63.50710000	0.00000000
448	3004.70940000	38.59360000	0.00000000
449	3004.74820000	64.06900000	0.00000000
450	3005.41340000	22.92570000	0.00000000
451	3005.62020000	65.39870000	0.00000000
452	3011.12820000	40.03320000	0.00000000
453	3011.86610000	13.44350000	0.00000000
454	3013.97460000	20.16820000	0.00000000
455	3014.17270000	11.55780000	0.00000000
456	3015.58610000	24.80940000	0.00000000
457	3015.82320000	32.29830000	0.00000000
458	3020.17800000	30.43630000	0.00000000
459	3023.57080000	27.05480000	0.00000000
460	3034.74090000	49.73880000	0.00000000
461	3038.11180000	40.80860000	0.00000000
462	3038.91050000	33.37210000	0.00000000
463	3039.56840000	23.84500000	0.00000000
464	3039.68800000	30.58770000	0.00000000
465	3040.12170000	32.51970000	0.00000000
466	3044.88400000	37.56680000	0.00000000
467	3050.50500000	20.33570000	0.00000000
468	3050.85340000	19.28970000	0.00000000
469	3054.36960000	28.47450000	0.00000000
470	3054.47290000	32.29040000	0.00000000
471	3058.28450000	35.72790000	0.00000000
472	3062.56070000	18.96100000	0.00000000
473	3067.13700000	18.02940000	0.00000000
474	3072.54860000	38.73780000	0.00000000
475	3073.21360000	12.38430000	0.00000000
476	3082.24810000	32.38600000	0.00000000
477	3083.62090000	36.51760000	0.00000000
478	3084.18080000	34.02050000	0.00000000
479	3086.14620000	35.38470000	0.00000000
480	3095.14490000	29.77710000	0.00000000
481	3095.43470000	30.93740000	0.00000000
482	3101.64440000	5.31410000	0.00000000
483	3105.16280000	41.43920000	0.00000000
484	3106.38160000	27.08220000	0.00000000
485	3106.66520000	35.67790000	0.00000000
486	3106.84170000	33.61680000	0.00000000
487	3111.60910000	38.79450000	0.00000000
488	3112.16190000	37.12460000	0.00000000
489	3117.72290000	3.06530000	0.00000000
490	3126.30670000	11.22160000	0.00000000
491	3130.09460000	12.57980000	0.00000000
492	3142.23940000	3.58070000	0.00000000
493	3177.74390000	7.72840000	0.00000000
494	3180.25730000	6.18440000	0.00000000
495	3184.79260000	9.09940000	0.00000000
496	3185.50100000	9.11460000	0.00000000
497	3194.72490000	6.24080000	0.00000000
498	3195.66930000	7.99830000	0.00000000
499	3202.04520000	3.14920000	0.00000000
500	3206.58120000	2.90280000	0.00000000
501	3207.80720000	12.39560000	0.00000000
502	3207.87080000	11.65010000	0.00000000
503	3215.84630000	5.81230000	0.00000000
504	3225.21300000	8.43470000	0.00000000

S6.2. Calculations on $Cu_2(1^{**})_4(H_2O)_2$

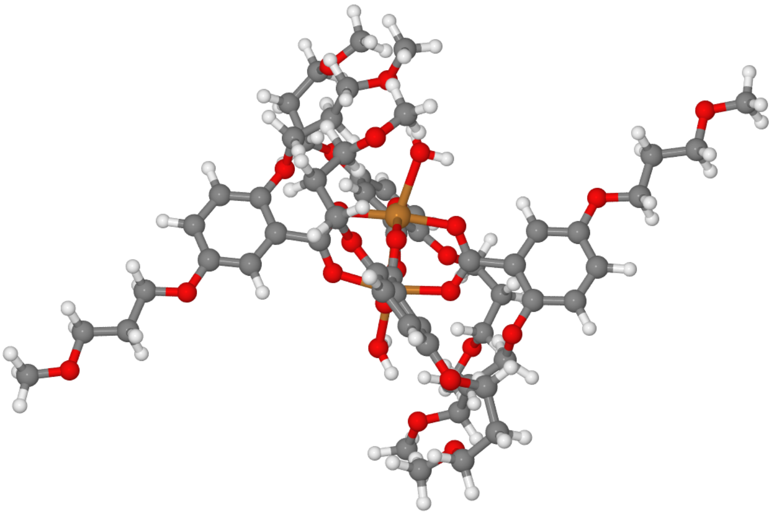

Route	: # opt freq b3lyp/genecp geom=connectivity int=ultrafine scf=noincfock	
SMILES	: COCCCOc1ccc(c(c1)[C]2O[Cu]345(O[C](O[Cu]3(O2)(O[C](O4)c6cc (ccc6OCCCOC)OCCCOC)(O[C](O5)c7cc(ccc7OCCCOC)OCCCOC)[OH2]) c8cc(ccc8OCCCOC)OCCCOC)[OH2])OCCCOC	
Formula	: $C_{60}H_{88}Cu_2O_{26}$	
Charge	: 0	
Multiplicity	: 1	
Dipole	: 9.0051	Debye
Energy	: -4691.07806650	a.u.
Gibbs Energy	: -4689.79802800	a.u.
Number of imaginary frequencies	: 0	

S6.2.1. Cartesian Co-ordinates (XYZ format)

176

O	0.16897701	-2.07245398	0.38908899
O	0.02110100	-0.72111601	2.19008398
O	2.01574612	-0.23166899	-0.80014801
O	1.67521405	1.21607995	0.88993597
C	0.12458200	-1.84515202	1.63182402
C	2.39869595	0.62766302	0.04622700
O	1.93039799	-2.25919199	3.79445291
O	3.57893491	3.25510597	0.77553999
O	0.27820900	4.33277321	4.02568007
C	1.02544999	-3.25502300	3.56304312
O	2.63983297	-0.30963001	6.78703594
C	4.38869476	2.21662092	0.38054001
C	-0.80253500	-4.06994200	2.18586898
H	-1.46757305	-3.89073706	1.35362804
C	0.12874500	-3.07765794	2.50007606
C	4.75372791	-0.03322700	-0.42271799
H	4.36947107	-0.99962002	-0.71803200
C	3.87380099	0.95210397	0.02248800
C	2.94017911	-2.45961404	4.78387880

H	3.57565594	-3.30735111	4.49919701
H	2.48498607	-2.67074990	5.75697088
C	3.34069610	3.32200789	2.19182992
H	4.19947290	3.80757904	2.67707491
H	3.24311996	2.30723691	2.58719611
C	2.04977894	4.08860493	2.42187905
H	2.11275601	5.09351206	1.99870396
H	1.25931895	3.55406904	1.89341295
C	3.75601006	-1.17852497	4.86708021
H	4.12054491	-0.91769201	3.86930108
H	4.62710524	-1.35870397	5.50513506
C	1.69451797	4.15814495	3.90247393
H	1.98238397	3.23093605	4.42149019
H	2.20676899	4.99077511	4.40264797
C	2.96426296	-0.01029200	5.43650484
H	3.56188989	0.91330302	5.38968515
H	2.04950905	0.14331999	4.84734392
C	-0.14420900	4.56593704	5.36183405
H	0.13921200	3.73590708	6.02295589
H	-1.23012400	4.65579605	5.34481192
H	0.29034999	5.49357319	5.75399590
C	1.80750406	0.65765101	7.38709402
H	2.28682899	1.64820397	7.40959692
H	1.61998200	0.33548301	8.41199303
H	0.84508198	0.74672699	6.86179113
O	-1.71821702	-6.28783703	2.67331100
C	-0.86184698	-5.25084782	2.92513108
O	-1.69042897	-7.60471201	-0.61722201
C	0.96558899	-4.44684505	4.29490089
H	1.64968598	-4.62036419	5.11404514
C	0.03048200	-5.42697477	3.98373508
C	-2.72132492	-6.13481808	1.66123104
H	-3.55089211	-5.53885221	2.05822301
H	-2.31503606	-5.62261581	0.78976703
C	-3.18246293	-7.52463102	1.24839199
H	-3.52126002	-8.08180046	2.12759089
H	-4.03840494	-7.41756296	0.57422298
C	-2.08524299	-8.31634998	0.54765600
H	-2.44770694	-9.31955719	0.27652499
H	-1.22628999	-8.43642616	1.22163904
C	-0.52258903	-8.11876678	-1.22577405
H	-0.69209200	-9.11916256	-1.65034103
H	-0.24574301	-7.42873001	-2.02364707
H	0.30839801	-8.17872238	-0.50954098
O	6.88057280	-0.85831100	-0.93713599
O	10.80416393	-3.21209598	-2.22330189
C	6.12819099	0.19582000	-0.50355202
C	5.75645924	2.44535208	0.26861599
H	6.13024187	3.43191195	0.51671797
C	6.63338995	1.45239902	-0.16362999
C	8.28902245	-0.68646801	-1.07426202
H	8.49888611	0.12520900	-1.78369403
H	8.72814941	-0.41251299	-0.10547100
C	8.86079407	-2.00364590	-1.57401705
H	8.39078617	-2.26398897	-2.52581096
H	8.61731339	-2.79700303	-0.86270702
C	10.37020969	-1.94736803	-1.76161599
H	10.87051582	-1.69817305	-0.81108397
H	10.64204884	-1.16406798	-2.48844194
C	12.19963360	-3.27283096	-2.43442011
H	12.75896168	-3.08303690	-1.50656402
H	12.42914295	-4.27904081	-2.78586793
H	12.52716732	-2.54684091	-3.19294405
O	-0.12543100	1.04393804	-2.10760498

O	-1.88197601	-0.98081201	-1.13826001
O	-0.61118001	2.32119393	-0.30844200
O	-2.23534203	0.18961300	0.75812203
C	-0.39211500	2.13559008	-1.54094303
C	-2.59974504	-0.54541701	-0.19351999
O	-1.68735099	2.37643099	-4.07082987
O	-3.57738900	-3.26425290	-0.69667602
O	0.11466700	-4.68325281	-3.32697392
C	-0.96989000	3.45274901	-3.65726495
O	-0.68416500	0.02543100	-6.48958492
C	-4.48224211	-2.24436498	-0.49872801
C	0.31590801	4.48379517	-1.87387002
H	0.72706902	4.40396500	-0.87796998
C	-0.37150499	3.38345194	-2.38612103
C	-5.00225115	0.04379300	0.09263700
H	-4.68087912	1.04461002	0.34938899
C	-4.05868912	-0.93137699	-0.21454000
C	-2.14933491	2.32156992	-5.42165613
H	-2.91696000	3.08796191	-5.58759022
H	-1.31494105	2.49789190	-6.10749292
C	-3.20178103	-3.50621390	-2.06733298
H	-3.98576689	-4.10184383	-2.55450010
H	-3.10886502	-2.54890800	-2.58485389
C	-1.86292303	-4.22684479	-2.05464792
H	-1.95732999	-5.20458698	-1.57569206
H	-1.17624605	-3.62536097	-1.45571899
C	-2.72548199	0.93238699	-5.64882088
H	-3.53785300	0.75560600	-4.93713903
H	-3.15201092	0.89763898	-6.65697718
C	-1.28227997	-4.38807583	-3.45210505
H	-1.40026295	-3.46440005	-4.03651381
H	-1.77538800	-5.20079613	-4.00331116
C	-1.68697405	-0.17090900	-5.50157118
H	-2.16521001	-1.15409195	-5.64042902
H	-1.24407804	-0.14107201	-4.49917221
C	0.72823799	-4.97644711	-4.57424593
H	0.64350802	-4.13263798	-5.27049017
H	1.78217804	-5.17410803	-4.37690020
H	0.27725500	-5.86474991	-5.03445101
C	0.38791201	-0.88816601	-6.36830282
H	0.06678300	-1.91369700	-6.61224794
H	1.15469801	-0.58475900	-7.08282614
H	0.80812800	-0.88898802	-5.35503721
O	1.11020100	6.77426195	-2.17499399
C	0.47103500	5.64495277	-2.62968493
O	1.22523105	7.32206011	1.26637101
C	-0.83428502	4.63222504	-4.39696598
H	-1.28856504	4.71683884	-5.37457085
C	-0.10744000	5.70780277	-3.89490390
C	2.28548908	6.62543678	-1.36596298
H	3.13406110	6.37095022	-2.01269794
H	2.16326809	5.82822180	-0.63308400
C	2.52591896	7.94588089	-0.64831501
H	2.66387510	8.74505329	-1.38393199
H	3.45437098	7.86354208	-0.07351400
C	1.38233197	8.33247089	0.27989799
H	1.59109604	9.30102158	0.75984401
H	0.45601901	8.43360329	-0.30052099
C	0.05371600	7.48422098	2.04413891
H	0.09059800	8.40814590	2.64038610
H	-0.01404900	6.62413311	2.71065307
H	-0.84342599	7.51268196	1.41109800
O	-7.19983006	0.79455602	0.37364900
O	-11.31819439	3.03528190	1.15814304

C	-6.36868286	-0.24838600	0.08030800
C	-5.84184313	-2.53270197	-0.48092300
H	-6.15460205	-3.55278492	-0.67181802
C	-6.79091597	-1.54859602	-0.20509399
C	-8.60533428	0.56434000	0.40735301
H	-8.84048176	-0.20523401	1.15500295
H	-8.94869041	0.20232600	-0.57129198
C	-9.27374268	1.88367796	0.76038700
H	-8.89928341	2.23169208	1.72639501
H	-9.00313663	2.63678694	0.01598700
C	-10.78976631	1.76591897	0.82452500
H	-11.19428062	1.42972898	-0.14464800
H	-11.08963490	1.02014506	1.57939506
C	-12.72779942	3.03994799	1.24388599
H	-13.19367409	2.76185703	0.28700301
H	-13.03202724	4.05432320	1.50326896
H	-13.08963203	2.34977889	2.02025890
H	7.68991804	1.67261899	-0.23434199
H	0.00731300	6.61501312	-4.47605419
H	-7.84004784	-1.81117404	-0.20443501
H	-0.01255400	-6.34760618	4.55325794
Cu	-0.34284601	0.88190597	1.02525902
Cu	0.07136000	-0.65995598	-1.05472398
O	0.95110297	-2.00037599	-2.73277903
H	1.85601699	-1.85442805	-2.43227696
H	0.79888201	-2.96260905	-2.71351695
O	-0.84875000	1.92003500	2.96388292
H	-0.56831902	1.25040901	3.59707093
H	-0.49753201	2.77592206	3.26966095

S6.2.2. Frequencies

Mode	IR frequency	IR intensity	Raman intensity
1	4.22720000	0.04650000	0.00000000
2	5.63460000	0.00760000	0.00000000
3	6.25380000	0.06860000	0.00000000
4	8.64210000	0.06840000	0.00000000
5	10.32190000	0.33060000	0.00000000
6	11.78750000	0.09170000	0.00000000
7	15.29660000	0.01230000	0.00000000
8	17.38570000	0.47240000	0.00000000
9	18.40180000	0.54910000	0.00000000
10	19.84390000	0.03520000	0.00000000
11	20.52210000	0.10070000	0.00000000
12	22.85900000	0.12430000	0.00000000
13	25.45460000	0.45580000	0.00000000
14	27.22580000	0.19920000	0.00000000
15	28.40300000	0.14640000	0.00000000
16	29.19170000	0.54570000	0.00000000
17	31.54150000	0.40920000	0.00000000
18	33.26960000	1.31120000	0.00000000
19	37.21080000	1.64270000	0.00000000
20	38.68770000	1.02080000	0.00000000
21	40.32020000	0.50380000	0.00000000
22	40.62790000	0.18250000	0.00000000
23	42.56220000	0.15090000	0.00000000
24	44.91960000	0.29440000	0.00000000
25	47.90070000	3.44390000	0.00000000
26	49.28610000	0.12120000	0.00000000
27	51.89110000	1.95140000	0.00000000
28	52.97860000	2.05440000	0.00000000
29	54.08860000	0.33710000	0.00000000
30	54.85500000	0.10960000	0.00000000
31	60.68510000	0.90720000	0.00000000
32	61.16340000	0.29990000	0.00000000
33	65.54240000	0.20450000	0.00000000
34	68.62640000	0.34930000	0.00000000
35	69.56120000	0.11230000	0.00000000
36	72.47360000	0.37600000	0.00000000
37	74.59140000	1.51590000	0.00000000
38	76.63060000	3.17910000	0.00000000
39	77.52060000	0.56310000	0.00000000
40	79.27700000	0.91750000	0.00000000
41	82.51370000	3.19950000	0.00000000
42	84.62200000	2.61230000	0.00000000
43	86.26580000	0.93090000	0.00000000
44	87.76840000	0.88930000	0.00000000
45	92.26760000	1.30160000	0.00000000
46	93.81180000	0.10580000	0.00000000
47	95.24420000	2.82450000	0.00000000
48	95.64080000	0.32400000	0.00000000
49	98.73440000	2.76480000	0.00000000
50	98.92100000	5.24100000	0.00000000
51	102.92820000	0.15800000	0.00000000
52	105.47680000	1.36130000	0.00000000
53	107.01190000	0.13260000	0.00000000
54	108.10380000	7.74220000	0.00000000
55	111.39610000	3.01510000	0.00000000
56	117.14450000	1.48800000	0.00000000
57	120.99640000	10.08290000	0.00000000
58	122.69920000	1.56710000	0.00000000
59	125.54130000	1.81170000	0.00000000
60	127.77690000	1.31940000	0.00000000

61	130.82890000	1.93890000	0.00000000
62	134.58390000	1.59810000	0.00000000
63	137.98860000	0.24370000	0.00000000
64	138.84760000	6.15570000	0.00000000
65	140.88670000	1.40490000	0.00000000
66	143.14660000	0.62010000	0.00000000
67	147.27270000	0.51010000	0.00000000
68	149.64180000	0.71640000	0.00000000
69	151.48100000	23.90880000	0.00000000
70	153.15740000	1.16450000	0.00000000
71	157.76990000	15.31600000	0.00000000
72	163.55820000	4.20380000	0.00000000
73	165.29370000	1.69020000	0.00000000
74	169.44440000	1.59290000	0.00000000
75	170.58750000	0.25480000	0.00000000
76	172.16970000	1.46290000	0.00000000
77	176.36620000	10.29700000	0.00000000
78	178.34140000	4.99510000	0.00000000
79	178.58410000	3.83770000	0.00000000
80	179.54640000	0.74550000	0.00000000
81	182.46170000	0.40980000	0.00000000
82	191.57940000	5.39320000	0.00000000
83	191.66790000	1.19030000	0.00000000
84	194.44170000	2.22220000	0.00000000
85	199.03580000	1.95760000	0.00000000
86	203.69860000	5.94030000	0.00000000
87	205.12610000	0.09070000	0.00000000
88	206.15600000	0.91480000	0.00000000
89	212.68460000	8.07170000	0.00000000
90	214.17790000	0.75900000	0.00000000
91	217.23850000	6.07300000	0.00000000
92	223.20160000	2.04240000	0.00000000
93	224.28940000	0.70410000	0.00000000
94	226.10800000	0.08100000	0.00000000
95	229.44090000	1.82620000	0.00000000
96	230.87400000	4.74600000	0.00000000
97	230.94690000	0.30670000	0.00000000
98	232.12590000	7.03760000	0.00000000
99	236.89930000	1.77170000	0.00000000
100	240.25770000	8.16130000	0.00000000
101	241.53710000	5.65440000	0.00000000
102	243.55620000	12.20540000	0.00000000
103	246.77530000	3.60230000	0.00000000
104	249.53790000	24.71190000	0.00000000
105	252.58550000	8.38790000	0.00000000
106	255.09090000	3.11630000	0.00000000
107	259.17630000	16.82500000	0.00000000
108	261.05720000	13.60720000	0.00000000
109	267.95290000	6.51430000	0.00000000
110	273.49650000	7.04940000	0.00000000
111	277.59820000	15.07190000	0.00000000
112	281.78250000	9.67150000	0.00000000
113	294.50020000	0.92320000	0.00000000
114	299.91530000	9.85590000	0.00000000
115	307.83150000	0.64970000	0.00000000
116	308.95700000	2.83270000	0.00000000
117	325.52940000	0.06860000	0.00000000
118	327.04240000	2.70690000	0.00000000
119	333.96520000	0.20100000	0.00000000
120	341.37580000	2.79330000	0.00000000
121	355.36440000	31.51290000	0.00000000
122	358.20640000	10.68650000	0.00000000
123	360.74130000	41.03440000	0.00000000
124	366.84810000	3.37430000	0.00000000

125	368.72980000	0.36840000	0.00000000
126	378.91530000	0.01630000	0.00000000
127	381.41780000	0.02610000	0.00000000
128	389.96870000	5.22050000	0.00000000
129	397.29050000	3.75880000	0.00000000
130	402.32320000	18.35310000	0.00000000
131	406.79490000	55.46250000	0.00000000
132	411.86430000	0.11080000	0.00000000
133	421.81380000	11.27330000	0.00000000
134	432.49880000	1.21660000	0.00000000
135	436.76280000	5.05650000	0.00000000
136	440.03740000	2.85470000	0.00000000
137	440.86090000	2.14060000	0.00000000
138	442.56600000	4.37850000	0.00000000
139	444.72550000	3.85100000	0.00000000
140	448.24050000	0.96360000	0.00000000
141	454.91050000	1.14780000	0.00000000
142	456.70170000	15.89800000	0.00000000
143	461.39940000	0.99550000	0.00000000
144	468.51870000	9.47480000	0.00000000
145	475.61380000	0.72260000	0.00000000
146	480.59910000	3.09140000	0.00000000
147	484.19140000	18.72300000	0.00000000
148	485.39640000	37.06020000	0.00000000
149	500.70260000	4.97500000	0.00000000
150	504.06730000	0.59830000	0.00000000
151	505.39410000	8.84650000	0.00000000
152	508.42510000	1.05570000	0.00000000
153	511.33460000	140.63340000	0.00000000
154	514.71990000	13.64790000	0.00000000
155	519.33750000	13.20650000	0.00000000
156	535.99340000	216.66990000	0.00000000
157	537.31470000	13.27130000	0.00000000
158	543.88100000	0.55160000	0.00000000
159	548.96750000	4.71630000	0.00000000
160	560.36710000	5.77720000	0.00000000
161	573.39130000	15.00060000	0.00000000
162	582.25670000	13.14140000	0.00000000
163	609.52890000	6.17010000	0.00000000
164	611.45050000	0.23200000	0.00000000
165	613.60710000	2.00010000	0.00000000
166	623.75530000	12.13910000	0.00000000
167	625.86100000	3.53720000	0.00000000
168	634.63820000	2.88900000	0.00000000
169	640.37080000	0.08620000	0.00000000
170	644.30390000	0.64970000	0.00000000
171	688.72400000	12.49420000	0.00000000
172	690.66220000	5.36250000	0.00000000
173	697.50150000	14.59680000	0.00000000
174	700.69780000	2.59530000	0.00000000
175	701.34580000	48.51340000	0.00000000
176	720.02830000	10.88990000	0.00000000
177	727.39930000	16.40890000	0.00000000
178	729.43380000	47.44800000	0.00000000
179	729.78260000	2.85560000	0.00000000
180	731.59260000	11.04040000	0.00000000
181	755.88670000	55.93490000	0.00000000
182	761.63010000	38.16130000	0.00000000
183	778.13130000	17.66710000	0.00000000
184	781.78920000	19.66200000	0.00000000
185	782.31380000	2.11450000	0.00000000
186	789.34300000	6.31760000	0.00000000
187	792.04700000	2.38290000	0.00000000
188	793.19400000	11.20370000	0.00000000

189	795.48060000	1.06310000	0.00000000
190	796.80630000	31.63330000	0.00000000
191	799.06650000	9.43220000	0.00000000
192	800.09810000	9.00940000	0.00000000
193	807.06560000	21.19050000	0.00000000
194	810.20260000	13.24690000	0.00000000
195	817.73000000	23.63800000	0.00000000
196	821.32290000	19.90570000	0.00000000
197	825.59100000	30.97340000	0.00000000
198	829.71120000	38.70400000	0.00000000
199	831.33690000	20.37770000	0.00000000
200	835.89350000	1.90010000	0.00000000
201	841.94520000	18.44580000	0.00000000
202	843.66790000	11.60140000	0.00000000
203	845.03130000	25.92130000	0.00000000
204	847.40990000	11.59010000	0.00000000
205	882.00040000	9.67320000	0.00000000
206	894.10660000	0.21580000	0.00000000
207	896.60790000	5.80550000	0.00000000
208	899.48490000	3.30470000	0.00000000
209	901.43710000	6.48010000	0.00000000
210	902.97060000	7.35330000	0.00000000
211	903.09310000	0.43910000	0.00000000
212	903.58590000	0.49060000	0.00000000
213	906.43920000	9.03490000	0.00000000
214	907.68180000	7.74540000	0.00000000
215	909.20580000	9.13640000	0.00000000
216	910.02530000	3.19700000	0.00000000
217	931.29500000	26.22990000	0.00000000
218	932.35300000	21.68000000	0.00000000
219	935.99110000	2.07390000	0.00000000
220	937.10750000	11.29590000	0.00000000
221	938.43660000	6.75580000	0.00000000
222	938.98960000	6.26610000	0.00000000
223	944.59360000	11.12150000	0.00000000
224	945.92120000	11.91670000	0.00000000
225	946.47850000	6.85540000	0.00000000
226	947.75260000	16.59960000	0.00000000
227	947.80160000	3.05890000	0.00000000
228	949.04090000	3.99490000	0.00000000
229	949.53820000	5.35460000	0.00000000
230	953.84140000	1.23410000	0.00000000
231	974.48990000	36.79250000	0.00000000
232	974.57200000	0.70590000	0.00000000
233	998.23460000	22.47860000	0.00000000
234	999.65070000	21.21120000	0.00000000
235	1004.16300000	115.61830000	0.00000000
236	1008.25870000	87.41670000	0.00000000
237	1019.43980000	109.22140000	0.00000000
238	1027.28570000	115.61860000	0.00000000
239	1057.59500000	217.07070000	0.00000000
240	1057.81650000	45.05250000	0.00000000
241	1058.28000000	16.37240000	0.00000000
242	1060.79230000	46.43700000	0.00000000
243	1065.10950000	76.50570000	0.00000000
244	1065.73620000	2.16030000	0.00000000
245	1066.54370000	55.04060000	0.00000000
246	1068.14870000	129.18540000	0.00000000
247	1068.26380000	44.17240000	0.00000000
248	1068.89090000	21.99070000	0.00000000
249	1086.35800000	17.80730000	0.00000000
250	1090.18480000	20.52290000	0.00000000
251	1098.07130000	21.36230000	0.00000000
252	1101.03420000	14.77320000	0.00000000

253	1101.03620000	0.36530000	0.00000000
254	1102.97610000	20.55340000	0.00000000
255	1107.04560000	27.99320000	0.00000000
256	1110.77860000	32.34640000	0.00000000
257	1111.14530000	126.41190000	0.00000000
258	1112.48380000	26.54590000	0.00000000
259	1116.13330000	6.57160000	0.00000000
260	1118.61420000	2.40910000	0.00000000
261	1119.48510000	12.86450000	0.00000000
262	1119.90000000	3.89160000	0.00000000
263	1122.43170000	37.55070000	0.00000000
264	1124.64650000	121.70960000	0.00000000
265	1125.06680000	190.95400000	0.00000000
266	1126.66370000	78.80860000	0.00000000
267	1147.88630000	102.85980000	0.00000000
268	1148.60980000	75.30440000	0.00000000
269	1150.29630000	171.44220000	0.00000000
270	1151.47270000	215.92230000	0.00000000
271	1152.74190000	380.60610000	0.00000000
272	1152.92630000	21.41350000	0.00000000
273	1158.88900000	1.91870000	0.00000000
274	1160.63160000	0.12620000	0.00000000
275	1163.92150000	0.23620000	0.00000000
276	1163.93400000	5.58370000	0.00000000
277	1164.04330000	4.94040000	0.00000000
278	1164.13150000	19.74780000	0.00000000
279	1167.08010000	16.23520000	0.00000000
280	1172.94110000	6.23650000	0.00000000
281	1178.41250000	3.86480000	0.00000000
282	1179.30230000	5.89500000	0.00000000
283	1179.72200000	1.89410000	0.00000000
284	1181.40260000	4.20560000	0.00000000
285	1182.29190000	2.23080000	0.00000000
286	1183.60920000	2.52400000	0.00000000
287	1186.80250000	8.29160000	0.00000000
288	1186.84770000	8.90580000	0.00000000
289	1212.66630000	41.31820000	0.00000000
290	1214.15610000	12.38510000	0.00000000
291	1214.52580000	34.46330000	0.00000000
292	1215.94220000	19.74500000	0.00000000
293	1216.92940000	47.00310000	0.00000000
294	1217.60650000	11.88530000	0.00000000
295	1223.14640000	30.71360000	0.00000000
296	1223.18050000	25.61930000	0.00000000
297	1229.02660000	156.66580000	0.00000000
298	1229.16230000	215.92800000	0.00000000
299	1237.41270000	1.02210000	0.00000000
300	1237.44430000	1.01980000	0.00000000
301	1241.64190000	361.45730000	0.00000000
302	1247.98620000	407.78180000	0.00000000
303	1248.30400000	55.77830000	0.00000000
304	1249.82160000	16.45290000	0.00000000
305	1253.02990000	50.24810000	0.00000000
306	1256.77980000	47.55510000	0.00000000
307	1257.34000000	3.04040000	0.00000000
308	1259.43810000	9.03450000	0.00000000
309	1262.44500000	1.95650000	0.00000000
310	1263.31460000	11.40960000	0.00000000
311	1264.15290000	2.71370000	0.00000000
312	1264.93210000	9.68670000	0.00000000
313	1274.69830000	43.69590000	0.00000000
314	1274.84520000	9.84790000	0.00000000
315	1288.28890000	163.59630000	0.00000000
316	1289.11490000	82.16170000	0.00000000

317	1291.54500000	42.05060000	0.00000000
318	1293.90970000	70.11640000	0.00000000
319	1302.76900000	15.53580000	0.00000000
320	1305.14800000	24.34860000	0.00000000
321	1305.77970000	12.01690000	0.00000000
322	1306.15540000	33.83280000	0.00000000
323	1306.33620000	10.52600000	0.00000000
324	1309.13220000	0.27780000	0.00000000
325	1309.16500000	0.27620000	0.00000000
326	1311.76520000	6.05490000	0.00000000
327	1314.26900000	60.36280000	0.00000000
328	1314.85250000	0.21890000	0.00000000
329	1315.13770000	0.45650000	0.00000000
330	1317.64900000	25.57030000	0.00000000
331	1320.03160000	113.97690000	0.00000000
332	1321.40190000	17.73400000	0.00000000
333	1322.44690000	77.76150000	0.00000000
334	1325.20320000	8.79570000	0.00000000
335	1325.74270000	22.49980000	0.00000000
336	1325.99790000	34.42720000	0.00000000
337	1326.61960000	2.90820000	0.00000000
338	1333.19340000	13.03890000	0.00000000
339	1335.72210000	3.73060000	0.00000000
340	1336.25060000	3.10060000	0.00000000
341	1378.08040000	22.32830000	0.00000000
342	1385.26960000	22.23210000	0.00000000
343	1385.54590000	46.28890000	0.00000000
344	1387.56630000	31.78900000	0.00000000
345	1388.44460000	128.77100000	0.00000000
346	1389.65210000	316.49770000	0.00000000
347	1391.81230000	127.35470000	0.00000000
348	1398.54260000	47.91670000	0.00000000
349	1413.90660000	6.68290000	0.00000000
350	1414.04650000	10.19860000	0.00000000
351	1415.19180000	9.53080000	0.00000000
352	1416.08900000	5.76770000	0.00000000
353	1420.96140000	19.37430000	0.00000000
354	1421.65700000	18.78610000	0.00000000
355	1424.49300000	6.28660000	0.00000000
356	1424.60270000	8.69400000	0.00000000
357	1426.73990000	27.84740000	0.00000000
358	1430.86200000	47.70170000	0.00000000
359	1431.47320000	27.26980000	0.00000000
360	1436.36240000	176.65560000	0.00000000
361	1436.54420000	84.49750000	0.00000000
362	1437.40200000	143.43600000	0.00000000
363	1438.08460000	31.96550000	0.00000000
364	1442.29390000	5.04840000	0.00000000
365	1442.35570000	7.86800000	0.00000000
366	1443.24170000	24.47720000	0.00000000
367	1449.67230000	23.95860000	0.00000000
368	1454.76750000	24.40750000	0.00000000
369	1463.36150000	7.28390000	0.00000000
370	1464.23560000	8.07670000	0.00000000
371	1465.98340000	8.51390000	0.00000000
372	1467.16040000	9.18300000	0.00000000
373	1476.36760000	0.64010000	0.00000000
374	1476.89540000	0.67720000	0.00000000
375	1477.37420000	4.20520000	0.00000000
376	1480.20320000	0.90200000	0.00000000
377	1480.63800000	0.09240000	0.00000000
378	1480.79020000	0.10820000	0.00000000
379	1482.72590000	6.13410000	0.00000000
380	1483.13420000	4.70740000	0.00000000

381	1483.77370000	6.96450000	0.00000000
382	1484.04190000	7.10080000	0.00000000
383	1486.21350000	14.87940000	0.00000000
384	1486.37130000	2.38020000	0.00000000
385	1487.29530000	7.68130000	0.00000000
386	1488.10670000	8.06890000	0.00000000
387	1488.47980000	12.75740000	0.00000000
388	1489.90210000	186.17570000	0.00000000
389	1494.36330000	3.40020000	0.00000000
390	1497.56740000	95.18390000	0.00000000
391	1500.29200000	44.04260000	0.00000000
392	1501.54110000	23.66470000	0.00000000
393	1502.06580000	9.56990000	0.00000000
394	1502.09660000	5.28220000	0.00000000
395	1502.51260000	22.54940000	0.00000000
396	1503.69630000	14.04590000	0.00000000
397	1503.87330000	24.21820000	0.00000000
398	1504.08960000	57.28840000	0.00000000
399	1505.48330000	15.58990000	0.00000000
400	1506.19060000	26.24170000	0.00000000
401	1506.25080000	1.94140000	0.00000000
402	1507.59220000	117.34820000	0.00000000
403	1508.57800000	58.42790000	0.00000000
404	1511.68400000	5.49230000	0.00000000
405	1513.92260000	36.91330000	0.00000000
406	1514.12400000	43.63830000	0.00000000
407	1514.21740000	105.44730000	0.00000000
408	1517.57560000	20.13670000	0.00000000
409	1518.36110000	34.47440000	0.00000000
410	1521.90770000	109.67710000	0.00000000
411	1525.97210000	5.18290000	0.00000000
412	1526.15060000	3.24010000	0.00000000
413	1527.03830000	8.42960000	0.00000000
414	1527.15780000	14.21120000	0.00000000
415	1528.27270000	59.11110000	0.00000000
416	1529.59560000	137.88800000	0.00000000
417	1532.26160000	11.14870000	0.00000000
418	1532.93480000	44.44100000	0.00000000
419	1534.15660000	22.33980000	0.00000000
420	1535.53000000	0.84190000	0.00000000
421	1535.84390000	12.32460000	0.00000000
422	1538.36590000	228.86660000	0.00000000
423	1545.76500000	5.98820000	0.00000000
424	1562.62710000	17.48270000	0.00000000
425	1604.85180000	61.00140000	0.00000000
426	1606.21110000	38.89750000	0.00000000
427	1617.04040000	1.47380000	0.00000000
428	1620.05760000	0.95750000	0.00000000
429	1628.57940000	63.87040000	0.00000000
430	1637.48010000	34.98110000	0.00000000
431	1647.56780000	21.92740000	0.00000000
432	1649.48600000	72.68820000	0.00000000
433	1650.90330000	28.65490000	0.00000000
434	1651.65320000	10.99970000	0.00000000
435	2936.05930000	35.34500000	0.00000000
436	2936.84470000	35.35650000	0.00000000
437	2944.79930000	23.44440000	0.00000000
438	2952.50290000	28.97480000	0.00000000
439	2954.07860000	155.44440000	0.00000000
440	2959.11730000	24.38860000	0.00000000
441	2959.44170000	58.47450000	0.00000000
442	2960.32000000	55.31130000	0.00000000
443	2961.12030000	110.32980000	0.00000000
444	2961.16490000	28.39340000	0.00000000

445	2963.15270000	91.18060000	0.00000000
446	2964.34130000	78.46160000	0.00000000
447	2964.34500000	53.75610000	0.00000000
448	2968.46660000	149.99570000	0.00000000
449	2970.93340000	147.75650000	0.00000000
450	2973.71830000	13.07300000	0.00000000
451	2978.88050000	54.39390000	0.00000000
452	2979.34410000	48.97470000	0.00000000
453	2981.01180000	57.81550000	0.00000000
454	2984.63690000	88.77800000	0.00000000
455	2988.08750000	21.12030000	0.00000000
456	2990.12170000	134.95240000	0.00000000
457	2996.94440000	83.76160000	0.00000000
458	2997.95290000	49.07540000	0.00000000
459	3001.06370000	44.58240000	0.00000000
460	3002.97370000	66.42100000	0.00000000
461	3004.72380000	65.27300000	0.00000000
462	3004.98810000	6.16920000	0.00000000
463	3007.16190000	41.41050000	0.00000000
464	3007.88670000	118.71700000	0.00000000
465	3007.93740000	13.51470000	0.00000000
466	3009.27560000	39.68590000	0.00000000
467	3013.12310000	69.74000000	0.00000000
468	3013.29630000	41.93480000	0.00000000
469	3013.44030000	38.69570000	0.00000000
470	3015.31480000	12.82700000	0.00000000
471	3015.51260000	34.22970000	0.00000000
472	3021.47640000	23.42500000	0.00000000
473	3025.75940000	30.22090000	0.00000000
474	3030.93260000	12.54760000	0.00000000
475	3036.09800000	63.22650000	0.00000000
476	3036.83470000	53.45980000	0.00000000
477	3039.38360000	59.01020000	0.00000000
478	3040.03450000	44.26880000	0.00000000
479	3040.51360000	23.44780000	0.00000000
480	3040.85460000	30.94860000	0.00000000
481	3051.90050000	15.72560000	0.00000000
482	3052.58450000	15.28280000	0.00000000
483	3054.60490000	29.36960000	0.00000000
484	3057.17950000	2.14870000	0.00000000
485	3057.36650000	18.72500000	0.00000000
486	3058.06580000	21.96090000	0.00000000
487	3070.45020000	7.85440000	0.00000000
488	3071.53200000	25.09280000	0.00000000
489	3075.82370000	27.88070000	0.00000000
490	3076.66400000	13.59960000	0.00000000
491	3079.80440000	38.69520000	0.00000000
492	3085.50060000	33.53390000	0.00000000
493	3096.53830000	28.22810000	0.00000000
494	3097.15230000	28.22080000	0.00000000
495	3102.82550000	37.98580000	0.00000000
496	3107.92270000	38.50940000	0.00000000
497	3110.86530000	26.48710000	0.00000000
498	3111.20770000	4.68040000	0.00000000
499	3111.50700000	21.16900000	0.00000000
500	3111.76030000	33.34790000	0.00000000
501	3112.74690000	32.03430000	0.00000000
502	3115.25330000	13.64120000	0.00000000
503	3115.36050000	20.80420000	0.00000000
504	3117.65140000	7.41180000	0.00000000
505	3120.34460000	5.84240000	0.00000000
506	3123.55990000	15.33680000	0.00000000
507	3179.41550000	10.25640000	0.00000000
508	3181.16870000	9.22120000	0.00000000

509	3184.02420000	9.94910000	0.00000000
510	3184.24550000	9.05950000	0.00000000
511	3205.70510000	17.00710000	0.00000000
512	3205.90050000	12.05970000	0.00000000
513	3208.48370000	1.27980000	0.00000000
514	3208.53680000	10.63880000	0.00000000
515	3208.96190000	9.50260000	0.00000000
516	3216.63410000	2.80810000	0.00000000
517	3220.38610000	6.01850000	0.00000000
518	3220.48540000	3.67600000	0.00000000
519	3610.63000000	571.87580000	0.00000000
520	3620.59170000	340.12050000	0.00000000
521	3838.62820000	130.63270000	0.00000000
522	3858.87660000	85.47930000	0.00000000

S6.3. Calculations on $Cu_2(1^{**})_4(DMF)_2$

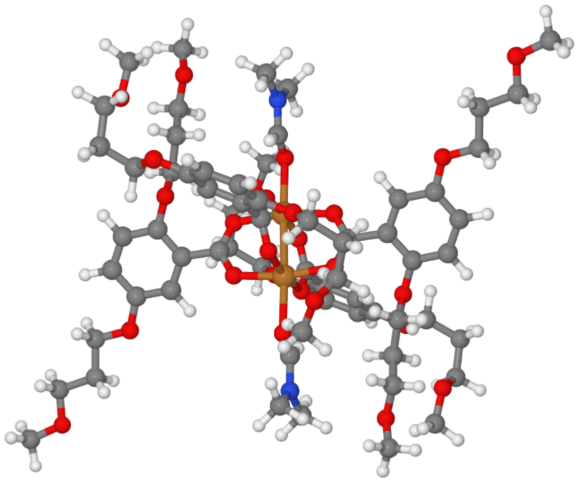

Route	: # opt freq b3lyp/genecp geom=connectivity int=ultrafine scf=noincfock	
SMILES	: CN(C)[CH]O[Cu]1234O[C](O[Cu]1(O[C](O2)c5cc(ccc5OCCCOC)OCCCOC)(O[C](O3)c6cc(ccc6OCCCOC)OCCCOC)(O[C](O4)c7cc(ccc7OCCCOC)OCCCOC)O[CH]N(C)C)c8cc(ccc8OCCCOC)OCCCOC	
Formula	: $C_{66}H_{98}Cu_2N_2O_{26}$	
Charge	: 0	
Multiplicity	: 1	
Dipole	: 0.0105	Debye
Energy	: -5035.33821408	a.u.
Gibbs Energy	: -5033.91839900	a.u.
Number of imaginary frequencies	: 0	

S6.3.1. Cartesian Co-ordinates (XYZ format)

194

O	0.28739399	2.68409204	-2.35433006
O	0.47476900	2.10625005	0.63390702
O	0.15239599	0.58134502	2.26809907
O	-1.84870696	1.04757905	-0.71498799
O	-2.11675406	-0.48665801	0.92002600
C	0.45316300	1.72187603	1.83819306
C	-2.53638411	0.35757399	0.08499000
C	0.39324799	4.94768620	-4.00704098
H	0.14946100	3.93915510	-4.33342695
H	1.33899903	5.26100683	-4.46129799
H	-0.39394501	5.63644695	-4.32840919
O	-1.18213296	2.73977804	3.88988304
O	-4.59668779	-1.76046598	0.27847901
O	-4.21113205	-5.78629398	2.88451600
C	0.10299800	3.19446492	3.88171506
O	-2.24960089	0.09090600	5.92637777
N	0.49851099	4.95047379	-2.55639291
C	-4.96987104	-0.43836701	0.15853301
C	2.23646092	3.20541310	2.71991491

H	2.83404803	2.83925200	1.89618003
C	0.92586499	2.74243999	2.84450698
C	-4.46950388	1.90088201	-0.18831401
H	-3.74880695	2.69590402	-0.32359901
C	-4.02637196	0.59877801	0.03213500
C	-1.96263301	2.87661409	5.07931805
H	-2.23533201	3.92864299	5.23201323
H	-1.38612294	2.52850199	5.94181490
C	0.43516600	3.80079293	-1.85567605
H	0.52680600	3.93147612	-0.77029902
C	-4.60865021	-2.26992702	1.62440097
H	-5.58736181	-2.05764389	2.07613397
H	-3.83368397	-1.75734496	2.20103693
C	-4.36002302	-3.77062607	1.57438803
H	-5.05199718	-4.23847389	0.86954099
H	-3.34543991	-3.95681095	1.21373200
C	-3.21036506	2.02316403	4.90571785
H	-3.76842308	2.36733699	4.02937508
H	-3.84964991	2.16517305	5.78372478
C	-4.53381777	-4.39950895	2.94941211
H	-3.88608503	-3.90656304	3.69087791
H	-5.57346201	-4.28260803	3.29224205
C	-2.89398789	0.54277998	4.74218082
H	-3.82418394	-0.02433400	4.57945013
H	-2.24485397	0.39021200	3.87242699
C	-4.49029016	-6.46238089	4.09534693
H	-3.91181707	-6.04873085	4.93396711
H	-4.21413898	-7.50845480	3.95693207
H	-5.55751085	-6.40592384	4.35097790
C	-1.71762002	-1.21229100	5.79367018
H	-2.51443791	-1.95728302	5.63949585
H	-1.19774902	-1.44450104	6.72473192
H	-1.01355302	-1.27734196	4.95488691
C	0.78039801	6.21812677	-1.89895797
H	1.77161896	6.58219385	-2.18468189
H	0.76039302	6.07855797	-0.81770402
H	0.02531500	6.96296406	-2.16840601
O	4.03348589	4.59798908	3.61977291
C	2.75817108	4.10147476	3.65481400
O	5.85124016	6.03605795	0.82602900
C	0.62608302	4.10803509	4.80314779
H	0.01090200	4.47908306	5.61218500
C	1.94103897	4.54766607	4.69495010
C	4.97742224	3.98052406	2.73619604
H	5.13719606	2.94036198	3.04323697
H	4.60450077	3.98205900	1.71006203
C	6.27073812	4.77702379	2.81085992
H	6.58688784	4.86215496	3.85531211
H	7.04846716	4.22525311	2.27324796
C	6.14796782	6.16991997	2.20846796
H	7.08732414	6.72843981	2.34153008
H	5.34759378	6.72850513	2.71343398
C	5.58709288	7.26836586	0.18520300
H	6.47458315	7.91870117	0.17959100
H	5.29596090	7.03869677	-0.83999199
H	4.76561117	7.80756283	0.67847002
O	-6.13873720	3.52077389	-0.44093201
O	-8.90648079	7.32760096	-1.10306597
C	-5.83104610	2.20399594	-0.24463500
C	-6.32455587	-0.13169900	0.07064700
H	-7.04066181	-0.94284499	0.13697800
C	-6.76793289	1.17603898	-0.11824600
C	-7.50861406	3.89723396	-0.54551500
H	-7.98050880	3.36421800	-1.38198102

H	-8.04332733	3.62491012	0.37462601
C	-7.55051517	5.40062380	-0.76983100
H	-6.99337387	5.64606714	-1.67755306
H	-7.05399513	5.90454197	0.06335800
C	-8.97194576	5.92911720	-0.89783800
H	-9.55243206	5.70633888	0.01278700
H	-9.49117470	5.44591284	-1.74203300
C	-10.17936325	7.92497778	-1.23625100
H	-10.79152298	7.78157711	-0.33369899
H	-10.02038765	8.99273586	-1.38926399
H	-10.72991371	7.51951408	-2.09786391
O	-0.15654600	-0.58023101	-2.26805305
O	2.11656404	0.48345801	-0.92304403
O	-0.28704900	-2.68393993	2.35452700
O	-0.47022200	-2.10741496	-0.63427001
O	1.84935498	-1.04440701	0.71812898
C	-0.45304200	-1.72191799	-1.83821106
C	2.53654504	-0.35754901	-0.08498200
C	-0.39366600	-4.94720221	4.00766516
H	-0.14973800	-3.93865395	4.33389187
H	-1.33950996	-5.26028204	4.46189690
H	0.39338100	-5.63603592	4.32923508
O	1.18196201	-2.73951507	-3.88969803
O	4.59617901	1.76021600	-0.27928400
O	4.21013880	5.78642082	-2.88455892
C	-0.10305300	-3.19462800	-3.88149691
O	2.24943900	-0.09097200	-5.92650700
N	-0.49879500	-4.95023108	2.55700803
C	4.96983910	0.43824399	-0.15930000
C	-2.23648000	-3.20560193	-2.71964502
H	-2.83416390	-2.83934593	-1.89600599
C	-0.92594099	-2.74259996	-2.84435797
C	4.46967316	-1.90090001	0.18855600
H	3.74908996	-2.69594097	0.32439500
C	4.02649879	-0.59889799	-0.03224100
C	1.96258497	-2.87653995	-5.07902479
H	2.23534298	-3.92858696	-5.23148823
H	1.38613200	-2.52862811	-5.94164324
C	-0.43506700	-3.80069995	1.85607398
H	-0.52656603	-3.93157506	0.77071100
C	4.60814095	2.26984501	-1.62514400
H	5.58683205	2.05763698	-2.07694793
H	3.83316493	1.75731397	-2.20182204
C	4.35950422	3.77053404	-1.57486200
H	5.05167294	4.23827791	-0.87013799
H	3.34502506	3.95663810	-1.21387601
C	3.21027708	-2.02301788	-4.90550280
H	3.76829696	-2.36701703	-4.02906895
H	3.84960890	-2.16517997	-5.78345108
C	4.53285980	4.39966011	-2.94982600
H	3.88491106	3.90683007	-3.69117904
H	5.57240200	4.28286314	-3.29300499
C	2.89388299	-0.54260802	-4.74225187
H	3.82407904	0.02454600	-4.57966805
H	2.24483109	-0.38984901	-3.87247205
C	4.48885489	6.46275187	-4.09535503
H	3.91010594	6.04924393	-4.93385315
H	4.21271706	7.50879002	-3.95664191
H	5.55599022	6.40638208	-4.35136604
C	1.71740901	1.21222496	-5.79402924
H	2.51418900	1.95727599	-5.63994980
H	1.19755101	1.44426405	-6.72514105
H	1.01331103	1.27738404	-4.95527792
C	-0.78079897	-6.21795511	1.89975703

H	-1.77214098	-6.58179188	2.18535805
H	-0.76056600	-6.07858896	0.81848103
H	-0.02589900	-6.96287918	2.16948199
O	-4.03335714	-4.59850121	-3.61932707
C	-2.75808597	-4.10187578	-3.65443397
O	-5.85127497	-6.03664207	-0.82596898
C	-0.62600702	-4.10837221	-4.80280876
H	-0.01081400	-4.47941780	-5.61183882
C	-1.94092095	-4.54814100	-4.69450617
C	-4.97737885	-3.98095012	-2.73590994
H	-5.13709116	-2.94079995	-3.04302812
H	-4.60458088	-3.98243690	-1.70973098
C	-6.27071285	-4.77741194	-2.81069207
H	-6.58681393	-4.86245203	-3.85516691
H	-7.04845285	-4.22566223	-2.27307296
C	-6.14800978	-6.17036390	-2.20841908
H	-7.08738995	-6.72882986	-2.34154010
H	-5.34765720	-6.72893476	-2.71343207
C	-5.58714914	-7.26902485	-0.18527700
H	-6.47466516	-7.91932583	-0.17969300
H	-5.29595613	-7.03946590	0.83992398
H	-4.76571178	-7.80820179	-0.67863703
O	6.13910913	-3.52060390	0.44105700
O	8.90729713	-7.32706690	1.10343695
C	5.83127213	-2.20391202	0.24441800
C	6.32457685	0.13172100	-0.07186000
H	7.04064083	0.94286501	-0.13868099
C	6.76806402	-1.17595696	0.11727200
C	7.50904608	-3.89696288	0.54522997
H	7.98124599	-3.36362791	1.38132000
H	8.04337978	-3.62493801	-0.37522000
C	7.55110598	-5.40027189	0.77006000
H	6.99433804	-5.64542103	1.67808998
H	7.05427790	-5.90450716	-0.06275300
C	8.97261238	-5.92865181	0.89768797
H	9.55272579	-5.70616817	-0.01324600
H	9.49215508	-5.44512415	1.74150503
C	10.18025970	-7.92433500	1.23632598
H	10.79205418	-7.78122520	0.33347899
H	10.02139759	-8.99204636	1.38978195
H	10.73113632	-7.51853800	2.09757400
H	-7.82997704	1.37161005	-0.17799900
H	-2.35087991	-5.24578190	-5.41493082
H	7.83013582	-1.37147796	0.17666300
H	2.35104895	5.24516821	5.41548300
Cu	-0.14661200	-0.91094798	0.95804101
Cu	0.14689700	0.91097498	-0.95808798

S6.3.2. Frequencies

Mode	IR frequency	IR intensity	Raman intensity
1	5.21390000	0.04650000	0.00000000
2	7.58940000	0.00000000	0.00000000
3	8.72260000	0.49600000	0.00000000
4	11.28310000	0.31950000	0.00000000
5	12.15000000	0.00000000	0.00000000
6	13.84870000	0.02660000	0.00000000
7	15.52690000	0.41850000	0.00000000
8	15.53530000	0.04460000	0.00000000
9	17.14810000	0.20060000	0.00000000
10	18.22920000	0.00000000	0.00000000
11	18.81780000	1.48820000	0.00000000
12	20.20380000	0.00090000	0.00000000
13	20.32990000	0.67380000	0.00000000
14	22.72370000	0.00020000	0.00000000
15	23.23670000	0.63430000	0.00000000
16	26.14040000	0.00000000	0.00000000
17	27.64210000	0.00000000	0.00000000
18	28.65220000	0.19040000	0.00000000
19	30.87290000	0.00260000	0.00000000
20	30.89390000	2.28860000	0.00000000
21	31.12820000	0.77920000	0.00000000
22	32.46760000	0.00000000	0.00000000
23	36.29700000	2.04540000	0.00000000
24	37.63410000	0.00000000	0.00000000
25	39.94840000	0.32110000	0.00000000
26	41.48490000	3.51180000	0.00000000
27	42.12670000	0.00000000	0.00000000
28	44.14300000	0.00040000	0.00000000
29	44.89150000	1.58610000	0.00000000
30	52.54730000	0.00000000	0.00000000
31	54.30030000	0.46460000	0.00000000
32	54.67760000	0.00010000	0.00000000
33	55.69350000	2.72600000	0.00000000
34	55.94760000	0.00010000	0.00000000
35	56.91940000	1.55300000	0.00000000
36	58.18370000	0.01320000	0.00000000
37	58.71380000	0.00000000	0.00000000
38	60.12900000	0.83780000	0.00000000
39	63.18960000	0.00000000	0.00000000
40	65.32400000	0.00000000	0.00000000
41	70.44300000	3.24650000	0.00000000
42	73.07030000	0.42620000	0.00000000
43	74.84860000	0.00000000	0.00000000
44	78.16540000	0.00010000	0.00000000
45	79.51680000	1.46550000	0.00000000
46	81.31060000	18.05620000	0.00000000
47	81.68600000	0.00000000	0.00000000
48	82.85900000	0.00000000	0.00000000
49	83.97420000	0.00000000	0.00000000
50	85.57700000	0.06960000	0.00000000
51	86.16270000	0.00020000	0.00000000
52	89.29170000	0.64570000	0.00000000
53	89.95500000	0.00080000	0.00000000
54	90.61970000	3.79500000	0.00000000
55	92.90250000	0.00000000	0.00000000
56	96.84370000	3.77400000	0.00000000
57	100.14970000	0.00000000	0.00000000
58	101.13700000	12.44000000	0.00000000
59	101.60350000	4.12440000	0.00000000
60	102.82890000	0.00010000	0.00000000

61	104.44410000	0.00000000	0.00000000
62	105.87040000	0.00020000	0.00000000
63	106.58660000	0.95640000	0.00000000
64	114.16220000	0.00000000	0.00000000
65	119.42330000	0.03540000	0.00000000
66	119.54590000	1.43640000	0.00000000
67	124.44370000	0.00000000	0.00000000
68	125.03500000	1.57310000	0.00000000
69	128.43540000	0.00040000	0.00000000
70	128.85490000	5.18990000	0.00000000
71	129.74540000	3.65170000	0.00000000
72	132.79720000	0.00180000	0.00000000
73	134.50110000	12.15110000	0.00000000
74	134.76150000	0.00360000	0.00000000
75	137.15650000	0.00000000	0.00000000
76	138.74750000	0.28570000	0.00000000
77	140.11050000	0.00000000	0.00000000
78	141.86580000	5.84500000	0.00000000
79	142.10290000	0.52900000	0.00000000
80	145.07910000	0.00000000	0.00000000
81	150.23010000	7.86700000	0.00000000
82	151.99350000	0.00000000	0.00000000
83	160.52280000	4.00000000	0.00000000
84	165.72720000	0.00010000	0.00000000
85	167.61950000	5.98390000	0.00000000
86	169.85800000	0.57980000	0.00000000
87	170.89460000	0.00000000	0.00000000
88	176.71370000	0.00000000	0.00000000
89	181.41920000	7.01440000	0.00000000
90	181.58730000	0.00090000	0.00000000
91	184.20690000	0.00180000	0.00000000
92	184.31440000	1.30880000	0.00000000
93	190.90620000	0.00000000	0.00000000
94	193.47260000	4.29340000	0.00000000
95	196.33280000	0.00000000	0.00000000
96	198.62030000	0.00000000	0.00000000
97	200.01340000	4.39460000	0.00000000
98	203.40200000	19.63050000	0.00000000
99	203.63170000	0.00280000	0.00000000
100	205.37270000	0.00010000	0.00000000
101	207.62970000	0.83670000	0.00000000
102	215.02520000	2.50490000	0.00000000
103	219.36080000	8.76380000	0.00000000
104	221.70530000	0.00080000	0.00000000
105	225.46510000	0.00010000	0.00000000
106	229.43620000	6.21580000	0.00000000
107	230.55390000	0.00010000	0.00000000
108	231.08530000	0.00030000	0.00000000
109	231.13270000	4.97450000	0.00000000
110	234.04190000	0.00110000	0.00000000
111	234.48480000	7.59020000	0.00000000
112	242.25650000	0.00030000	0.00000000
113	242.85080000	3.84110000	0.00000000
114	245.65640000	21.58710000	0.00000000
115	247.46000000	0.00300000	0.00000000
116	247.92150000	0.00170000	0.00000000
117	252.02850000	8.45180000	0.00000000
118	252.49210000	0.00050000	0.00000000
119	254.26500000	29.70890000	0.00000000
120	259.63400000	0.00000000	0.00000000
121	260.29810000	22.36160000	0.00000000
122	267.28300000	4.86340000	0.00000000
123	269.78680000	0.00020000	0.00000000
124	278.90980000	16.30380000	0.00000000

125	302.05330000	0.01650000	0.00000000
126	302.68490000	5.51910000	0.00000000
127	309.25100000	0.00020000	0.00000000
128	309.95990000	2.01970000	0.00000000
129	325.06400000	0.00000000	0.00000000
130	327.11120000	4.32760000	0.00000000
131	334.74100000	0.00010000	0.00000000
132	343.93450000	5.68990000	0.00000000
133	352.93300000	52.03590000	0.00000000
134	353.49850000	0.00100000	0.00000000
135	358.40240000	12.85340000	0.00000000
136	361.25350000	0.00030000	0.00000000
137	366.01310000	0.00020000	0.00000000
138	366.37480000	40.73910000	0.00000000
139	368.34950000	4.25550000	0.00000000
140	370.04330000	0.00030000	0.00000000
141	376.86050000	1.32680000	0.00000000
142	379.07380000	0.00000000	0.00000000
143	390.64510000	0.00230000	0.00000000
144	392.78450000	12.69820000	0.00000000
145	402.29750000	0.00020000	0.00000000
146	402.51840000	4.83410000	0.00000000
147	405.06660000	10.42300000	0.00000000
148	410.03850000	0.00020000	0.00000000
149	420.26300000	19.64630000	0.00000000
150	431.63930000	0.00020000	0.00000000
151	437.59890000	10.16740000	0.00000000
152	440.87010000	0.00000000	0.00000000
153	441.47380000	3.42040000	0.00000000
154	443.57910000	0.00000000	0.00000000
155	443.97460000	2.80110000	0.00000000
156	445.70610000	0.00050000	0.00000000
157	450.97920000	0.00030000	0.00000000
158	453.58640000	10.86680000	0.00000000
159	458.31520000	0.00020000	0.00000000
160	462.55460000	15.84550000	0.00000000
161	474.18820000	0.00010000	0.00000000
162	480.50690000	37.97590000	0.00000000
163	480.71360000	0.00360000	0.00000000
164	483.80300000	20.84780000	0.00000000
165	499.97010000	0.19720000	0.00000000
166	504.33840000	0.00000000	0.00000000
167	509.65330000	0.00000000	0.00000000
168	511.36230000	7.50680000	0.00000000
169	512.45220000	37.36580000	0.00000000
170	517.45910000	0.00010000	0.00000000
171	538.88750000	0.65280000	0.00000000
172	539.24500000	0.00040000	0.00000000
173	552.21770000	3.31920000	0.00000000
174	552.27760000	5.51220000	0.00000000
175	578.77570000	22.36890000	0.00000000
176	579.30430000	0.15230000	0.00000000
177	612.13310000	2.56230000	0.00000000
178	613.05310000	0.00010000	0.00000000
179	623.47220000	9.57310000	0.00000000
180	623.86880000	0.06100000	0.00000000
181	629.63500000	10.47650000	0.00000000
182	634.50800000	0.00270000	0.00000000
183	641.64170000	0.24820000	0.00000000
184	643.87830000	0.00000000	0.00000000
185	672.00400000	46.58830000	0.00000000
186	672.25630000	0.00310000	0.00000000
187	687.14170000	16.18560000	0.00000000
188	688.27990000	0.00000000	0.00000000

189	696.97880000	17.44400000	0.00000000
190	698.87890000	0.00290000	0.00000000
191	727.12060000	0.10160000	0.00000000
192	728.02600000	46.25300000	0.00000000
193	729.45140000	10.57690000	0.00000000
194	730.41130000	0.00900000	0.00000000
195	763.27510000	88.81430000	0.00000000
196	763.92420000	0.02830000	0.00000000
197	785.32040000	15.66920000	0.00000000
198	785.63520000	0.06220000	0.00000000
199	789.00880000	2.22790000	0.00000000
200	789.03750000	20.90390000	0.00000000
201	793.19140000	0.00550000	0.00000000
202	793.41500000	9.81550000	0.00000000
203	796.77840000	0.00020000	0.00000000
204	797.84490000	44.24980000	0.00000000
205	800.04340000	16.26930000	0.00000000
206	800.81090000	0.00440000	0.00000000
207	807.88500000	40.43300000	0.00000000
208	809.42320000	0.00780000	0.00000000
209	823.67720000	51.87150000	0.00000000
210	823.99300000	0.06380000	0.00000000
211	827.01330000	45.69620000	0.00000000
212	827.97660000	0.00050000	0.00000000
213	831.72570000	23.66940000	0.00000000
214	835.64650000	0.00040000	0.00000000
215	840.10840000	13.62510000	0.00000000
216	841.48860000	0.00210000	0.00000000
217	843.15420000	52.83710000	0.00000000
218	845.49840000	0.00100000	0.00000000
219	876.81570000	6.63290000	0.00000000
220	876.84310000	0.00540000	0.00000000
221	896.53030000	4.06260000	0.00000000
222	896.65670000	0.00080000	0.00000000
223	897.60280000	0.00140000	0.00000000
224	897.83860000	3.08920000	0.00000000
225	898.92340000	0.99100000	0.00000000
226	899.10680000	0.00000000	0.00000000
227	902.68540000	3.21400000	0.00000000
228	902.72010000	3.54420000	0.00000000
229	905.41550000	6.47440000	0.00000000
230	905.47250000	7.89880000	0.00000000
231	906.74850000	18.81390000	0.00000000
232	906.83660000	3.94130000	0.00000000
233	934.88840000	7.68020000	0.00000000
234	934.94120000	4.07750000	0.00000000
235	938.43530000	9.23070000	0.00000000
236	939.01970000	0.00150000	0.00000000
237	940.34550000	29.23930000	0.00000000
238	940.35320000	0.17620000	0.00000000
239	945.13040000	27.43650000	0.00000000
240	945.14240000	0.02350000	0.00000000
241	945.63750000	8.51870000	0.00000000
242	945.72650000	0.50040000	0.00000000
243	949.89980000	10.51280000	0.00000000
244	950.87220000	0.00010000	0.00000000
245	957.97880000	0.14490000	0.00000000
246	958.03480000	39.37050000	0.00000000
247	974.31970000	35.71200000	0.00000000
248	974.39220000	0.00130000	0.00000000
249	997.07560000	0.02740000	0.00000000
250	997.14800000	52.13440000	0.00000000
251	1006.88510000	198.88760000	0.00000000
252	1007.11690000	0.01710000	0.00000000

253	1026.88940000	0.00460000	0.00000000
254	1027.03230000	216.60870000	0.00000000
255	1050.97210000	0.06190000	0.00000000
256	1051.00630000	0.16000000	0.00000000
257	1057.93430000	170.23910000	0.00000000
258	1058.05950000	0.00550000	0.00000000
259	1061.45240000	0.00160000	0.00000000
260	1061.48690000	93.92640000	0.00000000
261	1062.38900000	0.00290000	0.00000000
262	1062.47040000	21.92600000	0.00000000
263	1065.64260000	14.65310000	0.00000000
264	1065.66880000	161.75150000	0.00000000
265	1068.36610000	193.63960000	0.00000000
266	1068.43330000	0.03870000	0.00000000
267	1080.69910000	23.12640000	0.00000000
268	1080.75690000	0.00080000	0.00000000
269	1094.27200000	9.08440000	0.00000000
270	1094.27790000	12.98120000	0.00000000
271	1101.10480000	11.01850000	0.00000000
272	1101.11510000	0.00020000	0.00000000
273	1101.66860000	33.98570000	0.00000000
274	1101.83150000	0.02820000	0.00000000
275	1111.52200000	166.86110000	0.00000000
276	1111.63850000	0.05700000	0.00000000
277	1111.89230000	97.14570000	0.00000000
278	1112.11180000	0.00730000	0.00000000
279	1112.61550000	292.51030000	0.00000000
280	1114.02070000	0.00230000	0.00000000
281	1116.70420000	46.75430000	0.00000000
282	1116.80700000	0.00010000	0.00000000
283	1118.68200000	10.91330000	0.00000000
284	1118.91660000	0.00110000	0.00000000
285	1122.79910000	108.62610000	0.00000000
286	1123.51290000	0.00310000	0.00000000
287	1129.93450000	1.48320000	0.00000000
288	1129.94480000	1.21790000	0.00000000
289	1137.99840000	346.22190000	0.00000000
290	1138.11860000	0.00230000	0.00000000
291	1149.13490000	0.00190000	0.00000000
292	1149.32370000	183.22740000	0.00000000
293	1149.96530000	22.79400000	0.00000000
294	1149.97580000	303.09910000	0.00000000
295	1152.70760000	449.79910000	0.00000000
296	1152.86150000	0.00010000	0.00000000
297	1161.25630000	1.02120000	0.00000000
298	1161.26260000	6.63690000	0.00000000
299	1164.02580000	0.26200000	0.00000000
300	1164.02650000	0.44780000	0.00000000
301	1165.50880000	0.00650000	0.00000000
302	1165.58600000	31.38870000	0.00000000
303	1167.86590000	14.46100000	0.00000000
304	1167.95260000	0.07460000	0.00000000
305	1178.63620000	5.56550000	0.00000000
306	1178.63800000	0.73520000	0.00000000
307	1179.21400000	4.86070000	0.00000000
308	1179.21780000	2.49790000	0.00000000
309	1181.78480000	0.00840000	0.00000000
310	1181.78980000	8.07010000	0.00000000
311	1183.49880000	2.54190000	0.00000000
312	1183.50550000	12.15250000	0.00000000
313	1186.83620000	1.42400000	0.00000000
314	1186.83790000	15.71240000	0.00000000
315	1217.00560000	61.55060000	0.00000000
316	1217.01490000	0.33170000	0.00000000

317	1217.47940000	0.00150000	0.00000000
318	1217.53260000	18.10080000	0.00000000
319	1219.19890000	6.62830000	0.00000000
320	1219.24380000	0.00000000	0.00000000
321	1223.11270000	54.55570000	0.00000000
322	1223.11670000	0.01660000	0.00000000
323	1228.31850000	0.48130000	0.00000000
324	1228.41570000	465.52000000	0.00000000
325	1237.14550000	1.49850000	0.00000000
326	1237.14560000	0.62700000	0.00000000
327	1242.20420000	13.49460000	0.00000000
328	1242.25850000	0.00460000	0.00000000
329	1247.48430000	0.04560000	0.00000000
330	1248.22980000	869.70380000	0.00000000
331	1255.54820000	9.98320000	0.00000000
332	1255.56140000	0.29450000	0.00000000
333	1260.85980000	5.65700000	0.00000000
334	1260.90080000	33.21150000	0.00000000
335	1262.96120000	0.55890000	0.00000000
336	1262.96830000	1.80000000	0.00000000
337	1265.54760000	20.56900000	0.00000000
338	1265.55130000	15.65650000	0.00000000
339	1271.63020000	28.29390000	0.00000000
340	1271.74370000	0.24520000	0.00000000
341	1276.89930000	54.40780000	0.00000000
342	1276.92730000	0.08670000	0.00000000
343	1289.06460000	276.24420000	0.00000000
344	1289.32790000	0.06700000	0.00000000
345	1295.60920000	57.95780000	0.00000000
346	1295.87840000	0.00160000	0.00000000
347	1305.52270000	57.43020000	0.00000000
348	1305.55890000	0.06970000	0.00000000
349	1306.04530000	22.07360000	0.00000000
350	1306.39550000	0.01340000	0.00000000
351	1307.64560000	8.36520000	0.00000000
352	1307.76600000	0.00010000	0.00000000
353	1309.19520000	0.01420000	0.00000000
354	1309.19630000	0.57650000	0.00000000
355	1314.39340000	0.67990000	0.00000000
356	1314.39510000	0.40890000	0.00000000
357	1315.49630000	9.13370000	0.00000000
358	1315.52570000	3.01430000	0.00000000
359	1318.34470000	0.00060000	0.00000000
360	1318.44990000	41.07460000	0.00000000
361	1321.61670000	140.42390000	0.00000000
362	1321.83450000	0.12010000	0.00000000
363	1323.91390000	0.00480000	0.00000000
364	1324.03050000	77.00980000	0.00000000
365	1325.19640000	0.02810000	0.00000000
366	1325.24540000	17.82950000	0.00000000
367	1328.03650000	26.51500000	0.00000000
368	1328.16330000	0.00790000	0.00000000
369	1383.63440000	0.01350000	0.00000000
370	1386.26130000	21.53440000	0.00000000
371	1386.50510000	0.00340000	0.00000000
372	1388.73910000	29.28830000	0.00000000
373	1389.32530000	0.01320000	0.00000000
374	1390.71140000	365.32530000	0.00000000
375	1397.38840000	193.77880000	0.00000000
376	1400.71180000	0.02490000	0.00000000
377	1413.77560000	25.67870000	0.00000000
378	1413.78880000	1.85270000	0.00000000
379	1417.13550000	8.29870000	0.00000000
380	1417.14310000	0.49640000	0.00000000

381	1418.39710000	258.11390000	0.00000000
382	1419.61270000	0.03690000	0.00000000
383	1422.06680000	78.03510000	0.00000000
384	1422.37990000	0.00030000	0.00000000
385	1425.53710000	5.14540000	0.00000000
386	1425.56420000	0.13530000	0.00000000
387	1433.74300000	0.00010000	0.00000000
388	1433.79500000	67.88120000	0.00000000
389	1435.43320000	59.84740000	0.00000000
390	1435.49880000	0.06590000	0.00000000
391	1435.80990000	125.21360000	0.00000000
392	1436.01520000	0.02520000	0.00000000
393	1436.48280000	248.61990000	0.00000000
394	1436.51310000	0.43200000	0.00000000
395	1440.79320000	29.86440000	0.00000000
396	1440.81030000	1.53810000	0.00000000
397	1444.22440000	125.49500000	0.00000000
398	1444.98360000	0.12920000	0.00000000
399	1446.07770000	0.73660000	0.00000000
400	1446.14160000	30.07200000	0.00000000
401	1451.44420000	45.15570000	0.00000000
402	1452.11660000	0.03440000	0.00000000
403	1463.87400000	18.16980000	0.00000000
404	1463.88010000	0.16160000	0.00000000
405	1465.26500000	13.15740000	0.00000000
406	1465.27970000	0.01860000	0.00000000
407	1471.38430000	33.56010000	0.00000000
408	1471.46840000	0.05130000	0.00000000
409	1478.90320000	1.31710000	0.00000000
410	1478.91380000	0.00290000	0.00000000
411	1480.72310000	0.11640000	0.00000000
412	1480.72340000	0.04310000	0.00000000
413	1480.85290000	0.00750000	0.00000000
414	1480.86340000	1.33860000	0.00000000
415	1483.82470000	0.45750000	0.00000000
416	1483.82530000	13.33240000	0.00000000
417	1484.29010000	0.06600000	0.00000000
418	1484.29780000	7.07050000	0.00000000
419	1485.65600000	15.11130000	0.00000000
420	1485.67500000	0.00930000	0.00000000
421	1485.93220000	11.77120000	0.00000000
422	1485.98350000	0.01250000	0.00000000
423	1486.39300000	0.00940000	0.00000000
424	1486.43250000	7.17610000	0.00000000
425	1494.67290000	13.22270000	0.00000000
426	1494.69190000	0.14920000	0.00000000
427	1499.70760000	196.97770000	0.00000000
428	1500.92830000	0.14940000	0.00000000
429	1500.94680000	8.27880000	0.00000000
430	1502.10090000	6.99430000	0.00000000
431	1502.10270000	0.08500000	0.00000000
432	1503.61110000	50.65900000	0.00000000
433	1503.89220000	0.00120000	0.00000000
434	1504.62980000	0.00170000	0.00000000
435	1504.65900000	35.69120000	0.00000000
436	1505.69230000	0.02900000	0.00000000
437	1505.71120000	36.95680000	0.00000000
438	1505.78100000	128.90430000	0.00000000
439	1505.85260000	0.11530000	0.00000000
440	1505.94980000	0.01270000	0.00000000
441	1506.54440000	22.92810000	0.00000000
442	1507.01170000	217.26130000	0.00000000
443	1507.22180000	0.02890000	0.00000000
444	1508.05100000	104.73930000	0.00000000

445	1509.08700000	0.00390000	0.00000000
446	1512.30880000	74.14390000	0.00000000
447	1513.14680000	0.00670000	0.00000000
448	1513.78540000	10.63820000	0.00000000
449	1513.79810000	1.18450000	0.00000000
450	1519.48010000	189.59150000	0.00000000
451	1520.28170000	0.01090000	0.00000000
452	1522.56100000	35.96830000	0.00000000
453	1526.29310000	0.00060000	0.00000000
454	1527.15800000	35.21280000	0.00000000
455	1527.37240000	0.00290000	0.00000000
456	1527.69480000	57.68840000	0.00000000
457	1528.36910000	0.00480000	0.00000000
458	1530.96290000	106.35160000	0.00000000
459	1533.71460000	0.00390000	0.00000000
460	1533.90000000	10.28760000	0.00000000
461	1535.66920000	0.00020000	0.00000000
462	1535.91380000	12.14330000	0.00000000
463	1538.52160000	0.00650000	0.00000000
464	1539.75220000	293.49240000	0.00000000
465	1542.57740000	0.00040000	0.00000000
466	1542.92730000	26.04490000	0.00000000
467	1554.84760000	0.00190000	0.00000000
468	1565.72690000	0.00210000	0.00000000
469	1606.63170000	83.91880000	0.00000000
470	1606.95650000	0.17120000	0.00000000
471	1621.51390000	8.45750000	0.00000000
472	1621.73600000	0.08130000	0.00000000
473	1648.62300000	40.12150000	0.00000000
474	1648.95940000	102.00780000	0.00000000
475	1649.20990000	0.04200000	0.00000000
476	1650.31460000	0.00050000	0.00000000
477	1706.14780000	1377.35070000	0.00000000
478	1709.51840000	0.00370000	0.00000000
479	2936.86970000	0.15830000	0.00000000
480	2936.87320000	69.28670000	0.00000000
481	2949.55300000	2.80840000	0.00000000
482	2949.56790000	58.50700000	0.00000000
483	2953.24100000	0.65390000	0.00000000
484	2953.25880000	71.69600000	0.00000000
485	2956.17780000	5.69420000	0.00000000
486	2956.18090000	42.32740000	0.00000000
487	2958.90260000	0.56850000	0.00000000
488	2959.01800000	263.28630000	0.00000000
489	2960.44420000	0.07860000	0.00000000
490	2960.45010000	115.11130000	0.00000000
491	2963.68510000	179.40950000	0.00000000
492	2963.70020000	0.08610000	0.00000000
493	2966.23940000	305.43330000	0.00000000
494	2966.28920000	0.20030000	0.00000000
495	2970.96150000	171.53240000	0.00000000
496	2970.97340000	0.28520000	0.00000000
497	2979.38390000	102.76870000	0.00000000
498	2979.40700000	1.36410000	0.00000000
499	2981.52150000	3.66070000	0.00000000
500	2981.53890000	79.32890000	0.00000000
501	2987.63080000	125.33890000	0.00000000
502	2987.69040000	11.17400000	0.00000000
503	2996.18030000	105.35120000	0.00000000
504	2996.22480000	0.01250000	0.00000000
505	3002.03500000	27.43050000	0.00000000
506	3002.04160000	146.83340000	0.00000000
507	3003.70940000	3.18040000	0.00000000
508	3003.71370000	127.90040000	0.00000000

509	3004.66950000	21.77740000	0.00000000
510	3004.67290000	3.02340000	0.00000000
511	3012.98610000	121.63380000	0.00000000
512	3012.98960000	16.99270000	0.00000000
513	3013.40380000	5.83270000	0.00000000
514	3013.41430000	62.95410000	0.00000000
515	3016.33750000	20.89530000	0.00000000
516	3016.34250000	23.85110000	0.00000000
517	3016.39840000	5.05970000	0.00000000
518	3016.41290000	46.72800000	0.00000000
519	3021.36180000	140.33770000	0.00000000
520	3021.44720000	0.05550000	0.00000000
521	3025.42090000	34.61660000	0.00000000
522	3025.43770000	27.84830000	0.00000000
523	3031.74130000	44.91910000	0.00000000
524	3031.74810000	0.00000000	0.00000000
525	3038.89740000	41.88780000	0.00000000
526	3038.90110000	39.35120000	0.00000000
527	3043.61650000	37.75350000	0.00000000
528	3043.62970000	2.96410000	0.00000000
529	3051.77620000	3.95850000	0.00000000
530	3051.77680000	23.71330000	0.00000000
531	3052.35870000	49.13290000	0.00000000
532	3052.46490000	1.25240000	0.00000000
533	3053.89770000	1.19730000	0.00000000
534	3053.90990000	23.87120000	0.00000000
535	3058.75060000	20.14050000	0.00000000
536	3058.76620000	19.05120000	0.00000000
537	3065.23950000	7.55830000	0.00000000
538	3065.27940000	17.91900000	0.00000000
539	3067.29720000	3.22690000	0.00000000
540	3067.31890000	68.48420000	0.00000000
541	3074.82850000	4.30300000	0.00000000
542	3074.83570000	32.78800000	0.00000000
543	3077.83670000	0.00620000	0.00000000
544	3077.84160000	21.75570000	0.00000000
545	3079.34880000	78.13200000	0.00000000
546	3079.35820000	0.06270000	0.00000000
547	3092.06880000	2.71820000	0.00000000
548	3092.07250000	50.81880000	0.00000000
549	3096.33140000	0.12950000	0.00000000
550	3096.33700000	57.08790000	0.00000000
551	3099.93900000	79.74930000	0.00000000
552	3099.95500000	0.02110000	0.00000000
553	3102.77230000	36.18510000	0.00000000
554	3102.77790000	4.76280000	0.00000000
555	3107.63790000	3.89570000	0.00000000
556	3107.64000000	54.74970000	0.00000000
557	3111.77720000	59.58330000	0.00000000
558	3111.77840000	6.15220000	0.00000000
559	3117.49590000	4.96400000	0.00000000
560	3117.50960000	5.47060000	0.00000000
561	3121.21230000	10.55050000	0.00000000
562	3121.21400000	9.11850000	0.00000000
563	3152.26500000	0.85750000	0.00000000
564	3152.26840000	0.71060000	0.00000000
565	3177.24000000	13.28890000	0.00000000
566	3177.25150000	8.48210000	0.00000000
567	3182.86420000	8.66170000	0.00000000
568	3182.87000000	10.15360000	0.00000000
569	3202.70300000	18.92890000	0.00000000
570	3202.72010000	1.09660000	0.00000000
571	3205.01540000	27.10060000	0.00000000
572	3205.03400000	1.36910000	0.00000000

573	3206.71960000	4.67540000	0.00000000
574	3206.87180000	4.12430000	0.00000000
575	3213.12520000	3.05070000	0.00000000
576	3213.22650000	2.06520000	0.00000000

S6.4. Calculations on $Cu_2(1^{**})_4(MeCN)_2$

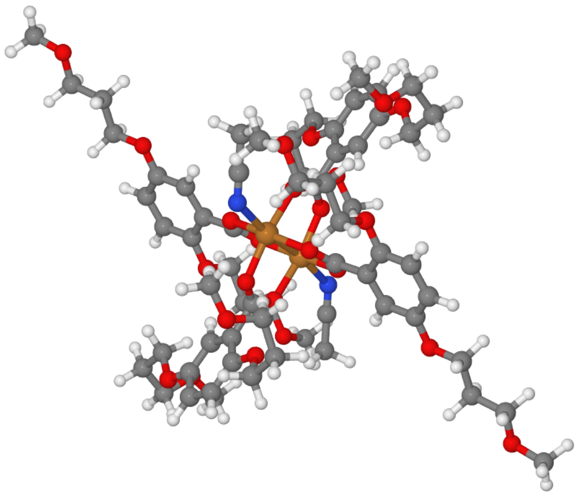

Route	: # opt freq b3lyp/genecp geom=connectivity int=ultrafine scf=noincfock		
SMILES	: C[C][N][Cu]1234O[C](O[Cu]1(O[C](O2)c5cc(ccc5OCCCOC)OCCCOC)(O[C](O3)c6cc(ccc6OCCCOC)OCCCOC)(O[C](O4)c7cc(ccc7OCCCOC)OCCCOC)[N][C]C)c8cc(ccc8OCCCOC)OCCCOC		
Formula	: $C_{64}H_{90}Cu_2N_2O_{24}$		
Charge	: 0		
Multiplicity	: 1		
Dipole	: 0.0375		Debye
Energy	: -4803.74640163		a.u.
Gibbs Energy	: -4802.43539900		a.u.
Number of imaginary frequencies	: 0		

S6.4.1. Cartesian Co-ordinates (XYZ format)

182

O	-0.17591999	2.23092890	-0.35300601
O	-0.06323100	0.87411302	-2.15737391
O	1.96234798	0.72246599	0.83273399
O	2.03534389	-0.63441199	-0.97088999
C	-0.20018999	1.98888397	-1.59315801
C	2.55866599	0.04918600	-0.05080600
O	1.57929695	2.97748399	-3.53730607
O	4.32258701	-2.27688503	-0.48432100
O	2.83191395	-5.75037289	-3.44400597
C	0.38010699	3.62008190	-3.45614409
O	2.22924209	0.52001500	-5.97426605
C	4.86848879	-1.03496695	-0.24265400
C	-1.73575997	3.81438994	-2.27746797
H	-2.38913393	3.44282603	-1.50013006
C	-0.50905299	3.17775989	-2.47051191
C	4.67235708	1.30209005	0.34602401
H	4.06044579	2.16461992	0.57273000
C	4.06657314	0.09575200	0.00377000
C	2.38657093	3.15198994	-4.70436621

H	2.83122492	4.15528584	-4.70680189
H	1.77160895	3.03171492	-5.60135221
C	4.23350191	-2.63853502	-1.87470102
H	5.24135590	-2.63353992	-2.31208992
H	3.61705995	-1.89875805	-2.39237905
C	3.60960507	-4.02255297	-1.96204603
H	4.20288515	-4.74020815	-1.38963604
H	2.61412692	-3.97951388	-1.51398802
C	3.47160196	2.08591294	-4.67055178
H	4.06164694	2.19475508	-3.75526595
H	4.14272594	2.24964809	-5.52049208
C	3.49811506	-4.48868990	-3.40607500
H	2.93498492	-3.76029897	-4.00842905
H	4.49674511	-4.59153080	-3.85753894
C	2.90822911	0.67267698	-4.73481798
H	3.72566295	-0.06182000	-4.65954781
H	2.22000408	0.50471097	-3.89831901
C	2.75289702	-6.28065205	-4.75474691
H	2.19591808	-5.61362123	-5.42739105
H	2.23183608	-7.23660278	-4.68923187
H	3.75201011	-6.44809008	-5.17975712
C	1.47738397	-0.67590100	-6.04246187
H	2.12766290	-1.56320000	-5.99064493
H	0.96021700	-0.67758900	-7.00324297
H	0.74069101	-0.73651999	-5.23190784
O	-3.29019690	5.56753111	-2.97846293
C	-2.10769892	4.88719511	-3.08969998
O	-4.72875786	6.90635014	0.04494900
C	0.00845300	4.70792294	-4.25378418
H	0.67841500	5.07649612	-5.01917791
C	-1.22418904	5.32677317	-4.07714319
C	-4.31778812	5.01430178	-2.14744306
H	-4.67061901	4.07204390	-2.58256102
H	-3.93473792	4.80889177	-1.14639497
C	-5.44273376	6.03377581	-2.05945706
H	-5.76042986	6.31776381	-3.06765294
H	-6.29730320	5.56289911	-1.56309795
C	-5.04815102	7.28493404	-1.28616095
H	-5.87482882	8.01186657	-1.28606606
H	-4.17876720	7.76061106	-1.76085603
C	-4.20503616	7.96475887	0.82245302
H	-4.95302486	8.75471115	0.98616302
H	-3.90665793	7.53897285	1.78068697
H	-3.32413507	8.41490936	0.34282801
O	6.53661489	2.66192889	0.73064500
O	9.77083015	5.99824715	1.72959399
C	6.06211281	1.42218900	0.40816301
C	6.25134706	-0.91342700	-0.14976899
H	6.85732889	-1.79774797	-0.31028101
C	6.85875797	0.30187801	0.16175300
C	7.94420385	2.84872103	0.85004699
H	8.34650326	2.17691207	1.62019396
H	8.43600464	2.60652900	-0.10182100
C	8.17891026	4.30350399	1.22487605
H	7.66021395	4.52346087	2.16145992
H	7.74914312	4.94934797	0.45489100
C	9.65667915	4.63109016	1.38371503
H	10.20185471	4.43016481	0.44656801
H	10.11153126	4.00235510	2.16698909
C	11.10988140	6.41241503	1.90446103
H	11.69771481	6.28484678	0.98358101
H	11.08875847	7.47057009	2.16637897
H	11.60480213	5.85483122	2.71313500
O	0.06112100	-0.87437803	2.15742993

O	-2.03572106	0.63541597	0.96984702
O	0.17713501	-2.23091602	0.35307100
O	-1.96296799	-0.72410297	-0.83177400
C	0.19974300	-1.98899698	1.59326303
C	-2.55919194	-0.04950100	0.05077700
O	-1.57908905	-2.97743201	3.53845191
O	-4.32304287	2.27695799	0.48365900
O	-2.83013892	5.75081587	3.44165707
C	-0.37985501	-3.61986208	3.45688105
O	-2.22796512	-0.51934701	5.97504902
C	-4.86895180	1.03496099	0.24247301
C	1.73552096	-3.81430101	2.27734303
H	2.38848090	-3.44287896	1.49960005
C	0.50886202	-3.17767501	2.47076988
C	-4.67302895	-1.30234098	-0.34529999
H	-4.06117582	-2.16499496	-0.57168698
C	-4.06711197	-0.09590300	-0.00359700
C	-2.38573408	-3.15168190	4.70598602
H	-2.83029795	-4.15501690	4.70893812
H	-1.77031302	-3.03109598	5.60261488
C	-4.23342514	2.63893890	1.87393296
H	-5.24114323	2.63428092	2.31163406
H	-3.61696792	1.89917505	2.39160490
C	-3.60917091	4.02282810	1.96066201
H	-4.20272398	4.74056911	1.38864696
H	-2.61406708	3.97948194	1.51180804
C	-3.47088194	-2.08571291	4.67244101
H	-4.06140423	-2.19487309	3.75750089
H	-4.14153719	-2.24927092	5.52278614
C	-3.49635506	4.48912001	3.40453196
H	-2.93263602	3.76080108	4.00640917
H	-4.49456406	4.59200621	3.85692501
C	-2.90760708	-0.67240399	4.73601294
H	-3.72515893	0.06199100	4.66099024
H	-2.21984100	-0.50460398	3.89910412
C	-2.74942207	6.28100777	4.75232506
H	-2.19155407	5.61393881	5.42419291
H	-2.22845602	7.23696804	4.68619108
H	-3.74798107	6.44841290	5.17865705
C	-1.47626102	0.67671698	6.04254103
H	-2.12672997	1.56388998	5.99097919
H	-0.95848602	0.67866802	7.00299501
H	-0.74010301	0.73732501	5.23150492
O	3.29041004	-5.56717300	2.97801495
C	2.10790205	-4.88689089	3.08963490
O	4.72868013	-6.90597296	-0.04584400
C	-0.00776300	-4.70751905	4.25457621
H	-0.67737699	-5.07598686	5.02032614
C	1.22485101	-5.32631302	4.07755518
C	4.31770515	-5.01389503	2.14665794
H	4.67052794	-4.07154512	2.58158493
H	3.93437791	-4.80863810	1.14568400
C	5.44275904	-6.03323412	2.05847812
H	5.76066017	-6.31716394	3.06662512
H	6.29718924	-5.56227589	1.56195700
C	5.04818106	-7.28445292	1.28527701
H	5.87489080	-8.01134777	1.28515899
H	4.17885113	-7.76014519	1.76005602
C	4.20499611	-7.96446896	-0.82324898
H	4.95301294	-8.75441265	-0.98688400
H	3.90661502	-7.53878880	-1.78153205
H	3.32411504	-8.41461563	-0.34358200
O	-6.53739882	-2.66221809	-0.72925502
O	-9.77193928	-5.99873781	-1.72647202

C	-6.06279087	-1.42238104	-0.40729100
C	-6.25182819	0.91345501	0.14974999
H	-6.85772896	1.79787898	0.30999601
C	-6.85934591	-0.30192301	-0.16126101
C	-7.94501400	-2.84897900	-0.84838098
H	-8.34739017	-2.17744708	-1.61873102
H	-8.43666267	-2.60639000	0.10346400
C	-8.17985439	-4.30389309	-1.22261405
H	-7.66132593	-4.52423477	-2.15920091
H	-7.74998903	-4.94946289	-0.45245501
C	-9.65766525	-4.63146687	-1.38108301
H	-10.20266819	-4.43019390	-0.44391099
H	-10.11262608	-4.00298214	-2.16449499
C	-11.11104202	-6.41291094	-1.90093303
H	-11.69868946	-6.28500080	-0.97998297
H	-11.09001446	-7.47115803	-2.16248989
H	-11.60609913	-5.85558605	-2.70970201
H	7.93735695	0.35454699	0.22118600
H	1.51798797	-6.16136885	4.70246220
H	-7.93795395	-0.35454300	-0.22058401
H	-1.51700795	6.16198492	-4.70198822
Cu	0.03393300	-0.79739201	-1.05286705
Cu	-0.03441600	0.79685098	1.05257297
N	0.05496500	2.51028204	2.66744709
N	-0.05549800	-2.51030397	-2.66672897
C	0.17135400	3.64082599	2.47907209
C	-0.17174000	-3.64141297	-2.48177004
C	-0.28770700	-5.06223822	-2.20528603
H	-0.42371300	-5.20000505	-1.13023102
H	-1.14543402	-5.48397207	-2.73278403
H	0.62928998	-5.56727123	-2.52076888
C	0.28764001	5.06074190	2.19811893
H	1.14713395	5.48338699	2.72199702
H	0.42082500	5.19508410	1.12227499
H	-0.62810898	5.56741524	2.51456189

S6.4.2. Frequencies

Mode	IR frequency	IR intensity	Raman intensity
1	4.68520000	1.29950000	0.00000000
2	6.86330000	0.00000000	0.00000000
3	8.30110000	0.42440000	0.00000000
4	8.93250000	3.82540000	0.00000000
5	11.20580000	0.00000000	0.00000000
6	12.03460000	4.63140000	0.00000000
7	13.23090000	0.00030000	0.00000000
8	13.90290000	1.19490000	0.00000000
9	15.80350000	0.42020000	0.00000000
10	17.16850000	0.00010000	0.00000000
11	19.31830000	0.31620000	0.00000000
12	19.71180000	0.00010000	0.00000000
13	21.48120000	0.00300000	0.00000000
14	21.89230000	0.51650000	0.00000000
15	24.07950000	0.15420000	0.00000000
16	25.76960000	0.00240000	0.00000000
17	26.48360000	3.76400000	0.00000000
18	28.47070000	0.00000000	0.00000000
19	29.55660000	2.67760000	0.00000000
20	32.15080000	0.00000000	0.00000000
21	32.80830000	0.00010000	0.00000000
22	33.72790000	1.49060000	0.00000000
23	38.07980000	1.18250000	0.00000000
24	40.99120000	4.29350000	0.00000000
25	41.40630000	0.00000000	0.00000000
26	42.79570000	0.00000000	0.00000000
27	45.07190000	1.61470000	0.00000000
28	45.15730000	0.00040000	0.00000000
29	52.55950000	0.00000000	0.00000000
30	53.34860000	0.15660000	0.00000000
31	54.64320000	0.00000000	0.00000000
32	55.30810000	2.34460000	0.00000000
33	56.06000000	3.82970000	0.00000000
34	58.10380000	1.69020000	0.00000000
35	58.20220000	0.00800000	0.00000000
36	61.90460000	3.25290000	0.00000000
37	62.58830000	0.00160000	0.00000000
38	63.42990000	0.00010000	0.00000000
39	69.63670000	2.06890000	0.00000000
40	73.08120000	0.00000000	0.00000000
41	74.05490000	2.49250000	0.00000000
42	75.54820000	0.59840000	0.00000000
43	77.92630000	0.00000000	0.00000000
44	80.59410000	0.73630000	0.00000000
45	82.33490000	0.00000000	0.00000000
46	83.66780000	0.00000000	0.00000000
47	86.11290000	0.00170000	0.00000000
48	86.24320000	1.08440000	0.00000000
49	89.18980000	1.47960000	0.00000000
50	91.05220000	0.00000000	0.00000000
51	94.97820000	5.47200000	0.00000000
52	95.97870000	0.00520000	0.00000000
53	97.86300000	0.00070000	0.00000000
54	99.85090000	26.50960000	0.00000000
55	100.86260000	0.00010000	0.00000000
56	102.27500000	8.57900000	0.00000000
57	103.85710000	6.21320000	0.00000000
58	103.98170000	0.01730000	0.00000000
59	104.77940000	0.00280000	0.00000000
60	106.16310000	9.53790000	0.00000000

61	108.37170000	0.00070000	0.00000000
62	115.99660000	9.19380000	0.00000000
63	116.82810000	0.00250000	0.00000000
64	120.52220000	0.00000000	0.00000000
65	124.92620000	0.31140000	0.00000000
66	124.99360000	13.95450000	0.00000000
67	131.07100000	0.21740000	0.00000000
68	135.10100000	10.11820000	0.00000000
69	135.23840000	0.17700000	0.00000000
70	136.93030000	0.00090000	0.00000000
71	138.32660000	0.13750000	0.00000000
72	139.62410000	0.00010000	0.00000000
73	141.77470000	0.01940000	0.00000000
74	141.84700000	3.57950000	0.00000000
75	145.12160000	4.66380000	0.00000000
76	145.42560000	0.00000000	0.00000000
77	153.01540000	10.86700000	0.00000000
78	155.19200000	0.00000000	0.00000000
79	160.40190000	0.49240000	0.00000000
80	164.39060000	0.00050000	0.00000000
81	165.66490000	3.29740000	0.00000000
82	170.12850000	0.00000000	0.00000000
83	170.55990000	1.34680000	0.00000000
84	172.42230000	0.00000000	0.00000000
85	173.93620000	1.52290000	0.00000000
86	178.12710000	0.00000000	0.00000000
87	181.78770000	0.00010000	0.00000000
88	182.61600000	8.78680000	0.00000000
89	186.82620000	0.00010000	0.00000000
90	193.39680000	5.43770000	0.00000000
91	195.27950000	0.00010000	0.00000000
92	197.19420000	0.00000000	0.00000000
93	200.34040000	4.77130000	0.00000000
94	203.87200000	16.55600000	0.00000000
95	205.02690000	0.00000000	0.00000000
96	205.39310000	0.00010000	0.00000000
97	208.47720000	0.79500000	0.00000000
98	215.46360000	2.38210000	0.00000000
99	220.48130000	4.24310000	0.00000000
100	223.22320000	0.00010000	0.00000000
101	225.89430000	0.00000000	0.00000000
102	230.59730000	4.78200000	0.00000000
103	230.79880000	0.00070000	0.00000000
104	232.86930000	0.00500000	0.00000000
105	233.17160000	7.33060000	0.00000000
106	235.18610000	8.18300000	0.00000000
107	235.59150000	0.00060000	0.00000000
108	241.01690000	3.49630000	0.00000000
109	242.38870000	0.00010000	0.00000000
110	246.73060000	20.85580000	0.00000000
111	247.96550000	0.00010000	0.00000000
112	248.47010000	0.00050000	0.00000000
113	254.22280000	24.10740000	0.00000000
114	256.39000000	0.00470000	0.00000000
115	257.66520000	25.57570000	0.00000000
116	267.62930000	3.72230000	0.00000000
117	270.25820000	0.00010000	0.00000000
118	279.33020000	16.51790000	0.00000000
119	300.53760000	0.00060000	0.00000000
120	302.07090000	4.96950000	0.00000000
121	308.38870000	0.00010000	0.00000000
122	309.21280000	2.02400000	0.00000000
123	324.94200000	0.00000000	0.00000000
124	327.12410000	3.93540000	0.00000000

125	334.28900000	0.00000000	0.00000000
126	343.59700000	5.06960000	0.00000000
127	357.83550000	13.84730000	0.00000000
128	360.15220000	0.00000000	0.00000000
129	368.51770000	1.65940000	0.00000000
130	370.40960000	0.00000000	0.00000000
131	377.56950000	0.80230000	0.00000000
132	379.33870000	0.00000000	0.00000000
133	390.66530000	0.00010000	0.00000000
134	393.10300000	2.39230000	0.00000000
135	393.20070000	0.12170000	0.00000000
136	394.79920000	13.40960000	0.00000000
137	400.34670000	0.05970000	0.00000000
138	400.46400000	0.55450000	0.00000000
139	405.50870000	9.22730000	0.00000000
140	411.30500000	0.00000000	0.00000000
141	421.49730000	15.63140000	0.00000000
142	433.22390000	0.00000000	0.00000000
143	438.78450000	9.60390000	0.00000000
144	440.55400000	0.00010000	0.00000000
145	441.08890000	3.87100000	0.00000000
146	442.96090000	0.00010000	0.00000000
147	443.41730000	2.60450000	0.00000000
148	446.64970000	0.00010000	0.00000000
149	451.72740000	0.00010000	0.00000000
150	453.73450000	11.47090000	0.00000000
151	458.78550000	0.00000000	0.00000000
152	464.41830000	12.96520000	0.00000000
153	474.97220000	0.00000000	0.00000000
154	480.93140000	0.00040000	0.00000000
155	482.42700000	33.94870000	0.00000000
156	484.63820000	23.38600000	0.00000000
157	500.85230000	0.41730000	0.00000000
158	505.78790000	0.00000000	0.00000000
159	509.87790000	0.00010000	0.00000000
160	511.91580000	7.77930000	0.00000000
161	513.49910000	36.46580000	0.00000000
162	518.43860000	0.00000000	0.00000000
163	540.03280000	0.71340000	0.00000000
164	540.22960000	0.00010000	0.00000000
165	552.33130000	0.57830000	0.00000000
166	552.38210000	7.66360000	0.00000000
167	577.73380000	23.44710000	0.00000000
168	578.44140000	0.00380000	0.00000000
169	612.91220000	2.76130000	0.00000000
170	613.82550000	0.00000000	0.00000000
171	622.63990000	8.67250000	0.00000000
172	623.09600000	0.00700000	0.00000000
173	629.96900000	10.33580000	0.00000000
174	635.01050000	0.00030000	0.00000000
175	641.96250000	0.10440000	0.00000000
176	644.09890000	0.00000000	0.00000000
177	687.54940000	15.95340000	0.00000000
178	688.63440000	0.00000000	0.00000000
179	696.98650000	17.11020000	0.00000000
180	698.86400000	0.00040000	0.00000000
181	727.09670000	0.03940000	0.00000000
182	727.84430000	43.01170000	0.00000000
183	729.71320000	11.02310000	0.00000000
184	730.55710000	0.00130000	0.00000000
185	762.44860000	91.21220000	0.00000000
186	763.12150000	0.00390000	0.00000000
187	785.77640000	14.27550000	0.00000000
188	786.10690000	0.00460000	0.00000000

189	791.80890000	0.00420000	0.00000000
190	791.95260000	4.84750000	0.00000000
191	792.34090000	0.00220000	0.00000000
192	792.59060000	26.72700000	0.00000000
193	796.33260000	0.00000000	0.00000000
194	797.26190000	46.48460000	0.00000000
195	799.26830000	13.94760000	0.00000000
196	800.07190000	0.00030000	0.00000000
197	807.75610000	41.76140000	0.00000000
198	809.36800000	0.00060000	0.00000000
199	823.29340000	50.17770000	0.00000000
200	823.79130000	0.00220000	0.00000000
201	827.12470000	46.36840000	0.00000000
202	828.35030000	0.00010000	0.00000000
203	831.34180000	24.14560000	0.00000000
204	835.59030000	0.00010000	0.00000000
205	840.03860000	11.52450000	0.00000000
206	841.87180000	0.00020000	0.00000000
207	844.00400000	53.96290000	0.00000000
208	846.15980000	0.00030000	0.00000000
209	896.65760000	3.74840000	0.00000000
210	896.76060000	0.00000000	0.00000000
211	897.70550000	0.00000000	0.00000000
212	897.83530000	3.57720000	0.00000000
213	901.09030000	0.17380000	0.00000000
214	901.15150000	0.00140000	0.00000000
215	902.92670000	3.44230000	0.00000000
216	902.94150000	4.43420000	0.00000000
217	905.52160000	4.50430000	0.00000000
218	905.54530000	11.87530000	0.00000000
219	905.94570000	17.67360000	0.00000000
220	905.98030000	1.90810000	0.00000000
221	934.84630000	0.33130000	0.00000000
222	934.89130000	2.43410000	0.00000000
223	935.27490000	15.10420000	0.00000000
224	935.29930000	2.02190000	0.00000000
225	938.41380000	8.68080000	0.00000000
226	939.11640000	0.00530000	0.00000000
227	939.17800000	29.57800000	0.00000000
228	939.26590000	0.00270000	0.00000000
229	944.32740000	25.07710000	0.00000000
230	944.34190000	0.00010000	0.00000000
231	946.38880000	12.61060000	0.00000000
232	946.46400000	0.13260000	0.00000000
233	949.34350000	10.80960000	0.00000000
234	950.52800000	0.00000000	0.00000000
235	956.35190000	0.23650000	0.00000000
236	956.41860000	37.88610000	0.00000000
237	974.31760000	35.99190000	0.00000000
238	974.40590000	0.00020000	0.00000000
239	996.72160000	0.01090000	0.00000000
240	996.79980000	54.33530000	0.00000000
241	1007.15170000	197.44900000	0.00000000
242	1007.43510000	0.00930000	0.00000000
243	1026.35470000	1.30470000	0.00000000
244	1026.51900000	215.52240000	0.00000000
245	1057.87800000	220.60880000	0.00000000
246	1058.00950000	0.01070000	0.00000000
247	1061.30310000	0.05810000	0.00000000
248	1061.35960000	37.46950000	0.00000000
249	1063.83700000	1.10990000	0.00000000
250	1063.87670000	59.66530000	0.00000000
251	1064.52970000	3.34600000	0.00000000
252	1064.55640000	152.43800000	0.00000000

253	1064.95110000	4.87790000	0.00000000
254	1065.00760000	1.84470000	0.00000000
255	1068.28890000	182.73060000	0.00000000
256	1068.35250000	0.00070000	0.00000000
257	1078.93210000	0.00390000	0.00000000
258	1078.97480000	11.52640000	0.00000000
259	1094.80720000	26.74660000	0.00000000
260	1094.81550000	5.25490000	0.00000000
261	1101.08320000	13.06840000	0.00000000
262	1101.09310000	0.00480000	0.00000000
263	1101.55290000	33.02940000	0.00000000
264	1101.75640000	0.00180000	0.00000000
265	1111.55340000	0.13710000	0.00000000
266	1111.57740000	80.64200000	0.00000000
267	1111.97360000	249.92430000	0.00000000
268	1113.07860000	0.00020000	0.00000000
269	1116.51340000	19.45990000	0.00000000
270	1116.69250000	0.00010000	0.00000000
271	1118.57190000	10.46860000	0.00000000
272	1118.78980000	0.00000000	0.00000000
273	1123.10470000	107.08040000	0.00000000
274	1123.67540000	0.00010000	0.00000000
275	1135.71260000	337.35730000	0.00000000
276	1135.79860000	1.73920000	0.00000000
277	1149.34350000	0.00650000	0.00000000
278	1149.60370000	33.29620000	0.00000000
279	1149.94350000	457.30540000	0.00000000
280	1150.01510000	0.01850000	0.00000000
281	1152.61470000	445.04730000	0.00000000
282	1152.77060000	0.00230000	0.00000000
283	1161.72340000	0.01960000	0.00000000
284	1161.72970000	2.56780000	0.00000000
285	1163.96430000	0.25810000	0.00000000
286	1163.96550000	0.50800000	0.00000000
287	1165.11870000	0.01560000	0.00000000
288	1165.17760000	31.73420000	0.00000000
289	1168.38360000	14.06080000	0.00000000
290	1168.48390000	0.00000000	0.00000000
291	1177.57090000	8.96480000	0.00000000
292	1177.57880000	1.26280000	0.00000000
293	1181.39100000	0.81330000	0.00000000
294	1181.39630000	6.93290000	0.00000000
295	1184.10990000	0.10580000	0.00000000
296	1184.11590000	10.86220000	0.00000000
297	1186.85110000	0.75880000	0.00000000
298	1186.85260000	16.46780000	0.00000000
299	1215.48550000	54.51550000	0.00000000
300	1215.52110000	0.07410000	0.00000000
301	1217.27760000	0.01770000	0.00000000
302	1217.35940000	34.23970000	0.00000000
303	1219.02030000	11.18490000	0.00000000
304	1219.06600000	0.04720000	0.00000000
305	1223.09920000	54.83040000	0.00000000
306	1223.10310000	0.88640000	0.00000000
307	1229.59760000	0.21030000	0.00000000
308	1229.68240000	469.80440000	0.00000000
309	1237.32080000	1.11750000	0.00000000
310	1237.32140000	0.96860000	0.00000000
311	1242.77900000	1.10680000	0.00000000
312	1242.81050000	0.00670000	0.00000000
313	1247.86830000	0.01030000	0.00000000
314	1248.59660000	864.83680000	0.00000000
315	1255.72210000	11.25190000	0.00000000
316	1255.74560000	0.00030000	0.00000000

317	1259.72240000	0.38370000	0.00000000
318	1259.77920000	48.80160000	0.00000000
319	1262.51850000	3.26290000	0.00000000
320	1262.52720000	0.00360000	0.00000000
321	1265.77100000	23.83960000	0.00000000
322	1265.77920000	8.76650000	0.00000000
323	1272.33190000	31.64490000	0.00000000
324	1272.42770000	0.00470000	0.00000000
325	1289.04340000	266.79880000	0.00000000
326	1289.30250000	0.00230000	0.00000000
327	1296.08970000	62.93730000	0.00000000
328	1296.40420000	0.00920000	0.00000000
329	1305.71590000	60.51350000	0.00000000
330	1305.73660000	0.06470000	0.00000000
331	1305.88900000	22.85390000	0.00000000
332	1306.30010000	0.00190000	0.00000000
333	1309.27280000	0.11660000	0.00000000
334	1309.27530000	0.51400000	0.00000000
335	1311.57440000	7.58690000	0.00000000
336	1311.63360000	0.17630000	0.00000000
337	1314.42290000	0.78870000	0.00000000
338	1314.42380000	0.09960000	0.00000000
339	1317.62090000	14.96300000	0.00000000
340	1317.64000000	0.09590000	0.00000000
341	1318.05950000	0.00030000	0.00000000
342	1318.18260000	41.56440000	0.00000000
343	1321.73360000	152.27790000	0.00000000
344	1321.95210000	0.02330000	0.00000000
345	1323.98250000	0.00030000	0.00000000
346	1324.12720000	83.86380000	0.00000000
347	1326.55230000	0.00060000	0.00000000
348	1326.57320000	9.11270000	0.00000000
349	1328.64570000	19.35520000	0.00000000
350	1328.80110000	0.00160000	0.00000000
351	1382.04430000	0.00060000	0.00000000
352	1386.12540000	24.55700000	0.00000000
353	1386.29310000	0.00600000	0.00000000
354	1387.76450000	22.19990000	0.00000000
355	1388.55050000	0.00060000	0.00000000
356	1390.29780000	366.92770000	0.00000000
357	1395.99320000	174.27460000	0.00000000
358	1400.51440000	0.00060000	0.00000000
359	1410.06640000	8.25170000	0.00000000
360	1410.16010000	12.94900000	0.00000000
361	1413.64960000	0.07180000	0.00000000
362	1413.67730000	36.52160000	0.00000000
363	1417.07840000	12.07120000	0.00000000
364	1417.08870000	0.07170000	0.00000000
365	1422.16170000	60.79360000	0.00000000
366	1422.31610000	0.00000000	0.00000000
367	1425.56620000	3.19270000	0.00000000
368	1425.57520000	0.12730000	0.00000000
369	1432.26580000	0.00100000	0.00000000
370	1432.29900000	60.88390000	0.00000000
371	1436.22660000	97.65970000	0.00000000
372	1436.24930000	3.68800000	0.00000000
373	1436.93020000	338.33180000	0.00000000
374	1437.12990000	0.00610000	0.00000000
375	1441.67540000	15.27900000	0.00000000
376	1441.69520000	0.06250000	0.00000000
377	1443.83390000	78.82660000	0.00000000
378	1444.48230000	0.01340000	0.00000000
379	1451.81730000	49.02260000	0.00000000
380	1452.43640000	0.00080000	0.00000000

381	1464.35640000	16.60140000	0.00000000
382	1464.36390000	2.02870000	0.00000000
383	1464.92570000	13.13900000	0.00000000
384	1464.94200000	0.17130000	0.00000000
385	1471.11890000	4.25180000	0.00000000
386	1471.16410000	0.05700000	0.00000000
387	1479.54270000	2.87540000	0.00000000
388	1479.54880000	0.01830000	0.00000000
389	1480.61100000	0.05920000	0.00000000
390	1480.63090000	2.49450000	0.00000000
391	1480.74090000	0.10710000	0.00000000
392	1480.74220000	0.08470000	0.00000000
393	1483.85870000	5.86390000	0.00000000
394	1483.86000000	9.16070000	0.00000000
395	1483.88370000	5.56230000	0.00000000
396	1483.90610000	0.15760000	0.00000000
397	1484.39480000	6.39580000	0.00000000
398	1484.44680000	0.11100000	0.00000000
399	1486.04900000	8.00550000	0.00000000
400	1486.06110000	0.36740000	0.00000000
401	1488.20480000	38.63030000	0.00000000
402	1488.43050000	0.09130000	0.00000000
403	1490.39560000	263.30900000	0.00000000
404	1493.48130000	0.36180000	0.00000000
405	1493.49990000	6.31390000	0.00000000
406	1502.14940000	6.89130000	0.00000000
407	1502.15320000	0.00050000	0.00000000
408	1503.67000000	104.07050000	0.00000000
409	1504.16330000	0.00030000	0.00000000
410	1504.55400000	0.00600000	0.00000000
411	1504.84670000	8.84130000	0.00000000
412	1505.25330000	0.00150000	0.00000000
413	1505.46760000	138.27730000	0.00000000
414	1505.58750000	61.72130000	0.00000000
415	1505.66590000	0.02890000	0.00000000
416	1506.58910000	97.09220000	0.00000000
417	1506.67660000	0.01260000	0.00000000
418	1507.41080000	146.98020000	0.00000000
419	1507.85880000	0.00030000	0.00000000
420	1512.69280000	20.61520000	0.00000000
421	1512.84420000	0.00200000	0.00000000
422	1513.32480000	81.65380000	0.00000000
423	1513.63260000	0.00230000	0.00000000
424	1519.38720000	131.87560000	0.00000000
425	1519.59960000	0.00710000	0.00000000
426	1523.24680000	19.71510000	0.00000000
427	1526.44700000	0.00000000	0.00000000
428	1527.19590000	18.79820000	0.00000000
429	1527.24900000	0.01690000	0.00000000
430	1527.65990000	48.86930000	0.00000000
431	1528.23480000	0.00150000	0.00000000
432	1531.32280000	96.63300000	0.00000000
433	1533.99340000	0.00030000	0.00000000
434	1534.22590000	14.55070000	0.00000000
435	1535.73750000	0.00000000	0.00000000
436	1536.02490000	11.89010000	0.00000000
437	1538.04490000	0.00060000	0.00000000
438	1539.87290000	300.66190000	0.00000000
439	1548.47310000	0.00030000	0.00000000
440	1561.72610000	0.00030000	0.00000000
441	1606.67160000	89.12860000	0.00000000
442	1606.93330000	0.02100000	0.00000000
443	1620.98680000	10.00540000	0.00000000
444	1621.22210000	0.00760000	0.00000000

445	1648.92560000	53.51150000	0.00000000
446	1649.21940000	95.05170000	0.00000000
447	1649.47950000	0.00360000	0.00000000
448	1650.63770000	0.00000000	0.00000000
449	2362.97830000	37.09130000	0.00000000
450	2363.10220000	20.71170000	0.00000000
451	2937.01950000	0.26050000	0.00000000
452	2937.02290000	69.61710000	0.00000000
453	2952.04800000	2.36230000	0.00000000
454	2952.06290000	43.05680000	0.00000000
455	2957.40960000	13.48260000	0.00000000
456	2957.41480000	40.52740000	0.00000000
457	2958.48800000	0.00150000	0.00000000
458	2958.51860000	75.71890000	0.00000000
459	2960.59040000	0.16220000	0.00000000
460	2960.59530000	111.01730000	0.00000000
461	2960.89030000	0.11920000	0.00000000
462	2960.98970000	259.71790000	0.00000000
463	2963.99260000	179.81770000	0.00000000
464	2964.00800000	0.01830000	0.00000000
465	2967.52830000	303.57580000	0.00000000
466	2967.58280000	0.18540000	0.00000000
467	2975.52480000	162.32160000	0.00000000
468	2975.54600000	5.34350000	0.00000000
469	2980.22380000	99.92800000	0.00000000
470	2980.24640000	0.32210000	0.00000000
471	2986.69810000	77.02270000	0.00000000
472	2986.73910000	26.43390000	0.00000000
473	2990.45040000	108.82000000	0.00000000
474	2990.50420000	21.67260000	0.00000000
475	2996.79930000	103.65280000	0.00000000
476	2996.84540000	0.87660000	0.00000000
477	3003.06770000	3.26790000	0.00000000
478	3003.07210000	158.22090000	0.00000000
479	3004.18590000	0.24480000	0.00000000
480	3004.19000000	131.67100000	0.00000000
481	3006.08400000	28.12650000	0.00000000
482	3006.09930000	16.84490000	0.00000000
483	3014.52620000	3.00830000	0.00000000
484	3014.53580000	64.96210000	0.00000000
485	3017.20620000	0.00350000	0.00000000
486	3017.21600000	43.05870000	0.00000000
487	3019.73750000	71.12510000	0.00000000
488	3019.75310000	49.01920000	0.00000000
489	3026.54760000	32.87030000	0.00000000
490	3026.61340000	34.71160000	0.00000000
491	3031.39730000	28.26110000	0.00000000
492	3031.40030000	5.51450000	0.00000000
493	3034.30870000	9.41120000	0.00000000
494	3034.36710000	11.69030000	0.00000000
495	3039.23040000	41.87910000	0.00000000
496	3039.23570000	44.14250000	0.00000000
497	3041.88600000	52.33470000	0.00000000
498	3041.90310000	0.00010000	0.00000000
499	3051.68950000	0.71320000	0.00000000
500	3051.69210000	30.87620000	0.00000000
501	3054.55380000	6.62960000	0.00000000
502	3054.57050000	9.61930000	0.00000000
503	3059.73930000	16.50080000	0.00000000
504	3059.74360000	21.36460000	0.00000000
505	3068.10180000	8.39870000	0.00000000
506	3068.13740000	18.55950000	0.00000000
507	3075.54080000	5.82510000	0.00000000
508	3075.54880000	33.62190000	0.00000000

509	3079.78200000	76.59040000	0.00000000
510	3079.79500000	1.83180000	0.00000000
511	3096.29830000	1.44270000	0.00000000
512	3096.30190000	44.11760000	0.00000000
513	3096.49450000	23.90500000	0.00000000
514	3096.50650000	37.60830000	0.00000000
515	3102.76360000	84.16960000	0.00000000
516	3102.78350000	0.00150000	0.00000000
517	3105.06270000	21.63120000	0.00000000
518	3105.08210000	8.42760000	0.00000000
519	3108.56240000	0.45640000	0.00000000
520	3108.57080000	53.33420000	0.00000000
521	3109.13520000	21.13660000	0.00000000
522	3109.20110000	12.36550000	0.00000000
523	3112.14180000	53.59240000	0.00000000
524	3112.14340000	11.92290000	0.00000000
525	3117.26650000	6.56770000	0.00000000
526	3117.29990000	6.90340000	0.00000000
527	3118.55700000	1.83310000	0.00000000
528	3118.56770000	0.98140000	0.00000000
529	3178.42740000	13.00680000	0.00000000
530	3178.43590000	7.64080000	0.00000000
531	3183.35200000	8.87530000	0.00000000
532	3183.35770000	9.58810000	0.00000000
533	3203.54290000	19.57210000	0.00000000
534	3203.55860000	0.12740000	0.00000000
535	3205.57080000	27.77700000	0.00000000
536	3205.58850000	0.25850000	0.00000000
537	3209.50120000	4.74350000	0.00000000
538	3209.57880000	3.77860000	0.00000000
539	3212.97040000	4.31230000	0.00000000
540	3212.99870000	0.72060000	0.00000000

S6.5. Calculations on $Cu_2(\mathbf{1}^*)_4$

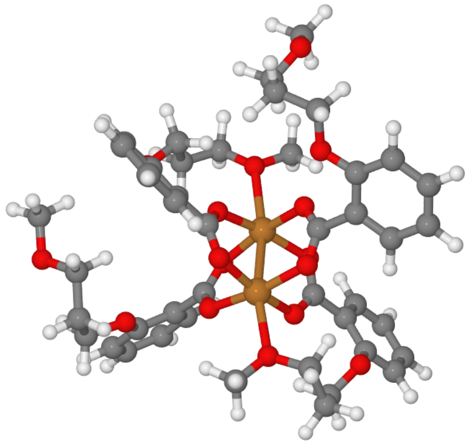

Route	: # opt freq b3lyp/genecp geom=connectivity int=ultrafine
SMILES	: COCCCOc1ccccc1[C]2O[Cu]3456O[C](c7ccccc7OCCCO3C)O[Cu]48(O2)(O[C](O5)c9ccccc9OCCCO8C)O[C](O6)c1ccccc1OCCCOC
Formula	: $C_{44}H_{52}Cu_2O_{16}$
Charge	: 0
Multiplicity	: 1
Dipole	: 1.2650 Debye
Energy	: -3307.13961972 a.u.
Gibbs Energy	: -3306.34228200 a.u.
Number of imaginary frequencies	: 0

S6.5.1. Cartesian Co-ordinates (XYZ format)

114

O	-0.91716200	1.36776495	1.62888801
O	-1.27338600	1.96108198	-0.52104998
O	1.91250396	1.20508695	1.71583796
O	1.48537004	2.32534194	-0.18828499
C	-1.50583196	2.01939607	0.71579301
C	2.09734201	2.15132499	0.89894497
O	-4.01742983	2.44563293	-0.57269400
C	-3.75729895	3.22430301	0.50343698
O	-6.63180494	0.00761300	-0.85201502
C	3.81249189	3.93691397	0.27968100
C	-2.26922607	3.73345494	2.34704900
H	-1.34455502	3.52849102	2.87118506
C	-2.54356408	3.00186396	1.19047904
C	3.42504096	3.39913511	2.60841799
H	2.90663290	2.79723692	3.34369898
C	3.12686896	3.18580604	1.25724101
C	-5.27686882	2.56835008	-1.24462605
H	-5.33821678	3.54825306	-1.73285794
H	-6.09592485	2.47440791	-0.52573401

C	-5.36317921	1.44663703	-2.26764393
H	-4.49332905	1.48774898	-2.92971110
H	-6.25676584	1.61019897	-2.87908602
C	-5.44491291	0.06649400	-1.63018596
H	-5.46151400	-0.70970201	-2.40961003
H	-4.56620407	-0.11248600	-0.99763602
C	-6.75399780	-1.20170200	-0.12716299
H	-6.83746386	-2.06682110	-0.80123502
H	-7.66529417	-1.12914503	0.46818000
H	-5.89578009	-1.36346102	0.53789699
C	-3.14489388	4.70921898	2.81275201
C	-4.63983107	4.20146179	0.98128802
H	-5.57333183	4.38639879	0.46893200
C	-4.32836390	4.93927002	2.12058902
C	4.35522413	4.35355902	2.99953508
C	4.76127100	4.87961388	0.68234998
H	5.29650402	5.42358017	-0.08721000
C	5.02795315	5.09478712	2.02987194
O	1.84623599	-1.28328097	0.44240499
O	-0.97098899	-1.13951194	0.34095299
O	1.98327899	-0.11531200	-1.48541296
O	-0.81118900	-0.52829301	-1.81649804
C	2.35503697	-1.01510894	-0.67715299
C	-1.24893999	-1.25369799	-0.88468701
O	2.44418502	-3.83638406	-0.75800598
O	-3.26600194	-2.50468802	0.93974501
O	0.12377700	-1.46995604	3.12828302
C	3.59303594	-3.20409608	-1.10652006
O	1.76338005	-6.28203011	1.85738397
C	-3.04292989	-2.99864411	-0.31908301
C	4.70450687	-1.08949006	-1.50578904
H	4.66277409	-0.00715800	-1.51482105
C	3.57228804	-1.79528105	-1.10297596
C	-2.10416698	-2.89772391	-2.55094504
H	-1.43248701	-2.41426206	-3.24861503
C	-2.15850997	-2.39697599	-1.24388003
C	2.37700105	-5.26456213	-0.83620203
H	2.51852703	-5.58122206	-1.87642598
H	3.15952110	-5.71405506	-0.21742900
C	-2.33847499	-2.85452509	1.99423695
H	-2.69804406	-3.77393603	2.47396588
H	-1.35171497	-3.03005290	1.57228196
C	-2.28945589	-1.69225097	2.98312402
H	-3.18303204	-1.69833302	3.61614490
H	-2.29804611	-0.76001900	2.41899300
C	1.00929403	-5.68627596	-0.32306701
H	0.23297900	-5.14378691	-0.87015599
H	0.87745500	-6.75365019	-0.52786797
C	-1.06594098	-1.70719397	3.88888192
H	-0.96395898	-2.67495799	4.40359497
H	-1.16523302	-0.93059999	4.65905714
C	0.83749402	-5.44953108	1.17046702
H	-0.18996400	-5.69828796	1.47633302
H	1.01897395	-4.39334822	1.41096401
C	1.30690706	-1.56249106	3.91316509
H	1.38262200	-2.55116296	4.38369894
H	2.14985800	-1.41617596	3.24000311
H	1.32387602	-0.79269701	4.69514084
C	1.76242304	-6.07801294	3.25359201
H	0.77829701	-6.29638386	3.69473410
H	2.49849200	-6.75928879	3.68231797
H	2.03875995	-5.04565811	3.51147509
C	5.87218094	-1.75272596	-1.87479401
C	4.76057482	-3.86798692	-1.49946296

H	4.79298782	-4.94836283	-1.51715302
C	5.89133692	-3.14240599	-1.86996102
C	-2.88102889	-3.98005199	-2.94530797
C	-3.82969809	-4.07748222	-0.73166299
H	-4.51647520	-4.50662899	-0.01165300
C	-3.74862695	-4.57132721	-2.02910995
H	5.76687479	5.83320189	2.32073903
H	6.78809690	-3.67714405	-2.16296506
H	-4.36695814	-5.41190577	-2.32424903
H	-5.02625895	5.69367123	2.46697497
H	-2.90510988	5.27735376	3.70340991
H	-2.81557894	-4.35346317	-3.96053290
H	6.74903679	-1.18850005	-2.16854692
H	4.56361294	4.50909281	4.05158806
Cu	0.35078001	1.00359797	-1.18248999
Cu	0.43413499	-0.04983300	1.19499505
O	3.65927196	3.71703506	-1.06690001
C	2.65915704	4.51126385	-1.73839998
H	1.69683695	4.37552309	-1.24309003
H	2.94995189	5.56793690	-1.68702102
C	2.58957195	4.05909109	-3.19015789
H	3.58878493	4.13664818	-3.63387990
H	1.95153797	4.76329899	-3.73539090
C	2.10200405	2.62239289	-3.39580607
O	0.75254601	2.40075397	-2.96352696
C	-0.25833699	2.85141301	-3.85478210
H	-0.19113600	2.32923603	-4.81781912
H	-1.21401894	2.62796688	-3.38243508
H	-0.19424599	3.93177795	-4.02671289
H	2.18735290	2.34504199	-4.45462990
H	2.70752001	1.93233097	-2.81150889

S6.5.2. Frequencies

Mode	IR frequency	IR intensity	Raman intensity
1	7.42400000	0.05270000	0.00000000
2	11.91100000	0.09340000	0.00000000
3	14.60130000	0.03590000	0.00000000
4	16.32940000	0.07430000	0.00000000
5	19.06440000	0.09800000	0.00000000
6	21.84360000	0.35880000	0.00000000
7	22.21120000	0.36000000	0.00000000
8	22.71750000	0.06510000	0.00000000
9	28.36790000	0.33230000	0.00000000
10	30.69970000	0.14450000	0.00000000
11	32.55320000	0.14270000	0.00000000
12	37.04470000	0.55210000	0.00000000
13	38.01830000	0.12790000	0.00000000
14	39.82690000	0.92000000	0.00000000
15	45.16920000	0.34550000	0.00000000
16	50.99710000	0.42920000	0.00000000
17	54.46010000	0.20720000	0.00000000
18	63.38540000	0.09520000	0.00000000
19	68.75430000	0.12190000	0.00000000
20	76.49760000	0.97650000	0.00000000
21	78.44610000	0.52060000	0.00000000
22	80.23080000	0.01660000	0.00000000
23	84.14360000	0.80230000	0.00000000
24	86.60330000	0.29670000	0.00000000
25	89.34200000	0.48840000	0.00000000
26	92.77190000	0.40030000	0.00000000
27	95.33130000	0.37820000	0.00000000
28	97.75040000	3.91190000	0.00000000
29	106.11190000	0.76880000	0.00000000
30	112.55600000	5.07620000	0.00000000
31	112.99860000	1.38530000	0.00000000
32	114.82500000	0.02760000	0.00000000
33	115.56790000	4.74610000	0.00000000
34	122.10540000	0.35670000	0.00000000
35	124.28350000	7.38040000	0.00000000
36	127.21150000	1.49100000	0.00000000
37	133.18890000	1.13270000	0.00000000
38	137.93980000	4.22020000	0.00000000
39	143.48260000	3.46820000	0.00000000
40	145.84260000	1.25830000	0.00000000
41	149.61870000	0.15890000	0.00000000
42	151.04300000	1.29910000	0.00000000
43	167.65620000	0.06920000	0.00000000
44	172.48410000	2.55800000	0.00000000
45	174.82500000	1.19150000	0.00000000
46	181.79780000	2.63390000	0.00000000
47	187.43880000	1.99790000	0.00000000
48	190.31640000	5.46500000	0.00000000
49	194.44870000	1.40630000	0.00000000
50	199.23230000	0.45390000	0.00000000
51	205.11620000	0.89170000	0.00000000
52	209.67280000	5.77800000	0.00000000
53	213.55880000	1.30140000	0.00000000
54	215.41420000	1.84530000	0.00000000
55	222.95200000	2.74130000	0.00000000
56	226.84800000	12.34410000	0.00000000
57	230.49750000	2.46800000	0.00000000
58	232.97480000	4.23840000	0.00000000
59	235.51020000	5.23820000	0.00000000
60	242.81630000	7.30720000	0.00000000

61	247.05930000	3.50040000	0.00000000
62	252.78110000	7.89010000	0.00000000
63	257.91920000	7.79550000	0.00000000
64	262.75390000	2.97960000	0.00000000
65	274.01890000	5.85230000	0.00000000
66	277.66710000	5.63480000	0.00000000
67	281.71840000	4.66150000	0.00000000
68	284.09600000	4.75940000	0.00000000
69	291.08260000	15.10150000	0.00000000
70	297.10240000	2.46570000	0.00000000
71	308.55640000	18.11530000	0.00000000
72	316.69770000	4.44780000	0.00000000
73	320.98290000	2.74590000	0.00000000
74	322.30580000	3.27980000	0.00000000
75	323.79110000	0.55510000	0.00000000
76	358.27330000	1.13390000	0.00000000
77	362.93760000	3.00480000	0.00000000
78	398.83360000	2.45580000	0.00000000
79	399.91650000	0.85020000	0.00000000
80	407.38230000	3.86870000	0.00000000
81	416.48960000	0.52120000	0.00000000
82	420.19220000	6.99590000	0.00000000
83	427.55860000	8.17070000	0.00000000
84	434.94450000	0.09330000	0.00000000
85	447.72180000	1.01450000	0.00000000
86	451.57800000	6.51250000	0.00000000
87	452.36440000	2.71510000	0.00000000
88	457.12100000	16.10860000	0.00000000
89	470.54650000	0.62970000	0.00000000
90	482.70330000	24.25740000	0.00000000
91	484.73020000	1.17750000	0.00000000
92	495.98320000	20.05640000	0.00000000
93	512.82370000	1.56760000	0.00000000
94	519.25870000	37.88220000	0.00000000
95	525.46260000	6.12820000	0.00000000
96	529.60250000	3.69950000	0.00000000
97	533.73030000	4.19930000	0.00000000
98	541.59730000	3.28350000	0.00000000
99	551.43530000	4.44350000	0.00000000
100	565.46610000	4.80180000	0.00000000
101	579.30630000	4.72830000	0.00000000
102	592.18150000	5.15460000	0.00000000
103	593.53680000	0.03840000	0.00000000
104	598.74460000	1.48980000	0.00000000
105	613.82130000	6.51180000	0.00000000
106	620.20800000	1.75720000	0.00000000
107	620.37880000	5.66800000	0.00000000
108	622.89300000	3.33690000	0.00000000
109	676.89420000	30.24920000	0.00000000
110	679.78990000	21.84710000	0.00000000
111	688.36190000	72.87380000	0.00000000
112	691.45810000	0.86260000	0.00000000
113	722.13490000	6.05640000	0.00000000
114	723.65260000	41.35390000	0.00000000
115	730.05490000	9.07990000	0.00000000
116	739.47210000	6.70930000	0.00000000
117	755.94460000	12.83620000	0.00000000
118	762.20160000	4.06030000	0.00000000
119	764.20820000	49.98320000	0.00000000
120	764.43680000	60.67560000	0.00000000
121	778.51750000	8.04260000	0.00000000
122	779.30590000	2.72380000	0.00000000
123	781.78060000	56.47670000	0.00000000
124	781.93290000	7.49380000	0.00000000

125	799.62280000	2.46850000	0.00000000
126	801.76490000	6.45920000	0.00000000
127	810.34610000	3.04630000	0.00000000
128	814.14200000	1.54310000	0.00000000
129	823.43890000	12.26450000	0.00000000
130	832.83530000	1.86750000	0.00000000
131	838.76530000	7.19650000	0.00000000
132	842.06800000	4.67810000	0.00000000
133	856.66650000	5.00890000	0.00000000
134	864.41150000	1.53890000	0.00000000
135	864.68710000	3.21480000	0.00000000
136	869.77920000	10.64310000	0.00000000
137	870.86940000	2.99020000	0.00000000
138	874.89260000	7.58880000	0.00000000
139	880.57970000	1.82560000	0.00000000
140	889.34700000	6.79720000	0.00000000
141	898.79690000	4.55470000	0.00000000
142	901.24900000	2.71380000	0.00000000
143	902.95560000	1.76610000	0.00000000
144	907.73690000	5.16720000	0.00000000
145	916.87790000	40.15700000	0.00000000
146	926.43300000	60.78090000	0.00000000
147	943.63470000	14.58450000	0.00000000
148	944.87190000	15.47990000	0.00000000
149	955.64820000	0.14140000	0.00000000
150	957.32470000	3.51000000	0.00000000
151	962.52430000	56.51410000	0.00000000
152	973.05280000	11.86970000	0.00000000
153	974.54310000	3.69140000	0.00000000
154	980.76690000	1.00340000	0.00000000
155	981.51210000	0.29090000	0.00000000
156	994.35880000	0.11540000	0.00000000
157	995.76240000	0.47640000	0.00000000
158	996.11490000	25.96510000	0.00000000
159	997.31450000	34.29810000	0.00000000
160	1005.06750000	118.19420000	0.00000000
161	1035.51080000	7.35780000	0.00000000
162	1046.39490000	6.65870000	0.00000000
163	1061.39300000	57.91310000	0.00000000
164	1061.43040000	3.75850000	0.00000000
165	1061.59430000	7.40250000	0.00000000
166	1062.69990000	51.72380000	0.00000000
167	1071.11990000	17.19360000	0.00000000
168	1072.47520000	15.90650000	0.00000000
169	1098.54620000	72.54430000	0.00000000
170	1104.77710000	7.61680000	0.00000000
171	1107.80050000	52.55700000	0.00000000
172	1108.37540000	9.16850000	0.00000000
173	1109.29170000	13.34330000	0.00000000
174	1112.05230000	5.45410000	0.00000000
175	1114.52270000	44.76000000	0.00000000
176	1118.13860000	13.78410000	0.00000000
177	1118.48370000	12.42380000	0.00000000
178	1119.23630000	5.63840000	0.00000000
179	1124.13780000	61.39830000	0.00000000
180	1126.15620000	76.13200000	0.00000000
181	1128.85040000	54.37770000	0.00000000
182	1132.01150000	67.46180000	0.00000000
183	1147.28510000	97.16730000	0.00000000
184	1150.28140000	120.17970000	0.00000000
185	1167.62920000	6.83400000	0.00000000
186	1168.41460000	6.08080000	0.00000000
187	1172.29180000	25.08190000	0.00000000
188	1172.72020000	3.49790000	0.00000000

189	1173.80810000	2.08400000	0.00000000
190	1177.47130000	3.28530000	0.00000000
191	1178.76900000	3.45440000	0.00000000
192	1180.06830000	0.95970000	0.00000000
193	1182.04040000	16.20870000	0.00000000
194	1182.50020000	2.94410000	0.00000000
195	1187.23560000	10.14540000	0.00000000
196	1187.52970000	15.12610000	0.00000000
197	1208.16250000	12.19660000	0.00000000
198	1213.15440000	28.60380000	0.00000000
199	1215.58070000	28.37300000	0.00000000
200	1218.16320000	19.11070000	0.00000000
201	1239.83480000	94.73240000	0.00000000
202	1242.98310000	54.14420000	0.00000000
203	1245.56630000	44.07780000	0.00000000
204	1254.30530000	14.11510000	0.00000000
205	1255.52510000	17.89760000	0.00000000
206	1258.48660000	18.00860000	0.00000000
207	1263.43240000	11.25780000	0.00000000
208	1264.50120000	10.83450000	0.00000000
209	1277.47900000	133.13790000	0.00000000
210	1279.49460000	189.10630000	0.00000000
211	1280.96780000	17.84330000	0.00000000
212	1281.83920000	8.81730000	0.00000000
213	1284.54310000	7.37990000	0.00000000
214	1295.76190000	5.10130000	0.00000000
215	1303.80400000	80.42300000	0.00000000
216	1305.09950000	57.08760000	0.00000000
217	1317.74670000	8.28190000	0.00000000
218	1320.28220000	3.92070000	0.00000000
219	1321.69770000	5.22940000	0.00000000
220	1323.23170000	4.72340000	0.00000000
221	1325.63420000	3.30620000	0.00000000
222	1327.35310000	45.57470000	0.00000000
223	1329.29140000	33.69900000	0.00000000
224	1338.09510000	6.16770000	0.00000000
225	1358.74510000	0.66700000	0.00000000
226	1382.15260000	67.13160000	0.00000000
227	1387.96400000	43.15670000	0.00000000
228	1388.89850000	3.68650000	0.00000000
229	1389.32650000	10.26320000	0.00000000
230	1391.89380000	236.33210000	0.00000000
231	1397.82590000	500.40420000	0.00000000
232	1403.76090000	15.74350000	0.00000000
233	1409.64600000	16.86400000	0.00000000
234	1414.45250000	6.59160000	0.00000000
235	1416.00450000	5.60720000	0.00000000
236	1419.31200000	6.12610000	0.00000000
237	1425.46760000	10.54270000	0.00000000
238	1426.59730000	18.01070000	0.00000000
239	1428.25400000	13.62020000	0.00000000
240	1429.58880000	17.19500000	0.00000000
241	1463.04630000	15.23070000	0.00000000
242	1463.88980000	3.72600000	0.00000000
243	1464.07940000	5.36990000	0.00000000
244	1469.24740000	38.83000000	0.00000000
245	1472.66070000	3.61250000	0.00000000
246	1474.72600000	0.48810000	0.00000000
247	1475.22690000	6.82730000	0.00000000
248	1475.91430000	54.29570000	0.00000000
249	1477.57300000	6.12800000	0.00000000
250	1478.91530000	1.31390000	0.00000000
251	1482.28270000	0.79850000	0.00000000
252	1482.91500000	5.60070000	0.00000000

253	1486.27770000	13.63790000	0.00000000
254	1487.88270000	21.21260000	0.00000000
255	1488.35080000	5.75710000	0.00000000
256	1493.59290000	26.83410000	0.00000000
257	1494.58450000	146.75240000	0.00000000
258	1497.93870000	57.98740000	0.00000000
259	1500.20980000	8.64640000	0.00000000
260	1501.13970000	38.03870000	0.00000000
261	1502.16570000	45.29410000	0.00000000
262	1503.68530000	10.66730000	0.00000000
263	1505.48760000	72.77500000	0.00000000
264	1507.68380000	8.26130000	0.00000000
265	1508.41150000	25.75810000	0.00000000
266	1508.69670000	61.99080000	0.00000000
267	1513.07750000	23.90650000	0.00000000
268	1514.36580000	10.33000000	0.00000000
269	1522.03830000	3.90580000	0.00000000
270	1525.77740000	45.85580000	0.00000000
271	1527.12880000	47.20970000	0.00000000
272	1528.29130000	57.34420000	0.00000000
273	1530.49870000	2.70460000	0.00000000
274	1532.01790000	19.53670000	0.00000000
275	1542.65130000	22.47210000	0.00000000
276	1563.93790000	8.18080000	0.00000000
277	1606.22930000	13.48110000	0.00000000
278	1609.90550000	8.69920000	0.00000000
279	1618.55790000	15.51700000	0.00000000
280	1621.53870000	11.64620000	0.00000000
281	1635.77560000	35.42760000	0.00000000
282	1636.76970000	76.93490000	0.00000000
283	1637.78500000	14.01330000	0.00000000
284	1638.88870000	15.46390000	0.00000000
285	2958.58860000	30.53940000	0.00000000
286	2964.18220000	42.33660000	0.00000000
287	2966.79580000	24.42060000	0.00000000
288	2969.40540000	86.78790000	0.00000000
289	2975.64830000	75.16620000	0.00000000
290	2986.34300000	91.15900000	0.00000000
291	2990.43680000	46.88050000	0.00000000
292	2998.47920000	69.23330000	0.00000000
293	3001.61700000	37.57940000	0.00000000
294	3003.97890000	63.18230000	0.00000000
295	3004.64670000	87.72560000	0.00000000
296	3006.83210000	53.30260000	0.00000000
297	3006.95460000	35.13810000	0.00000000
298	3007.38850000	38.52530000	0.00000000
299	3014.23640000	9.76720000	0.00000000
300	3020.73040000	31.77840000	0.00000000
301	3023.96270000	22.25540000	0.00000000
302	3028.62050000	64.45320000	0.00000000
303	3036.18150000	51.36550000	0.00000000
304	3040.34520000	43.77640000	0.00000000
305	3041.49650000	25.76600000	0.00000000
306	3043.37050000	41.05450000	0.00000000
307	3046.33170000	36.27040000	0.00000000
308	3059.14450000	28.58770000	0.00000000
309	3062.41720000	21.10240000	0.00000000
310	3065.12010000	20.35980000	0.00000000
311	3084.51860000	37.46520000	0.00000000
312	3086.06000000	31.52220000	0.00000000
313	3101.54900000	13.55960000	0.00000000
314	3104.71620000	44.72880000	0.00000000
315	3106.06870000	39.69770000	0.00000000
316	3121.59310000	2.76010000	0.00000000

317	3130.66800000	9.08070000	0.00000000
318	3132.88520000	12.44880000	0.00000000
319	3136.92810000	4.87830000	0.00000000
320	3138.60350000	5.94870000	0.00000000
321	3165.23170000	5.80640000	0.00000000
322	3165.23450000	0.03670000	0.00000000
323	3165.69140000	6.21390000	0.00000000
324	3166.00180000	5.48420000	0.00000000
325	3180.21770000	15.12490000	0.00000000
326	3181.09630000	14.96030000	0.00000000
327	3182.38060000	15.66160000	0.00000000
328	3184.25600000	20.55630000	0.00000000
329	3189.52440000	19.23010000	0.00000000
330	3190.60510000	14.47530000	0.00000000
331	3196.24580000	13.79480000	0.00000000
332	3202.37950000	8.22940000	0.00000000
333	3203.76290000	8.61850000	0.00000000
334	3204.73520000	6.10550000	0.00000000
335	3210.72000000	9.41180000	0.00000000
336	3212.81880000	8.74810000	0.00000000

S6.6. Calculations on $Cu_2(1^*)_4(H_2O)_2$

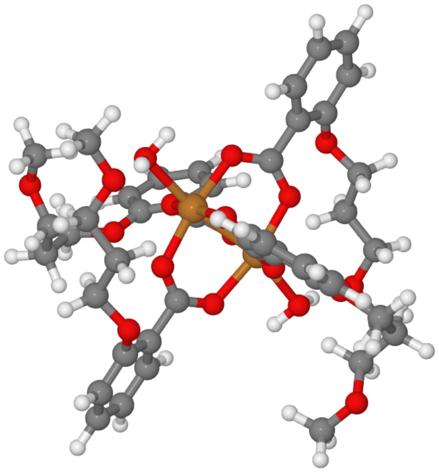

Route	: # opt freq b3lyp/genecp geom=connectivity int=ultrafine
SMILES	: COCCCOc1ccccc1[C]2O[Cu]345(O[C](O[Cu]3(O2)(O[C](O4)c6ccccc6OCCCOC)(O[C](O5)c7ccccc7OCCCOC)[OH2])c8ccccc8OCCCOC)[OH2]
Formula	: $C_{44}H_{56}Cu_2O_{18}$
Charge	: 0
Multiplicity	: 1
Dipole	: 2.9510 Debye
Energy	: -3460.07702365 a.u.
Gibbs Energy	: -3459.23808700 a.u.
Number of imaginary frequencies	: 0

S6.6.1. Cartesian Co-ordinates (XYZ format)

120

O	-0.35517299	-2.08409595	1.06525004
O	-2.01260996	-1.29084694	-0.24568000
O	-0.20503500	0.62006402	1.93455505
O	-1.49881995	1.44022000	0.28348300
C	-1.51756704	-2.11639810	0.56652999
C	-1.08609998	1.39622104	1.47584200
O	-4.20363808	-1.91700399	1.42207301
O	-1.71194005	4.19872713	0.90540200
O	-2.21709204	3.38449407	-3.74009490
C	-3.71452188	-3.17564702	1.31176698
O	-6.27418900	-0.28879800	-0.90980798
C	-2.04879093	3.66939998	2.12452102
C	-1.77796805	-4.57206821	0.89744997
H	-0.73387301	-4.64982796	0.62072903
C	-2.35888410	-3.30548811	0.94876802
C	-1.99745095	1.88250506	3.75268507
H	-1.72880995	0.86439002	4.00472689
C	-1.72894800	2.34046698	2.45791602
C	-5.61391783	-1.71449006	1.56096196

H	-5.95120192	-2.08673096	2.53605103
H	-6.14309216	-2.25254989	0.76904398
C	-2.71304011	4.11378622	-0.12634200
H	-3.40056992	4.96519518	-0.02931800
H	-3.27849388	3.18670702	-0.00800500
C	-1.98966801	4.11017799	-1.46358001
H	-1.39636505	5.02217102	-1.57079601
H	-1.29589701	3.26912689	-1.45491099
C	-5.87178183	-0.22033300	1.44168603
H	-5.31644201	0.30873901	2.22229505
H	-6.93852806	-0.04244100	1.61435294
C	-2.94265890	3.95179105	-2.64210892
H	-3.77992511	3.28564191	-2.38681507
H	-3.36666608	4.91660213	-2.95111609
C	-5.47960997	0.34433001	0.08307700
H	-5.65361595	1.43233800	0.06850800
H	-4.41515398	0.16376799	-0.10734900
C	-2.94646001	3.41329503	-4.95896721
H	-3.88163710	2.84346890	-4.88211823
H	-2.31366110	2.96271706	-5.72393513
H	-3.18013000	4.44462585	-5.25084114
C	-5.90062809	0.07859800	-2.22390389
H	-6.09727478	1.14634895	-2.41085505
H	-6.50958490	-0.51264298	-2.90953588
H	-4.83836412	-0.11752900	-2.41518688
C	-2.52789211	-5.71605682	1.15811598
C	-4.46208000	-4.32667589	1.58371997
H	-5.50273418	-4.24441719	1.86581302
C	-3.86954188	-5.58499098	1.49842799
C	-2.60426211	2.70672107	4.69334984
C	-2.63350606	4.49994421	3.08218598
H	-2.83914304	5.52864790	2.81018710
C	-2.91836691	4.02224684	4.35693216
O	1.89664495	1.04450703	0.05025000
O	1.66609800	-1.65624201	-0.82632500
O	0.54145598	1.41001904	-1.70823801
O	-0.03436000	-1.27829099	-2.25867891
C	1.57861698	1.64116204	-1.01320100
C	1.04300296	-1.83536696	-1.90709496
O	4.29111004	2.12822008	-0.12467000
O	2.05926394	-4.48081303	-1.22465599
O	3.13579798	-3.46731091	3.28876996
C	3.78818393	2.96780705	-1.05198097
O	4.91273022	2.85539794	3.29199791
C	2.16068196	-4.04103422	-2.52147508
C	1.95917499	3.58581400	-2.51744604
H	0.95583701	3.39384294	-2.87246394
C	2.47072792	2.74064398	-1.52493894
C	1.67803299	-2.42805791	-4.25470114
H	1.26159596	-1.47121501	-4.54374409
C	1.64856100	-2.78938890	-2.90408897
C	5.56487083	2.39280701	0.47274399
H	6.35850382	2.28157592	-0.27600899
H	5.58683586	3.41162992	0.87051201
C	3.18792105	-4.19869089	-0.37511700
H	3.97053599	-4.94809198	-0.55529100
H	3.58075809	-3.20847511	-0.61645901
C	2.69065404	-4.22484016	1.06104302
H	2.30626202	-5.21780491	1.31041598
H	1.85664797	-3.52466011	1.13125002
C	5.74338579	1.39127302	1.60380697
H	5.66591883	0.37598699	1.20366800
H	6.75085402	1.51425505	2.01555991
C	3.76922297	-3.81682491	2.05435991

H	4.33612299	-2.95042491	1.68273306
H	4.48307085	-4.63295603	2.23242688
C	4.71889687	1.56902504	2.71570992
H	4.85148907	0.78965300	3.48149705
H	3.70538998	1.47198498	2.31081295
C	4.06317186	-3.22701693	4.33747005
H	4.74485111	-2.40273404	4.09062719
H	3.48621988	-2.96164894	5.22350121
H	4.65321302	-4.12689018	4.55130196
C	3.91326690	3.19156289	4.23179007
H	3.90671706	2.49345493	5.08259201
H	4.13858318	4.19293308	4.60145903
H	2.91363692	3.19333792	3.77631497
C	2.69564605	4.64487219	-3.03182292
C	4.52753401	4.03674603	-1.58091402
H	5.53369379	4.21955299	-1.23131800
C	3.98390698	4.86558485	-2.55532098
C	2.23950195	-3.26942897	-5.20833111
C	2.69826007	-4.89337683	-3.48656106
H	3.05650210	-5.86592293	-3.16973591
C	2.74644089	-4.50876713	-4.82251978
H	-3.37576509	4.67909622	5.08839417
H	4.57874107	5.68562794	-2.94300008
H	3.16971588	-5.18000984	-5.56132603
H	-4.46793413	-6.46505022	1.70728195
H	-2.06728101	-6.69446421	1.09382105
H	2.26713896	-2.96606112	-6.24835777
H	2.26896691	5.28707409	-3.79283810
H	-2.81677008	2.32927108	5.68676281
Cu	-0.88020003	0.12993500	-1.08625805
Cu	0.89524502	-0.55977100	0.67981201
O	2.08357191	-0.93266398	2.60849690
H	1.38583004	-0.57498801	3.17007804
O	-2.14231300	0.63965398	-2.94352508
H	-1.63859904	0.05518800	-3.52220011
H	2.22974300	-1.85411596	2.89432311
H	-1.96031201	1.54903603	-3.24804997

S6.6.2. Frequencies

Mode	IR frequency	IR intensity	Raman intensity
1	4.81300000	0.01530000	0.00000000
2	9.69090000	0.00190000	0.00000000
3	15.10850000	0.17180000	0.00000000
4	15.40220000	0.13740000	0.00000000
5	16.88190000	0.14210000	0.00000000
6	17.03570000	0.02480000	0.00000000
7	19.91130000	0.11180000	0.00000000
8	22.10650000	0.27640000	0.00000000
9	24.22100000	0.46310000	0.00000000
10	27.38580000	0.59380000	0.00000000
11	30.55480000	0.69850000	0.00000000
12	31.82040000	0.02470000	0.00000000
13	34.68270000	0.69990000	0.00000000
14	45.32020000	0.54710000	0.00000000
15	46.74320000	2.21440000	0.00000000
16	49.45440000	0.52530000	0.00000000
17	52.01540000	0.38880000	0.00000000
18	55.13130000	1.04660000	0.00000000
19	60.01000000	1.15360000	0.00000000
20	62.95800000	2.71840000	0.00000000
21	65.33800000	0.87050000	0.00000000
22	70.11970000	1.12290000	0.00000000
23	74.88390000	5.71770000	0.00000000
24	76.02540000	2.21220000	0.00000000
25	77.51870000	0.11320000	0.00000000
26	83.55330000	1.58760000	0.00000000
27	86.67640000	1.47510000	0.00000000
28	87.01990000	1.86690000	0.00000000
29	94.60570000	2.28310000	0.00000000
30	96.53690000	1.83190000	0.00000000
31	97.99950000	0.34690000	0.00000000
32	102.27480000	6.44970000	0.00000000
33	107.08670000	0.96990000	0.00000000
34	109.25610000	1.81400000	0.00000000
35	110.50580000	3.30280000	0.00000000
36	114.11340000	2.61940000	0.00000000
37	117.18330000	3.23560000	0.00000000
38	120.05290000	1.57000000	0.00000000
39	123.97530000	0.87680000	0.00000000
40	128.53860000	9.07090000	0.00000000
41	133.22740000	0.62160000	0.00000000
42	138.59360000	1.53740000	0.00000000
43	140.85860000	0.01690000	0.00000000
44	144.01940000	22.34530000	0.00000000
45	152.72960000	4.28010000	0.00000000
46	155.00480000	2.86680000	0.00000000
47	157.88850000	2.64510000	0.00000000
48	160.59430000	24.79290000	0.00000000
49	174.78430000	2.58120000	0.00000000
50	177.65850000	9.33600000	0.00000000
51	179.24210000	3.21170000	0.00000000
52	180.55880000	1.19730000	0.00000000
53	186.45570000	1.64130000	0.00000000
54	187.40740000	0.88080000	0.00000000
55	192.53290000	0.27950000	0.00000000
56	201.18820000	1.51690000	0.00000000
57	207.08230000	2.50800000	0.00000000
58	209.89120000	5.35780000	0.00000000
59	211.36680000	9.84480000	0.00000000
60	215.75870000	4.82880000	0.00000000

61	217.38480000	2.68320000	0.00000000
62	221.33820000	8.23510000	0.00000000
63	225.40400000	0.03030000	0.00000000
64	230.13750000	1.45120000	0.00000000
65	232.52080000	3.44310000	0.00000000
66	236.45140000	34.33410000	0.00000000
67	241.23850000	16.91650000	0.00000000
68	244.40720000	2.17000000	0.00000000
69	247.64260000	5.12680000	0.00000000
70	249.20600000	7.55410000	0.00000000
71	254.23060000	11.06950000	0.00000000
72	267.98120000	9.17840000	0.00000000
73	274.49420000	2.80630000	0.00000000
74	282.19650000	2.39080000	0.00000000
75	284.51470000	10.31120000	0.00000000
76	287.55030000	8.37540000	0.00000000
77	290.27600000	3.85360000	0.00000000
78	299.36130000	14.93720000	0.00000000
79	310.72480000	10.10950000	0.00000000
80	317.66750000	0.69790000	0.00000000
81	320.35860000	0.68880000	0.00000000
82	322.20800000	0.52900000	0.00000000
83	388.44720000	13.27100000	0.00000000
84	395.97000000	4.68860000	0.00000000
85	397.40210000	9.82940000	0.00000000
86	400.55880000	38.26940000	0.00000000
87	404.87470000	9.15260000	0.00000000
88	407.80380000	1.10900000	0.00000000
89	409.01500000	4.94190000	0.00000000
90	409.68230000	59.91410000	0.00000000
91	422.09710000	10.92520000	0.00000000
92	438.02290000	6.86690000	0.00000000
93	442.68980000	1.57220000	0.00000000
94	446.11380000	4.92730000	0.00000000
95	446.81560000	6.73330000	0.00000000
96	457.48150000	3.53140000	0.00000000
97	468.28860000	0.94440000	0.00000000
98	472.61210000	3.87140000	0.00000000
99	474.49050000	3.84020000	0.00000000
100	487.98600000	30.80270000	0.00000000
101	493.42660000	0.83040000	0.00000000
102	498.66540000	37.25420000	0.00000000
103	527.88630000	5.46380000	0.00000000
104	534.75370000	1.75610000	0.00000000
105	537.85860000	265.53330000	0.00000000
106	543.37880000	2.74370000	0.00000000
107	546.22350000	7.12400000	0.00000000
108	552.61980000	184.60840000	0.00000000
109	555.44080000	52.26300000	0.00000000
110	558.27620000	32.41740000	0.00000000
111	578.58310000	2.97450000	0.00000000
112	589.85750000	6.54230000	0.00000000
113	597.59200000	0.42060000	0.00000000
114	598.54490000	5.29410000	0.00000000
115	617.18240000	7.55010000	0.00000000
116	618.91960000	6.82690000	0.00000000
117	623.94650000	4.02420000	0.00000000
118	628.44600000	2.95010000	0.00000000
119	677.85810000	57.11630000	0.00000000
120	678.83290000	3.19670000	0.00000000
121	688.63350000	62.11550000	0.00000000
122	691.43150000	0.20650000	0.00000000
123	712.20630000	10.12720000	0.00000000
124	732.26960000	6.95290000	0.00000000

125	733.54770000	12.62980000	0.00000000
126	735.06360000	31.09170000	0.00000000
127	736.98630000	65.39550000	0.00000000
128	741.82990000	80.20510000	0.00000000
129	764.83920000	45.27390000	0.00000000
130	769.42640000	50.92320000	0.00000000
131	776.29850000	4.93310000	0.00000000
132	778.01590000	11.03310000	0.00000000
133	778.93100000	5.77130000	0.00000000
134	780.10450000	7.96700000	0.00000000
135	781.09390000	35.76220000	0.00000000
136	782.82420000	33.57770000	0.00000000
137	801.34930000	3.52230000	0.00000000
138	806.73820000	2.47310000	0.00000000
139	809.85860000	8.40130000	0.00000000
140	811.77650000	0.35230000	0.00000000
141	828.31250000	7.73880000	0.00000000
142	830.15040000	0.91280000	0.00000000
143	838.02250000	8.57250000	0.00000000
144	841.16540000	1.41120000	0.00000000
145	865.65380000	2.44140000	0.00000000
146	869.10440000	4.03020000	0.00000000
147	870.49210000	10.98110000	0.00000000
148	870.85380000	0.38610000	0.00000000
149	872.85080000	6.93320000	0.00000000
150	879.39790000	0.11720000	0.00000000
151	892.01150000	4.08020000	0.00000000
152	893.35180000	2.07330000	0.00000000
153	899.69230000	4.74810000	0.00000000
154	899.86220000	0.53760000	0.00000000
155	904.71710000	4.26970000	0.00000000
156	907.90730000	5.34280000	0.00000000
157	943.85180000	14.39180000	0.00000000
158	944.75790000	21.20740000	0.00000000
159	945.82880000	15.57600000	0.00000000
160	948.20720000	20.78460000	0.00000000
161	959.05280000	0.63790000	0.00000000
162	963.16160000	0.31910000	0.00000000
163	967.07430000	1.05800000	0.00000000
164	973.97030000	0.76600000	0.00000000
165	982.53100000	0.54190000	0.00000000
166	990.37680000	0.14180000	0.00000000
167	992.42350000	0.20380000	0.00000000
168	994.54490000	32.54590000	0.00000000
169	998.11710000	37.22850000	0.00000000
170	998.71300000	1.48730000	0.00000000
171	1023.22380000	75.74280000	0.00000000
172	1023.60000000	150.38060000	0.00000000
173	1059.54680000	19.81190000	0.00000000
174	1059.87770000	14.34790000	0.00000000
175	1061.34920000	59.84660000	0.00000000
176	1064.85980000	43.10040000	0.00000000
177	1065.12870000	5.68540000	0.00000000
178	1066.24620000	5.75180000	0.00000000
179	1070.26650000	15.38550000	0.00000000
180	1075.44890000	14.55360000	0.00000000
181	1085.36920000	14.47500000	0.00000000
182	1087.15470000	15.72030000	0.00000000
183	1105.28260000	11.31340000	0.00000000
184	1106.90210000	10.74600000	0.00000000
185	1114.72580000	121.25050000	0.00000000
186	1114.92320000	6.13650000	0.00000000
187	1116.20120000	10.77510000	0.00000000
188	1117.72180000	5.72240000	0.00000000

189	1122.38530000	39.49320000	0.00000000
190	1123.80430000	92.51240000	0.00000000
191	1125.30040000	97.16230000	0.00000000
192	1127.69420000	117.22860000	0.00000000
193	1148.79250000	66.30050000	0.00000000
194	1149.16300000	133.70370000	0.00000000
195	1159.14570000	2.50610000	0.00000000
196	1160.48650000	0.64590000	0.00000000
197	1166.42160000	9.76560000	0.00000000
198	1166.71480000	7.24900000	0.00000000
199	1172.10120000	15.56270000	0.00000000
200	1173.64390000	8.86840000	0.00000000
201	1177.92090000	3.36190000	0.00000000
202	1178.68780000	4.71700000	0.00000000
203	1181.38390000	4.23460000	0.00000000
204	1181.88160000	11.83840000	0.00000000
205	1182.18520000	2.28130000	0.00000000
206	1182.99880000	4.20860000	0.00000000
207	1186.17970000	14.18320000	0.00000000
208	1187.55880000	26.40510000	0.00000000
209	1214.19300000	7.37810000	0.00000000
210	1214.25540000	36.85980000	0.00000000
211	1216.74000000	23.44200000	0.00000000
212	1217.02320000	20.76540000	0.00000000
213	1239.18370000	45.53250000	0.00000000
214	1239.85670000	39.10920000	0.00000000
215	1254.04410000	16.75470000	0.00000000
216	1257.85950000	20.66510000	0.00000000
217	1259.11690000	51.85310000	0.00000000
218	1261.97260000	11.58640000	0.00000000
219	1262.25890000	58.20600000	0.00000000
220	1264.06740000	6.79410000	0.00000000
221	1280.80560000	289.69430000	0.00000000
222	1281.66980000	77.48920000	0.00000000
223	1283.10520000	17.33050000	0.00000000
224	1283.39720000	15.06470000	0.00000000
225	1301.10530000	139.90730000	0.00000000
226	1306.24010000	56.62220000	0.00000000
227	1307.50510000	6.49170000	0.00000000
228	1309.87350000	8.79050000	0.00000000
229	1316.21370000	3.20290000	0.00000000
230	1318.29970000	4.94910000	0.00000000
231	1319.83860000	4.20030000	0.00000000
232	1320.50910000	5.08890000	0.00000000
233	1320.82130000	2.18380000	0.00000000
234	1321.23900000	3.49020000	0.00000000
235	1325.92570000	51.01830000	0.00000000
236	1327.77250000	7.65230000	0.00000000
237	1328.02740000	0.53080000	0.00000000
238	1328.91100000	38.53500000	0.00000000
239	1375.01580000	155.00560000	0.00000000
240	1387.97960000	78.28190000	0.00000000
241	1388.33390000	100.29300000	0.00000000
242	1390.92240000	39.00170000	0.00000000
243	1398.24600000	400.84240000	0.00000000
244	1402.63450000	24.99670000	0.00000000
245	1414.04710000	8.69360000	0.00000000
246	1414.38710000	6.80520000	0.00000000
247	1421.70960000	1.95640000	0.00000000
248	1423.78820000	1.20780000	0.00000000
249	1432.32580000	18.97340000	0.00000000
250	1432.39250000	16.99880000	0.00000000
251	1437.34410000	35.26700000	0.00000000
252	1439.02660000	46.32640000	0.00000000

253	1462.58890000	9.53450000	0.00000000
254	1465.21480000	2.18090000	0.00000000
255	1465.27150000	12.41050000	0.00000000
256	1473.74900000	29.60240000	0.00000000
257	1475.37440000	26.28130000	0.00000000
258	1476.46860000	0.39260000	0.00000000
259	1478.12370000	0.53210000	0.00000000
260	1478.85180000	0.57690000	0.00000000
261	1479.56450000	5.27880000	0.00000000
262	1480.67910000	2.39690000	0.00000000
263	1483.28570000	1.43300000	0.00000000
264	1486.72210000	10.48040000	0.00000000
265	1487.33120000	6.89890000	0.00000000
266	1487.71320000	8.74340000	0.00000000
267	1493.57300000	242.62660000	0.00000000
268	1498.51990000	38.07250000	0.00000000
269	1499.94170000	1.45180000	0.00000000
270	1502.28530000	8.75960000	0.00000000
271	1502.58150000	12.08070000	0.00000000
272	1503.34680000	66.53060000	0.00000000
273	1504.68280000	71.45970000	0.00000000
274	1505.98120000	63.23780000	0.00000000
275	1508.13950000	6.71130000	0.00000000
276	1509.96660000	0.97610000	0.00000000
277	1510.22490000	33.45680000	0.00000000
278	1516.65180000	29.96070000	0.00000000
279	1517.16240000	57.04760000	0.00000000
280	1517.93160000	48.38110000	0.00000000
281	1522.97180000	16.36560000	0.00000000
282	1526.38160000	11.17750000	0.00000000
283	1526.65070000	23.34320000	0.00000000
284	1529.90160000	7.06380000	0.00000000
285	1531.40860000	3.84490000	0.00000000
286	1532.00710000	14.65200000	0.00000000
287	1541.53200000	22.17610000	0.00000000
288	1555.75580000	7.06460000	0.00000000
289	1611.42990000	13.23140000	0.00000000
290	1613.07370000	5.42990000	0.00000000
291	1613.74780000	29.11710000	0.00000000
292	1620.82370000	14.59760000	0.00000000
293	1635.80460000	57.03680000	0.00000000
294	1637.35720000	36.28840000	0.00000000
295	1637.87780000	0.55880000	0.00000000
296	1638.91430000	59.21580000	0.00000000
297	1640.93380000	29.47160000	0.00000000
298	1648.37980000	34.12580000	0.00000000
299	2947.72920000	23.60210000	0.00000000
300	2956.61310000	126.84210000	0.00000000
301	2959.70430000	32.19260000	0.00000000
302	2969.22820000	95.79700000	0.00000000
303	2969.63900000	23.40120000	0.00000000
304	2971.56440000	18.51720000	0.00000000
305	2986.83000000	21.99280000	0.00000000
306	2987.20860000	47.13880000	0.00000000
307	2988.63780000	150.16040000	0.00000000
308	2990.19030000	90.00730000	0.00000000
309	3002.90060000	46.26930000	0.00000000
310	3003.79130000	71.26360000	0.00000000
311	3004.18470000	45.61580000	0.00000000
312	3007.69850000	61.43400000	0.00000000
313	3011.52310000	60.44450000	0.00000000
314	3026.47220000	28.66700000	0.00000000
315	3031.68220000	12.12000000	0.00000000
316	3033.71930000	22.36520000	0.00000000

317	3038.70940000	56.55910000	0.00000000
318	3039.04450000	47.89710000	0.00000000
319	3041.47260000	28.81660000	0.00000000
320	3048.79860000	21.75050000	0.00000000
321	3051.21350000	16.39480000	0.00000000
322	3055.21000000	12.88430000	0.00000000
323	3063.65310000	17.76680000	0.00000000
324	3064.02840000	20.49340000	0.00000000
325	3077.73500000	28.32320000	0.00000000
326	3077.97680000	17.40200000	0.00000000
327	3081.15520000	37.80310000	0.00000000
328	3082.92350000	40.36540000	0.00000000
329	3103.41140000	40.59030000	0.00000000
330	3103.80870000	37.32790000	0.00000000
331	3112.12200000	13.39330000	0.00000000
332	3116.36200000	20.65250000	0.00000000
333	3117.78610000	20.20840000	0.00000000
334	3120.92670000	7.78730000	0.00000000
335	3164.00460000	8.53560000	0.00000000
336	3164.95610000	7.15150000	0.00000000
337	3165.63970000	2.27540000	0.00000000
338	3165.95070000	1.81440000	0.00000000
339	3179.71780000	13.49760000	0.00000000
340	3179.98120000	13.60160000	0.00000000
341	3183.44900000	18.96630000	0.00000000
342	3184.67310000	24.27770000	0.00000000
343	3189.34620000	17.97660000	0.00000000
344	3189.71440000	15.90690000	0.00000000
345	3198.45690000	12.10030000	0.00000000
346	3199.17210000	8.43760000	0.00000000
347	3199.97650000	8.59230000	0.00000000
348	3207.49120000	11.19740000	0.00000000
349	3212.49840000	5.92060000	0.00000000
350	3215.07700000	8.52510000	0.00000000
351	3590.68950000	812.36770000	0.00000000
352	3591.59070000	114.00090000	0.00000000
353	3840.16520000	76.44310000	0.00000000
354	3840.67290000	126.68080000	0.00000000

S6.7. Calculations on $Cu_2(1^*)_4(MeCN)_2$

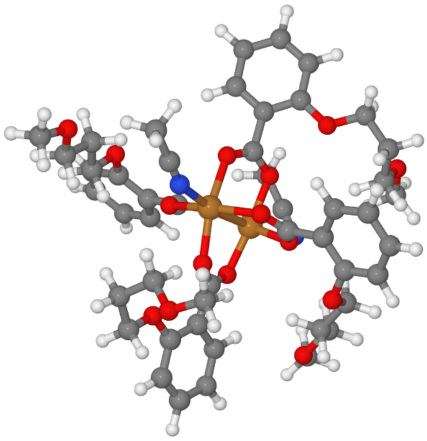

Route	: # opt freq b3lyp/genecp geom=connectivity int=ultrafine scf=yqc	
SMILES	: CC#N.CC#[N][Cu]1234O[C](O[Cu]1(O[C](O2)c5ccccc5OCCCOC)(O[C](O3)c6ccccc6OCCCOC)O[C](O4)c7ccccc7OCCCOC)c8ccccc8OCCCOC	
Formula	: $C_{48}H_{58}Cu_2N_2O_{16}$	
Charge	: 0	
Multiplicity	: 1	
Dipole	: 17.1020	Debye
Energy	: -3572.73632621	a.u.
Gibbs Energy	: -3571.86987800	a.u.
Number of imaginary frequencies	: 0	

S6.7.1. Cartesian Co-ordinates (XYZ format)

126

O	-0.31935701	-2.17103004	-0.85143101
O	1.59674203	-1.81398702	0.28429300
O	0.46300900	0.33082399	-2.02138495
O	1.95419896	0.85683900	-0.41407299
C	0.80330998	-2.51977611	-0.41047499
C	1.55117905	0.80465800	-1.61726999
O	3.36788297	-3.26053905	-1.36818695
O	3.11533499	3.26671600	-1.37372899
O	3.40401602	4.14564991	3.28575802
C	2.51994801	-4.28257513	-1.10423195
O	6.45505095	-2.15695405	0.08522700
C	3.28850007	2.47072601	-2.47169995
C	0.27835199	-4.95083284	-0.46649200
H	-0.72071302	-4.66403818	-0.16156200
C	1.21929395	-3.94283390	-0.67514497
C	2.61389995	0.61140198	-3.86640406
H	1.98238599	-0.25618199	-4.01079988
C	2.50825596	1.31625402	-2.66231394
C	4.69792986	-3.54287601	-1.82115996

H	4.65478897	-4.11989784	-2.75257611
H	5.23237419	-4.12541103	-1.06434500
C	4.00003290	3.04927611	-0.25348499
H	4.99241495	3.45288706	-0.49484301
H	4.08427382	1.97651303	-0.06695800
C	3.37441611	3.75180292	0.93879700
H	3.34760499	4.83160019	0.76663202
H	2.34433293	3.40324306	1.02168596
C	5.39842987	-2.21356392	-2.05258489
H	4.81852579	-1.61619103	-2.76220012
H	6.37223721	-2.41950893	-2.50936389
C	4.09214306	3.45905399	2.24709511
H	4.09333706	2.37923002	2.44956398
H	5.14100599	3.79894996	2.21044111
C	5.61213684	-1.40711999	-0.77966100
H	6.08536005	-0.44490200	-1.02722394
H	4.65131807	-1.19668806	-0.29304200
C	3.93597198	3.86690402	4.56442595
H	3.88053989	2.79530406	4.79974079
H	3.34228992	4.42305183	5.29131413
H	4.98546314	4.18871212	4.64443922
C	6.74278498	-1.47780895	1.29254496
H	7.30700207	-0.55179298	1.10634398
H	7.35540390	-2.14509010	1.90068197
H	5.82888794	-1.22063804	1.84304905
C	0.61327797	-6.29288721	-0.62720197
C	2.84987712	-5.63287878	-1.27358401
H	3.84203410	-5.91352320	-1.59836900
C	1.90280402	-6.62504101	-1.02712202
C	3.51156712	1.00921595	-4.85081816
C	4.17336893	2.87555289	-3.47414088
H	4.74133205	3.78534102	-3.31830812
C	4.29372406	2.14604807	-4.65200520
O	-1.27161098	1.69123602	-0.17545600
O	-2.05057907	-0.80699402	0.98099601
O	0.13541999	1.59518504	1.57956100
O	-0.19824800	-1.07006502	2.23915005
C	-0.74773997	2.17106700	0.86009198
C	-1.45071900	-1.15174496	2.02447891
O	-3.14748406	3.55358791	0.04987400
O	-3.77713609	-2.93623090	1.76747096
O	-6.49678421	-2.61228800	-2.16042209
C	-2.31532502	4.20853806	0.88872999
O	-3.51222610	4.26384497	-3.40647697
C	-3.37479496	-2.51562905	3.01099610
C	-0.29863399	4.19934988	2.23466992
H	0.59204400	3.68685198	2.57051992
C	-1.14382803	3.54383707	1.33027804
C	-1.83706605	-1.37052500	4.48188210
H	-0.95990700	-0.74763602	4.60187483
C	-2.24820495	-1.69330800	3.18191195
C	-4.23952103	4.24529886	-0.56235802
H	-5.00262308	4.48219919	0.18913200
H	-3.88587594	5.17445993	-1.01859498
C	-4.83401394	-2.16100192	1.17687500
H	-5.71541500	-2.19209194	1.83204997
H	-4.50115585	-1.12437201	1.07628000
C	-5.14366102	-2.76632905	-0.18269201
H	-5.38542795	-3.82640791	-0.06576500
H	-4.24819088	-2.69593406	-0.80377197
C	-4.80272102	3.32450700	-1.63477898
H	-5.10845709	2.37922812	-1.17603600
H	-5.69479418	3.79517198	-2.06167006
C	-6.30339289	-2.05496812	-0.86321503

H	-6.10437298	-0.97564799	-0.94799602
H	-7.22548723	-2.17503309	-0.27442399
C	-3.80193996	3.03899288	-2.74627209
H	-4.22702980	2.31765795	-3.46208501
H	-2.88963509	2.60254693	-2.32292509
C	-7.66228199	-2.12019300	-2.79639912
H	-7.62151909	-1.03045297	-2.93539906
H	-7.72920322	-2.60219002	-3.77253699
H	-8.56432438	-2.36382604	-2.21901989
C	-2.45726991	4.14862108	-4.34038782
H	-2.70879698	3.44588304	-5.14921808
H	-2.29735899	5.13761282	-4.77182388
H	-1.52898705	3.80980110	-3.86183906
C	-0.56599498	5.48584414	2.68477798
C	-2.58347106	5.50232220	1.35722303
H	-3.47650909	6.01782799	1.03304195
C	-1.71379006	6.13312006	2.24054289
C	-2.54119992	-1.82201099	5.59115982
C	-4.06238604	-2.98828793	4.13090181
H	-4.90796614	-3.64757490	3.97212505
C	-3.65555906	-2.64017391	5.41392994
H	4.98607016	2.47367907	-5.41963577
H	-1.94364095	7.13709402	2.58101797
H	-4.20248985	-3.01362395	6.27248383
H	2.18247008	-7.66452217	-1.15905905
H	-0.12498800	-7.06432676	-0.44440201
H	-2.21558499	-1.54681098	6.58745480
H	0.12055300	5.97175598	3.36689496
H	3.58893895	0.44508100	-5.77304220
Cu	0.92953300	-0.08045200	0.96120298
Cu	-1.02779996	-0.24777600	-0.72601002
N	-2.56445694	-0.62811899	-2.27590609
N	3.27592397	-0.32028899	3.00258303
C	2.90847707	-1.40304804	3.15502906
C	2.42856407	-2.76441789	3.32854509
H	1.34083402	-2.74485302	3.41263700
H	2.69820809	-3.35691905	2.45318508
H	2.86431789	-3.21019602	4.22474480
C	-3.11105299	-1.23476100	-3.08564591
C	-3.81669307	-2.02536106	-4.07873392
H	-4.72779799	-2.42757702	-3.62557292
H	-4.07655621	-1.40447402	-4.93839502
H	-3.17978692	-2.84690404	-4.41289520

S6.7.2. Frequencies

Mode	IR frequency	IR intensity	Raman intensity
1	5.81960000	0.18610000	0.00000000
2	7.19060000	0.04480000	0.00000000
3	13.40690000	1.74730000	0.00000000
4	14.28830000	0.04410000	0.00000000
5	16.07050000	0.14340000	0.00000000
6	17.10820000	1.83310000	0.00000000
7	17.84770000	0.79910000	0.00000000
8	18.00880000	0.06710000	0.00000000
9	19.24200000	0.67370000	0.00000000
10	21.47120000	0.13260000	0.00000000
11	23.52070000	1.25530000	0.00000000
12	25.22750000	2.12540000	0.00000000
13	26.92900000	0.36230000	0.00000000
14	29.83510000	1.59180000	0.00000000
15	32.07420000	5.28440000	0.00000000
16	35.02020000	1.16180000	0.00000000
17	36.36520000	2.02980000	0.00000000
18	37.44800000	0.28290000	0.00000000
19	41.85410000	4.04020000	0.00000000
20	47.20410000	2.55110000	0.00000000
21	52.28320000	0.29040000	0.00000000
22	53.98620000	1.49690000	0.00000000
23	56.63530000	0.13030000	0.00000000
24	58.71540000	0.83180000	0.00000000
25	60.53530000	4.47690000	0.00000000
26	61.57010000	0.23500000	0.00000000
27	71.14710000	0.53310000	0.00000000
28	72.14140000	0.60700000	0.00000000
29	75.06000000	1.85620000	0.00000000
30	75.90890000	3.48760000	0.00000000
31	76.82570000	1.12930000	0.00000000
32	80.61840000	0.56630000	0.00000000
33	83.04820000	1.25650000	0.00000000
34	84.92300000	0.80960000	0.00000000
35	89.23880000	2.97330000	0.00000000
36	94.05460000	13.95550000	0.00000000
37	95.60830000	0.73030000	0.00000000
38	99.31150000	0.88790000	0.00000000
39	101.74860000	6.55880000	0.00000000
40	104.88330000	5.22850000	0.00000000
41	109.56930000	0.08030000	0.00000000
42	112.93890000	6.92660000	0.00000000
43	113.70850000	1.46260000	0.00000000
44	118.06100000	0.66560000	0.00000000
45	118.78600000	0.68190000	0.00000000
46	120.51350000	0.53580000	0.00000000
47	125.93210000	3.51890000	0.00000000
48	128.30830000	0.19870000	0.00000000
49	132.01990000	0.97330000	0.00000000
50	134.82090000	5.55540000	0.00000000
51	138.79830000	1.51770000	0.00000000
52	140.34330000	0.75610000	0.00000000
53	146.12550000	7.07680000	0.00000000
54	150.29880000	3.67450000	0.00000000
55	152.19260000	2.08700000	0.00000000
56	155.90620000	6.49980000	0.00000000
57	163.53960000	5.02160000	0.00000000
58	164.98880000	2.58400000	0.00000000
59	180.49680000	2.39780000	0.00000000
60	181.86110000	1.10680000	0.00000000

61	184.84970000	5.71040000	0.00000000
62	186.32180000	5.85590000	0.00000000
63	197.12400000	3.92460000	0.00000000
64	198.94530000	2.25800000	0.00000000
65	203.04070000	6.98660000	0.00000000
66	207.64850000	3.73670000	0.00000000
67	208.99700000	4.14670000	0.00000000
68	214.02350000	0.57100000	0.00000000
69	221.20270000	0.41520000	0.00000000
70	224.13330000	3.68910000	0.00000000
71	231.11680000	2.30570000	0.00000000
72	231.46460000	5.45350000	0.00000000
73	233.13010000	6.82520000	0.00000000
74	236.12370000	11.48130000	0.00000000
75	241.96330000	1.71770000	0.00000000
76	245.83370000	1.27050000	0.00000000
77	247.26710000	10.78750000	0.00000000
78	267.79500000	8.70340000	0.00000000
79	275.56690000	4.70270000	0.00000000
80	277.96960000	3.70660000	0.00000000
81	283.79480000	1.87880000	0.00000000
82	291.46740000	4.83480000	0.00000000
83	295.64800000	7.50370000	0.00000000
84	299.08400000	5.42090000	0.00000000
85	304.15880000	6.98960000	0.00000000
86	315.27670000	2.83930000	0.00000000
87	320.62160000	0.07940000	0.00000000
88	322.43080000	2.67790000	0.00000000
89	387.78640000	0.58890000	0.00000000
90	389.76630000	0.70310000	0.00000000
91	392.82940000	0.31760000	0.00000000
92	396.27630000	1.88090000	0.00000000
93	401.82170000	1.98210000	0.00000000
94	403.39480000	1.21320000	0.00000000
95	404.04770000	0.67380000	0.00000000
96	404.68830000	8.16060000	0.00000000
97	407.39850000	5.26930000	0.00000000
98	413.79510000	5.09780000	0.00000000
99	422.98920000	3.62040000	0.00000000
100	435.12330000	8.18550000	0.00000000
101	445.07010000	3.96460000	0.00000000
102	448.27730000	1.60820000	0.00000000
103	449.87190000	4.16880000	0.00000000
104	458.36170000	5.92600000	0.00000000
105	464.84670000	2.41600000	0.00000000
106	471.74480000	2.07400000	0.00000000
107	474.34480000	2.76200000	0.00000000
108	493.43660000	22.59190000	0.00000000
109	496.87080000	12.34360000	0.00000000
110	507.97480000	37.67540000	0.00000000
111	529.81210000	5.11230000	0.00000000
112	533.43630000	1.11800000	0.00000000
113	536.61060000	3.17520000	0.00000000
114	545.47140000	4.39040000	0.00000000
115	554.42700000	8.55550000	0.00000000
116	558.28980000	3.22080000	0.00000000
117	580.06060000	1.83720000	0.00000000
118	591.08020000	3.21880000	0.00000000
119	598.77550000	3.03960000	0.00000000
120	600.36740000	0.79150000	0.00000000
121	620.81110000	5.64680000	0.00000000
122	620.95050000	6.26190000	0.00000000
123	621.53740000	4.78150000	0.00000000
124	628.86820000	3.13630000	0.00000000

125	678.63780000	27.03040000	0.00000000
126	679.11390000	32.83910000	0.00000000
127	689.05400000	64.07550000	0.00000000
128	691.08310000	2.05490000	0.00000000
129	712.19670000	9.38350000	0.00000000
130	730.56390000	17.04540000	0.00000000
131	733.28120000	31.38800000	0.00000000
132	736.14640000	4.60980000	0.00000000
133	766.40790000	50.52200000	0.00000000
134	771.91740000	46.57410000	0.00000000
135	776.00630000	9.67220000	0.00000000
136	780.69770000	37.70430000	0.00000000
137	781.61650000	9.98900000	0.00000000
138	785.05950000	34.07010000	0.00000000
139	786.06290000	7.19240000	0.00000000
140	788.63590000	2.18910000	0.00000000
141	801.87170000	3.06800000	0.00000000
142	805.56790000	3.35350000	0.00000000
143	809.22870000	7.59190000	0.00000000
144	813.96750000	1.49460000	0.00000000
145	829.78250000	9.37850000	0.00000000
146	831.52150000	1.64150000	0.00000000
147	837.73890000	9.49250000	0.00000000
148	841.66560000	2.07320000	0.00000000
149	867.21350000	2.24470000	0.00000000
150	869.23630000	2.17590000	0.00000000
151	869.83270000	12.37830000	0.00000000
152	870.70230000	0.31500000	0.00000000
153	873.12740000	7.73280000	0.00000000
154	879.03590000	1.42020000	0.00000000
155	893.66180000	6.14220000	0.00000000
156	895.59960000	1.89000000	0.00000000
157	897.88380000	3.48410000	0.00000000
158	899.68500000	2.22450000	0.00000000
159	901.80960000	2.52540000	0.00000000
160	903.01450000	0.59010000	0.00000000
161	929.66320000	1.60670000	0.00000000
162	940.03540000	2.06340000	0.00000000
163	944.38180000	14.19030000	0.00000000
164	946.65940000	15.38160000	0.00000000
165	956.92170000	21.28730000	0.00000000
166	959.35550000	21.25500000	0.00000000
167	961.27980000	1.13060000	0.00000000
168	967.33520000	0.72590000	0.00000000
169	973.08670000	0.35850000	0.00000000
170	974.96270000	1.05780000	0.00000000
171	984.85320000	0.82540000	0.00000000
172	990.02480000	0.36960000	0.00000000
173	995.92180000	1.12920000	0.00000000
174	995.97090000	35.02360000	0.00000000
175	997.52550000	39.28800000	0.00000000
176	1003.21150000	1.86390000	0.00000000
177	1016.77070000	102.49270000	0.00000000
178	1030.55510000	111.80190000	0.00000000
179	1059.20070000	14.46030000	0.00000000
180	1061.05660000	7.56320000	0.00000000
181	1061.60800000	52.31390000	0.00000000
182	1064.15400000	27.36560000	0.00000000
183	1065.11780000	34.82460000	0.00000000
184	1065.65310000	9.45580000	0.00000000
185	1066.64120000	10.38650000	0.00000000
186	1069.98910000	2.97100000	0.00000000
187	1070.11660000	2.23500000	0.00000000
188	1070.41340000	15.79380000	0.00000000

189	1075.09810000	13.84590000	0.00000000
190	1077.96350000	6.51390000	0.00000000
191	1094.17080000	10.21080000	0.00000000
192	1096.05870000	10.17150000	0.00000000
193	1107.26240000	13.52650000	0.00000000
194	1110.61390000	5.51280000	0.00000000
195	1115.53130000	71.55550000	0.00000000
196	1115.90790000	25.34770000	0.00000000
197	1117.03330000	6.98300000	0.00000000
198	1117.32170000	8.48480000	0.00000000
199	1123.82830000	70.64480000	0.00000000
200	1125.49670000	64.15370000	0.00000000
201	1138.07840000	150.03260000	0.00000000
202	1145.02450000	59.19260000	0.00000000
203	1145.86950000	189.07140000	0.00000000
204	1149.34100000	107.71200000	0.00000000
205	1161.85300000	2.20570000	0.00000000
206	1162.34540000	3.14610000	0.00000000
207	1165.20240000	11.60330000	0.00000000
208	1166.50160000	6.55870000	0.00000000
209	1171.63080000	15.72940000	0.00000000
210	1173.48000000	10.57500000	0.00000000
211	1178.19440000	5.42930000	0.00000000
212	1178.81710000	3.66630000	0.00000000
213	1180.75990000	11.07470000	0.00000000
214	1182.39730000	11.98890000	0.00000000
215	1184.27490000	7.38530000	0.00000000
216	1184.50310000	3.71560000	0.00000000
217	1186.72850000	18.11350000	0.00000000
218	1187.19970000	16.73600000	0.00000000
219	1214.03840000	24.97120000	0.00000000
220	1215.83530000	23.73420000	0.00000000
221	1219.83230000	15.57920000	0.00000000
222	1223.07210000	16.45340000	0.00000000
223	1236.89930000	18.42430000	0.00000000
224	1239.02390000	2.35470000	0.00000000
225	1250.40870000	107.72620000	0.00000000
226	1255.39380000	17.66280000	0.00000000
227	1256.17340000	18.91100000	0.00000000
228	1259.37500000	88.00050000	0.00000000
229	1261.29100000	24.76040000	0.00000000
230	1265.31880000	3.62630000	0.00000000
231	1278.09370000	266.39680000	0.00000000
232	1279.52710000	110.82660000	0.00000000
233	1281.83110000	12.21280000	0.00000000
234	1284.32890000	14.07300000	0.00000000
235	1301.29880000	119.20610000	0.00000000
236	1303.51490000	2.17130000	0.00000000
237	1303.96900000	67.29780000	0.00000000
238	1307.90380000	0.40730000	0.00000000
239	1312.90500000	0.77360000	0.00000000
240	1313.14900000	2.37790000	0.00000000
241	1319.65300000	7.10260000	0.00000000
242	1319.92510000	2.20670000	0.00000000
243	1320.98020000	6.15140000	0.00000000
244	1322.07860000	10.66700000	0.00000000
245	1322.53140000	25.06750000	0.00000000
246	1323.04790000	35.74830000	0.00000000
247	1323.38030000	9.67950000	0.00000000
248	1328.90390000	35.55700000	0.00000000
249	1360.61830000	125.02310000	0.00000000
250	1375.71700000	122.76960000	0.00000000
251	1385.73900000	323.06000000	0.00000000
252	1387.23840000	3.12030000	0.00000000

253	1390.74400000	115.55890000	0.00000000
254	1393.48750000	89.52680000	0.00000000
255	1406.78860000	32.04390000	0.00000000
256	1413.36920000	6.80380000	0.00000000
257	1414.91020000	6.47970000	0.00000000
258	1415.12510000	9.13210000	0.00000000
259	1422.58450000	8.38850000	0.00000000
260	1424.96900000	1.40740000	0.00000000
261	1430.67400000	20.79970000	0.00000000
262	1430.97610000	11.67790000	0.00000000
263	1438.01160000	48.88020000	0.00000000
264	1440.65050000	41.81250000	0.00000000
265	1461.32880000	13.16590000	0.00000000
266	1462.88490000	6.23620000	0.00000000
267	1464.50030000	5.47040000	0.00000000
268	1467.19860000	4.75790000	0.00000000
269	1471.39270000	3.93490000	0.00000000
270	1473.05830000	9.45880000	0.00000000
271	1476.59430000	26.14670000	0.00000000
272	1477.23920000	35.50920000	0.00000000
273	1478.96490000	18.85680000	0.00000000
274	1479.89090000	3.35660000	0.00000000
275	1481.07430000	1.92990000	0.00000000
276	1481.30290000	1.62440000	0.00000000
277	1481.77160000	1.16060000	0.00000000
278	1483.27790000	5.25230000	0.00000000
279	1483.48900000	2.82150000	0.00000000
280	1483.67390000	6.36180000	0.00000000
281	1485.86950000	5.51480000	0.00000000
282	1489.34110000	8.01510000	0.00000000
283	1497.60630000	61.63840000	0.00000000
284	1501.49850000	44.60920000	0.00000000
285	1502.36190000	6.93200000	0.00000000
286	1503.37340000	80.59650000	0.00000000
287	1504.56710000	25.53920000	0.00000000
288	1505.71590000	28.70810000	0.00000000
289	1505.96310000	72.19580000	0.00000000
290	1506.69270000	163.88800000	0.00000000
291	1508.80220000	18.18420000	0.00000000
292	1509.90070000	4.00550000	0.00000000
293	1511.00470000	21.92970000	0.00000000
294	1518.33550000	38.92870000	0.00000000
295	1518.44220000	3.80930000	0.00000000
296	1524.12040000	55.29290000	0.00000000
297	1526.31830000	40.78750000	0.00000000
298	1528.13570000	20.48630000	0.00000000
299	1530.54930000	3.17220000	0.00000000
300	1532.89570000	1.32880000	0.00000000
301	1533.70210000	9.01060000	0.00000000
302	1537.06270000	2.37920000	0.00000000
303	1545.84130000	22.80800000	0.00000000
304	1561.29820000	17.77130000	0.00000000
305	1611.07250000	12.43270000	0.00000000
306	1613.71960000	13.73040000	0.00000000
307	1614.80190000	32.12550000	0.00000000
308	1619.09740000	12.70800000	0.00000000
309	1635.50660000	58.51200000	0.00000000
310	1635.64850000	22.44510000	0.00000000
311	1637.35250000	4.70530000	0.00000000
312	1637.71020000	54.41210000	0.00000000
313	2354.77670000	19.17800000	0.00000000
314	2381.72060000	29.96200000	0.00000000
315	2937.25490000	52.61090000	0.00000000
316	2952.81880000	46.05070000	0.00000000

317	2957.08960000	21.49100000	0.00000000
318	2962.77300000	101.69090000	0.00000000
319	2964.13510000	44.90650000	0.00000000
320	2965.33490000	90.61760000	0.00000000
321	2972.23720000	87.65160000	0.00000000
322	2973.99320000	72.22920000	0.00000000
323	2985.40680000	24.93090000	0.00000000
324	2988.75980000	101.95380000	0.00000000
325	2992.52310000	77.82980000	0.00000000
326	3002.81810000	47.40190000	0.00000000
327	3005.63750000	38.72690000	0.00000000
328	3006.93390000	24.76400000	0.00000000
329	3011.66630000	45.68710000	0.00000000
330	3017.23950000	55.14440000	0.00000000
331	3019.14870000	64.75480000	0.00000000
332	3022.81420000	21.19530000	0.00000000
333	3025.67410000	28.87380000	0.00000000
334	3029.35240000	19.24720000	0.00000000
335	3031.11990000	13.51980000	0.00000000
336	3039.63880000	37.38510000	0.00000000
337	3041.61500000	30.37860000	0.00000000
338	3050.06480000	12.44750000	0.00000000
339	3050.49050000	7.06640000	0.00000000
340	3050.84960000	15.57730000	0.00000000
341	3060.79440000	21.30090000	0.00000000
342	3063.67940000	18.00030000	0.00000000
343	3067.89490000	14.06200000	0.00000000
344	3080.76550000	15.99850000	0.00000000
345	3081.15320000	47.46830000	0.00000000
346	3083.53010000	31.87210000	0.00000000
347	3103.45770000	26.02020000	0.00000000
348	3103.65200000	47.88450000	0.00000000
349	3103.77350000	25.03340000	0.00000000
350	3105.09660000	26.25740000	0.00000000
351	3106.28350000	35.62550000	0.00000000
352	3108.66010000	28.92140000	0.00000000
353	3116.41070000	22.53000000	0.00000000
354	3118.74070000	0.60590000	0.00000000
355	3125.54360000	1.40330000	0.00000000
356	3130.82630000	1.41720000	0.00000000
357	3163.98550000	2.64370000	0.00000000
358	3164.54700000	12.61040000	0.00000000
359	3165.62780000	6.16720000	0.00000000
360	3165.73190000	2.41170000	0.00000000
361	3178.76200000	15.53870000	0.00000000
362	3180.05380000	13.17000000	0.00000000
363	3182.67330000	15.40550000	0.00000000
364	3188.99400000	24.54460000	0.00000000
365	3189.10230000	11.86580000	0.00000000
366	3190.58480000	18.92070000	0.00000000
367	3196.61520000	11.59380000	0.00000000
368	3199.89830000	8.49300000	0.00000000
369	3203.18180000	6.41810000	0.00000000
370	3210.50830000	9.77040000	0.00000000
371	3210.76050000	9.17310000	0.00000000
372	3220.53390000	4.62370000	0.00000000

S6.8. Calculations on $Cu_2(1^*)_4(DMF)_2$

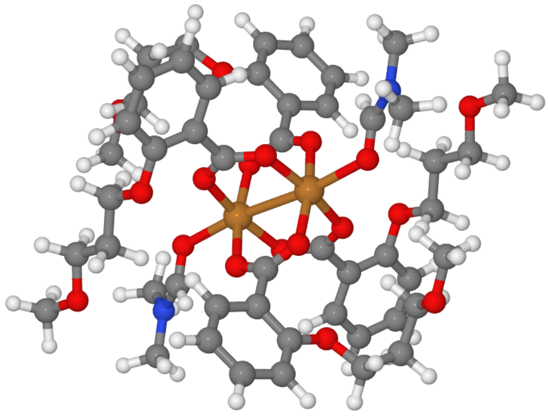

Route	: # opt freq b3lyp/genecp geom=connectivity int=ultrafine	
SMILES	: CN(C)[CH]O[Cu]1234O[C](O[Cu]1(O[C](O2)c5ccccc5OCCCOC)(O[C](O3)c6ccccc6OCCCOC)(O[C](O4)c7ccccc7OCCCOC)O[CH]N(C)C)c8ccccc8OCCCOC	
Formula	: $C_{50}H_{66}Cu_2N_2O_{18}$	
Charge	: 0	
Multiplicity	: 1	
Dipole	: 0.0006	Debye
Energy	: -3804.32795546	a.u.
Gibbs Energy	: -3803.35506000	a.u.
Number of imaginary frequencies	: 0	

S6.8.1. Cartesian Co-ordinates (XYZ format)

138

O	-2.88549590	0.64598697	2.04175591
O	-1.17017603	1.98147297	-0.14347699
O	0.73859900	1.72055495	-1.32382405
O	0.16515900	0.85116798	2.11006308
O	2.05620694	0.55752802	0.91169900
C	-0.28224599	2.35789609	-0.96060699
C	1.41476297	0.87241697	1.95011699
C	-3.93590593	1.37754798	4.53741789
H	-3.62277699	0.37583801	4.25214005
H	-5.01435280	1.38408601	4.72823095
H	-3.41726589	1.67295599	5.45453978
O	1.49230099	4.50388002	-0.69569403
O	3.83824110	-0.43514201	2.90241909
O	6.06240511	-2.67175794	-0.69149798
C	0.42312500	4.73905087	-1.49875605
O	4.74982500	3.75875711	-1.75559604
N	-3.59845090	2.29165792	3.45645499
C	3.40467000	0.70642501	3.53099990
C	-1.67483306	3.88903403	-2.36080098

H	-2.38814592	3.07672191	-2.43613505
C	-0.51752901	3.69939995	-1.61027205
C	1.76035404	2.44412804	3.87010288
H	0.83026803	2.91105890	3.57132292
C	2.22117090	1.34933901	3.13170409
C	2.59112096	5.42074203	-0.70002800
H	2.27969790	6.37883377	-0.26583999
H	2.92827797	5.58610487	-1.72751606
C	-3.07786894	1.83459496	2.29980707
H	-2.81940699	2.62610793	1.58561802
C	4.83194304	-0.23709200	1.87870800
H	5.77512407	0.07445900	2.34858489
H	4.49212503	0.54970098	1.20148206
C	4.99226284	-1.54835796	1.12892604
H	5.29026508	-2.33997703	1.82272303
H	4.02519178	-1.82246101	0.70311201
C	3.70897698	4.79996109	0.12342900
H	3.35560989	4.61875820	1.14317298
H	4.53131104	5.52115583	0.18044101
C	6.02274704	-1.43882799	0.01604600
H	5.75960112	-0.62217402	-0.67310703
H	7.02000713	-1.21846199	0.42972100
C	4.22320509	3.49227309	-0.46246400
H	5.00986290	3.07701612	0.18696100
H	3.41032100	2.75956297	-0.52389699
C	7.08397484	-2.70360708	-1.66451705
H	6.94106913	-1.92915499	-2.43285894
H	7.05236292	-3.68579888	-2.13841009
H	8.07707405	-2.56203294	-1.21367896
C	5.10931683	2.58031106	-2.44949889
H	5.93926907	2.05761194	-1.94867098
H	5.43668985	2.88011789	-3.44645405
H	4.26296091	1.88707101	-2.53384495
C	-3.83083296	3.70988798	3.66702795
H	-4.88813496	3.90114999	3.87605810
H	-3.55273604	4.26210499	2.76960111
H	-3.23787308	4.07968521	4.51022291
C	-1.89675498	5.08869791	-3.03566504
C	0.19331200	5.94517708	-2.16751909
H	0.90566802	6.75531197	-2.09115505
C	-0.95916802	6.10977221	-2.93544102
C	2.47912312	2.92696595	4.95789385
C	4.10809994	1.17821896	4.64084196
H	5.00023079	0.64508301	4.94848919
C	3.65549707	2.28808999	5.34637594
O	-0.73862797	-1.72056699	1.32370496
O	-2.05613399	-0.55729198	-0.91173601
O	2.88564491	-0.64582402	-2.04168296
O	1.17034698	-1.98134005	0.14365400
O	-0.16502699	-0.85128599	-2.10992002
C	0.28231201	-2.35783696	0.96063399
C	-1.41464806	-0.87239200	-1.95005906
C	3.93552303	-1.37739503	-4.53756809
H	3.62248898	-0.37567800	-4.25221300
H	5.01392603	-1.38397098	-4.72862911
H	3.41665912	-1.67277896	-5.45457077
O	-1.49228203	-4.50377607	0.69574201
O	-3.83827806	0.43486601	-2.90245795
O	-6.06307793	2.67152691	0.69105601
C	-0.42308900	-4.73898315	1.49877501
O	-4.74962902	-3.75841808	1.75580704
N	3.59829307	-2.29150295	-3.45653105
C	-3.40456295	-0.70668298	-3.53096604
C	1.67488801	-3.88900089	2.36081004

H	2.38822007	-3.07670307	2.43613601
C	0.51758200	-3.69934797	1.61029100
C	-1.76009095	-2.44426298	-3.86993694
H	-0.82997799	-2.91110206	-3.57109904
C	-2.22100496	-1.34945595	-3.13162303
C	-2.59114099	-5.42059088	0.70011097
H	-2.27977800	-6.37868881	0.26589301
H	-2.92825794	-5.58595800	1.72761202
C	3.07797790	-1.83443403	-2.29976702
H	2.81971002	-2.62594795	-1.58551002
C	-4.83195114	0.23674899	-1.87873602
H	-5.77509403	-0.07493700	-2.34860492
H	-4.49205112	-0.54997301	-1.20146799
C	-4.99245882	1.54803705	-1.12903094
H	-5.29041815	2.33959699	-1.82291400
H	-4.02547407	1.82222795	-0.70308203
C	-3.70900989	-4.79974508	-0.12328100
H	-3.35570598	-4.61858082	-1.14305401
H	-4.53140020	-5.52088022	-0.18022200
C	-6.02311993	1.43850803	-0.01631400
H	-5.75998688	0.62198102	0.67299497
H	-7.02027512	1.21794701	-0.43013999
C	-4.22309923	-3.49200797	0.46262199
H	-5.00978279	-3.07671309	-0.18674400
H	-3.41015697	-2.75935411	0.52396703
C	-7.08490086	2.70341492	1.66380596
H	-6.94213820	1.92906594	2.43227911
H	-7.05349016	3.68566608	2.13758993
H	-8.07787323	2.56170702	1.21272898
C	-5.10891819	-2.57992101	2.44972396
H	-5.93896198	-2.05721593	1.94905603
H	-5.43607283	-2.87965894	3.44676995
H	-4.26251507	-1.88670599	2.53383994
C	3.83067894	-3.70972800	-3.66713405
H	4.88794899	-3.90096211	-3.87635398
H	3.55275512	-4.26194715	-2.76965499
H	3.23757696	-4.07954407	-4.51021910
C	1.89679897	-5.08867598	3.03566003
C	-0.19328301	-5.94512320	2.16751504
H	-0.90564197	-6.75525284	2.09114003
C	0.95920300	-6.10973978	2.93542600
C	-2.47880602	-2.92724299	-4.95769882
C	-4.10793877	-1.17861998	-4.64078617
H	-5.00012302	-0.64559001	-4.94846582
C	-3.65522599	-2.28849006	-5.34624815
H	4.21337080	2.64476299	6.20505285
H	1.11883104	-7.04959202	3.45250297
H	-4.21308517	-2.64529991	-6.20487595
H	-1.11879897	7.04961491	-3.45253611
H	-2.79211497	5.21791410	-3.63179898
H	-2.11447406	-3.78600597	-5.50964117
H	2.79215908	-5.21790791	3.63178992
H	2.11486197	3.78571010	5.50991297
Cu	1.10922098	-0.15113500	-0.70757401
Cu	-1.10913503	0.15120600	0.70763600

S6.8.2. Frequencies

Mode	IR frequency	IR intensity	Raman intensity
1	9.36000000	1.60200000	0.00000000
2	10.82910000	0.00060000	0.00000000
3	11.00470000	0.81970000	0.00000000
4	13.94360000	0.00000000	0.00000000
5	14.68750000	0.54620000	0.00000000
6	15.61070000	0.54250000	0.00000000
7	16.43240000	1.68800000	0.00000000
8	16.65370000	0.00010000	0.00000000
9	18.52910000	0.39530000	0.00000000
10	20.27990000	0.00000000	0.00000000
11	20.39640000	0.00440000	0.00000000
12	22.38920000	0.00000000	0.00000000
13	26.52960000	1.81600000	0.00000000
14	27.93140000	0.00090000	0.00000000
15	28.11820000	0.58080000	0.00000000
16	32.11530000	0.00000000	0.00000000
17	33.39160000	1.26750000	0.00000000
18	34.11070000	0.00000000	0.00000000
19	34.43020000	1.15000000	0.00000000
20	37.65810000	0.14170000	0.00000000
21	44.17910000	0.00000000	0.00000000
22	47.31720000	2.10280000	0.00000000
23	47.90030000	0.00640000	0.00000000
24	52.74260000	0.00000000	0.00000000
25	54.76120000	0.09740000	0.00000000
26	60.46380000	0.00000000	0.00000000
27	61.28670000	2.10320000	0.00000000
28	64.89070000	0.00510000	0.00000000
29	65.27180000	11.32730000	0.00000000
30	68.57960000	0.00000000	0.00000000
31	73.36350000	0.50410000	0.00000000
32	75.27210000	0.00000000	0.00000000
33	77.75250000	0.00000000	0.00000000
34	81.40050000	0.03050000	0.00000000
35	83.61600000	0.00000000	0.00000000
36	84.27520000	4.24720000	0.00000000
37	88.65270000	4.40580000	0.00000000
38	92.81860000	1.54120000	0.00000000
39	94.66110000	0.00000000	0.00000000
40	95.91790000	0.00000000	0.00000000
41	102.91840000	0.00030000	0.00000000
42	103.41720000	8.07300000	0.00000000
43	104.19700000	0.00000000	0.00000000
44	110.05190000	1.60230000	0.00000000
45	110.21460000	0.00000000	0.00000000
46	111.74250000	0.21970000	0.00000000
47	114.34920000	0.00000000	0.00000000
48	120.34970000	4.20500000	0.00000000
49	121.94770000	0.00000000	0.00000000
50	129.55460000	0.00000000	0.00000000
51	130.19000000	0.60140000	0.00000000
52	131.57360000	0.00000000	0.00000000
53	137.57020000	0.00000000	0.00000000
54	140.63930000	0.00010000	0.00000000
55	142.63600000	15.19500000	0.00000000
56	145.51370000	2.87140000	0.00000000
57	146.92240000	15.56750000	0.00000000
58	153.72640000	0.00000000	0.00000000
59	155.97590000	12.44360000	0.00000000
60	156.30180000	0.00000000	0.00000000

61	173.33100000	0.00080000	0.00000000
62	173.60370000	1.31760000	0.00000000
63	179.79700000	1.69900000	0.00000000
64	184.97780000	0.00000000	0.00000000
65	189.75540000	0.00000000	0.00000000
66	190.75710000	2.40440000	0.00000000
67	196.07460000	0.00000000	0.00000000
68	199.85180000	16.38090000	0.00000000
69	202.65110000	0.00000000	0.00000000
70	205.88160000	7.66060000	0.00000000
71	206.58230000	0.00000000	0.00000000
72	209.10860000	0.00000000	0.00000000
73	220.49790000	6.19380000	0.00000000
74	228.99120000	0.00000000	0.00000000
75	230.55340000	6.35900000	0.00000000
76	230.68470000	0.00070000	0.00000000
77	234.13890000	14.79550000	0.00000000
78	235.34250000	7.55100000	0.00000000
79	236.59190000	0.00020000	0.00000000
80	243.96190000	0.26410000	0.00000000
81	244.13720000	0.00010000	0.00000000
82	246.96380000	13.94780000	0.00000000
83	247.24560000	0.00070000	0.00000000
84	259.52860000	7.81330000	0.00000000
85	268.33730000	0.00000000	0.00000000
86	276.13300000	12.26370000	0.00000000
87	285.75220000	11.32120000	0.00000000
88	287.67760000	0.00010000	0.00000000
89	294.17010000	0.00000000	0.00000000
90	299.61820000	14.24940000	0.00000000
91	303.39480000	10.62440000	0.00000000
92	308.06130000	0.00000000	0.00000000
93	318.87940000	0.00000000	0.00000000
94	321.12190000	0.94540000	0.00000000
95	342.26830000	0.02290000	0.00000000
96	342.33140000	33.25680000	0.00000000
97	372.20820000	69.83190000	0.00000000
98	372.47500000	0.04640000	0.00000000
99	390.98680000	0.22210000	0.00000000
100	394.54730000	0.00000000	0.00000000
101	400.21130000	0.00060000	0.00000000
102	400.22820000	3.45560000	0.00000000
103	402.31630000	4.65580000	0.00000000
104	403.81780000	0.00000000	0.00000000
105	405.38080000	13.42410000	0.00000000
106	411.10080000	0.00000000	0.00000000
107	416.75590000	10.35140000	0.00000000
108	427.93040000	0.00000000	0.00000000
109	437.51460000	0.00000000	0.00000000
110	439.14590000	8.50950000	0.00000000
111	443.13690000	0.00000000	0.00000000
112	449.60850000	22.83250000	0.00000000
113	467.55600000	0.00000000	0.00000000
114	470.58390000	9.74270000	0.00000000
115	471.91230000	0.00000000	0.00000000
116	480.61360000	19.18580000	0.00000000
117	495.25720000	0.00000000	0.00000000
118	500.80510000	49.48600000	0.00000000
119	523.60920000	8.25910000	0.00000000
120	528.11790000	0.00000000	0.00000000
121	542.23950000	0.00030000	0.00000000
122	542.69690000	6.59540000	0.00000000
123	554.69380000	12.65720000	0.00000000
124	556.20070000	0.00000000	0.00000000

125	585.39780000	1.79480000	0.00000000
126	598.28880000	8.39800000	0.00000000
127	599.06950000	0.00000000	0.00000000
128	601.56940000	0.00000000	0.00000000
129	620.64820000	12.83110000	0.00000000
130	620.91830000	0.00010000	0.00000000
131	622.34380000	6.84710000	0.00000000
132	622.51490000	0.00020000	0.00000000
133	669.51030000	46.86060000	0.00000000
134	669.69050000	0.00520000	0.00000000
135	679.47870000	55.42960000	0.00000000
136	680.26770000	0.00000000	0.00000000
137	688.00640000	69.64500000	0.00000000
138	691.79130000	0.00000000	0.00000000
139	733.11440000	0.00100000	0.00000000
140	733.95460000	36.39170000	0.00000000
141	744.48080000	17.32020000	0.00000000
142	745.16090000	0.00120000	0.00000000
143	763.88900000	0.01200000	0.00000000
144	763.95310000	95.64530000	0.00000000
145	777.37350000	25.12320000	0.00000000
146	777.49470000	0.00000000	0.00000000
147	780.09210000	64.56280000	0.00000000
148	780.28740000	0.00300000	0.00000000
149	788.85130000	4.44650000	0.00000000
150	788.92480000	0.00020000	0.00000000
151	797.28710000	0.00000000	0.00000000
152	798.92710000	8.94130000	0.00000000
153	809.61500000	9.73340000	0.00000000
154	810.55290000	0.00000000	0.00000000
155	830.74380000	9.60030000	0.00000000
156	832.33370000	0.00000000	0.00000000
157	839.20230000	8.89100000	0.00000000
158	841.53740000	0.00000000	0.00000000
159	864.29070000	2.51070000	0.00000000
160	864.33420000	0.00050000	0.00000000
161	869.71090000	12.50880000	0.00000000
162	870.36680000	0.00000000	0.00000000
163	872.39780000	8.05430000	0.00000000
164	874.26190000	0.00000000	0.00000000
165	874.62270000	4.06660000	0.00000000
166	879.11890000	0.00000000	0.00000000
167	892.11820000	10.08700000	0.00000000
168	892.45120000	0.00010000	0.00000000
169	896.99080000	3.84860000	0.00000000
170	897.20810000	0.00010000	0.00000000
171	899.70530000	3.39900000	0.00000000
172	899.76110000	0.00040000	0.00000000
173	945.52590000	32.61020000	0.00000000
174	945.54190000	0.04040000	0.00000000
175	957.44530000	2.24330000	0.00000000
176	957.45740000	0.11520000	0.00000000
177	959.51140000	29.11830000	0.00000000
178	959.53820000	0.06420000	0.00000000
179	965.78640000	0.00250000	0.00000000
180	965.86870000	11.23750000	0.00000000
181	983.14880000	1.35620000	0.00000000
182	983.16660000	0.03290000	0.00000000
183	988.31160000	0.49880000	0.00000000
184	988.34130000	0.00150000	0.00000000
185	998.12850000	0.00230000	0.00000000
186	998.21540000	71.67300000	0.00000000
187	1027.12150000	0.03490000	0.00000000
188	1027.25000000	212.82990000	0.00000000

189	1036.82280000	5.77040000	0.00000000
190	1036.90880000	0.02190000	0.00000000
191	1059.38380000	27.02620000	0.00000000
192	1059.41080000	0.02400000	0.00000000
193	1064.73630000	0.00040000	0.00000000
194	1064.78810000	23.67440000	0.00000000
195	1065.64500000	83.97610000	0.00000000
196	1065.82750000	0.00170000	0.00000000
197	1069.02650000	35.76610000	0.00000000
198	1069.03040000	0.00000000	0.00000000
199	1080.10220000	15.67330000	0.00000000
200	1080.12380000	0.02110000	0.00000000
201	1096.72120000	12.13650000	0.00000000
202	1096.76550000	0.00340000	0.00000000
203	1106.12540000	0.00750000	0.00000000
204	1106.16450000	26.34570000	0.00000000
205	1109.53470000	235.04470000	0.00000000
206	1110.26830000	0.00040000	0.00000000
207	1114.99820000	105.07740000	0.00000000
208	1115.18950000	0.00030000	0.00000000
209	1117.97820000	24.60700000	0.00000000
210	1118.00890000	0.00220000	0.00000000
211	1124.90720000	159.01310000	0.00000000
212	1125.57220000	0.00000000	0.00000000
213	1127.18870000	3.41450000	0.00000000
214	1127.21990000	0.00090000	0.00000000
215	1145.85410000	257.25940000	0.00000000
216	1146.00980000	0.06950000	0.00000000
217	1149.46760000	0.04860000	0.00000000
218	1149.65600000	242.49600000	0.00000000
219	1161.53230000	0.08100000	0.00000000
220	1161.54990000	7.23560000	0.00000000
221	1166.56580000	0.00060000	0.00000000
222	1166.63520000	16.94280000	0.00000000
223	1171.01480000	28.05450000	0.00000000
224	1171.63680000	0.00000000	0.00000000
225	1175.20150000	0.99230000	0.00000000
226	1175.20380000	0.42000000	0.00000000
227	1178.99140000	1.94470000	0.00000000
228	1178.99260000	9.82440000	0.00000000
229	1180.90390000	17.12650000	0.00000000
230	1181.20380000	0.00000000	0.00000000
231	1183.26170000	0.01510000	0.00000000
232	1183.29770000	9.84010000	0.00000000
233	1185.70780000	12.88830000	0.00000000
234	1185.78310000	0.00010000	0.00000000
235	1217.95960000	39.00040000	0.00000000
236	1217.97700000	0.31780000	0.00000000
237	1220.96670000	31.40480000	0.00000000
238	1220.96750000	6.20050000	0.00000000
239	1238.62750000	35.51300000	0.00000000
240	1238.68480000	0.01120000	0.00000000
241	1251.96700000	188.93360000	0.00000000
242	1251.98210000	0.76870000	0.00000000
243	1258.67590000	47.30650000	0.00000000
244	1258.79210000	0.00830000	0.00000000
245	1264.76900000	0.00500000	0.00000000
246	1264.80190000	11.33280000	0.00000000
247	1277.82970000	0.41830000	0.00000000
248	1277.85200000	51.82060000	0.00000000
249	1279.64350000	341.35300000	0.00000000
250	1279.70700000	0.41100000	0.00000000
251	1283.01340000	0.00190000	0.00000000
252	1283.06120000	17.67860000	0.00000000

253	1304.73870000	0.12040000	0.00000000
254	1304.74560000	2.74350000	0.00000000
255	1307.63970000	0.00010000	0.00000000
256	1307.78000000	69.90860000	0.00000000
257	1315.70610000	6.97160000	0.00000000
258	1315.71770000	0.26620000	0.00000000
259	1319.41960000	6.10680000	0.00000000
260	1319.51390000	0.00040000	0.00000000
261	1321.18450000	6.79560000	0.00000000
262	1321.19220000	0.00800000	0.00000000
263	1322.12230000	0.00060000	0.00000000
264	1322.17930000	2.65000000	0.00000000
265	1330.81540000	0.04360000	0.00000000
266	1330.85950000	59.75080000	0.00000000
267	1388.44580000	0.00000000	0.00000000
268	1390.54900000	15.87430000	0.00000000
269	1390.94410000	0.00000000	0.00000000
270	1398.30000000	250.97250000	0.00000000
271	1398.49010000	336.06580000	0.00000000
272	1404.50400000	0.00000000	0.00000000
273	1414.63970000	0.06320000	0.00000000
274	1414.64330000	16.90840000	0.00000000
275	1417.50120000	214.07480000	0.00000000
276	1418.58540000	0.00230000	0.00000000
277	1425.45670000	8.69990000	0.00000000
278	1425.70550000	0.00000000	0.00000000
279	1433.88990000	31.38020000	0.00000000
280	1433.91620000	0.09040000	0.00000000
281	1438.09320000	17.34080000	0.00000000
282	1438.23680000	0.00000000	0.00000000
283	1438.55960000	0.00120000	0.00000000
284	1438.68680000	120.33360000	0.00000000
285	1445.84790000	35.26920000	0.00000000
286	1445.88100000	0.00190000	0.00000000
287	1465.63500000	11.52060000	0.00000000
288	1465.65050000	0.05400000	0.00000000
289	1470.32910000	38.09070000	0.00000000
290	1470.40600000	0.00100000	0.00000000
291	1474.65490000	22.79820000	0.00000000
292	1475.54700000	65.97350000	0.00000000
293	1476.67990000	0.00000000	0.00000000
294	1476.96350000	0.00000000	0.00000000
295	1478.39370000	1.70190000	0.00000000
296	1478.39630000	3.01790000	0.00000000
297	1480.86480000	0.00150000	0.00000000
298	1480.91720000	8.67890000	0.00000000
299	1481.34370000	4.69350000	0.00000000
300	1481.51800000	0.00000000	0.00000000
301	1483.29820000	12.94250000	0.00000000
302	1483.30230000	1.28850000	0.00000000
303	1487.23760000	0.87960000	0.00000000
304	1487.25590000	18.37720000	0.00000000
305	1497.27930000	268.57270000	0.00000000
306	1499.40170000	0.00040000	0.00000000
307	1499.47230000	10.87970000	0.00000000
308	1502.15760000	0.00350000	0.00000000
309	1502.32420000	93.81940000	0.00000000
310	1503.36800000	39.18010000	0.00000000
311	1503.52540000	0.00010000	0.00000000
312	1504.70710000	6.46520000	0.00000000
313	1504.75630000	0.00100000	0.00000000
314	1506.50620000	0.01070000	0.00000000
315	1506.54320000	34.93070000	0.00000000
316	1507.59200000	0.00110000	0.00000000

317	1507.63120000	85.38900000	0.00000000
318	1509.76500000	17.58890000	0.00000000
319	1509.94790000	0.00030000	0.00000000
320	1516.57370000	60.35390000	0.00000000
321	1516.75560000	0.00080000	0.00000000
322	1521.40400000	73.13730000	0.00000000
323	1527.00230000	0.00000000	0.00000000
324	1527.69620000	63.86750000	0.00000000
325	1530.32370000	0.00000000	0.00000000
326	1531.20590000	5.03560000	0.00000000
327	1531.57940000	0.00000000	0.00000000
328	1533.11630000	33.37930000	0.00000000
329	1542.21600000	0.00010000	0.00000000
330	1542.64850000	30.47100000	0.00000000
331	1550.51480000	0.00000000	0.00000000
332	1562.69400000	0.00000000	0.00000000
333	1612.88600000	18.38620000	0.00000000
334	1613.37890000	0.00000000	0.00000000
335	1623.31830000	20.69840000	0.00000000
336	1623.34990000	0.00730000	0.00000000
337	1634.35080000	85.04900000	0.00000000
338	1634.82950000	0.00000000	0.00000000
339	1636.87060000	29.34270000	0.00000000
340	1637.35470000	0.00000000	0.00000000
341	1706.35370000	1416.92630000	0.00000000
342	1709.54650000	0.00220000	0.00000000
343	2947.65800000	1.31330000	0.00000000
344	2947.67390000	51.02340000	0.00000000
345	2953.16860000	2.94240000	0.00000000
346	2953.17790000	44.70070000	0.00000000
347	2961.68250000	0.13500000	0.00000000
348	2961.80570000	238.92670000	0.00000000
349	2963.29970000	166.17100000	0.00000000
350	2963.34060000	0.68380000	0.00000000
351	2983.53510000	0.35070000	0.00000000
352	2983.55630000	78.99670000	0.00000000
353	2985.77300000	179.29360000	0.00000000
354	2985.87280000	0.46820000	0.00000000
355	3001.47880000	94.44750000	0.00000000
356	3001.51310000	0.01470000	0.00000000
357	3001.70510000	131.55450000	0.00000000
358	3001.72220000	11.78730000	0.00000000
359	3012.76400000	22.57170000	0.00000000
360	3012.76680000	31.01700000	0.00000000
361	3018.34620000	170.16180000	0.00000000
362	3018.46010000	0.00710000	0.00000000
363	3023.73950000	23.30680000	0.00000000
364	3023.75150000	43.12780000	0.00000000
365	3032.10780000	29.09850000	0.00000000
366	3032.11140000	1.87430000	0.00000000
367	3043.07180000	51.73910000	0.00000000
368	3043.09150000	0.04850000	0.00000000
369	3046.66230000	0.08360000	0.00000000
370	3046.67320000	32.49240000	0.00000000
371	3052.04330000	37.91590000	0.00000000
372	3052.07280000	2.53330000	0.00000000
373	3061.43600000	0.08220000	0.00000000
374	3061.46300000	94.84770000	0.00000000
375	3062.63480000	33.41030000	0.00000000
376	3062.63640000	2.73070000	0.00000000
377	3067.16310000	0.50460000	0.00000000
378	3067.17330000	24.51480000	0.00000000
379	3078.70720000	0.79960000	0.00000000
380	3078.71410000	22.70900000	0.00000000

381	3081.03560000	76.18040000	0.00000000
382	3081.04800000	0.85870000	0.00000000
383	3100.11350000	77.03780000	0.00000000
384	3100.13020000	0.02780000	0.00000000
385	3102.27980000	3.93600000	0.00000000
386	3102.28730000	60.13170000	0.00000000
387	3106.76030000	37.76470000	0.00000000
388	3106.77530000	5.14180000	0.00000000
389	3126.70120000	10.19260000	0.00000000
390	3126.70800000	9.36030000	0.00000000
391	3153.79920000	0.57040000	0.00000000
392	3153.80440000	0.50700000	0.00000000
393	3163.92720000	3.47740000	0.00000000
394	3163.93050000	0.00070000	0.00000000
395	3164.12080000	14.29640000	0.00000000
396	3164.12570000	0.05070000	0.00000000
397	3177.85500000	32.29990000	0.00000000
398	3177.85650000	0.04580000	0.00000000
399	3180.34850000	26.78640000	0.00000000
400	3180.35710000	1.23430000	0.00000000
401	3188.21710000	37.74930000	0.00000000
402	3188.24280000	0.00490000	0.00000000
403	3193.12150000	25.97910000	0.00000000
404	3193.14220000	0.37320000	0.00000000
405	3199.17690000	18.70280000	0.00000000
406	3199.21130000	0.16590000	0.00000000
407	3205.82480000	23.73660000	0.00000000
408	3205.85070000	0.00660000	0.00000000

S6.9. Calculations on $Cu_2(2^*)_4(DMF)_2$

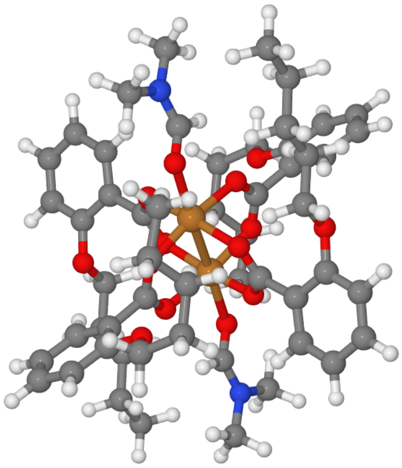

Route	:	# opt freq b3lyp/genecp geom=connectivity int=ultrafine	
SMILES	:	CCCCCOc1ccccc1[C]2O[Cu]345(O[C](O[Cu]3(O2)(O[C](O4)c6ccccc6OCCCCC)(O[C](O5)c7ccccc7OCCCCC)O[CH]N(C)C)c8ccccc8OCCCCC)O[CH]N(C)C	
Formula	:	$C_{54}H_{74}Cu_2N_2O_{14}$	
Charge	:	0	
Multiplicity	:	1	
Dipole	:	0.0643	Debye
Energy	:	-3660.73306107	a.u.
Gibbs Energy	:	-3659.66549300	a.u.
Number of imaginary frequencies	:	1	

S6.9.1. Cartesian Co-ordinates (XYZ format)

146

O	2.84180689	0.50237900	-2.13468790
O	1.28901303	1.89794099	0.09978400
O	-0.58052498	1.76649904	1.36200500
O	-0.19528100	0.87845898	-2.09369206
O	-2.05331111	0.68421799	-0.82536203
C	0.46533701	2.33107901	0.95528901
C	-1.43466198	0.97704899	-1.88313794
C	4.32447481	1.13175297	-4.43558693
H	4.09276819	0.12848100	-4.08618689
H	5.40993214	1.27285802	-4.44916105
H	3.94151998	1.26009202	-5.45291185
O	-1.16225100	4.60863113	0.79590797
O	-3.98767996	-0.13181999	-2.76489210
C	-0.02948400	4.76043606	1.53002501
N	3.69572806	2.09088707	-3.53854609
C	-3.49534798	0.99022299	-3.38207793
C	2.03743410	3.74529505	2.28127694
H	2.68566608	2.87760401	2.32057500
C	0.83013099	3.64880896	1.59427202

C	-1.74024904	2.60843492	-3.75663090
H	-0.76623303	2.99728894	-3.48782301
C	-2.25129890	1.53328300	-3.02187991
C	-2.14691806	5.64493084	0.80158597
H	-1.73355103	6.55363178	0.34566399
H	-2.42368388	5.88023996	1.83590698
C	2.98724198	1.67753196	-2.46662688
H	2.53541303	2.49399090	-1.88794994
C	-4.97317314	0.10346500	-1.73779297
H	-5.82647181	0.63993299	-2.17442608
H	-4.52563906	0.71991599	-0.95442402
C	-5.40676212	-1.24317002	-1.18584800
H	-5.76645899	-1.86520898	-2.01355505
H	-4.52725506	-1.74135101	-0.76657999
C	-3.34840703	5.15632677	0.00435300
H	-3.03523397	5.00484610	-1.03364599
H	-4.08503199	5.96996212	-0.00425300
C	-6.49595404	-1.11166298	-0.11580300
H	-6.13358498	-0.46653599	0.69369501
H	-7.37269115	-0.60679197	-0.54246402
C	-3.99228811	3.86500692	0.53066301
H	-4.80178213	3.58852911	-0.15561301
H	-3.25779605	3.05645394	0.48547301
C	-8.03457355	-2.32969499	1.52632499
H	-7.70962715	-1.70284295	2.36296105
H	-8.31975842	-3.30512500	1.93066597
H	-8.93412495	-1.87090099	1.10420895
C	-5.26848507	2.67326498	2.38645601
H	-6.12330294	2.45784402	1.73671901
H	-5.64330721	2.75122190	3.41159296
H	-4.58738899	1.81900799	2.33624506
C	3.81819510	3.50174999	-3.86351705
H	4.86960888	3.80658197	-3.88049698
H	3.29632401	4.09685087	-3.11356497
H	3.38076305	3.71304393	-4.84503412
C	2.39230895	4.92334080	2.93723011
C	0.33588901	5.94603920	2.17569089
H	-0.30847400	6.81337118	2.13138199
C	1.53806198	6.01787901	2.87942696
C	-2.46449399	3.17166090	-4.80078793
C	-4.20670795	1.54332197	-4.44969702
H	-5.14899302	1.08691299	-4.73018789
C	-3.70208001	2.63332796	-5.14996576
O	0.57092398	-1.75837600	-1.37469804
O	2.05099797	-0.70339298	0.82144397
O	-2.84173012	-0.50099999	2.13646102
O	-1.27728605	-1.90689099	-0.08330200
O	0.19853400	-0.86007297	2.10346508
C	-0.46480399	-2.33107996	-0.95364702
C	1.43499899	-0.97759199	1.88560295
C	-4.32847118	-1.12346601	4.43658209
H	-4.09572077	-0.12125000	4.08484888
H	-5.41401196	-1.26407599	4.44860506
H	-3.94739509	-1.24917996	5.45493889
O	1.16369402	-4.60612917	-0.79922903
O	3.98802996	0.12800699	2.76204705
C	0.02928900	-4.75936317	-1.53076506
N	-3.69852090	-2.08530903	3.54329491
C	3.49658489	-0.99318200	3.38164401
C	-2.03926802	-3.74481297	-2.27807903
H	-2.68779898	-2.87727499	-2.31663108
C	-0.83090597	-3.64829707	-1.59321499
C	1.73928201	-2.60792804	3.76077795
H	0.76410198	-2.99554110	3.49424005

C	2.25126600	-1.53463399	3.02422690
C	2.14841104	-5.64237022	-0.80552000
H	1.73552501	-6.55082607	-0.34868100
H	2.42394805	-5.87819481	-1.84005594
C	-2.98812699	-1.67519104	2.47138000
H	-2.53555894	-2.49338198	1.89573300
C	4.97342920	-0.10820000	1.73505604
H	5.82652712	-0.64487499	2.17180395
H	4.52553082	-0.72487497	0.95203900
C	5.40752602	1.23808706	1.18262696
H	5.76806307	1.86000395	2.01006389
H	4.52803421	1.73673701	0.76387501
C	3.35092711	-5.15359116	-0.00995300
H	3.03916001	-5.00188923	1.02843201
H	4.08748388	-5.96730423	-0.00219500
C	6.49607277	1.10575795	0.11203400
H	6.13280010	0.46096599	-0.69731802
H	7.37262106	0.60017198	0.53824502
C	3.99430609	-3.86245108	-0.53729600
H	4.80541992	-3.58664489	0.14734800
H	3.26042891	-3.05346704	-0.49007601
C	8.03502083	2.32262707	-1.53064895
H	7.70930004	1.69600403	-2.36715293
H	8.32079601	3.29783797	-1.93510902
H	8.93438244	1.86316395	-1.10885596
C	5.26743221	-2.67093110	-2.39534211
H	6.12381887	-2.45642805	-1.74737298
H	5.63995790	-2.74872398	-3.42132998
H	4.58705711	-1.81621099	-2.34329200
C	-3.82217503	-3.49523091	3.87187910
H	-4.87375021	-3.79955912	3.88785601
H	-3.29927301	-4.09259176	3.12444401
H	-3.38654494	-3.70406103	4.85472393
C	-2.39495707	-4.92312813	-2.93327594
C	-0.33669099	-5.94511604	-2.17568207
H	0.30820301	-6.81212997	-2.13279104
C	-1.54029500	-6.01739502	-2.87702799
C	2.46463394	-3.17138696	4.80413914
C	4.20915318	-1.54636800	4.44832993
H	5.15267897	-1.09130895	4.72685194
C	3.70384598	-2.63494992	5.15044212
H	-4.26739979	3.05210996	-5.97514677
H	-1.80452394	-6.94270515	-3.37718606
H	4.26991606	-3.05400205	5.97496605
H	1.80162299	6.94310379	3.38010311
H	3.32524109	4.97922993	3.48522305
H	2.05899096	-4.01256609	5.35439014
H	-3.32887697	-4.97933102	-3.47955799
H	-2.05927896	4.01411200	-5.34940481
Cu	-1.08603203	-0.07805300	0.75393403
Cu	1.08711600	0.07767500	-0.75150102
C	4.55629921	-3.95646310	-1.96098304
H	3.74396205	-4.16380882	-2.66580796
H	5.25172806	-4.80371904	-2.02930093
C	-6.93241692	-2.45893407	0.47114101
H	-6.05970812	-2.95696998	0.91027898
H	-7.27771711	-3.11196995	-0.33927399
C	-4.55737686	3.95914602	1.95310903
H	-5.25357008	4.80592012	2.01958203
H	-3.74671507	4.16738892	2.65960097
C	6.93335819	2.45270205	-0.47505000
H	7.27946377	3.10546207	0.33524600
H	6.06086683	2.95140791	-0.91386199

S6.9.2. Frequencies

Mode	IR frequency	IR intensity	Raman intensity
1	-4.42010000	1.27570000	0.00000000
2	9.76210000	0.00030000	0.00000000
3	10.63720000	1.20550000	0.00000000
4	11.78900000	0.40170000	0.00000000
5	14.53050000	0.00060000	0.00000000
6	14.93460000	0.28570000	0.00000000
7	15.67410000	0.00600000	0.00000000
8	16.29340000	1.46950000	0.00000000
9	18.39590000	1.50730000	0.00000000
10	19.48770000	0.06560000	0.00000000
11	20.28850000	0.00120000	0.00000000
12	21.21880000	0.00030000	0.00000000
13	22.45870000	0.81160000	0.00000000
14	24.66670000	0.00010000	0.00000000
15	26.94160000	1.82570000	0.00000000
16	29.56730000	0.29490000	0.00000000
17	29.94490000	0.00070000	0.00000000
18	31.83250000	0.00020000	0.00000000
19	32.67340000	0.35070000	0.00000000
20	33.90400000	1.46730000	0.00000000
21	34.15730000	0.00980000	0.00000000
22	38.75450000	0.26750000	0.00000000
23	42.11520000	0.00060000	0.00000000
24	44.30990000	0.39970000	0.00000000
25	47.72680000	0.00010000	0.00000000
26	53.93510000	0.00000000	0.00000000
27	57.08350000	0.11510000	0.00000000
28	59.33090000	0.00010000	0.00000000
29	63.72260000	0.38270000	0.00000000
30	70.19900000	0.00020000	0.00000000
31	74.08030000	0.65060000	0.00000000
32	74.73320000	0.00170000	0.00000000
33	77.07500000	5.08930000	0.00000000
34	78.96030000	0.00020000	0.00000000
35	83.27580000	0.00130000	0.00000000
36	85.69900000	0.51720000	0.00000000
37	87.42220000	0.00010000	0.00000000
38	87.85160000	0.69180000	0.00000000
39	89.07030000	1.77120000	0.00000000
40	94.28180000	0.00020000	0.00000000
41	95.64380000	3.02480000	0.00000000
42	98.97390000	0.00000000	0.00000000
43	105.79310000	0.00310000	0.00000000
44	109.05600000	1.51740000	0.00000000
45	111.12130000	0.00010000	0.00000000
46	112.30820000	0.00130000	0.00000000
47	113.88500000	0.99390000	0.00000000
48	120.81200000	0.00890000	0.00000000
49	121.96600000	3.29540000	0.00000000
50	124.75830000	6.42350000	0.00000000
51	127.18990000	0.00200000	0.00000000
52	127.93000000	0.00410000	0.00000000
53	137.04580000	1.52060000	0.00000000
54	137.11010000	7.88200000	0.00000000
55	138.34590000	0.00210000	0.00000000
56	142.30010000	1.33110000	0.00000000
57	149.63060000	10.98010000	0.00000000
58	151.22370000	0.00080000	0.00000000
59	160.16390000	6.47700000	0.00000000
60	162.07870000	0.04420000	0.00000000

61	170.62380000	0.02510000	0.00000000
62	170.73420000	0.28130000	0.00000000
63	185.02290000	2.70830000	0.00000000
64	187.00470000	0.00100000	0.00000000
65	187.91970000	1.52790000	0.00000000
66	193.80480000	0.00020000	0.00000000
67	197.27380000	0.00440000	0.00000000
68	198.48990000	0.00550000	0.00000000
69	200.08120000	18.70370000	0.00000000
70	207.05180000	0.02190000	0.00000000
71	207.57820000	0.00450000	0.00000000
72	208.96110000	4.66360000	0.00000000
73	219.39120000	4.94350000	0.00000000
74	224.55610000	0.01950000	0.00000000
75	228.76060000	15.23460000	0.00000000
76	231.06320000	0.02690000	0.00000000
77	234.09990000	7.62400000	0.00000000
78	243.30740000	0.00310000	0.00000000
79	243.36930000	0.44330000	0.00000000
80	244.82920000	0.92770000	0.00000000
81	244.91530000	0.01160000	0.00000000
82	252.58400000	5.01630000	0.00000000
83	260.72640000	0.00150000	0.00000000
84	263.55130000	0.00070000	0.00000000
85	264.25260000	1.87380000	0.00000000
86	268.60460000	6.85240000	0.00000000
87	282.77120000	12.36670000	0.00000000
88	284.68830000	0.09710000	0.00000000
89	293.23940000	0.00350000	0.00000000
90	294.39550000	2.66310000	0.00000000
91	298.88880000	8.04600000	0.00000000
92	300.57440000	0.01720000	0.00000000
93	301.65200000	0.03560000	0.00000000
94	304.66620000	8.69140000	0.00000000
95	346.74410000	40.03460000	0.00000000
96	346.88750000	0.13290000	0.00000000
97	364.78100000	56.97300000	0.00000000
98	364.89540000	1.18650000	0.00000000
99	381.88260000	0.00060000	0.00000000
100	386.80890000	1.58310000	0.00000000
101	392.15460000	0.65740000	0.00000000
102	393.41050000	0.00490000	0.00000000
103	395.59170000	3.85960000	0.00000000
104	400.14610000	0.00100000	0.00000000
105	400.32330000	5.65880000	0.00000000
106	408.56230000	0.00050000	0.00000000
107	416.84490000	10.67010000	0.00000000
108	427.45000000	0.00250000	0.00000000
109	433.75120000	6.68240000	0.00000000
110	437.42030000	0.00170000	0.00000000
111	439.96600000	0.00210000	0.00000000
112	447.53280000	0.09610000	0.00000000
113	447.88770000	2.00280000	0.00000000
114	450.72530000	23.25270000	0.00000000
115	463.64900000	0.00010000	0.00000000
116	472.38700000	17.95490000	0.00000000
117	490.34660000	0.00020000	0.00000000
118	497.93270000	56.88020000	0.00000000
119	518.04230000	11.75900000	0.00000000
120	519.33590000	0.03020000	0.00000000
121	529.80860000	6.86230000	0.00000000
122	532.11140000	0.03150000	0.00000000
123	554.06890000	15.13470000	0.00000000
124	555.18640000	0.20310000	0.00000000

125	585.51590000	1.64750000	0.00000000
126	598.44750000	7.50030000	0.00000000
127	599.38710000	0.05660000	0.00000000
128	601.59780000	0.00050000	0.00000000
129	618.46990000	9.83930000	0.00000000
130	618.76550000	0.01740000	0.00000000
131	620.41620000	5.32130000	0.00000000
132	620.62510000	0.28780000	0.00000000
133	669.75230000	49.01220000	0.00000000
134	669.97000000	0.02490000	0.00000000
135	679.65040000	55.63310000	0.00000000
136	680.39220000	0.05530000	0.00000000
137	687.63940000	64.90900000	0.00000000
138	691.50940000	0.00010000	0.00000000
139	732.99390000	3.42720000	0.00000000
140	733.94080000	32.48320000	0.00000000
141	734.62560000	17.70530000	0.00000000
142	734.68970000	2.38290000	0.00000000
143	739.83030000	8.31510000	0.00000000
144	739.84030000	0.51150000	0.00000000
145	744.83850000	12.41720000	0.00000000
146	745.70720000	2.14630000	0.00000000
147	765.15550000	22.60150000	0.00000000
148	765.34540000	81.03490000	0.00000000
149	778.52320000	54.44480000	0.00000000
150	778.75980000	7.13900000	0.00000000
151	780.10090000	4.15450000	0.00000000
152	780.12670000	0.04620000	0.00000000
153	784.36650000	1.03350000	0.00000000
154	784.65660000	0.00570000	0.00000000
155	797.83930000	0.06620000	0.00000000
156	799.52450000	9.30040000	0.00000000
157	809.79330000	9.09930000	0.00000000
158	810.75990000	0.07020000	0.00000000
159	829.99400000	10.03210000	0.00000000
160	831.33440000	0.00040000	0.00000000
161	834.34890000	8.28410000	0.00000000
162	836.14430000	0.00020000	0.00000000
163	853.48820000	4.46610000	0.00000000
164	853.75040000	0.00120000	0.00000000
165	864.37400000	1.62880000	0.00000000
166	864.54170000	0.97050000	0.00000000
167	865.42570000	0.07500000	0.00000000
168	865.49840000	0.01360000	0.00000000
169	868.17710000	13.46170000	0.00000000
170	868.90850000	0.00420000	0.00000000
171	870.93320000	11.12570000	0.00000000
172	873.30490000	0.00080000	0.00000000
173	873.83150000	7.16250000	0.00000000
174	878.10070000	0.00050000	0.00000000
175	890.75650000	10.07400000	0.00000000
176	891.28970000	0.41820000	0.00000000
177	900.30670000	11.77160000	0.00000000
178	900.40060000	0.00470000	0.00000000
179	924.61390000	2.95220000	0.00000000
180	924.62770000	0.19630000	0.00000000
181	934.30270000	4.27570000	0.00000000
182	934.33930000	11.54970000	0.00000000
183	957.69860000	1.44730000	0.00000000
184	958.01200000	1.31030000	0.00000000
185	960.49180000	1.90380000	0.00000000
186	960.85800000	1.17610000	0.00000000
187	983.80220000	0.37790000	0.00000000
188	984.06390000	0.32400000	0.00000000

189	986.34570000	0.44070000	0.00000000
190	986.54180000	0.32270000	0.00000000
191	991.64730000	1.43720000	0.00000000
192	991.66280000	0.49920000	0.00000000
193	1014.30910000	0.01940000	0.00000000
194	1014.46620000	130.89730000	0.00000000
195	1022.61800000	41.36270000	0.00000000
196	1022.69020000	0.00500000	0.00000000
197	1022.90390000	0.00700000	0.00000000
198	1022.98580000	80.17740000	0.00000000
199	1029.48620000	42.18160000	0.00000000
200	1029.52770000	0.06170000	0.00000000
201	1040.72390000	0.13730000	0.00000000
202	1040.97780000	0.12030000	0.00000000
203	1044.34880000	0.08110000	0.00000000
204	1044.36530000	20.45860000	0.00000000
205	1059.31740000	12.07150000	0.00000000
206	1059.44160000	4.96110000	0.00000000
207	1064.38260000	0.27190000	0.00000000
208	1064.39060000	0.23120000	0.00000000
209	1066.38020000	6.27220000	0.00000000
210	1066.44480000	46.05850000	0.00000000
211	1069.39360000	22.46290000	0.00000000
212	1069.49570000	7.23610000	0.00000000
213	1079.45980000	12.09180000	0.00000000
214	1079.47440000	1.23570000	0.00000000
215	1092.87830000	13.04930000	0.00000000
216	1092.94080000	11.16560000	0.00000000
217	1106.98860000	306.26070000	0.00000000
218	1107.98620000	0.01080000	0.00000000
219	1115.15470000	77.48230000	0.00000000
220	1115.37290000	0.16450000	0.00000000
221	1122.20730000	109.72820000	0.00000000
222	1122.77100000	0.00440000	0.00000000
223	1127.63230000	0.34290000	0.00000000
224	1127.64600000	0.13870000	0.00000000
225	1142.54570000	5.49200000	0.00000000
226	1142.56260000	0.00020000	0.00000000
227	1149.23740000	5.42980000	0.00000000
228	1149.27060000	0.02940000	0.00000000
229	1155.51720000	13.20290000	0.00000000
230	1155.60860000	0.36410000	0.00000000
231	1166.92310000	0.45350000	0.00000000
232	1166.95260000	13.09930000	0.00000000
233	1171.42080000	23.14790000	0.00000000
234	1171.99200000	0.02320000	0.00000000
235	1175.74060000	1.48440000	0.00000000
236	1175.74280000	1.91540000	0.00000000
237	1180.32350000	16.03670000	0.00000000
238	1180.65640000	0.01620000	0.00000000
239	1186.03510000	1.92330000	0.00000000
240	1186.07200000	8.29540000	0.00000000
241	1186.76270000	1.17400000	0.00000000
242	1186.83720000	0.21720000	0.00000000
243	1238.54150000	2.43980000	0.00000000
244	1238.55290000	0.24110000	0.00000000
245	1249.47980000	1.06380000	0.00000000
246	1249.59640000	22.95410000	0.00000000
247	1250.61750000	230.86230000	0.00000000
248	1250.91360000	14.84060000	0.00000000
249	1260.45010000	3.19880000	0.00000000
250	1260.48070000	0.01730000	0.00000000
251	1275.99750000	0.41600000	0.00000000
252	1276.17890000	39.27680000	0.00000000

253	1276.42330000	0.47290000	0.00000000
254	1276.47320000	3.99130000	0.00000000
255	1276.71630000	413.60180000	0.00000000
256	1277.06740000	4.18910000	0.00000000
257	1282.74430000	6.46170000	0.00000000
258	1282.87770000	17.36130000	0.00000000
259	1301.68440000	0.47350000	0.00000000
260	1301.74240000	0.16770000	0.00000000
261	1306.16120000	1.69730000	0.00000000
262	1306.30140000	48.60110000	0.00000000
263	1313.00550000	2.76790000	0.00000000
264	1313.05730000	9.89860000	0.00000000
265	1320.56690000	6.92320000	0.00000000
266	1320.60360000	1.12750000	0.00000000
267	1325.03170000	4.81020000	0.00000000
268	1325.09570000	0.46090000	0.00000000
269	1327.37220000	0.45630000	0.00000000
270	1327.39720000	12.53360000	0.00000000
271	1331.34620000	13.20330000	0.00000000
272	1331.48220000	35.82570000	0.00000000
273	1336.40800000	0.39630000	0.00000000
274	1336.41350000	0.19180000	0.00000000
275	1350.94470000	1.13290000	0.00000000
276	1350.96300000	0.48900000	0.00000000
277	1354.87930000	3.43170000	0.00000000
278	1354.89810000	0.55780000	0.00000000
279	1388.55890000	0.22970000	0.00000000
280	1393.59970000	31.34780000	0.00000000
281	1393.90660000	0.02250000	0.00000000
282	1397.83590000	274.94330000	0.00000000
283	1398.17110000	264.18310000	0.00000000
284	1400.98700000	0.04000000	0.00000000
285	1401.18110000	23.13480000	0.00000000
286	1403.14130000	0.02220000	0.00000000
287	1403.39090000	18.80930000	0.00000000
288	1404.00700000	0.22110000	0.00000000
289	1412.26600000	19.63510000	0.00000000
290	1412.40150000	0.00160000	0.00000000
291	1414.42950000	3.69090000	0.00000000
292	1414.44410000	7.09640000	0.00000000
293	1416.04340000	225.09400000	0.00000000
294	1417.80330000	0.08160000	0.00000000
295	1425.01420000	8.83300000	0.00000000
296	1425.05110000	22.40090000	0.00000000
297	1428.17960000	51.90480000	0.00000000
298	1428.22250000	5.22650000	0.00000000
299	1438.26820000	28.66800000	0.00000000
300	1438.32110000	0.14140000	0.00000000
301	1445.74270000	30.17970000	0.00000000
302	1445.79040000	1.40390000	0.00000000
303	1470.39460000	29.56760000	0.00000000
304	1470.45670000	0.05760000	0.00000000
305	1473.98000000	19.97940000	0.00000000
306	1475.57100000	68.69050000	0.00000000
307	1475.79420000	0.38000000	0.00000000
308	1476.93270000	0.09660000	0.00000000
309	1477.66760000	6.46800000	0.00000000
310	1477.68650000	0.91950000	0.00000000
311	1481.87330000	7.11990000	0.00000000
312	1481.88150000	0.05550000	0.00000000
313	1489.30770000	3.10100000	0.00000000
314	1489.35320000	0.00050000	0.00000000
315	1489.41740000	1.88160000	0.00000000
316	1489.62730000	0.01590000	0.00000000

317	1493.83720000	11.47630000	0.00000000
318	1493.98780000	0.07000000	0.00000000
319	1498.17620000	13.51410000	0.00000000
320	1498.33170000	0.17880000	0.00000000
321	1499.76340000	18.79760000	0.00000000
322	1499.78480000	0.20860000	0.00000000
323	1500.32260000	199.54180000	0.00000000
324	1501.39890000	0.62240000	0.00000000
325	1501.45220000	35.89350000	0.00000000
326	1503.04800000	0.04990000	0.00000000
327	1503.33590000	18.63280000	0.00000000
328	1503.76430000	0.04340000	0.00000000
329	1504.49110000	82.83200000	0.00000000
330	1505.45140000	0.00090000	0.00000000
331	1505.67990000	22.58460000	0.00000000
332	1509.00630000	7.43400000	0.00000000
333	1509.22950000	73.26810000	0.00000000
334	1512.37020000	9.47520000	0.00000000
335	1512.51340000	130.48000000	0.00000000
336	1513.40780000	56.23240000	0.00000000
337	1513.51430000	3.17910000	0.00000000
338	1515.12040000	25.86140000	0.00000000
339	1515.26570000	0.01110000	0.00000000
340	1519.41670000	46.29990000	0.00000000
341	1521.01300000	0.05720000	0.00000000
342	1523.83790000	28.77860000	0.00000000
343	1530.61490000	0.26600000	0.00000000
344	1531.01980000	43.29050000	0.00000000
345	1542.56340000	0.00630000	0.00000000
346	1542.87610000	30.17110000	0.00000000
347	1554.70380000	0.02510000	0.00000000
348	1560.98490000	0.03300000	0.00000000
349	1613.19630000	20.66570000	0.00000000
350	1613.69090000	0.40100000	0.00000000
351	1623.29400000	12.33390000	0.00000000
352	1623.45300000	8.64440000	0.00000000
353	1633.93170000	86.59860000	0.00000000
354	1634.44430000	0.20960000	0.00000000
355	1636.54750000	31.94440000	0.00000000
356	1637.04180000	0.18570000	0.00000000
357	1711.00330000	1383.54670000	0.00000000
358	1714.09180000	0.03700000	0.00000000
359	2988.96220000	37.56870000	0.00000000
360	2989.04930000	16.56450000	0.00000000
361	2992.47220000	28.42450000	0.00000000
362	2992.49350000	0.10990000	0.00000000
363	2995.09700000	98.23830000	0.00000000
364	2995.12410000	6.25580000	0.00000000
365	2997.04010000	45.83690000	0.00000000
366	2997.05150000	0.32760000	0.00000000
367	3008.18430000	15.21070000	0.00000000
368	3008.20500000	41.75560000	0.00000000
369	3009.16500000	59.34510000	0.00000000
370	3009.22440000	28.05760000	0.00000000
371	3011.90850000	71.16110000	0.00000000
372	3011.92610000	3.17410000	0.00000000
373	3013.56870000	7.74990000	0.00000000
374	3013.58050000	40.26050000	0.00000000
375	3017.08840000	72.55920000	0.00000000
376	3017.09610000	2.73630000	0.00000000
377	3018.15170000	45.84720000	0.00000000
378	3018.18640000	41.21020000	0.00000000
379	3018.29050000	1.39190000	0.00000000
380	3018.29560000	19.75520000	0.00000000

381	3019.64180000	151.24880000	0.00000000
382	3019.74620000	0.14380000	0.00000000
383	3024.46060000	0.00580000	0.00000000
384	3024.48370000	53.45740000	0.00000000
385	3030.84220000	18.92780000	0.00000000
386	3030.88840000	40.81430000	0.00000000
387	3039.48460000	16.14010000	0.00000000
388	3039.50530000	18.26090000	0.00000000
389	3040.75760000	40.14830000	0.00000000
390	3040.81810000	9.34480000	0.00000000
391	3042.49450000	12.25020000	0.00000000
392	3042.51170000	33.25310000	0.00000000
393	3059.52660000	13.20290000	0.00000000
394	3059.54590000	53.42680000	0.00000000
395	3060.15670000	1.60270000	0.00000000
396	3060.18140000	84.64240000	0.00000000
397	3065.30630000	15.86610000	0.00000000
398	3065.38130000	11.99050000	0.00000000
399	3068.53730000	7.17320000	0.00000000
400	3068.55440000	35.01730000	0.00000000
401	3072.78300000	1.70000000	0.00000000
402	3072.81350000	109.00190000	0.00000000
403	3078.30680000	118.84420000	0.00000000
404	3078.31770000	1.42070000	0.00000000
405	3080.75570000	12.02380000	0.00000000
406	3080.83970000	12.41470000	0.00000000
407	3081.49450000	20.77900000	0.00000000
408	3081.51390000	63.95210000	0.00000000
409	3085.93200000	60.29640000	0.00000000
410	3086.05840000	50.45190000	0.00000000
411	3088.87330000	96.77730000	0.00000000
412	3088.91250000	5.10670000	0.00000000
413	3120.04560000	12.43210000	0.00000000
414	3120.06020000	11.32770000	0.00000000
415	3157.25720000	0.15580000	0.00000000
416	3157.25870000	0.24920000	0.00000000
417	3163.03160000	2.76590000	0.00000000
418	3163.03860000	0.92040000	0.00000000
419	3163.92450000	10.07700000	0.00000000
420	3163.93470000	3.79520000	0.00000000
421	3177.06690000	16.94780000	0.00000000
422	3177.13170000	16.54580000	0.00000000
423	3179.54270000	13.31020000	0.00000000
424	3179.72200000	12.59700000	0.00000000
425	3187.43770000	27.45210000	0.00000000
426	3187.51500000	13.08380000	0.00000000
427	3192.13570000	15.98590000	0.00000000
428	3192.31700000	12.41230000	0.00000000
429	3198.96710000	10.89590000	0.00000000
430	3199.39670000	9.06670000	0.00000000
431	3206.07330000	19.03980000	0.00000000
432	3206.12250000	5.75660000	0.00000000

S6.10. Calculations on $Cu_2(\mathbf{2}^*)_4(MeCN)_2$

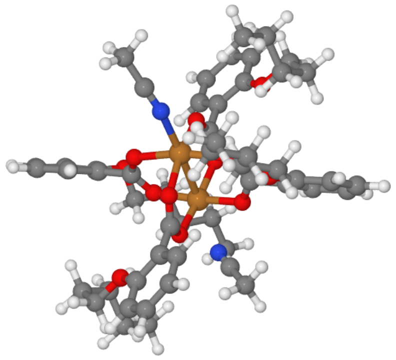

Route	: # opt freq b3lyp/genecp geom=connectivity int=ultrafine	
SMILES	: CCCCCOc1ccccc1[C]2O[Cu]345O[C](O[Cu]3(O2)(O[C](O4)c6ccccc6OCCCCC)(O[C](O5)c7ccccc7OCCCCC)[N]#CC)c8ccccc8OCCCCC.CC#N	
Formula	: $C_{52}H_{66}Cu_2N_2O_{12}$	
Charge	: 0	
Multiplicity	: 1	
Dipole	: 17.5263	Debye
Energy	: -3429.13945740	a.u.
Gibbs Energy	: -3428.18636100	a.u.
Number of imaginary frequencies	: 0	

S6.10.1. Cartesian Co-ordinates (XYZ format)

134

O	-0.86562502	1.91687202	0.80141598
O	0.78531200	1.94604897	-0.74019903
O	0.89057797	0.20711800	2.31273007
O	2.34386110	0.02087200	0.59623498
C	-0.09444400	2.48799610	-0.01003100
C	2.04380703	0.12838100	1.82235205
O	1.99735999	4.38096094	0.19813301
O	4.44469786	-1.52993798	1.62817204
C	0.77607203	4.88837099	-0.09946700
C	4.35411692	-0.59297001	2.62294412
C	-1.58431995	4.44062185	-0.41381201
H	-2.39205098	3.71922493	-0.44320199
C	-0.29358000	3.97340703	-0.17488800
C	3.10610509	1.05121899	3.88703704
H	2.19747901	1.62654805	4.01326799
C	3.20015502	0.19431899	2.78485608
C	3.11092210	5.26750708	0.34885901
H	2.89662504	5.99386501	1.14253104
H	3.27056789	5.82063293	-0.58434403

C	5.17330122	-1.12740004	0.44439200
H	6.21869183	-0.93281001	0.71940702
H	4.72884703	-0.20985700	0.05266600
C	5.07527113	-2.24792194	-0.57485902
H	5.48554087	-3.16518402	-0.13598700
H	4.01725483	-2.43079495	-0.78294700
C	4.32981300	4.43232012	0.71391201
H	4.12037992	3.91959596	1.65784705
H	5.15486002	5.12909794	0.90960002
C	5.80543709	-1.91301799	-1.88019001
H	5.37148905	-1.00279903	-2.30881095
H	6.86114788	-1.69262397	-1.67055905
C	4.75505209	3.40184689	-0.34227800
H	5.56564617	2.79864693	0.08294800
H	3.92445588	2.71436000	-0.52618402
C	6.43711615	-2.70023799	-4.22947884
H	6.00297117	-1.80889797	-4.69252682
H	6.36076784	-3.52007508	-4.94951677
H	7.50123787	-2.50221109	-4.06363392
C	5.76990700	2.94654989	-2.63986397
H	6.63529110	2.42781901	-2.21554208
H	6.08442593	3.39544201	-3.58657694
H	5.00988388	2.19179893	-2.85929203
C	-1.83190203	5.79538107	-0.62328899
C	0.52108300	6.25128508	-0.29338101
H	1.32594597	6.97051001	-0.23271000
C	-0.77321398	6.69390106	-0.56191498
C	4.15321779	1.16775799	4.79349804
C	5.39221001	-0.48787099	3.55256104
H	6.25928402	-1.12578499	3.42625308
C	5.29974508	0.39252499	4.62472916
O	-0.39548901	-2.05736303	1.17817295
O	-2.12800598	-0.37935099	-0.33571699
O	0.95649201	-2.01554489	-0.63313699
O	-0.61266202	-0.10843200	-1.98679101
C	0.34829900	-2.58696508	0.31920999
C	-1.78784597	-0.30228701	-1.53718603
O	-1.60564303	-4.49228621	-0.20944400
O	-4.38360691	0.85264701	-1.41751897
C	-0.41573700	-5.00124311	0.20978300
C	-4.13699293	0.05453900	-2.48457694
C	1.87091601	-4.50308180	0.83700502
H	2.64968705	-3.76931405	1.01087606
C	0.60753000	-4.06876183	0.45043200
C	-2.52081490	-1.26813996	-3.71360803
H	-1.51629400	-1.66588700	-3.78611898
C	-2.84126306	-0.49537301	-2.59648395
C	-2.70003295	-5.37917185	-0.44843599
H	-2.42651510	-6.10399294	-1.22529995
H	-2.92799711	-5.93603277	0.46862200
C	-5.70244217	1.35877395	-1.20484602
H	-5.98933697	2.01826000	-2.03471589
H	-6.41695786	0.52670300	-1.16793394
C	-5.69770384	2.11945295	0.11162300
H	-4.95591307	2.92241907	0.05142400
H	-5.35950804	1.44209301	0.90157503
C	-3.89204907	-4.54944086	-0.90546602
H	-3.63332510	-4.07007599	-1.85463297
H	-4.71049786	-5.24923515	-1.11829102
C	-7.07309484	2.69989610	0.45801601
H	-7.81199694	1.88961303	0.50981998
H	-7.40709591	3.36533093	-0.34886801
C	-4.36227703	-3.47636604	0.08747100
H	-5.18051195	-2.91859603	-0.38367501

H	-3.55492902	-2.75669909	0.24728100
C	-8.45901394	4.05266714	2.12973404
H	-9.21065426	3.26228309	2.22132802
H	-8.43367004	4.59754992	3.07725906
H	-8.80325031	4.74734116	1.35713398
C	-5.41305017	-2.91856098	2.34203911
H	-6.28506804	-2.44191790	1.88272798
H	-5.72620106	-3.31821489	3.31156111
H	-4.66643715	-2.13827705	2.51688004
C	2.12704492	-5.86223698	1.01934004
C	-0.15334800	-6.36329794	0.38170600
H	-0.92506897	-7.09786415	0.19668300
C	1.11303902	-6.78371382	0.79022902
C	-3.46477699	-1.54212797	-4.69964600
C	-5.07931185	-0.21221501	-3.48551297
H	-6.07425785	0.20580199	-3.42128205
C	-4.74353123	-1.01063800	-4.57694292
H	6.11611509	0.46286699	5.33512020
H	1.29941297	-7.84391689	0.92179298
H	-5.49211979	-1.20727098	-5.33659220
H	-0.94673401	7.75341988	-0.71487802
H	-2.83882499	6.14133310	-0.82388800
H	-3.20132303	-2.15889311	-5.55037212
H	3.11078906	-6.19030905	1.33282495
H	4.06972599	1.84717703	5.63387823
Cu	0.93246800	-0.04286800	-0.75753897
Cu	-0.81230301	-0.06793200	1.19171906
N	-2.12380505	-0.15245900	3.00293589
N	2.66413307	0.25183499	-2.98465109
C	1.92339504	0.97524399	-3.49311495
C	0.96233302	1.88216102	-4.09780121
H	1.24108696	2.10392404	-5.12965918
H	-0.02364400	1.41545498	-4.06989193
H	0.92646599	2.80546188	-3.51756692
C	-2.43577194	-0.37054899	4.08722496
C	-2.81984496	-0.65584803	5.45955515
H	-2.00543308	-1.17565703	5.96800089
H	-3.03441691	0.27343100	5.99059486
H	-3.70892692	-1.28905797	5.47476101
C	5.23311520	4.00392389	-1.66992497
H	6.01437712	4.74985504	-1.47275102
H	4.40981579	4.54372311	-2.15200210
C	5.72423887	-3.03894997	-2.91739297
H	4.67021513	-3.25874996	-3.12041712
H	6.15301085	-3.95545006	-2.49329591
C	-4.84452391	-4.01767588	1.43882203
H	-4.01456881	-4.50882483	1.95839906
H	-5.60886002	-4.78930092	1.27759397
C	-7.08513880	3.47351098	1.78170204
H	-6.34773922	4.28319216	1.73182702
H	-6.75190210	2.80954599	2.58803010

S6.10.2. Frequencies

Mode	IR frequency	IR intensity	Raman intensity
1	7.36010000	0.04550000	0.00000000
2	8.92640000	0.48290000	0.00000000
3	9.66890000	0.76530000	0.00000000
4	10.96910000	2.73750000	0.00000000
5	11.55170000	0.64160000	0.00000000
6	14.34240000	0.18750000	0.00000000
7	15.27180000	0.68970000	0.00000000
8	16.43290000	2.30490000	0.00000000
9	17.61070000	0.53370000	0.00000000
10	17.96730000	0.68580000	0.00000000
11	18.73920000	0.12670000	0.00000000
12	22.57930000	1.45990000	0.00000000
13	24.50420000	3.59850000	0.00000000
14	25.94410000	0.09440000	0.00000000
15	26.86840000	0.22860000	0.00000000
16	27.70320000	0.52020000	0.00000000
17	30.63590000	0.64920000	0.00000000
18	32.49320000	2.76360000	0.00000000
19	35.28160000	4.25500000	0.00000000
20	35.43550000	0.19950000	0.00000000
21	37.33980000	0.68170000	0.00000000
22	39.89470000	2.47550000	0.00000000
23	44.16250000	2.54750000	0.00000000
24	46.16570000	6.11070000	0.00000000
25	47.34690000	1.91960000	0.00000000
26	47.71170000	3.54160000	0.00000000
27	49.40670000	1.38720000	0.00000000
28	56.95900000	0.61440000	0.00000000
29	63.07980000	0.43700000	0.00000000
30	66.65940000	1.00350000	0.00000000
31	69.75330000	0.75810000	0.00000000
32	70.46670000	0.81330000	0.00000000
33	73.53450000	0.91870000	0.00000000
34	77.45350000	1.44630000	0.00000000
35	79.16000000	2.50780000	0.00000000
36	81.37770000	2.95140000	0.00000000
37	82.26690000	3.13330000	0.00000000
38	84.80740000	0.38890000	0.00000000
39	85.87290000	0.50370000	0.00000000
40	86.69170000	0.53940000	0.00000000
41	91.90490000	0.66110000	0.00000000
42	97.28840000	3.02250000	0.00000000
43	102.48000000	0.71940000	0.00000000
44	105.28350000	0.48730000	0.00000000
45	111.15260000	3.03600000	0.00000000
46	115.38840000	0.04550000	0.00000000
47	115.97820000	1.74540000	0.00000000
48	123.84970000	0.10860000	0.00000000
49	129.04200000	4.08860000	0.00000000
50	131.10490000	0.57460000	0.00000000
51	132.47310000	0.35120000	0.00000000
52	139.79220000	1.73650000	0.00000000
53	141.77910000	1.62860000	0.00000000
54	147.96240000	2.97670000	0.00000000
55	150.14610000	0.52980000	0.00000000
56	154.43690000	1.44030000	0.00000000
57	164.43830000	2.49820000	0.00000000
58	165.46870000	1.81750000	0.00000000
59	179.00870000	2.54890000	0.00000000
60	185.18970000	1.97900000	0.00000000

61	189.15400000	1.51080000	0.00000000
62	191.48480000	2.91380000	0.00000000
63	195.50880000	2.57930000	0.00000000
64	198.86010000	0.45760000	0.00000000
65	201.96950000	3.07840000	0.00000000
66	204.86870000	4.39320000	0.00000000
67	207.54210000	2.70900000	0.00000000
68	210.39770000	1.40120000	0.00000000
69	215.20940000	1.13260000	0.00000000
70	221.61650000	0.91680000	0.00000000
71	225.27800000	0.05680000	0.00000000
72	227.17800000	2.60590000	0.00000000
73	235.64830000	11.73850000	0.00000000
74	244.18390000	0.07680000	0.00000000
75	244.30940000	0.07060000	0.00000000
76	249.15650000	3.07990000	0.00000000
77	252.76890000	0.45670000	0.00000000
78	255.42340000	2.58730000	0.00000000
79	260.32940000	2.34020000	0.00000000
80	269.09160000	3.02490000	0.00000000
81	281.81910000	7.93130000	0.00000000
82	286.87260000	3.17310000	0.00000000
83	292.38690000	1.03670000	0.00000000
84	294.38820000	7.33990000	0.00000000
85	295.51560000	2.13150000	0.00000000
86	301.14410000	0.91700000	0.00000000
87	306.45180000	2.80570000	0.00000000
88	311.58700000	4.92210000	0.00000000
89	367.29840000	4.18780000	0.00000000
90	383.02490000	0.60780000	0.00000000
91	385.60980000	0.96150000	0.00000000
92	390.48370000	0.51390000	0.00000000
93	390.73580000	1.02980000	0.00000000
94	391.20540000	1.24730000	0.00000000
95	392.01550000	0.62290000	0.00000000
96	394.32810000	1.65690000	0.00000000
97	395.98730000	1.44360000	0.00000000
98	410.65690000	4.13320000	0.00000000
99	428.77760000	3.28760000	0.00000000
100	430.34820000	5.40960000	0.00000000
101	432.42830000	1.17990000	0.00000000
102	442.91810000	0.07030000	0.00000000
103	446.52360000	1.59950000	0.00000000
104	449.94810000	0.60510000	0.00000000
105	455.26380000	17.60710000	0.00000000
106	463.76530000	0.20250000	0.00000000
107	476.38740000	18.49750000	0.00000000
108	479.96610000	7.71100000	0.00000000
109	497.02890000	3.65360000	0.00000000
110	505.31080000	36.72100000	0.00000000
111	514.85890000	7.34520000	0.00000000
112	517.83430000	5.89070000	0.00000000
113	527.06300000	7.75930000	0.00000000
114	532.26970000	1.02730000	0.00000000
115	540.73840000	5.87130000	0.00000000
116	556.57480000	8.21620000	0.00000000
117	585.38640000	0.14780000	0.00000000
118	597.29880000	5.72980000	0.00000000
119	599.48850000	2.24690000	0.00000000
120	601.13980000	0.64800000	0.00000000
121	617.70980000	6.06800000	0.00000000
122	618.53900000	2.35940000	0.00000000
123	619.63770000	1.70000000	0.00000000
124	622.25630000	5.38340000	0.00000000

125	680.59370000	25.19010000	0.00000000
126	687.26060000	32.46110000	0.00000000
127	688.18260000	60.76740000	0.00000000
128	693.20990000	6.33510000	0.00000000
129	726.89910000	7.42720000	0.00000000
130	730.57670000	22.38530000	0.00000000
131	732.35480000	7.56340000	0.00000000
132	735.76690000	6.89110000	0.00000000
133	738.94750000	2.92760000	0.00000000
134	740.02860000	7.13260000	0.00000000
135	742.35720000	6.68480000	0.00000000
136	751.57650000	8.99620000	0.00000000
137	763.61360000	37.93020000	0.00000000
138	764.61200000	57.58760000	0.00000000
139	766.25820000	63.48420000	0.00000000
140	779.85130000	28.32310000	0.00000000
141	780.76780000	6.34780000	0.00000000
142	781.55120000	2.69660000	0.00000000
143	782.26810000	1.77940000	0.00000000
144	782.58690000	1.06500000	0.00000000
145	793.47690000	3.42500000	0.00000000
146	800.40360000	7.11930000	0.00000000
147	805.24000000	5.16100000	0.00000000
148	810.18560000	3.43870000	0.00000000
149	827.15640000	5.96340000	0.00000000
150	830.75120000	4.33810000	0.00000000
151	834.10760000	7.40850000	0.00000000
152	836.23040000	0.39170000	0.00000000
153	852.49490000	3.07510000	0.00000000
154	853.37120000	1.65310000	0.00000000
155	862.53940000	1.76020000	0.00000000
156	862.74810000	1.26970000	0.00000000
157	863.98250000	2.22870000	0.00000000
158	866.35300000	0.16130000	0.00000000
159	867.66490000	13.41340000	0.00000000
160	868.50960000	3.48680000	0.00000000
161	870.48520000	9.50280000	0.00000000
162	876.56720000	0.70390000	0.00000000
163	877.84890000	1.67910000	0.00000000
164	892.69480000	5.34200000	0.00000000
165	901.15360000	7.20630000	0.00000000
166	902.30600000	5.29650000	0.00000000
167	923.54790000	2.04240000	0.00000000
168	924.27470000	2.29560000	0.00000000
169	930.92310000	1.54750000	0.00000000
170	932.79900000	6.56010000	0.00000000
171	933.18540000	7.09740000	0.00000000
172	940.85500000	2.89090000	0.00000000
173	954.01140000	1.46020000	0.00000000
174	957.59730000	1.06520000	0.00000000
175	960.57490000	2.39190000	0.00000000
176	964.36030000	2.10990000	0.00000000
177	979.23130000	0.31940000	0.00000000
178	983.68060000	0.66970000	0.00000000
179	985.85670000	0.09430000	0.00000000
180	988.44480000	0.62910000	0.00000000
181	992.46600000	0.59870000	0.00000000
182	994.36290000	0.77540000	0.00000000
183	1007.35110000	92.87240000	0.00000000
184	1019.60020000	5.13070000	0.00000000
185	1021.43690000	39.34650000	0.00000000
186	1021.99470000	8.46720000	0.00000000
187	1022.91750000	29.89680000	0.00000000
188	1029.04460000	19.79230000	0.00000000

189	1030.25610000	20.50390000	0.00000000
190	1042.28760000	52.60120000	0.00000000
191	1043.59900000	2.65810000	0.00000000
192	1043.63040000	16.03160000	0.00000000
193	1059.20400000	9.62340000	0.00000000
194	1060.31450000	3.02230000	0.00000000
195	1063.29960000	2.72860000	0.00000000
196	1063.49160000	1.58620000	0.00000000
197	1063.89540000	4.59810000	0.00000000
198	1064.36960000	1.10790000	0.00000000
199	1065.76560000	13.44080000	0.00000000
200	1066.07600000	16.29210000	0.00000000
201	1066.22110000	29.56490000	0.00000000
202	1069.14330000	16.01320000	0.00000000
203	1070.84620000	17.00740000	0.00000000
204	1072.59210000	11.19890000	0.00000000
205	1092.62890000	11.91720000	0.00000000
206	1094.55470000	11.13390000	0.00000000
207	1115.10730000	33.30170000	0.00000000
208	1121.17080000	79.86890000	0.00000000
209	1121.46960000	28.80510000	0.00000000
210	1122.54820000	39.89430000	0.00000000
211	1142.64730000	3.16960000	0.00000000
212	1143.16640000	12.22270000	0.00000000
213	1148.32620000	3.10160000	0.00000000
214	1148.78330000	1.25290000	0.00000000
215	1152.72880000	6.13500000	0.00000000
216	1154.10390000	7.29310000	0.00000000
217	1166.51210000	7.67290000	0.00000000
218	1170.97090000	15.65010000	0.00000000
219	1171.57200000	13.87020000	0.00000000
220	1173.24920000	8.41430000	0.00000000
221	1180.73410000	9.14980000	0.00000000
222	1186.24730000	2.13010000	0.00000000
223	1186.83410000	15.39500000	0.00000000
224	1186.92450000	1.54010000	0.00000000
225	1187.16840000	4.32550000	0.00000000
226	1188.03460000	4.05750000	0.00000000
227	1239.02250000	0.89010000	0.00000000
228	1239.82450000	1.73020000	0.00000000
229	1247.40600000	1.89990000	0.00000000
230	1249.98910000	0.43140000	0.00000000
231	1251.32260000	132.52460000	0.00000000
232	1259.73410000	2.00090000	0.00000000
233	1260.76880000	0.98270000	0.00000000
234	1273.61790000	98.11780000	0.00000000
235	1275.07860000	213.69720000	0.00000000
236	1277.34730000	142.44020000	0.00000000
237	1277.81980000	46.29260000	0.00000000
238	1280.48120000	118.72160000	0.00000000
239	1281.97430000	12.57160000	0.00000000
240	1301.68550000	9.56860000	0.00000000
241	1302.99520000	0.67630000	0.00000000
242	1303.40400000	38.57900000	0.00000000
243	1305.67150000	71.34410000	0.00000000
244	1307.42600000	12.44780000	0.00000000
245	1313.87160000	7.64390000	0.00000000
246	1314.86370000	7.68130000	0.00000000
247	1320.31430000	5.16480000	0.00000000
248	1326.04620000	17.98550000	0.00000000
249	1326.56050000	30.81950000	0.00000000
250	1327.99410000	10.76140000	0.00000000
251	1328.06330000	0.64080000	0.00000000
252	1328.40980000	9.50330000	0.00000000

253	1330.36410000	15.02600000	0.00000000
254	1332.38620000	26.52080000	0.00000000
255	1337.03010000	0.43540000	0.00000000
256	1339.65990000	0.53390000	0.00000000
257	1347.88050000	0.85060000	0.00000000
258	1351.43400000	0.94260000	0.00000000
259	1356.04620000	0.70150000	0.00000000
260	1356.63890000	1.86980000	0.00000000
261	1377.14990000	78.07350000	0.00000000
262	1388.50140000	216.29700000	0.00000000
263	1394.63590000	36.11510000	0.00000000
264	1395.03260000	50.26840000	0.00000000
265	1397.24500000	105.53760000	0.00000000
266	1397.64470000	102.66660000	0.00000000
267	1400.43720000	12.29360000	0.00000000
268	1401.29750000	6.33630000	0.00000000
269	1402.52240000	8.20380000	0.00000000
270	1403.58460000	4.08670000	0.00000000
271	1405.20640000	37.56880000	0.00000000
272	1409.37130000	3.41100000	0.00000000
273	1411.03480000	4.13030000	0.00000000
274	1411.56510000	2.24360000	0.00000000
275	1414.75440000	1.63910000	0.00000000
276	1416.00580000	5.53190000	0.00000000
277	1425.20460000	14.51610000	0.00000000
278	1425.61620000	16.24090000	0.00000000
279	1426.00360000	29.30320000	0.00000000
280	1429.66170000	32.86190000	0.00000000
281	1460.47590000	13.23280000	0.00000000
282	1470.95250000	9.94230000	0.00000000
283	1471.94570000	8.51350000	0.00000000
284	1473.41820000	29.98230000	0.00000000
285	1476.26860000	34.25470000	0.00000000
286	1476.55980000	50.66180000	0.00000000
287	1476.90900000	1.16180000	0.00000000
288	1477.62400000	9.23880000	0.00000000
289	1477.82940000	3.28940000	0.00000000
290	1478.95880000	22.28830000	0.00000000
291	1489.16080000	0.06520000	0.00000000
292	1489.35050000	2.04360000	0.00000000
293	1489.84460000	1.72250000	0.00000000
294	1490.81210000	0.89670000	0.00000000
295	1492.34320000	2.10700000	0.00000000
296	1493.97880000	6.08460000	0.00000000
297	1497.41360000	13.16790000	0.00000000
298	1499.32240000	15.68560000	0.00000000
299	1499.75900000	142.40170000	0.00000000
300	1500.86410000	15.67900000	0.00000000
301	1501.54500000	8.15670000	0.00000000
302	1501.61040000	6.18110000	0.00000000
303	1501.98080000	6.63600000	0.00000000
304	1502.34850000	7.82810000	0.00000000
305	1503.79660000	75.04510000	0.00000000
306	1507.47800000	48.58190000	0.00000000
307	1510.23600000	20.66550000	0.00000000
308	1510.79180000	114.66630000	0.00000000
309	1512.36880000	16.19680000	0.00000000
310	1512.61100000	25.77510000	0.00000000
311	1513.57000000	47.70950000	0.00000000
312	1514.33350000	11.62550000	0.00000000
313	1515.55180000	41.36640000	0.00000000
314	1520.07230000	11.14460000	0.00000000
315	1527.70430000	16.33720000	0.00000000
316	1529.58370000	26.53370000	0.00000000

317	1531.22720000	18.54150000	0.00000000
318	1531.70420000	27.66460000	0.00000000
319	1552.67760000	6.79730000	0.00000000
320	1560.75920000	5.29170000	0.00000000
321	1611.44750000	11.18070000	0.00000000
322	1618.66800000	14.31510000	0.00000000
323	1620.59260000	12.65830000	0.00000000
324	1624.80020000	8.35850000	0.00000000
325	1633.92970000	54.34400000	0.00000000
326	1635.46570000	36.49310000	0.00000000
327	1635.64270000	64.55010000	0.00000000
328	1636.40260000	3.21260000	0.00000000
329	2355.01830000	20.38970000	0.00000000
330	2390.46050000	21.93780000	0.00000000
331	2984.29810000	25.90970000	0.00000000
332	2988.17020000	3.19510000	0.00000000
333	2993.30540000	16.56690000	0.00000000
334	2993.93070000	76.44050000	0.00000000
335	2994.39550000	27.75780000	0.00000000
336	2994.63110000	1.00430000	0.00000000
337	2996.96560000	54.40910000	0.00000000
338	2997.76730000	36.69150000	0.00000000
339	3005.69530000	21.30530000	0.00000000
340	3009.65570000	53.16440000	0.00000000
341	3011.47310000	25.08300000	0.00000000
342	3011.97910000	54.12110000	0.00000000
343	3014.58890000	33.76990000	0.00000000
344	3015.34320000	3.34680000	0.00000000
345	3015.76770000	45.59020000	0.00000000
346	3016.48480000	20.68590000	0.00000000
347	3018.52850000	35.90160000	0.00000000
348	3018.98100000	20.70590000	0.00000000
349	3019.21760000	53.17830000	0.00000000
350	3020.56140000	18.44210000	0.00000000
351	3022.64450000	21.75030000	0.00000000
352	3029.17110000	29.19370000	0.00000000
353	3029.39140000	52.95680000	0.00000000
354	3034.39760000	17.10800000	0.00000000
355	3035.29360000	22.40220000	0.00000000
356	3036.02300000	37.03270000	0.00000000
357	3038.34890000	18.27450000	0.00000000
358	3042.30010000	15.45320000	0.00000000
359	3048.60010000	1.35740000	0.00000000
360	3050.10880000	15.96050000	0.00000000
361	3050.21480000	4.96010000	0.00000000
362	3062.75800000	25.17380000	0.00000000
363	3062.81740000	35.13920000	0.00000000
364	3071.41590000	6.00120000	0.00000000
365	3073.91940000	55.95960000	0.00000000
366	3074.98290000	28.29940000	0.00000000
367	3077.35180000	0.81740000	0.00000000
368	3077.87410000	48.68510000	0.00000000
369	3079.72930000	9.66960000	0.00000000
370	3080.09050000	53.74390000	0.00000000
371	3081.69450000	98.51400000	0.00000000
372	3082.35210000	53.99300000	0.00000000
373	3085.86140000	35.97960000	0.00000000
374	3086.32410000	73.84260000	0.00000000
375	3090.15000000	47.49360000	0.00000000
376	3092.07810000	53.14580000	0.00000000
377	3121.39160000	0.20710000	0.00000000
378	3121.86950000	0.28020000	0.00000000
379	3125.57270000	1.54020000	0.00000000
380	3129.41580000	0.65600000	0.00000000

381	3163.01080000	2.72740000	0.00000000
382	3164.39610000	7.34820000	0.00000000
383	3164.81280000	5.04070000	0.00000000
384	3166.00410000	5.69360000	0.00000000
385	3177.87050000	17.35360000	0.00000000
386	3178.79960000	9.74090000	0.00000000
387	3180.76580000	16.43630000	0.00000000
388	3184.27360000	18.11570000	0.00000000
389	3188.10870000	17.26140000	0.00000000
390	3191.58230000	17.09590000	0.00000000
391	3194.40520000	13.89140000	0.00000000
392	3198.90800000	10.14600000	0.00000000
393	3200.26730000	7.28520000	0.00000000
394	3206.06620000	12.83330000	0.00000000
395	3209.51230000	10.76750000	0.00000000
396	3209.99180000	10.09520000	0.00000000

S6.11. Calculations on $Cu_2(2^*)_4(H_2O)_2$

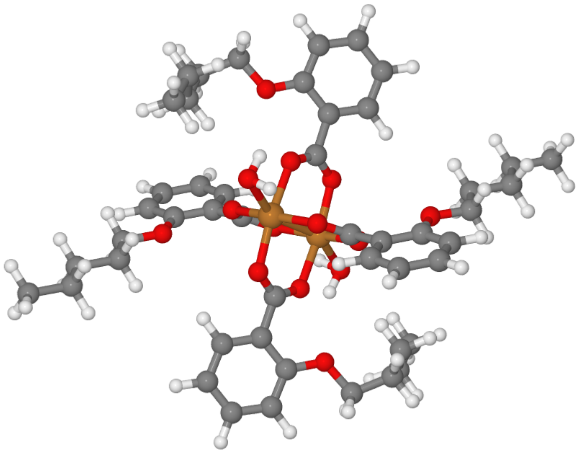

Route	: # opt freq b3lyp/genecp geom=connectivity int=ultrafine	
SMILES	: CCCCCOc1ccccc1[C]2O[Cu]345(O[C](O[Cu]3(O2)(O[C](O4)c6ccccc6OCCCCC)(O[C](O5)c7ccccc7OCCCCC)[OH2])c8ccccc8OCCCCC)[OH2]	
Formula	: $C_{48}H_{64}Cu_2O_{14}$	
Charge	: 0	
Multiplicity	: 1	
Dipole	: 0.0071	Debye
Energy	: -3316.47638885	a.u.
Gibbs Energy	: -3315.54872700	a.u.
Number of imaginary frequencies	: 1	

S6.11.1. Cartesian Co-ordinates (XYZ format)

```
128

O    1.26610804 -1.67506504  0.99311101
O   -0.15201400 -2.15825009 -0.68862301
O   -0.75136501 -0.08553700  2.15463090
O   -2.18456507 -0.56790501  0.48256901
C    0.71016198 -2.46645403  0.18123600
C   -1.88249195 -0.42932901  1.69611502
O   -1.02047503 -4.72219181 -0.10011800
O   -4.65153790  0.20492500  1.40647900
C    0.29904300 -4.99016619  0.04899000
C   -4.30472088 -0.46244100  2.53074098
C    2.52737188 -4.13307381  0.47519499
H    3.16765499 -3.27770996  0.64889199
C    1.17247605 -3.89545894  0.23849000
C   -2.52740097 -1.37302995  3.90454793
H   -1.47103798 -1.56764901  4.03838205
C   -2.93382096 -0.74676299  2.72468305
C   -1.95560896 -5.80553389 -0.16727500
H   -1.81798398 -6.46349096  0.69890898
H   -1.77179098 -6.39339113 -1.07444501
```

C	-6.03061914	0.45667601	1.12853396
H	-6.46528912	1.07864106	1.92190695
H	-6.58063698	-0.49245101	1.09734702
C	-6.10516882	1.16688597	-0.21348500
H	-5.53135204	2.09702802	-0.14625800
H	-5.60821295	0.54463500	-0.96386999
C	-3.36158490	-5.22172499	-0.15631901
H	-3.50161600	-4.69135284	0.79068601
H	-4.06035519	-6.06806183	-0.15092701
C	-7.54619884	1.46723294	-0.63964099
H	-8.11491585	0.53002900	-0.69674098
H	-8.03949547	2.07706308	0.12865400
C	-3.69587803	-4.27697277	-1.31942403
H	-4.70231819	-3.87671208	-1.14970303
H	-3.02148604	-3.41716504	-1.28390706
C	-9.07680321	2.48605704	-2.41826892
H	-9.65768814	1.56371498	-2.51731706
H	-9.10684204	3.00140405	-3.38196898
H	-9.58672523	3.12054491	-1.68643796
C	-4.10442114	-3.96726108	-3.82045603
H	-5.14020395	-3.64941692	-3.66337109
H	-4.04987478	-4.44481087	-4.80304384
H	-3.48256803	-3.06835294	-3.84467196
C	3.04583907	-5.42375278	0.48245499
C	0.82600802	-6.28809214	0.06488600
H	0.17696200	-7.13980579	-0.08167600
C	2.18747807	-6.49674702	0.27106300
C	-3.44566488	-1.75843406	4.87572479
C	-5.22419596	-0.84367502	3.51657391
H	-6.27638483	-0.63154900	3.38848996
C	-4.79480886	-1.49113202	4.67226601
O	0.15458900	2.16053510	0.68867701
O	2.18506598	0.56624800	-0.48058400
O	-1.26884401	1.67645597	-0.98831600
O	0.74983901	0.09066400	-2.15284801
C	-0.71030301	2.46828294	-0.17867100
C	1.88197398	0.43108499	-1.69425297
O	1.02108300	4.72406483	0.09829400
O	4.65050316	-0.20731500	-1.40705800
C	-0.29871199	4.99205303	-0.04816300
C	4.30388880	0.46222401	-2.53008294
C	-2.52801394	4.13495207	-0.46924600
H	-3.16872907	3.27955508	-0.64116699
C	-1.17261302	3.89731598	-0.23541801
C	2.52706504	1.37736499	-3.90150595
H	1.47089505	1.57361197	-4.03445101
C	2.93325400	0.74867499	-2.72283101
C	1.95638204	5.80733204	0.16412000
H	1.81769800	6.46523905	-0.70193702
H	1.77381396	6.39527798	1.07148504
C	6.02942610	-0.46131399	-1.13036704
H	6.46275520	-1.08269095	-1.92493200
H	6.58072615	0.48703200	-1.09813094
C	6.10390520	-1.17359698	0.21055500
H	5.52882195	-2.10288310	0.14231300
H	5.60827494	-0.55177498	0.96217400
C	3.36225390	5.22330284	0.15140399
H	3.50104403	4.69295597	-0.79579800
H	4.06118202	6.06950283	0.14521700
C	7.54482508	-1.47644699	0.63531399
H	8.11482716	-0.54007798	0.69328701
H	8.03677559	-2.08589196	-0.13414700
C	3.69772196	4.27840710	1.31406701
H	4.70377016	3.87773108	1.14301705

H	3.02289701	3.41889596	1.27943003
C	9.07531643	-2.49969697	2.41149592
H	9.65749359	-1.57825994	2.51137209
H	9.10534382	-3.01637411	3.37448311
H	9.58388138	-3.13387799	1.67845702
C	4.10915089	3.96852207	3.81463408
H	5.14446402	3.64983511	3.65616202
H	4.05634499	4.44625616	4.79722786
H	3.48662496	3.07010603	3.83987904
C	-3.04641008	5.42565680	-0.47587001
C	-0.82562500	6.29000902	-0.06342700
H	-0.17620499	7.14173412	0.08140800
C	-2.18751788	6.49867487	-0.26675999
C	3.44530797	1.76304495	-4.87258387
C	5.22333097	0.84376597	-3.51584101
H	6.27530193	0.63002700	-3.38867092
C	4.79419899	1.49359798	-4.67028522
H	-5.52740622	-1.77974105	5.41801405
H	-2.57036591	7.51333094	-0.26835999
H	5.52678394	1.78237498	-5.41598082
H	2.57038999	-7.51137686	0.27309901
H	4.10288191	-5.58676910	0.65422201
H	3.10975409	2.26416707	-5.77238083
H	-4.10381699	5.58867788	-0.64536798
H	-3.10993505	-2.25769591	5.77648878
Cu	-0.83405501	-0.26574299	-0.94636798
Cu	0.83373398	0.26748401	0.94846898
O	1.59025395	1.37431598	2.92592502
H	1.21101606	2.17455697	2.53420401
O	-1.58935905	-1.37219799	-2.92482495
H	-1.20912802	-2.17220402	-2.53358603
H	0.86247897	0.99910903	3.43847489
H	-0.86186999	-0.99550200	-3.43670011
C	-7.63740396	2.19033003	-1.98856294
H	-7.14279985	1.58321095	-2.75552201
H	-7.07194614	3.12807488	-1.93279898
C	-3.64939094	-4.92136717	-2.71083689
H	-4.27957392	-5.82041883	-2.71733093
H	-2.63058090	-5.26181412	-2.92992806
C	3.65334797	4.92285299	2.70552611
H	2.63502502	5.26395321	2.92586899
H	4.28409910	5.82151079	2.71120501
C	7.63601208	-2.20148110	1.98319495
H	7.06927776	-3.13840508	1.92656398
H	7.14274979	-1.59474099	2.75131893

S6.11.2. Frequencies

Mode	IR frequency	IR intensity	Raman intensity
1	-6.53940000	0.18600000	0.00000000
2	7.16910000	0.00020000	0.00000000
3	7.48450000	0.04090000	0.00000000
4	10.71520000	0.00000000	0.00000000
5	11.82320000	0.08810000	0.00000000
6	15.59300000	0.02030000	0.00000000
7	15.63040000	0.11040000	0.00000000
8	16.98700000	0.18090000	0.00000000
9	19.97970000	1.39910000	0.00000000
10	23.73800000	0.00080000	0.00000000
11	24.00710000	1.03700000	0.00000000
12	28.36770000	0.00010000	0.00000000
13	31.20370000	0.42350000	0.00000000
14	33.61800000	0.00000000	0.00000000
15	34.46780000	0.22830000	0.00000000
16	35.48260000	0.00000000	0.00000000
17	41.28610000	1.70320000	0.00000000
18	45.98070000	0.00050000	0.00000000
19	46.25860000	0.30600000	0.00000000
20	48.57960000	0.28830000	0.00000000
21	49.20710000	0.00010000	0.00000000
22	59.97060000	0.00000000	0.00000000
23	63.18360000	5.63810000	0.00000000
24	63.92540000	0.00100000	0.00000000
25	68.01800000	8.75050000	0.00000000
26	77.35670000	0.00060000	0.00000000
27	80.76900000	7.33720000	0.00000000
28	82.90070000	0.00230000	0.00000000
29	82.97200000	1.41410000	0.00000000
30	86.23440000	1.65570000	0.00000000
31	86.78500000	0.00020000	0.00000000
32	87.67750000	0.00010000	0.00000000
33	97.41820000	1.19310000	0.00000000
34	98.12030000	0.00120000	0.00000000
35	101.01050000	1.22800000	0.00000000
36	104.85510000	2.23250000	0.00000000
37	105.31470000	0.00040000	0.00000000
38	108.42980000	0.00000000	0.00000000
39	113.45880000	0.00000000	0.00000000
40	119.20640000	22.87540000	0.00000000
41	123.11990000	0.00000000	0.00000000
42	131.97600000	0.00000000	0.00000000
43	136.99710000	3.40810000	0.00000000
44	137.18650000	0.00020000	0.00000000
45	144.63050000	0.00010000	0.00000000
46	148.52940000	9.37180000	0.00000000
47	154.59050000	1.08020000	0.00000000
48	155.02180000	0.00030000	0.00000000
49	162.05410000	9.17590000	0.00000000
50	164.77290000	0.00020000	0.00000000
51	173.07360000	17.48600000	0.00000000
52	178.12770000	0.00010000	0.00000000
53	181.09280000	11.35580000	0.00000000
54	190.06880000	0.00000000	0.00000000
55	193.63010000	0.00080000	0.00000000
56	194.27610000	3.10880000	0.00000000
57	199.39530000	0.00000000	0.00000000
58	202.35890000	0.00010000	0.00000000
59	205.22150000	0.00010000	0.00000000
60	210.25410000	2.01760000	0.00000000

61	213.21820000	2.28920000	0.00000000
62	213.88660000	0.00080000	0.00000000
63	216.71130000	0.00000000	0.00000000
64	219.43880000	0.94640000	0.00000000
65	223.07430000	0.21810000	0.00000000
66	224.23250000	0.00010000	0.00000000
67	233.44050000	20.39950000	0.00000000
68	244.13290000	1.92750000	0.00000000
69	244.31060000	0.00020000	0.00000000
70	246.39840000	27.76410000	0.00000000
71	250.45480000	98.95250000	0.00000000
72	250.90370000	1.21930000	0.00000000
73	258.67670000	0.00010000	0.00000000
74	263.39210000	27.91050000	0.00000000
75	263.54530000	0.48100000	0.00000000
76	269.40430000	4.38460000	0.00000000
77	283.68560000	2.04720000	0.00000000
78	283.74800000	13.22350000	0.00000000
79	291.35890000	21.83220000	0.00000000
80	291.70190000	0.00350000	0.00000000
81	299.79400000	0.00590000	0.00000000
82	300.78220000	8.85900000	0.00000000
83	306.69510000	0.00010000	0.00000000
84	309.84170000	13.14150000	0.00000000
85	363.14760000	11.46450000	0.00000000
86	369.65060000	0.00020000	0.00000000
87	388.25150000	0.00000000	0.00000000
88	391.57900000	3.08870000	0.00000000
89	409.57090000	1.09620000	0.00000000
90	430.02110000	0.00000000	0.00000000
91	433.42170000	1.48670000	0.00000000
92	436.24850000	0.00000000	0.00000000
93	443.79470000	0.00720000	0.00000000
94	444.10840000	6.89440000	0.00000000
95	451.04940000	0.00000000	0.00000000
96	458.88660000	21.18280000	0.00000000
97	462.09150000	0.00040000	0.00000000
98	474.86680000	0.00060000	0.00000000
99	476.47150000	37.35830000	0.00000000
100	479.71920000	28.06790000	0.00000000
101	500.72810000	37.46640000	0.00000000
102	503.63220000	0.00180000	0.00000000
103	514.45370000	0.00090000	0.00000000
104	514.66370000	10.91950000	0.00000000
105	519.40470000	0.20430000	0.00000000
106	521.91370000	266.89720000	0.00000000
107	534.30310000	18.96180000	0.00000000
108	537.43400000	0.00010000	0.00000000
109	540.20130000	18.71820000	0.00000000
110	541.15180000	0.00070000	0.00000000
111	582.88850000	23.35100000	0.00000000
112	592.95540000	31.62940000	0.00000000
113	595.02860000	0.00010000	0.00000000
114	597.65370000	0.00010000	0.00000000
115	604.97790000	468.49000000	0.00000000
116	610.29670000	0.03330000	0.00000000
117	620.14240000	6.94320000	0.00000000
118	621.84200000	35.15150000	0.00000000
119	622.25640000	0.00350000	0.00000000
120	623.10710000	0.00070000	0.00000000
121	687.22760000	0.00010000	0.00000000
122	688.57970000	65.97950000	0.00000000
123	688.98870000	71.67480000	0.00000000
124	695.07620000	0.00000000	0.00000000

125	722.15530000	0.00210000	0.00000000
126	723.86680000	22.17420000	0.00000000
127	726.11480000	16.99220000	0.00000000
128	727.45360000	0.00240000	0.00000000
129	733.47960000	11.69380000	0.00000000
130	733.56310000	0.04630000	0.00000000
131	739.71670000	3.23770000	0.00000000
132	739.72510000	4.30100000	0.00000000
133	764.96270000	0.01670000	0.00000000
134	765.29470000	126.42600000	0.00000000
135	765.72090000	97.54030000	0.00000000
136	765.75390000	2.20360000	0.00000000
137	782.84000000	0.63770000	0.00000000
138	782.88300000	9.07740000	0.00000000
139	783.76520000	1.11170000	0.00000000
140	784.09980000	0.00020000	0.00000000
141	798.31540000	0.00000000	0.00000000
142	802.29500000	9.24270000	0.00000000
143	802.82440000	6.64220000	0.00000000
144	806.04320000	0.00000000	0.00000000
145	827.38280000	7.62510000	0.00000000
146	828.95780000	0.00000000	0.00000000
147	835.64430000	12.86770000	0.00000000
148	837.21070000	0.00000000	0.00000000
149	852.61320000	3.38200000	0.00000000
150	852.78010000	0.00000000	0.00000000
151	862.13380000	5.34710000	0.00000000
152	862.18570000	0.00010000	0.00000000
153	865.05540000	6.16690000	0.00000000
154	865.10370000	0.14410000	0.00000000
155	870.45340000	0.00070000	0.00000000
156	870.52190000	17.54970000	0.00000000
157	871.17010000	19.80360000	0.00000000
158	877.31820000	0.00920000	0.00000000
159	877.35530000	0.78760000	0.00000000
160	880.25640000	0.00000000	0.00000000
161	902.69550000	11.55200000	0.00000000
162	902.76560000	0.00010000	0.00000000
163	925.09870000	6.07400000	0.00000000
164	925.12950000	0.00010000	0.00000000
165	933.81380000	3.46530000	0.00000000
166	933.83040000	7.84400000	0.00000000
167	958.80580000	1.37880000	0.00000000
168	958.88590000	1.18910000	0.00000000
169	959.39600000	3.67440000	0.00000000
170	959.47380000	0.25520000	0.00000000
171	982.82150000	1.02810000	0.00000000
172	982.85110000	0.13570000	0.00000000
173	983.40780000	0.68330000	0.00000000
174	983.44000000	0.11250000	0.00000000
175	993.23100000	1.12260000	0.00000000
176	993.23760000	0.03010000	0.00000000
177	1019.36200000	100.13130000	0.00000000
178	1019.37170000	2.17860000	0.00000000
179	1019.79470000	5.98680000	0.00000000
180	1019.82670000	0.01380000	0.00000000
181	1029.62590000	33.16410000	0.00000000
182	1029.66590000	0.00060000	0.00000000
183	1041.51160000	108.00920000	0.00000000
184	1041.57360000	0.40500000	0.00000000
185	1042.65400000	1.71090000	0.00000000
186	1042.66190000	15.91220000	0.00000000
187	1063.81370000	1.73910000	0.00000000
188	1063.82320000	0.00010000	0.00000000

189	1066.43900000	16.87880000	0.00000000
190	1066.44630000	42.26930000	0.00000000
191	1072.87100000	31.80790000	0.00000000
192	1072.93760000	0.06900000	0.00000000
193	1073.57790000	23.41110000	0.00000000
194	1073.65000000	0.17870000	0.00000000
195	1094.12520000	18.78270000	0.00000000
196	1094.16220000	5.40150000	0.00000000
197	1121.16490000	0.00030000	0.00000000
198	1121.30760000	61.17190000	0.00000000
199	1121.73020000	96.73750000	0.00000000
200	1122.41540000	0.00030000	0.00000000
201	1143.38040000	23.42260000	0.00000000
202	1143.41260000	0.03280000	0.00000000
203	1147.32860000	4.12400000	0.00000000
204	1147.34710000	0.28940000	0.00000000
205	1153.40170000	6.96350000	0.00000000
206	1153.44590000	0.00090000	0.00000000
207	1172.93760000	0.00020000	0.00000000
208	1173.47000000	26.84690000	0.00000000
209	1173.92100000	24.29160000	0.00000000
210	1175.06980000	0.00010000	0.00000000
211	1187.37780000	0.00320000	0.00000000
212	1187.41260000	39.54640000	0.00000000
213	1187.69290000	18.77630000	0.00000000
214	1187.94210000	0.00080000	0.00000000
215	1188.75090000	9.25190000	0.00000000
216	1188.76950000	0.20120000	0.00000000
217	1239.13930000	0.04930000	0.00000000
218	1239.14480000	1.50990000	0.00000000
219	1247.84430000	1.94390000	0.00000000
220	1247.86510000	0.04970000	0.00000000
221	1259.97510000	6.83670000	0.00000000
222	1259.98510000	7.12980000	0.00000000
223	1274.19450000	73.03660000	0.00000000
224	1274.59100000	0.00070000	0.00000000
225	1275.73710000	437.29800000	0.00000000
226	1276.17100000	0.16450000	0.00000000
227	1281.77190000	306.13650000	0.00000000
228	1282.61150000	0.00120000	0.00000000
229	1301.80080000	0.00540000	0.00000000
230	1301.95580000	112.12750000	0.00000000
231	1302.66950000	18.55370000	0.00000000
232	1302.69440000	0.00130000	0.00000000
233	1305.32640000	0.01440000	0.00000000
234	1305.48230000	187.58830000	0.00000000
235	1314.15130000	4.52400000	0.00000000
236	1314.17530000	15.74180000	0.00000000
237	1326.14440000	0.00510000	0.00000000
238	1326.61670000	56.22960000	0.00000000
239	1326.75890000	0.11750000	0.00000000
240	1326.78420000	74.38600000	0.00000000
241	1326.79560000	33.95490000	0.00000000
242	1327.30750000	0.00300000	0.00000000
243	1329.53550000	16.30460000	0.00000000
244	1329.62640000	0.00760000	0.00000000
245	1337.94070000	0.03820000	0.00000000
246	1337.94340000	0.70030000	0.00000000
247	1345.63950000	0.84560000	0.00000000
248	1345.65180000	1.22340000	0.00000000
249	1356.57840000	0.28500000	0.00000000
250	1356.60320000	1.31260000	0.00000000
251	1381.59890000	0.00480000	0.00000000
252	1387.64410000	406.66100000	0.00000000

253	1395.24760000	174.27040000	0.00000000
254	1395.65430000	0.02720000	0.00000000
255	1396.09720000	161.79290000	0.00000000
256	1398.58450000	0.00080000	0.00000000
257	1399.68450000	91.02240000	0.00000000
258	1401.53520000	0.00440000	0.00000000
259	1401.89760000	8.65390000	0.00000000
260	1402.92470000	0.00190000	0.00000000
261	1414.64740000	1.23760000	0.00000000
262	1414.64990000	2.38850000	0.00000000
263	1419.37050000	17.11950000	0.00000000
264	1419.41480000	0.10730000	0.00000000
265	1426.13750000	2.17780000	0.00000000
266	1426.15260000	23.86290000	0.00000000
267	1430.02000000	67.36710000	0.00000000
268	1430.06740000	0.00010000	0.00000000
269	1467.49190000	80.66800000	0.00000000
270	1472.64060000	36.75080000	0.00000000
271	1475.34150000	0.00080000	0.00000000
272	1475.68010000	0.00150000	0.00000000
273	1475.94670000	8.08070000	0.00000000
274	1476.15740000	0.00400000	0.00000000
275	1489.82830000	0.04780000	0.00000000
276	1489.87580000	14.67580000	0.00000000
277	1489.96560000	9.82880000	0.00000000
278	1490.09070000	0.00650000	0.00000000
279	1492.75690000	163.55000000	0.00000000
280	1493.33250000	0.05140000	0.00000000
281	1493.79420000	139.71380000	0.00000000
282	1499.67230000	11.83930000	0.00000000
283	1499.72390000	0.03860000	0.00000000
284	1501.67180000	3.72540000	0.00000000
285	1501.67330000	11.29450000	0.00000000
286	1502.44250000	16.03730000	0.00000000
287	1502.48550000	0.16700000	0.00000000
288	1504.70400000	2.99920000	0.00000000
289	1504.72500000	0.03120000	0.00000000
290	1508.13500000	45.86230000	0.00000000
291	1508.96650000	0.00060000	0.00000000
292	1509.93030000	212.62080000	0.00000000
293	1509.99760000	0.18590000	0.00000000
294	1511.97050000	15.99650000	0.00000000
295	1515.45270000	0.00020000	0.00000000
296	1516.29320000	99.56340000	0.00000000
297	1517.15820000	0.04850000	0.00000000
298	1517.25760000	14.27590000	0.00000000
299	1527.29030000	0.00000000	0.00000000
300	1528.71530000	54.54090000	0.00000000
301	1529.13870000	0.00020000	0.00000000
302	1530.35010000	27.21360000	0.00000000
303	1543.29640000	0.00090000	0.00000000
304	1544.92250000	0.00090000	0.00000000
305	1617.72830000	46.56610000	0.00000000
306	1617.87190000	26.19800000	0.00000000
307	1618.00410000	0.00790000	0.00000000
308	1618.12370000	0.00620000	0.00000000
309	1636.66610000	103.97320000	0.00000000
310	1636.91650000	94.27950000	0.00000000
311	1637.23520000	0.00160000	0.00000000
312	1637.57310000	0.00200000	0.00000000
313	1647.99850000	113.22730000	0.00000000
314	1648.42510000	0.22810000	0.00000000
315	2986.17700000	44.79770000	0.00000000
316	2986.22020000	0.48970000	0.00000000

317	2994.77940000	67.40160000	0.00000000
318	2994.80560000	0.91040000	0.00000000
319	2996.13340000	3.02560000	0.00000000
320	2996.14000000	8.61110000	0.00000000
321	2999.19100000	82.53640000	0.00000000
322	2999.22840000	0.34710000	0.00000000
323	3011.61830000	14.49010000	0.00000000
324	3011.63920000	26.70350000	0.00000000
325	3011.70320000	116.17900000	0.00000000
326	3011.74620000	2.03990000	0.00000000
327	3016.16360000	4.24170000	0.00000000
328	3016.16650000	3.97250000	0.00000000
329	3018.32220000	45.96940000	0.00000000
330	3018.32680000	4.32930000	0.00000000
331	3019.24760000	108.87740000	0.00000000
332	3019.27060000	0.22410000	0.00000000
333	3020.27520000	29.62160000	0.00000000
334	3020.28970000	0.15570000	0.00000000
335	3024.32530000	9.99210000	0.00000000
336	3024.34110000	31.75430000	0.00000000
337	3030.72210000	10.30230000	0.00000000
338	3030.75450000	91.19140000	0.00000000
339	3034.95910000	62.92920000	0.00000000
340	3034.98200000	27.52590000	0.00000000
341	3037.86450000	18.38340000	0.00000000
342	3037.87600000	23.56080000	0.00000000
343	3043.05710000	8.28460000	0.00000000
344	3043.07120000	19.65150000	0.00000000
345	3063.25970000	0.00010000	0.00000000
346	3063.27670000	61.90120000	0.00000000
347	3076.43220000	68.88260000	0.00000000
348	3076.44960000	24.36960000	0.00000000
349	3077.27270000	0.13030000	0.00000000
350	3077.28350000	0.36160000	0.00000000
351	3080.74010000	27.42370000	0.00000000
352	3080.77590000	62.45780000	0.00000000
353	3081.67730000	2.18550000	0.00000000
354	3081.70210000	202.73290000	0.00000000
355	3086.48160000	2.14650000	0.00000000
356	3086.48900000	76.95290000	0.00000000
357	3093.91260000	69.55630000	0.00000000
358	3093.94480000	5.29000000	0.00000000
359	3166.32190000	13.10770000	0.00000000
360	3166.32340000	0.08600000	0.00000000
361	3166.35630000	11.61280000	0.00000000
362	3166.36060000	0.01540000	0.00000000
363	3185.04820000	38.79440000	0.00000000
364	3185.06270000	0.45160000	0.00000000
365	3185.30430000	35.57610000	0.00000000
366	3185.32410000	1.51260000	0.00000000
367	3202.92110000	12.07830000	0.00000000
368	3202.96270000	4.24940000	0.00000000
369	3203.20750000	14.03830000	0.00000000
370	3203.22780000	0.53520000	0.00000000
371	3210.82800000	18.94860000	0.00000000
372	3210.85930000	0.96080000	0.00000000
373	3212.11230000	18.68870000	0.00000000
374	3212.13890000	0.38720000	0.00000000
375	3747.16850000	5.62630000	0.00000000
376	3747.20760000	62.52470000	0.00000000
377	3836.82480000	33.14590000	0.00000000
378	3836.94040000	115.29240000	0.00000000
